# Altered liver sinusoidal endothelial cells in MASLD and their evolution following lanifibranor treatment

**DOI:** 10.1016/j.jhepr.2025.101366

**Published:** 2025-02-22

**Authors:** Pierre-Emmanuel Rautou, Shivani Chotkoe, Louise Biquard, Guillaume Wettstein, Denise van der Graaff, Yao Liu, Joris De Man, Christophe Casteleyn, Sofie Thys, Winnok H. De Vos, Pierre Bedossa, Michael P. Cooreman, Martine Baudin, Jean-Louis Abitbol, Philippe Huot-Marchand, Lucile Dzen, Miguel Albuquerque, Pierre Broqua, Jean-Louis Junien, Luisa Vonghia, Manal F. Abdelmalek, Wilhelmus J. Kwanten, Valérie Paradis, Sven M. Francque

**Affiliations:** 1 Université Paris-Cité, Inserm, Centre de recherche sur l'inflammation, Paris, France; 2 AP-HP, Hôpital Beaujon, Service d'Hépatologie, DMU DIGEST, Centre de Référence des Maladies Vasculaires du Foie, FILFOIE, ERN RARE-LIVER, Clichy, France; 3 Department of Gastroenterology and Hepatology, Antwerp University Hospital, Antwerp, Belgium; 4 Laboratory of Experimental Medicine and Paediatrics, University of Antwerp, Antwerp, Belgium; 5 INVENTIVA, Daix, France and New York, NY, USA; 6 Department of Hepatology, Beijing Hospital of Traditional Chinese Medicine, Capital Medical University, Beijing, China; 7 Department of Morphology, Imaging, Orthopedics, Rehabilitation and Nutrition, Faculty of Veterinary Medicine, Ghent University, Merelbeke, Belgium; 8 Comparative Perinatal Development, Department of Veterinary Sciences, Faculty of Pharmaceutical, Biomedical and Veterinary Sciences, University of Antwerp, Antwerp, Belgium; 9 Laboratory of Cell Biology and Histology, University of Antwerp, Antwerp, Belgium; 10 Antwerp Centre for Advanced Microscopy (ACAM), University of Antwerp, Antwerp, Belgium; 11 μNEURO, Centre of Excellence, University of Antwerp, Antwerp, Belgium; 12 Liverpat, Paris, France; 13 Division of Gastroenterology and Hepatology, Mayo Clinic, Rochester, MN, USA; 14 AP-HP, Hôpital Beaujon, Department of Pathology, FHU MOSAIC, Clichy, France

**Keywords:** Intrahepatic vascular resistance, Vascular biology, LSECs, CD34, MASH, Liver fibrosis, Liver inflammation, PPAR, lanifibranor

## Abstract

**Background & Aims:**

Data on changes in liver sinusoidal endothelial cells (LSECs) in patients with metabolic dysfunction-associated steatotic liver disease (MASLD) and their response to treatment are limited. This study aimed at determining (i) features associated with LSEC capillarisation in patients with MASLD; (ii) whether LSEC changes can regress with the pan-peroxisome proliferator-activated receptor (PPAR) agonist lanifibranor; (iii) the role of the different PPAR isotypes on LSEC changes in MASLD.

**Methods:**

We analysed CD34 expression, a marker of LSEC capillarisation, on liver biopsies from patients considered for inclusion in the NATIVE trial at baseline (n = 249), and after 24 weeks of placebo or lanifibranor (n = 173). Two rat models of MASLD were used to investigate the effect of lanifibranor or of mono-PPAR agonists on LSECs.

**Results:**

Lobular CD34 staining was more intense in patients with isolated steatosis than in those with no MASLD (52% *vs.* 10%; *p* = 0.03). In the overall cohort, this staining was more intense in patients with metabolic dysfunction-associated steatohepatitis (MASH) than in those without (63% *vs.* 41%; *p* = 0.01) and strongly correlated with liver fibrosis and to a lesser extent with liver inflammation. Lanifibranor treatment was associated with more common improvement in CD34 periportal staining (*p* = 0.025), and less frequent worsening of lobular staining (*p* = 0.028). Compared with healthy rats, rats with MASLD had higher CD34 staining, portal venous pressure, intrahepatic vascular resistance, and impaired liver endothelial function. Lanifibranor normalised or strongly improved these abnormalities, whereas mono-PPAR agonists caused partial improvements.

**Conclusions:**

In patients, LSEC capillarisation was increased at the earliest stages of MASLD and was associated with liver fibrosis and inflammation. In both patients and rats with MASLD, lanifibranor treatment was associated with improvement in liver endothelial phenotype.

**Impact and implications:**

Data on changes in liver sinusoidal endothelial cells (LSECs) in patients with metabolic dysfunction-associated steatotic liver disease (MASLD) and their response to treatment are limited. This study demonstrates that LSEC capillarisation is already present in the lobular zone of the liver of patients and rats at the stage of isolated steatosis, before metabolic dysfunction-associated steatohepatitis (MASH) onset, and progresses with liver fibrosis, and to a lesser extent with liver inflammation. Lanifibranor treatment, a pan-peroxisome proliferator-activated receptor agonist currently tested in a phase III clinical trial, improves LSEC capillarisation but also intrahepatic vascular resistance and portal pressure in MASLD. Targeting LSECs appears to be a promising approach to improve MASH.

## Highlights

• LSEC capillarisation – particularly in the lobular zone – is associated with liver fibrosis and to a lesser extent inflammation in MASH.
• Lanifibranor treatment dose-dependently reduces LSEC capillarisation in the periportal zone.
• LSEC capillarisation is present in preclinical models of early MASLD.
• Lanifibranor improves both LSEC dysfunction and LSEC capillarisation in preclinical models of MASLD.

## Graphical abstract

**Figure:**
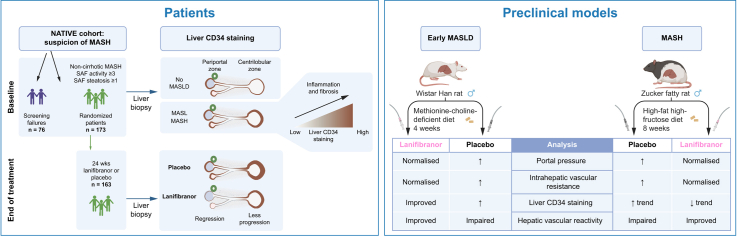

## Introduction

Metabolic dysfunction-associated steatotic liver disease (MASLD), formerly known as non-alcoholic fatty liver disease (NAFLD), is defined as the presence of steatosis (*i.e.* abnormal hepatic triglyceride accumulation) in >5% of hepatocytes according to histological analysis, in the presence of cardiometabolic risk factors.^1^ MASLD encompasses a spectrum of different conditions, including isolated steatosis (metabolic dysfunction-associated steatotic liver [MASL]) and metabolic dysfunction-associated steatohepatitis (MASH).^2^^,^^3^ Contrary to MASL, MASH can progress to cirrhosis and hepatocellular carcinoma. It is estimated that 25% of the adult population worldwide has MASLD, increasing concomitantly with the global obesity epidemic.^4^

The current view of MASH pathogenesis focuses on hepatocytic alterations with metabolic and lipotoxic stresses leading to cell death and the onset of liver inflammation.[5], [6], [7], [8] However, an increased intrahepatic vascular resistance (IHVR) has repetitively been documented, both in humans[9], [10], [11], [12] and in preclinical models,[13], [14], [15], [16], [17], [18], [19] as an early event in MASLD, significantly contributing to the progression of the disease through impaired intrahepatic blood flow and subsequent hepatic hypoxia.[20], [21], [22] Liver sinusoidal endothelial cells (LSECs) play a central role in the vascular aspects of liver diseases, including in MASH. In chronic liver diseases, capillarisation, that is loss of fenestrae and development of a basal membrane, is an important feature because it is associated with the loss of the antifibrotic and the anti-inflammatory properties of LSECs.^13^^,^^23^ In MASLD, animal data suggested that LSECs undergo capillarisation already at the stage of MASL, before MASH onset.^24^ A small human study did not confirm this finding in patients.^25^ This study included only 29 patients and did not consider the potential spatial distribution of capillarisation (which might coincide with the perisinusoidal fibrosis, typically found in the lobular area in MASH).[25], [26], [27]

Despite its prevalence and potential severity, therapeutic options for MASH are currently limited. A thyroid hormone receptor beta-selective agonist has recently been approved,^28^ and several other candidate molecules are being investigated in phase II and III trials.^29^ Among those, peroxisome proliferator-activated receptor (PPAR) agonists have sparked major interest as therapeutic agents for MASH, given the pleiotropic roles of the three PPAR isotypes (α, β/δ, γ) in the regulation of energy metabolism, fibrosis, and inflammation, but also their role in endothelial cells and their impact on IHVR in models of portal hypertension.[30], [31], [32] However, the effect of PPAR agonists on the hepatic vasculature in early MASLD and MASH has not been investigated.

In the present study we analysed liver biopsies from 249 patients with a suspicion of MASH screened for inclusion in the NATIVE clinical trial,^31^ aiming at identifying clinical and histological features associated with LSECs capillarisation and its spatial distribution in MASLD, and at assessing whether LSEC changes in patients can regress following treatment with the pan-PPAR agonist lanifibranor. We further investigated the role of the different PPAR isotypes (and their therapeutic potential) on the structural and functional aspects of the altered hepatic vascular biology, including LSEC changes, in two animal models of early MASLD, with the aim to understand the role of these vascular changes in disease progression. We also examined the effects of the combined pan-PPAR approach, and its potential benefit compared to mono-PPAR agonists.

## Materials and methods

### Clinical approaches

#### Clinical study design and patients

The clinical section of the present work is an ancillary study of the NATIVE study (funded by Inventiva Pharma; NATIVE ClinicalTrials.gov number, NCT03008070), a phase IIb, double-blind, randomised, placebo-controlled trial evaluating the efficacy and safety of lanifibranor in patients with biopsy-proven non-cirrhotic MASH with severe disease activity.^33^^,^^34^ NATIVE was approved by independent ethical committees and appropriate authorities in all 16 countries where at least one patient underwent randomisation and complies with the declaration of Helsinki (Table S1).^33^ All patients gave written informed consent.

Between February 2018 and July 2019, a total of 868 adult patients presenting with a suspicion of MASH were screened. They underwent a liver biopsy if none was obtained in the preceding 6 months. These biopsies will be designated hereafter as obtained at baseline. Classification of the liver lesions on histology (no MASL, MASL, MASH) was performed according to the Steatosis-Activity-Fibrosis (SAF) scoring system and algorithm described by Bedossa *et al.*^26^ Patients meeting the study eligibility criteria for whom the liver biopsy confirmed the presence of MASH without cirrhosis, with a SAF activity score ≥3 and SAF steatosis score ≥1, were included in the NATIVE trial (n = 247) and randomised in a 1:1:1 ratio to receive placebo, 800 mg, or 1,200 mg lanifibranor orally once daily for 24 weeks.^31^^,^^33^ At the end of the treatment period, patients underwent another liver biopsy. These patients are hereafter referred to as ‘randomised patients’. The 621 patients who did not meet these criteria are referred to as ‘screening failures’, including 297 patients with a liver biopsy of sufficient quality but with non-inclusion criteria; 207 of these 297 patients had available unstained liver slides. Among them, we selected 76 patients to obtain a balanced distribution of mild, moderate, and severe steatosis, MASH activity and fibrosis, as detailed in Table S2.

#### Histological scoring of density of CD34 positive vessels

Analysis of CD34 staining was performed by two approaches: (i) a semiquantitative approach based on a three-tier grading system according to the extent of the sinusoidal CD34 positivity, performed by an expert pathologist (VP) unaware of the patients groups, separating periportal and lobular zones, with lobular score of 1 indicating CD34 positivity restricted to the centrolobular area and lobular score of 2 extending to mediolobular and with periportal score of 1 indicating CD34 positivity restricted to the periportal area and periportal score of 2 extending to mediolobular; and (ii) an automatically quantitative approach, defined as the number of vessels per unit of area (μm^2^) (Fig. S1). Co-staining with erythroblast transformation-specific related gene (ERG)^35^ confirmed endothelial localisation of the CD34 staining (Fig. S1).^35^ More details are provided in the Supplementary **Materials and methods.**

### Preclinical approaches

#### Animal models

We used two rat models to represent two stages of the disease. Early MASLD was modelled by feeding male Wistar Han rats with a methionine-choline-deficient diet (MCDD) for 4 weeks, to induce severe steatosis in the absence of MASH.^16^^,^^17^ To study the intrahepatic effects of the different PPAR isotypes, rats (n = 6–8/group) underwent gavage once a day (QD) with either placebo (1% methylcellulose + 0.05% poloxamer), fenofibrate (PPAR-α agonist, 30 mg/kg), GW501516 (PPAR-β/δ agonist, 10 mg/kg), rosiglitazone (PPAR-γ agonist, 5 mg/kg), or lanifibranor^36^ (balanced pan-PPAR agonist, not directly affecting other pathways than PPARs; 100 mg/kg), during the entire 4 weeks of diet as a preventive treatment (Fig. S2A). The doses of the mono-PPAR agonists were chosen because their potency and efficacy for their respective nuclear receptors were similar to that of lanifibranor for the same respective nuclear receptor.

To exclude model specificity and to examine the effects at the stage of steatohepatitis rather than isolated steatosis, the most important results of lanifibranor were tested in a second model considered a more clinically relevant representation, that is male Zucker fatty rats (ZFR, fa/fa) were fed a high-fat high-fructose diet (HFHFD) (Fig. S2B), and compared with Zucker lean rats (ZLR) fed a chow diet (CD)**.** More details are provided in the Supplementary **Materials and methods.**

#### Histology, CD34 immunohistochemistry, *in vivo* haemodynamics, *in situ ex vivo* liver perfusion, and vascular corrosion casting

Details are provided in the Supplementary **Materials and methods**.

### Statistical analyses

Details are provided in the Supplementary **Materials and methods**.

## Results

### Patients

Between February 2018 and July 2019, 868 patients with a suspicion of MASH were screened and 247 patients were randomised in the NATIVE study. Among the latter, 173 patients had baseline liver tissue available for CD34 staining, including 163 with liver tissue also available at week 24. These 173 patients were selected for the present study. Among the 297 patients not randomised in NATIVE study, but with available liver tissue remaining from the screening period, 76 were selected for the present study (Fig. S3). These 173 and 76 patients will be designated hereafter as the whole baseline cohort (N = 249). The main characteristics of all patients at baseline are summarised in Table S3.

### LSEC CD34 staining according to the presence of MASL or MASH

**Fig. 1:**
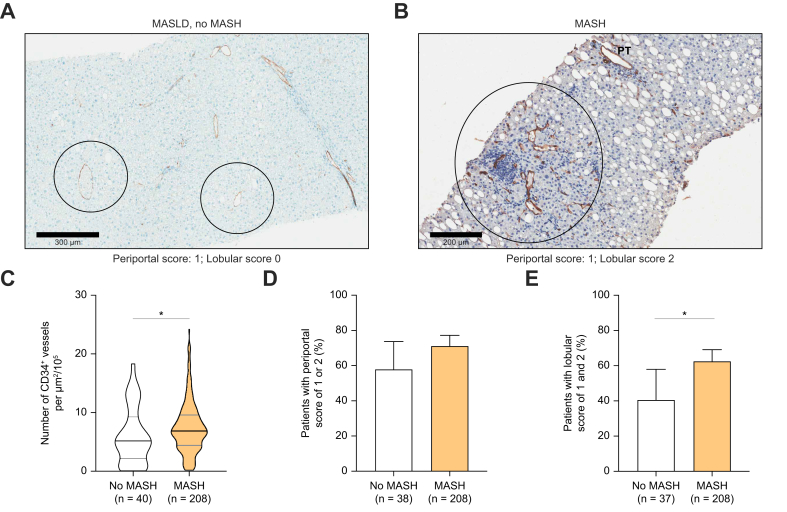
CD34 staining is more pronounced in patients with MASH than in patients without MASH (classified according to the SAF algorithm^26^), particularly in the lobular area. Density of CD34-positive vessels, periportal score, and lobular score were available in 248, 246 and 245 patients, respectively, as detailed in Fig. S3. (A,B) Representative images of CD34 staining with corresponding periportal and lobular score of patients with no MASH and MASH, respectively. (C) Density of CD34-positive vessels is displayed for patients without MASH and with MASH. Percentage of patients with, respectively, periportal (D) and lobular (E) score of 1 or 2 is displayed. Lobular areas are circled. For violin plots, the bars represent the median ± IQR, otherwise bars represent 95% CIs. ∗*p* <0.05. The Wilcoxon-Mann-Whitney *U* test, the Χ^2^ test, or Fisher test was used when appropriate. Patients’ numbers vary between graphs because vessel density, periportal score, and lobular score were unavailable for technical and staining quality reasons for, respectively, one, three, and four patients out of 249. MASH, metabolic dysfunction-associated steatohepatitis; PT, portal tracts; SAF, Steatosis-Activity-Fibrosis score.

Immunohistological staining for CD34 was performed as illustrated in Fig. 1A and B. We observed that the manual semiquantitative assessments of CD34 staining performed by the expert pathologist were strongly correlated with the automatic quantification of the density of CD34-positive vessels, attesting the consistency of these evaluations (Fig. S4).

**Fig. 2:**
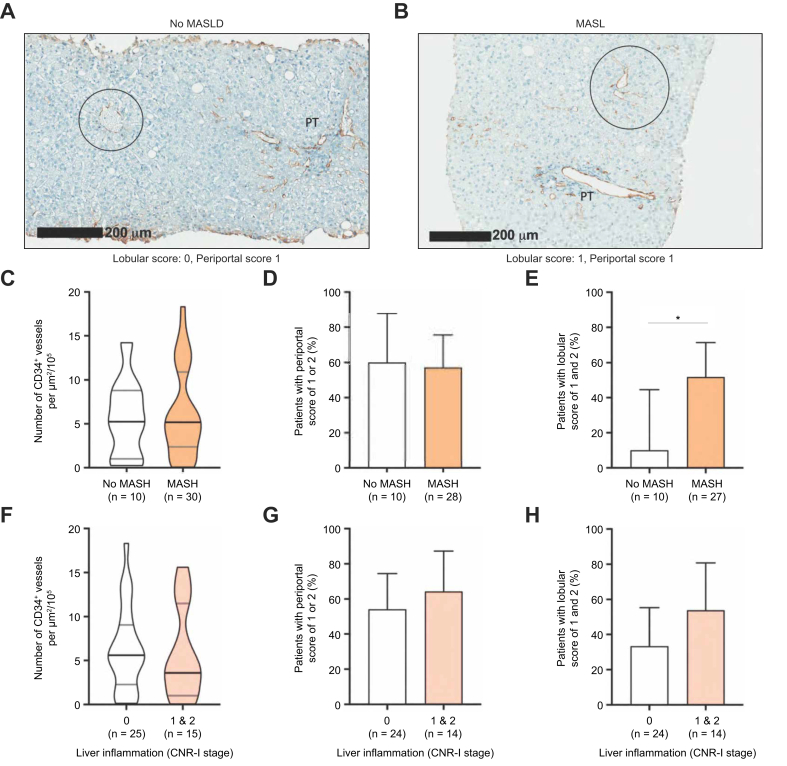
Comparison of CD34 staining between patients with isolated steatosis (MASL, but no MASH) and without MASLD (according to the SAF algorithm^26^). Density of CD34-positive vessels, periportal score, and lobular score were available in 40, 38, and 37 patients without MASH, respectively, as detailed in Fig. S3. (A,B) Representative images of CD34 staining with corresponding periportal and lobular score of patients with no MASLD and MASL, respectively. (C) Density of CD34 positive vessels is displayed for patients without MASLD and with MASL. Percentage of patients with, respectively, periportal (D) and lobular (E) score of 1 or 2 is displayed. Density of CD34-positive vessels (F), periportal score (G), and lobular score (H) is displayed according to the level of liver inflammation (CRN-I stages). The Wilcoxon-Mann-Whitney *U* test was used. Lobular areas are circled. For violin plots, the bars represent the median ± IQR, otherwise bars represent 95% CIs. Patients’ numbers vary between graphs because vessel density, periportal score, and lobular score were unavailable for technical and staining quality reasons for, respectively, one, three, and four patients out of 249. ∗*p* <0.05. CRN, Clinical Research Network; MASL, metabolic dysfunction-associated steatotic liver; MASLD, metabolic dysfunction-associated steatotic liver disease; MASH, metabolic dysfunction-associated steatohepatitis; PT, portal tracts; SAF, Steatosis-Activity-Fibrosis score.

Of the 249 patients from the whole baseline cohort, 209 had MASH (40 had no MASH and were obviously recruited out of the NATIVE screening failures). When comparing liver CD34 staining in patients without MASH and with MASH, we observed that patients with MASH had a higher density of CD34 positive vessels (Fig. 1C, *p* <0.05) and a higher rate of CD34 staining in the lobular area (Fig. 1E, *p* <0.05), but not in the periportal area (Fig. 1D). Interestingly, when focusing on the no-MASH group (n = 40), we observed that lobular CD34 staining was more common in patients with MASL than in those with normal histology (Fig. 2A–E). There was no association in patients without MASH between CD34 staining and liver inflammation (Fig. 2F–H).

### LSEC CD34 expression, localisation, and liver histology in MASLD

**Fig. 3:**
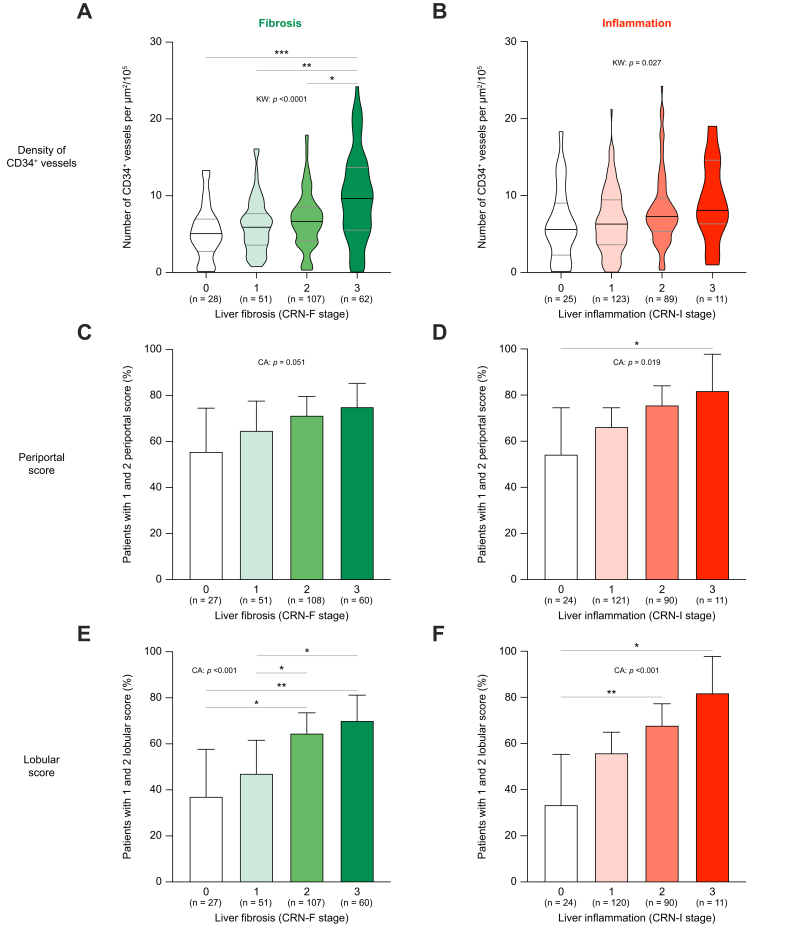
Relationship between CD34 staining level and localisation and histological features of MASLD in 249 patients with a suspicion of MASH. Density of CD34-positive vessels, periportal score, and lobular score were available in 248, 246, and 245 patients, respectively, as detailed in Fig. S3. Baseline density of CD34-positive vessels is displayed according to fibrosis (CRN-F grade) (A) and according to inflammation (CRN-I grade) (B). Percentage of patients with periportal score for CD34 staining of 1 or 2 is displayed according to fibrosis (CRN-F grade) (C) and according to inflammation (CRN-I grade) (D). Percentage of patients with lobular score for CD34 staining of 1 or 2 is displayed according to fibrosis (CRN-F grade) (E) and according to inflammation (CRN-I grade) (F). When appropriate, Kruskal–Wallis, and *post hoc* Dunn’s tests were performed between all columns, with ∗*p* <0.05; ∗∗*p* <0.01; ∗∗∗*p* <0.001. For violin plots, the bars represent the median ± IQR, otherwise bars represent 95% CIs. Patients’ numbers vary between graphs because vessel density, periportal score, and lobular score were unavailable for technical and staining quality reasons for, respectively, one, three, and four patients out of 249. CA, Cochran–Armitage; CRN, Clinical Research Network; KW, Kruskal–Wallis; MASL, metabolic dysfunction-associated steatotic liver; MASLD, metabolic dysfunction-associated steatotic liver disease; MASH, metabolic dysfunction-associated steatohepatitis; PT, portal tracts; SAF, Steatosis-Activity-Fibrosis score.

In the whole baseline cohort, the density of CD34-positive vessels was strongly linked with liver fibrosis (*p* <0.001) (Fig. 3A) and liver inflammation (*p* = 0.027) using the NASH Clinical Research Network (CRN) scoring system^37^ instead of the SAF-score,^26^ because of a more granular score for inflammation ranging from 0 to 3 (Fig. 3B). Periportal staining and lobular staining were also associated with liver fibrosis and liver inflammation, although the association was more pronounced for lobular staining (Fig. 3C–F). No association was found between CD34 staining and steatosis or ballooning (Fig. S5).

### LSEC CD34 expression and clinical and laboratory features

**Fig. 4:**
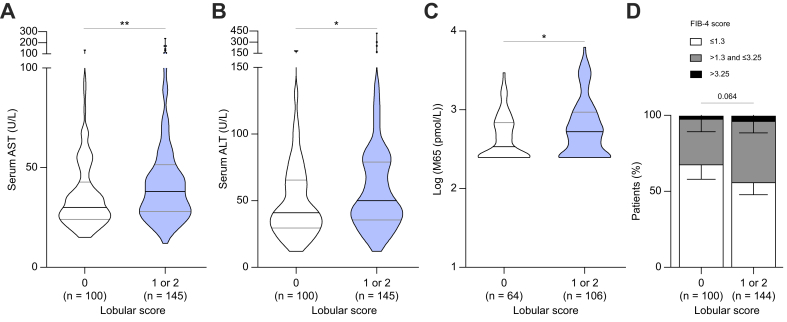
Association of CD34 staining in the lobular area with clinical features. CD34 lobular score was available 245 patients, as detailed in Fig. S3. On the analysis population (screening failure and randomised patients), serum AST (A), serum ALT (B), cytokeratin 18 M65 fragments (C) and FIB-4 score (D) were significantly increased in patients with CD34 lobular staining score of 1 or 2. The Wilcoxon-Mann-Whitney *U* test, the Χ^2^ test, or Fisher test was used when appropriate. For violin plots, the bars represent the median ± IQR, otherwise bars represent 95% CIs. ∗*p* <0.05; ∗∗*p* <0.01. AST, aspartate aminotransferase; ALT, alanine aminotransferase; FIB-4, Fibrosis-4.

We then aimed at identifying what clinical and laboratory features are linked to LSEC CD34 staining. We found statistically significant positive correlations between the density of CD34-positive vessels and serum aspartate aminotransferase (AST), serum γ-glutamyltransferase, Fibrosis-4 (FIB-4) score^38^ and liver stiffness measurement using vibration-controlled transient elastography (FibroScan®, Echosens, Paris, France), but with Spearman correlation coefficients <0.212, indicating only weak correlations (Table S4). There was no association between periportal CD34 staining and clinical or laboratory features (Table S4), whereas patients with lobular CD34 staining had higher serum AST (Fig. 4A), serum alanine aminotransferase (ALT) (Fig. 4B), cytokeratin 18 M65 (a marker of cell death) (Fig. 4C) and a trend towards higher FIB-4 (Fig. 4D) than patients without lobular CD34 staining.

### Effect of lanifibranor on LSEC capillarisation

**Fig. 5:**
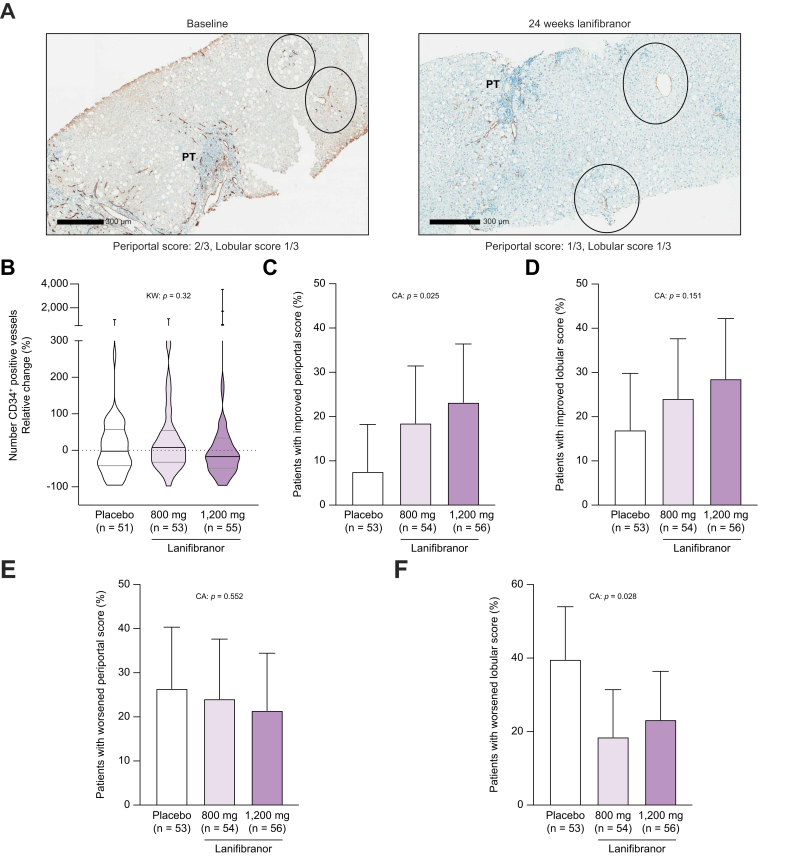
Effect of lanifibranor treatment on CD34 staining. (A) Representative images of CD34 staining of a patient treated with 1,200 mg lanifibranor at baseline (left) and after 24 weeks of treatment (right). (B) Relative change of density of CD34 positive vessels according to treatment group in randomised patients. (C,D) Percentage of patients with improved (decreased by minimum 1 stage) periportal score (C) and lobular score (D) at week 24. (E,F) Percentage of patients with worsened (increased by minimum 1 stage) periportal score (E) and lobular score (F) at week 24. For violin plots, the bars represent the median ± IQR, otherwise bars represent 95% CIs. CA, Cochran–Armitage; KW, Kruskal–Wallis.

In patients with MASH randomised into the NATIVE trial, 24-week treatment with lanifibranor at both dosages had no effect on the density of CD34-positive vessels (Fig. 5A). Improvement in periportal score was more common in patients treated with lanifibranor than in those treated with placebo, with a dose–response effect (placebo: 7.5%. lanifibranor 800 mg: 18.5%. lanifibranor 1,200 mg: 23.2%. *p* = 0.025, Fig. 5C). Patients with lanifibranor treatment had less worsening of lobular score (placebo: 39.6%, lanifibranor 800 mg: 18.5%, lanifibranor 1,200 mg: 23.2%; *p* = 0.028, Fig. 5F).

### Animal models

#### Early MASLD

**Fig. 6:**
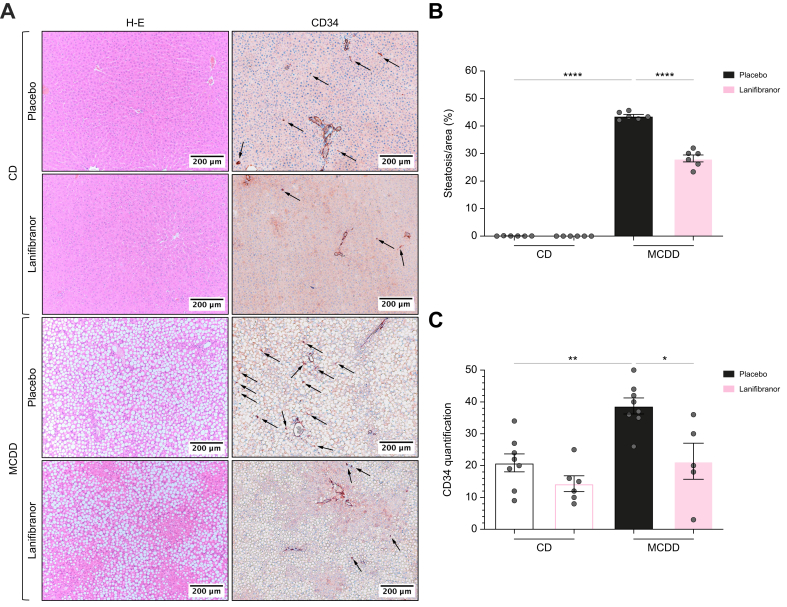
Assessment of steatosis and LSEC capillarisation in early MASLD. Eight-week-old male Wistar Han rats (n = 6–8/group) were either fed a chow diet (CD) or a methionine-choline-deficient diet (MCDD) for 4 weeks and preventively treated with either placebo or lanifibranor (100 mg/kg) daily QD via oral gavage. (A) Images of H&E-stained and CD34-stained liver tissue sections (Olympus BX43, microscope lens 10 × /0.45 NA Plan Apo; resolution 1 pixel = 0.442 μm). (B) Steatosis quantification defined as fraction of macrovesicular fat droplets per area (%). (C) Blinded CD34 semiquantification. Data were analysed using two-way ANOVA followed by the *post hoc* Tukey test and presented as mean ± standard error of the mean with ∗*p* <0.05; ∗∗*p* <0.01; ∗∗∗∗*p* <0.0001. Arrows indicate CD34-positive staining. LSEC, liver sinusoidal endothelial cell; MASLD, metabolic dysfunction-associated steatotic liver disease.

After 4 weeks of MCDD, animals developed marked hepatomegaly (liver/total body weight [TBW] ratio): MCDD 4.6 (4.4–5.0) *vs*. CD 3.0 (2.9–3.2), *p* <0.001; Table 1; Fig. S6, Table S5). At histology, placebo-treated rats fed an MCDD had severe grade 3 steatosis with no microscopic indications of liver inflammation or ballooning (Fig. 6A), nor fibrosis (Fig. S7A). Likewise, rats fed an MCDD had no liver collagen-1 protein expression (Fig. S7B). Fenofibrate induced full inhibition of steatosis in MCDD rats (Fig. S8) despite a significant increase in liver volume (Fig. S6). GW501516 reduced steatosis area by 8.3% (absolute reduction compared with 43.7% in MCDD with placebo), *p* <0.01. Rosiglitazone resulted in a slight reduction of 3.7% (Fig. S8). Lanifibranor improved steatosis in MCDD rats compared to the placebo group, mainly in the centrilobular zones, with a reduction in steatosis area of 15.4%, *p* <0.0001 (Fig. 6B).

**Table 1:** Baseline characteristics, haemodynamics and pressures in an early MASLD rat model.

Parameter	Groups (n)	Chow diet		MCDD	
		Placebo	Lanifibranor	Placebo	Lanifibranor
**Demographic characteristics**					
Age (weeks)	58/38/64/38	8	8	8	8
Body weight (g): baseline	50/38/50/38	251.0 (242.8; 263.3)	249.5 (243.0; 257.0)	251.0 (242.8; 263.3)	250.0 (243.5; 258.0)
Body weight (g): after 4 weeks	58/38/62/38	349.0 (328.3; 360.3)∗	338.5 (326.3; 373.0)^‡^	210.0 (202.0; 219.3)	211.0 (203.8; 215.0)
Δ Weight (g)	50/38/50/38	92.5 (72.0; 104.5)∗	87.0 (69.3; 103.3)^‡^	-43.5 (-50.0; -38.75)	-43.5 (-46.0; -35.0)
Liver weight (g)	54/38/60/37	10.5 (9.3; 11.3)	10.4 (9.6; 11.1)	10.5 (9.3; 11.3)	9.7 (9.2; 11.1)
% liver/total body weight	56/38/60/37	3.0 (2.9; 3.2)∗	3.0 (2.9; 3.2)^‡^	4.6 (4.4; 5.1)	4.7 (4.4; 5.1)

**Haemodynamics and pressures**					
MABP (mmHg)	43/31/40/37	123.7 (106.3; 130.8)	110.7 (101.1; 122.1)^‡^	121.9 (115.3; 132.3)	92.7 (78.1; 104.1)∗
*In vivo* PVP (mmHg)	46/31/40/37	3.5 (3.2; 3.9)∗	3.3 (3.1; 3.7)	5.6 (5.1; 6.4)	3.7 (3.2; 4.0)∗
Portal blood flow (ml/min)	38/28/34/34	12.5 (11.0; 15.1)∗	13.8 (12.1; 15.1)^‡^	10.1 (9.2; 11.3)	10.8 (9.2; 12.7)
THPG (mmHg) at 10 ml/min	8/8/7/8	3.7 ± 0.1∗	3.5 ± 0.1	4.8 ± 0.2	3.5 ± 0.1∗
THPG (mmHg) at 30 ml/min	8/8/7/8	6.6 ± 0.2∗	7.4 ± 0.4	8.3 ± 0.4	6.3 ± 0.3∗

**Histological parameters; H-E, PSR**					
Steatosis grade^§^	6/6/6/6	0 (0; 0)∗	0 (0; 0)^‡^	3 (3; 3)	3 (3; 3)
Lobular inflammation grade^¶^		0 (0; 0)	0 (0; 0)	0 (0; 0)	0 (0; 0)
Ballooning grade∗∗		0 (0; 0)	0 (0; 0)	0 (0; 0)	0 (0; 0)
Fibrosis stage^††^		0 (0; 0)	0 (0; 0)	0 (0; 0)	0 (0; 0)
NAS		0 (0; 0)∗	0 (0; 0)^‡^	3 (3; 3)	3 (3; 3)

**Histological parameters; CD34 staining**					
CD34 quantification	6/6/6/6	20.9 ± 2.8∗	14.3 ± 2.5	38.8 ± 2.5	21.4 ± 5.7∗

Male Wistar Han rats of 8 weeks old (n = 6–8/group) were either fed a chow diet (CD) or a methionine-choline-deficient diet (MCDD) for 4 weeks and simultaneously treated with either placebo or lanifibranor (100 mg/kg) daily QD via oral gavage. Pooled data were analysed using the Kruskal–Wallis test followed by the Dunn test and presented as median (IQR). The THPG data were analysed using a generalised estimating equation model followed by least significant difference *post hoc* testing when appropriate. ∗For comparison with MCDD + placebo; ^†^CD + lanifibranor *vs.* CD + placebo; ^‡^CD + lanifibranor *vs.* MCDD + lanifibranor; ∗^,†,‡^*p* <0.05. ^§^Steatosis was assessed as the percentage of hepatocytes containing large and medium-sized intracytoplasmic lipid droplets and graded as 0 (<5%), 1 (5–33%), 2 (34–66%), or 3 (≥67%), according to the non-alcoholic steatohepatitis Clinical Research Network (NASH CRN) grading system. ^¶^Lobular inflammation was classified as grade 0 (no foci), grade 1 (<2 foci per 200 × field) or grade 2 (2–4 foci per 200 × field), according to the NASH CRN scoring system. ∗∗Ballooning was classified as grade 0 (no balloon hepatocyte) grade 1 (few but definite ballooned hepatocytes) or grade 2 (prominent ballooning), according to the NASH CRN grading system. ^††^Fibrosis was classified as stage F0 (no fibrosis), stage F1 (mild fibrosis), stage F2 (significant fibrosis), stage F3 (advanced fibrosis), or stage F4 (cirrhosis), according to the SAF–NASH CRN staging system. MABP, mean arterial blood pressure; NAS, NAFLD Activity Score; PVP, portal venous pressure; SAF, Steatosis-Activity-Fibrosis score; THPG, transhepatic pressure gradient.

#### MASH

After 8 weeks of diet and treatment (Fig. S2B), in placebo groups, both TBW and liver weight were significantly increased in HFHFD-fed ZFRs compared with lean controls (Table 2). Histology demonstrated that ZLRs on a CD had normal liver histology without any steatosis, ballooning, inflammation, or fibrosis (Figs S9 and S10), whereas in ZFRs borderline MASH was present (NAFLD Activity Score [NAS] = 0.0 (0.0–0.25) in ZRLs *vs.* 3.0 (3.0–4.3) in ZFRs, *p* <0.001). Out of all animals, one had definite MASH (NAS score = 5), whereas five had borderline MASH (NAS = 3–4). The animals with MASH had mild steatosis (7.0 ± 1.5% *vs.* 0.04 ± 0.0% in ZLRs, *p* <0.001, Fig. S9B), ballooning (Fig. S9A) and inflammation as well, without the development of fibrosis (Fig. S10A). Likewise, rats with MASH had no liver collagen-1 protein expression (Fig. S10B). In HFHFD-fed ZFRs, lanifibranor even further increased TBW and decreased liver/TBW ratio. However, there was no difference in liver weight compared with placebo-treated HFHFD-fed ZFRs (Table 2; Fig. S11). At histology, lanifibranor tended to decrease the NAS score (from 3.0 [3.0–4.3] to 2.0 [1.8 – 3.3]), however not significantly. Steatosis decreased from 7.0 ± 1.5% to 2.3 ± 0.5% (Fig. S9B, *p* <0.004), mainly in the lobular area, while in the periportal area microvesicular steatosis was still present together with macrovesicular steatosis and a tendency of little to no ballooning (from 1.0 [1.0–1.5] to 0.0 [0.0–1.0]) (Fig. S9A). HFHFD-fed ZFRs treated with placebo had a significantly increased spleen weight compared with ZLRs. Treatment with lanifibranor caused minimal insignificant decrease of the spleen weight in ZFRs (Table 2; Fig. S11D).

**Table 2:** Baseline characteristics, haemodynamics, and pressures in a rat model of MASH.

Parameter	Groups (n)	Chow diet		HFHFD	
		Placebo	Lanifibranor	Placebo	Lanifibranor
**Demographic characteristics**					
Age (weeks)	30/29/30/30	8	8	8	8
Body weight (g): baseline	30/29/30/30	146.0 ± 5.2∗	137.0 ± 4.5^‡^	219.0 ± 8.02	208.0 ± 6.0
Body weight (g): after 8 weeks	30/29/30/29	372.0 ± 5.2∗	370.2 ± 5.6^‡^	691.9 ± 10.36	803.0 ± 14.9∗
Δ Body Weight(g)	30/28/30/29	220.0 ± 8.2∗	234.0 ± 6.0^‡^	469.6 ± 14.76	592.0 ± 17.6∗
Liver weight (g)	29/29/28/28	11.4 (10.1; 12.0)^∗,^^†^	11.0 (10.1; 11.8)^†,^^‡^	25.4 (23.4; 28.4)^†^	25.5 (24.1; 27.5)^†^
% liver/total body weight	29/29/28/28	3.0 (2.8; 3.2)^∗,^^†^	2.8 (2.8; 3.1)^†,^^‡^	3.7 (3.4; 4.1)^†^	3.1 (3.0; 3.6)^∗,^^†^
Spleen weight (mg)	6/5/6/6	555.0 ± 41.1∗	618.0 ± 29.1	802.0 ± 40.0	689.0 ± 56.4
% spleen/total body weight	6/5/6/6	0.15 ± 0.01∗	0.16 ± 0.03^‡^	0.12 ± 0.00	0.09 ± 0.01∗

**Haemodynamics and pressures**					
MABP (mmHg)	19/18/19/16	133.0 ± 2.7∗	129.0 ± 3.2	159.0 ± 2.5	133.0 ± 3.0∗
*In vivo* portal pressure (mmHg)	19/18/19/16	4.9 ± 0.3∗	4.9 ± 0.4	7.1 ± 0.2	5.2 ± 0.2∗
Portal blood flow (ml/min)	19/20/19/14	19.4 ± 0.8	19.6 ± 0.7^‡^	21.5 ± 0.8	26.2 ± 1.5∗
THPG (mmHg) at 20 ml/min	8/8/8/7	5.0 ± 0.2∗	4.9 ± 0.1	6.3 ± 0.2	4.9 ± 0.2∗
THPG (mmHg) at 30 ml/min	8/8/8/7	6.3 ± 0.3∗	6.2 ± 0.1	7.7 ± 0.2	6.4 ± 0.1∗

**Histological parameters; H-E, PSR**					
Steatosis grade^§^	6/5/6/6	0 (0; 0)^∗,^^†^	0 (0; 0)^†,‡^	1 (1; 2)^†^	1 (1; 1.3)^†^
S0 – no.		6	5	0	0
S1 – no.		0	0	4	5
S2 – no.		0	0	2	1
S3 – no.		0	0	0	0
Lobular inflammation grade^¶^		0 (0; 0.25)^∗,^^†^	0 (0; 1)^†^	1 (1; 1.3)^†^	1 (0; 1)^†^
I0 – no.		5	3	0	2
I1 – no.		1	2	5	4
I2 – no.		0	0	1	0
I3 – no.		0	0	0	0
Ballooning grade∗∗		0 (0; 0)^∗,^^†^	0 (0; 0)^†^	1 (1; 1.3)^†^	0 (0; 1)^†^
B0 – no.		6	5	0	4
B1 – no.		0	0	5	2
B2 – no.		0	0	1	0
Fibrosis stage^††^		0 (0; 0)^†^	0 (0; 0)^†^	0 (0; 0)^†^	0 (0; 0)^†^
NAS		0 (0; 0.3)^∗,^^†^	0 (0; 1)^†^	3 (3; 4.3)^†^	2 (1.8; 3)^†^
**Histological parameters; CD34 staining**					
CD34 quantification	6/5/6/6	21.3 ± 3.1	16.2 ± 4.7	28.0 ± 4.8	22.0 ± 2.2

Eight-week-old male Zucker fatty rats fed a high-fat high-fructose diet (HFHFD) and 8-week-old male Zucker lean rats fed a chow diet (CD) were concomitantly treated with either placebo or lanifibranor (100 mg/kg) daily QD via oral gavage during the whole period of 8 weeks of diet. Data were analysed using two-way ANOVA followed by the *post hoc* Tukey test and presented as mean ± standard error of the mean or the Kruskal–Wallis test followed by the Dunn test and presented as median (IQR). The THPG data were analysed using a generalised estimating equation model followed by least significant difference *post hoc* testing when appropriate. ∗For comparison with HFHFD + placebo; ^†^CD + lanifibranor *vs.* CD + placebo; ^‡^CD + lanifibranor *vs.* HFHFD + lanifibranor; ∗^,†,‡^*p* <0.05. ^§^Steatosis was assessed as the percentage of hepatocytes containing large and medium-sized intracytoplasmic lipid droplets and graded as 0 (<5%), 1 (5–33%), 2 (34–66%), or 3 (≥67%), according to the non-alcoholic steatohepatitis Clinical Research Network (NASH CRN) grading system. ^¶^Lobular inflammation was classified as grade 0 (no foci), grade 1 (<2 foci per 200 × field) or grade 2 (2–4 foci per 200 × field), according to the NASH CRN scoring system. ∗∗Ballooning was classified as grade 0 (no balloon hepatocyte) grade 1 (few but definite ballooned hepatocytes) or grade 2 (prominent ballooning), according to the NASH CRN grading system. ^††^Fibrosis was classified as stage F0 (no fibrosis), stage F1 (mild fibrosis), stage F2 (significant fibrosis), stage F3 (advanced fibrosis), or stage F4 (cirrhosis), according to the SAF–NASH CRN staging system. MABP, mean arterial blood pressure; NAS, NAFLD Activity Score; PVP, portal venous pressure; SAF, Steatosis-Activity-Fibrosis score; THPG, transhepatic pressure gradient.

### LSEC CD34 staining

#### Capillarisation in early MASLD

We then assessed liver CD34 staining. In placebo-treated groups, MCDD-fed rats presented twice more CD34 staining than control rats (Fig. 6A and C). In MCDD-fed rats, fenofibrate, GW501516, and lanifibranor almost normalised CD34 staining, whereas treatment with rosiglitazone had minimal effect (Fig. S12). Of note, treatment with all mono-agonists and lanifibranor showed a trend towards decreased CD34 staining in CD-fed rats, however, with no statistical significance (Fig. 6; Fig. S12, Table S6).

#### Capillarisation in MASH

In placebo-treated animals, there was a non-significant trend towards increased CD34 staining in MASH livers compared to control livers. Lanifibranor, also insignificantly, tended to lower the CD34 staining in control livers as well as in MASH livers (Table 2; Fig. S9A and B).

### *In vivo* haemodynamics and pressures

#### Early MASLD

**Fig. 7:**
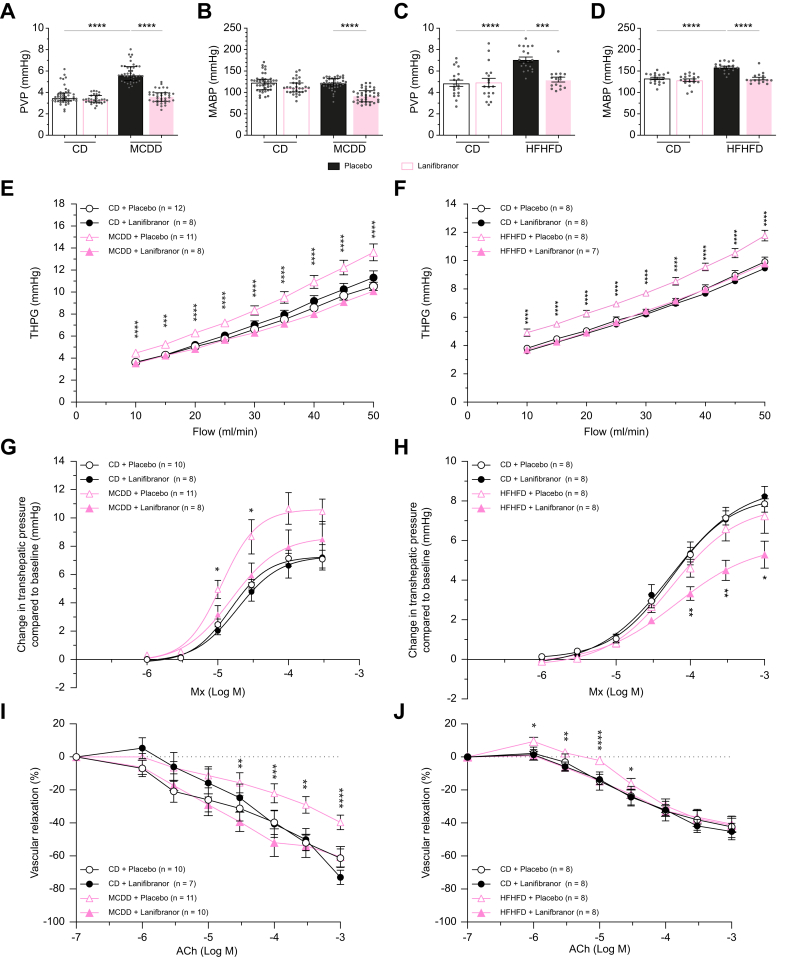
*In vivo* haemodynamics and pressures assessment, and *in situ ex vivo* liver perfusions in two preclinical models of MASLD. Eight-week-old male Wistar Han rats (n = 7–12/group per experiment) were either fed a chow diet (CD) or a methionine-choline-deficient diet (MCDD) for 4 weeks and preventively treated with either placebo or lanifibranor (100 mg/kg) daily QD via oral gavage. *In vivo* parameters: (A) PVP, (B) MABP. *Ex vivo* parameters: (E) THPG, (G) dose–response Mx, (I) dose–response ACh. Model 2: 8-week-old male Zucker fatty rats (n = 7–8/group per experiment) fed a high-fat high-fructose diet (HFHFD) and 8-week-old male Zucker lean rats (n = 8/group per experiment) fed a chow diet (CD) were concomitantly treated with either placebo or lanifibranor (100 mg/kg) daily QD via oral gavage during the complete period of 8 weeks of diet. *In vivo* parameters: (C) PVP, (D) MABP. *Ex vivo* parameters: (F) THPG, (H) dose–response Mx, (J) dose–response ACh. Pooled *in vivo* data (n = 16–43/group) were analysed using two-way ANOVA (C,D) followed by the *post hoc* Tukey test and presented as mean ± standard error of the mean or Kruskal–Wallis test (A,B) followed by the Dunn test and presented as median (IQR). The THPG and vascular relaxation data were analysed using a generalised estimating equation model followed by least significant difference *post hoc* testing. Data are presented as mean ± SEM. ∗*p* <0.05; ∗∗*p* <0.01; ∗∗∗*p* <0.001; ∗∗∗∗*p* <0.0001. For clarity only the comparisons with MCDD-placebo are shown in perfusion graphs. ACh, acetylcholine; Log M, logarithmic concentration in mol/L; MABP, mean arterial blood pressure; MASLD, metabolic dysfunction-associated steatotic liver disease; Mx, methoxamine; PVP, portal venous pressure; QD, once per day; THPG, transhepatic pressure gradient.

In placebo-treated animals, MCDD-fed rats had a significantly higher portal venous pressure (measured as described in the Supplementary **Materials and methods)** compared with CD rats. The values were 5.6 (5.1–6.4) and 3.5 (3.2–3.9) mmHg, respectively, *p* <0.0001 (Fig. 7A; Table 1). In CD rats, none of the drugs induced a change in portal vein pressure (PVP) measurements. In MCDD-fed rats, all mono-PPAR agonists tended to decrease PVP, however, only fenofibrate caused a significant decrease (Fig. S13). Lanifibranor had a more pronounced effect (*p* <0.0001) compared with the mono-agonists and completely normalised the PVP in MCDD rats. Besides the impact on PVP, lanifibranor and also GW501516 to a lesser extent, decreased the mean arterial blood pressure in MCDD rats (Fig. 7B and S14A), while fenofibrate and rosiglitazone did not (Table 1; Fig. S14A, Table S5). There was no difference in pulse rate (Fig. S14D) nor in caudal cava vein pressure (CCVP) between the groups (data not shown).

#### MASH

In line with our findings in the MCDD model of steatosis, in ZFR after 8 weeks of HFHFD, the PVP was significantly increased compared to controls (MASH 7.1 ± 0.2 mmHg *vs.* controls 4.9 ± 0.2 mmHg, *p* <0.0001, Fig. 7C, Table 2). Lanifibranor caused a significant decrease of PVP in ZFR from 7.1 ± 0.2 mmHg to 5.2 ± 0.2 mmHg, *p* < 0.001. Besides the impact on PVP, the mean arterial pressure, that was elevated as well, decreased with lanifibranor treatment (Fig. 7D). The systolic and diastolic pressures were both decreased by lanifibranor (Fig. S15A and B). No differences in pulse rate (Fig. S15D) and CCVP were observed between the groups (data not shown).

### *In situ ex vivo* liver perfusion: assessment of the intrahepatic vascular resistance

#### Early MASLD

In line with the *in vivo* data, the transhepatic pressure gradient (THPG) in MCDD-fed rats was significantly elevated at every perfusion flow velocity compared with CD-fed rats in placebo-treated groups (Fig. 7E; Fig. S16A). Fenofibrate significantly improved the THPG to normal values in MCDD rats at low perfusion flows, but this effect decreased at higher flows (Fig. S16B). Both GW501516 and rosiglitazone did not significantly improve the THPG in MCDD rats (Fig. S16C and D). Treatment with lanifibranor induced no changes in the THPG measurements in control rats, but it normalised the values at all flow rates in MCDD rats (Fig. 7E). Data points at 10 and 30 ml/min can be found in Table 1 and Table S5.

#### MASH

The THPG in placebo-treated ZFR was significantly increased at all flows compared with controls, and ZFR treated with lanifibranor during 8 weeks of HFHFD demonstrated normalisation of the increased THPG (Fig. 7F). Data points at 20 and 30 ml/min can be found in Table 2.

### Dose–response experiments; hepatic vascular reactivity

#### Endothelin pathway

Endothelin-1 (ET-1) showed a dose-dependent increase of the THPG both in placebo-treated CD-fed animals and MCDD-fed animals, with a significantly increased responsiveness to ET-1 in MCDD compared with CD animals (Fig. S17, Table S7). Although fenofibrate increased ET-1 responsiveness, GW501516 and rosiglitazone considerably decreased ET-1 response, but this effect diminishes at the higher dose (Fig. S17B–D). The hyperreactivity to ET-1 was barely decreased with lanifibranor treatment (Fig. S17E). As lanifibranor did not relevantly alter ET-1 reactivity, experiments were not validated in HFHFD-fed ZFR rats.

#### Alpha-1 adrenergic pathway (methoxamine)

##### Early MASLD

In line with previous data,^17^ MCDD-fed animals showed a significantly increased vascular reactivity (Emax) to methoxamine compared to CD, but with no difference in sensitivity (EC_50_) to the drug (Fig. 7G; Table S8). All three mono-agonists (Fig. S18B–D) as well as lanifibranor decreased methoxamine hyperreactivity in MCDD animals close to control values (Fig. 7G).

##### MASH

In contrast to our findings in the MCDD model of steatosis, the HFHFD-fed ZFR were not hyperresponsive to methoxamine. However, lanifibranor significantly decreased Mx responsiveness below control values in HFHFD-fed ZFR without affecting the responses in CD rats (Fig. 7H; Table S9).

#### Muscarinergic pathway (acetylcholine)

##### Early MASLD

After pre-constriction with 3 × 10^-5^ mol/L methoxamine, the vasodilatory response to acetylcholine (ACh) was overall blunted in a dose-dependent manner in MCDD rats compared with control rats in placebo-treated groups (Fig. 7I; Table S10). Both fenofibrate and GW501516 did not improve impaired responsiveness to acetylcholine, while rosiglitazone improved acetylcholine hyporeactivity only at the highest doses (Fig. S19B–D). Lanifibranor improved the reactivity of ACh to control values in MCDD rats (Fig. 7I).

##### MASH

In HFHFD-fed ZFR, after pre-constriction with 1.5 × 10^-4^ mol/L methoxamine, the vasodilatory response to ACh was blunted at lower doses. Treatment with lanifibranor normalised acetylcholine hyporeactivity in HFHFD-fed ZFR (Fig. 7J; Table S11).

### Vascular corrosion casting; 3D structure of liver sinusoids

In the placebo-treated CD group (Fig. S20A), examination of liver vascular corrosion casts using scanning electron microscopy unveiled a regular arrangement of sinusoids within lobules, characterised by small sinusoids with even diameters. In contrast, the MCDD-fed group exhibited a disruption of this regular sinusoidal pattern, resulting in a disarrayed network of vessels. Within this irregular arrangement, the sinusoids demonstrated uneven and enlarged diameters. Furthermore, a notable observation was the presence of numerous vessels that branched into *cul-de-sac*-like dilated vessel stumps, commonly referred to as blebs (Fig. S20B). Fenofibrate treatment substantially improved the sinusoidal organisation in MCDD-fed rats, with a more regular arrangement of the sinusoids comparable to control animals, and a considerable decrease of the number of blebs (Fig. S20C). GW501516 (Fig. S20D) induced minor improvements, whereas rosiglitazone had no noticeable effect, yielding images comparable to placebo-treated MCDD rats (Fig. S20E). Lanifibranor induced (heterogeneously distributed) improvements of the sinusoidal organisation compared with placebo-treated steatotic rat livers, with more regular and untangled patterns, and more small sinusoids with diameters resembling those of controls (Fig. S20F).

## Discussion

In this study, we demonstrated in a large cohort of patients with MASLD that CD34 staining, representing LSEC capillarisation, appears already at the stage of MASL – hence before MASH onset – and increases with the severity of MASLD, being strongly linked to liver fibrosis, and to a lesser extent to liver inflammation, and that it regresses following treatment with the pan-PPAR agonist lanifibranor.^33^ Then, using two animal models of MASLD, we extended those results and showed that the beneficial effect of lanifibranor on LSEC capillarisation was accompanied by a functional improvement attested by a normalisation of portal pressure and of intrahepatic vascular resistance. The effect of lanifibranor was more pronounced than that of single PPAR agonists.

A first major observation in the present study is that CD34 LSEC expression is higher in the lobular area in patients with MASL than in those with no MASLD. Such an early change in LSEC phenotype is also observed here in rats fed a MCDD and had been previously reported in mouse models,^24^ but had not been observed in patients to date. A previous study including 39 patients covering the MASLD spectrum did not reveal any difference in global CD34 staining between patients with MASL and those without MASLD, which is in line with our observation when only global CD34 staining was compared.^25^ The increase in lobular CD34 expression between both groups appeared in our study when we refined the quantification by considering the zonation of CD34 staining. It is well known that the LSEC phenotype differs between periportal and lobular areas in the healthy liver.^39^^,^^40^ We can thus speculate that lobular LSECs are particularly sensitive to stimuli derived from the portal vein in a context of metabolic syndrome, like excessive dietary macronutrients or gut microbiota-derived products, leading to early capillarisation in that area.^13^ This localisation of LSEC capillarisation, confined at the early stages of MASLD to the lobular area, is reminiscent of the zone 3 perisinusoidal fibrosis typical for MASLD.^26^^,^^27^ Although normal LSECs are known to maintain hepatic stellate cell quiescence, capillarised LSECs lose this ability, thus allowing fibrosis deposition.^13^ Our observations thus reinforce the hypothesis that LSEC capillarisation occurs in MASLD before the development of liver fibrosis and contributes to its development^13^ and are also in line with the observations of endothelial dysfunction early in the disease course.^16^^,^^41^

A second major finding of this study is a strong link between liver CD34 staining and liver fibrosis – and to a lesser extent liver inflammation – observed in 249 patients covering the whole MASLD spectrum (except for MASH cirrhosis), consistent with the associations between liver CD34 staining and FIB-4 (a marker of liver fibrosis), serum AST, ALT, and cytokeratin 18 M65 (a marker of cell death) concentrations observed in the same patients. In cohorts of 37 and 39 patients, higher CD34 staining has been described in patients with MASH as compared with MASL,^25^^,^^42^ further increasing with the stage of liver fibrosis.^42^ The present study not only firmly establishes those associations, but also identifies that they are much more pronounced for lobular than for periportal CD34 staining. Importantly, our large patient population allows us to confidently rule out a link between CD34 staining and hepatocyte ballooning or steatosis, suggesting that drivers for LSEC capillarisation might not be derived from hepatocytes but possibly rather from circulating cells or mediators present in the portal blood in the context of metabolic syndrome. Dedicated studies would be needed to investigate this hypothesis.

A third major finding is that CD34 staining can regress with MASH treatment – in this case lanifibranor – after only 24 weeks in patients, which is in line with our preclinical observations. The rapid regression of CD34 staining observed in the present study contrasts with data obtained in patients with hepatitis C virus-related cirrhosis where CD34 staining remained unchanged 5 years after sustained virological response.^43^ A first explanation for these differences could be that the patients included in the present study were at a less advanced stage of their liver disease, as none had cirrhosis. Indeed, capillarisation might regress more quickly at earlier stages of the liver disease. Another explanation could be that the observed effect is related to the drug itself, as PPARs regulate endothelial function and phenotype,[44], [45], [46] besides glucose and fatty acid metabolism.^5^ Lanifibranor (IVA337) is a pan-PPAR agonist that has a moderate and well-balanced activity on the three PPAR isoforms, thereby addressing the different components of MASH.^36^^,^^47^ Investigations carried out in the present study, using two rat models of MASLD, support that view of an effect of lanifibranor on endothelial function and phenotype and extend the previous demonstration of a beneficial effect of lanifibranor in animal models of cirrhosis.^32^ Indeed, we observed that only lanifibranor was able to normalise the IHVR, as attested by normalisation of PVP and of the *ex vivo* measured THPG, whereas PPARs mono-agonists showed only partial improvements. This effect of lanifibranor was accounted for by a restoration of liver endothelial function rather than to an effect on liver steatosis that was only partially improved. This vascular effect of lanifibranor might explain the significant results of this drug on both MASH resolution and fibrosis regression after only 24 months of treatment, compared with the absence of efficacy in terms of fibrosis regression by the PPAR-γ agonist pioglitazone or the glucagon-like peptide 1 receptor agonist semaglutide after 1.5 years of treatment.^48^

A final interesting finding is that lanifibranor decreased the mean arterial blood pressure (MABP) in MASLD animals. This appeared to be mainly a PPAR-β/δ agonistic effect. Interestingly, the MABP lowering effect by lanifibranor was also observed in patients during the NATIVE 2b trial.^31^ Patients with MASH usually have cardiometabolic alterations including hypertension, and treatments that improve their cardiometabolic health can be beneficial beyond a pure liver-centred benefit.^34^ This needs, however, further confirmation in the ongoing phase III NATiV3 trial (NCT04849728).

In conclusion, this study showed that LSEC capillarisation occurred in patients already at the stage of simple steatosis, just as in animal models. In patients, LSEC capillarisation further increased with MASH, and was strongly associated with liver fibrosis and to a lesser extent inflammation, but regressed following treatment with the pan-PPAR agonist lanifibranor. Lanifibranor also normalised PVP and IHVR in rats with early MASLD as well as in those with MASH, mainly by improving functional alterations, but also structural vascular alterations. The effect of lanifibranor was more pronounced than that of mono-PPAR agonists.

## Abbreviations

ACh, acetylcholine; AST, aspartate aminotransferase; ALT, alanine aminotransferase; CCVP, caudal cava venous pressure; CD, chow diet; CRN, Clinical Research Network; EC_50_, half maximal effective concentration; Emax, maximum effect; EGR, erythroblast transformation-specific related gene; ET-1, endothelin-1; FIB-4, Fibrosis-4; HFHFD, high-fat high-fructose diet; IHVR, intrahepatic vascular resistance; LSECs, liver sinusoidal endothelial cells; MABP, mean arterial blood pressure; MASL, metabolic dysfunction-associated steatotic liver; MASLD, metabolic dysfunction-associated steatotic liver disease; MASH, metabolic dysfunction-associated steatohepatitis; MCDD, methionine-choline-deficient diet; Mx, methoxamine; NAFLD, non-alcoholic fatty liver disease; NAS, NAFLD Activity Score; PPARs, peroxisome proliferator-activated receptors; PVP, portal venous pressure; QD, once a day; SAF, Steatosis-Activity-Fibrosis score; TBW, total body weight; THPG, transhepatic pressure gradient; ZFR, Zucker fatty rat; ZLR, Zucker lean rat.

## Financial support

This study was funded by 10.13039/100016915Inventiva Pharma. P-ER’s research laboratory is supported by the Fondation pour la Recherche Médicale (10.13039/501100002915FRM EQU202303016287), ‘Institut National de la Santé et de la Recherche Médicale’ (ATIP AVENIR), by the ‘Agence Nationale pour la Recherche’ (ANR-18-CE14-0006-01, RHU QUID-NASH, ANR-18-IDEX-0001, ANR-22-CE14-0002), by ‘Émergence, Ville de Paris’, by 10.13039/501100004097Fondation ARC (R23087HH), by the European Union’s 10.13039/501100007601Horizon 2020 research and innovation programme under grant agreement No 847949, and by France 2030 RHU LIVER-TRACK (ANR-23-RHUS-0014). SMF holds a senior clinical investigator fellowship from the Research Foundation Flanders (FWO) (1802154N). WDV holds investigator fellowships from the FWO (I000123N, I003420N).

## Authors’ contributions

Conceptualisation (lead): P-ER, VP, SMF. Conceptualisation (equal): SC, J-LJ, WJK. Visualization (lead): SC. Investigation (lead): SC. Investigation (equal): YL, CC, ST, PB, PH-M, LD, MFA. Methodology (equal): P-ER, SC, LB, DVdG, YL, JDM, CC, ST, WHDV, PB, PH-M, LD, MA, LV, WJK, VP, SF. Data curation (lead): P-ER, SC. Data curation (equal): WJK. Software (equal): WHDV. Formal analysis (lead): P-ER, SC, LB. Supervision (lead): P-ER, WJK, VP, SF. Supervision (equal): DVdG, JDM, LV. Writing – original draft (lead): P-ER, SC, LB. Writing – review and editing (lead): WJK, VP, SF. Writing – review and editing (equal): GW, DVdG, YL, JDM, CC, WHDV, PB, MPC, MB, J-LA, PH-M, LD, PB, J-LJ, LV, MFA. Funding acquisition (lead): P-ER, GW, PB, VP, SF.

## Data availability statement

The data that support the findings of this study are available from the corresponding author, upon reasonable request.

## Conflicts of interest

P-ER has received research funding from Terrafirma and acted as consultant for Mursla, Genfit, Boehringer Ingelheim, Cook, Jazz, and Abbelight, and received speaker fees from AbbVie. SMF has been lecturer for AbbVie, Allergan, Bayer, Eisai, Genfit, Gilead Sciences, Janssens Cilag, Intercept, Inventiva, Merck Sharp & Dome, Novo Nordisk, Promethera, Siemens. He has acted as consultant for AbbVie, Actelion, Aelin Therapeutics, AgomAb, Aligos Therapeutics, Allergan, Astellas, Astra Zeneca, Bayer, Boehringer Ingelheim, Bristoll-Meyers Squibb, CSL Behring, Coherus, Echosens, Eisai, Enyo, Galapagos, Galmed, Genetech, Genfit, Genflow Bio, Gilead Sciences, Intercept, Inventiva, Janssens Pharmaceutica, Julius Clinical, Madrigal, Medimmune, Merck Sharp & Dome, NGM Bio, Novartis, Novo Nordisk, PRO.MED.CS, Promethera, Roche. His institution has received grants from Astellas, Falk Pharma, Genfit, Gilead Sciences, GlympsBio, Janssens Pharmaceutica, Inventiva, Merck Sharp & Dome, Pfizer, Roche. WJK received lecturer fees for the PanNASH initiative and received travel grants from Ipsen and Norgine. He is a co-inventor of a patent on the use of lipopigment imaging for disease (filed by MGH/MIT: US 20190307390). MFA has acted as an advisor for 89Bio, Boehringer Ingelheim, Hanmi, Intercept, Inventiva, Madrigal, and Novo Nordisk. She has received grants (paid to her institution) from 89Bio, Akero, Hamni, Inventiva, Madrigal and Novo Nordisk. She has served as a speaker for MedScape, Chronic Liver Disease Foundation, Clinical Care Options, and Fishawack, Inc.

Please refer to the accompanying ICMJE disclosure forms for further details.
