# Supplementary material for: Altered liver sinusoidal endothelial cells in MASLD and their evolution following lanifibranor treatment
Source: JHEP Rep. 2025 Feb 22;7(6):101366. doi: 10.1016/j.jhepr.2025.101366 (PMC12142333; doi:10.1016/j.jhepr.2025.101366)
Supplement: Multimedia component 1 [file mmc1.pdf]

Altered liver sinusoidal endothelial cells in MASLD and their evolution following lanifibranor treatment

Pierre-Emmanuel Rautou, Shivani Chotkoe, Louise Biquard, Guillaume Wettstein, Denise Van der Graaff, Yao Liu, Joris De Man, Christophe Casteleyn, Sofie Thys, Winnok H. De Vos, Pierre Bedossa, Michael P. Cooreman, Martine Baudin, Jean-Louis Abitbol, Philippe Huot-Marchand, Lucile Dzen, Miguel Albuquerque, Pierre Broqua, Jean-Louis Junien, Luisa Vonghia, Manal F. Abdelmalek, Wilhelmus J. Kwanten, Valérie Paradis, Sven M. Francque

Table of contents

Supplementary materials and methods……………………………………………......2

Supplementary figures………………………………………………………………..….7

Supplementary tables…………………………………………………………….….28

Supplementary references………………………………………………………..….46

Supplementary materials and methods

Clinical approaches

Liver biopsy and immunostaining

Liver biopsy slides were stained (Hematoxylin & Eosin, Picrosirius red staining, Perls staining) in a central histology laboratory. Histologic features of MASLD and MASH were assessed using the NASH Clinical Research Network (NASH CRN) Scoring System [1].

Formalin-fixed paraffin-embedded liver biopsies were subjected to CD34 immunostaining, a marker of LSEC capillarisation. Briefly, tissue sections (3 µm) underwent dewaxing, antigen retrieval, and then incubation with antibody against CD34 (monoclonal mouse anti-human CD34, ab8536, clone QBEnd-10, batch number GR49632-27, Abcam, UK, dilution 1:500) using an automated immunohistochemical stainer according to the manufacturer's guidelines (streptavidin-peroxidase protocol, BenchMark, Ventana). Negative controls were systematically included by incubating with PBS instead of primary antibody. To confirm CD34 colocalisation with endothelial cells, double immunostaining with antibody against Erythroblast transformation-specific related gene (ERG, a nuclear marker of LSECs [2]) (ABCAM, ab92513, dilution 1:50) was performed using Ultraview universal alkaline phosphatase red detection and Ultraview DAB kits (Ventana, Benchmark, USA) in selected cases (n=29).

Histological scoring of density of CD34 positive vessels

Quantitative analysis was assessed on digitised slides using Scanscope AT Turbo (LEICA©, Wetzlar, Germany). Slide scanning was performed at x20 magnification (microscope lens 20X/0.75 NA Plan Apo; resolution 1 pixel=0.5 µm) using the manufacturer's software (Imagescope, Leica). Digital images were analysed with a dedicated microvessel algorithm (HALO, Indica Labs, Albuquerque, US) that quantifies positive pixels (brown stained pixel defined by RGB composite) surrounding white areas **(Fig. S1)**. All images were annotated to exclude large vessels and portal tracts.

Preclinical approaches

Animal models

Early MASLD: Male Wistar Han rats (Charles River, Germany; 200-250 g) were fed a chow diet (CD, ICN Biomedicals SA, Asse, Belgium) or a methionine-choline-deficient diet (MCDD, Envigo RMS B.V., Indianapolis, Indiana USA) for 4 weeks, which is known to induce severe steatosis in the absence of MASH in this rat strain [3, 4]. This model hence recapitulates the liver MASLD phenotype and is suited to study the intrahepatic features of the disease, although it does not recapitulate the classical metabolic syndrome phenotype in which the disease usually develops. To study the intrahepatic effects of the different PPAR isotypes, rats (n = 6-8/group) underwent gavage once a day (QD) with either placebo (1% methylcellulose + 0.05% poloxamer), fenofibrate (PPAR-α agonist, 30 mg/kg), GW501516 (PPAR-β/δ agonist, 10 mg/kg), rosiglitazone (PPAR-γ agonist, 5 mg/kg) or lanifibranor [5] (100 mg/kg), during the entire 4 weeks of diet as a preventive treatment **(Fig. S2A)**. Lanifibranor (IVA337) is a pan-PPAR agonist with a unique molecular design that has a moderate and well-balanced activity on the three PPAR isoforms, thereby addressing the different components of MASH [5, 6]. To evaluate the specificity of lanifibranor for modulating PPAR isoforms, comprehensive off-target profiling for more than 100 targets was previously conducted using CEREP's (Eurofins) advanced screening platform. These tests confirmed that lanifibranor did not exhibit off-target modulation, demonstrating its specificity for PPAR pathways without affecting other molecular targets.

MASH: To exclude model specificity and to examine the effects at the stage of steatohepatitis rather than isolated steatosis, the most important results of lanifibranor were tested in a second model considered a more clinically relevant representation of MASLD: male Zucker fatty rats (ZFR, fa/fa) (Charles River, USA; 8 weeks old, n = 6-8/group) were fed a high-fat high-fructose diet (HFHFD, D16042610, Research Diets, New Brunswick, NJ, USA) and compared to lean control rats (ZLR, fa/+) (Charles River, USA; 8 weeks old, n = 6-8/group) fed a chow diet for 8 weeks. Additionally, ZFR were treated orally with lanifibranor (100 mg/kg) or placebo preventively during the complete 8 weeks of HFHFD **(Fig. S2B).** No treatment-related mortality was observed. All animals were kept in a 12 h:12 h light/dark cycle with controlled temperature, humidity, unlimited access to their food and water in enriched cages of up to two animals. The animals were treated according to the ARRIVE guidelines. The protocol was approved by the Antwerp University Ethical Committee on Animal Experiments (ECD 2021-24). With respect to the ethical guidelines, sample size was calculated with G*Power software considering effect size and variability based on prior data from our own research group and from the literature (specified in ECD 2021-24).

Western blots

Rat liver tissue was homogenised with a Precellys 24 homogeniser (Bertin technologies) in 1x RIPA lysis buffer (ab156034) containing a protease inhibitor cocktail (cOmplete Mine; Roche Diagnostics, Mannheim, Germany) and a phosphatase inhibitor cocktail (PhosSTOP; Roche). Protein concentrations were determined with a bicinchonicic acid assay (BCA). Protein samples of the liver were run on an SDS-polyacrylamide gel (Bolt™ Bis-Tris 4-12%; Life Technologies, Carlsbad (CA), USA) and transferred to a PVDF membrane. Membranes were subsequently blocked in Tris-buffered saline containing 0.1% Tween and 5% non-fat dry milk. Membranes were probed overnight at 4°C with primary antibody solution with the following antibodies: anti-COL1A (sc-59772, Santa Cruz Biotechnology, Germany) and anti-GAPDH (39-8600, Thermo Scientific). After incubation with appropriate horseradish peroxidase secondary antibodies, chemiluminescent signals were obtained with Pierce™ ECL Plus Western Blotting Substrate (32132; Thermo Scientific) using a ChemiDoc™ MP imaging system (Bio-Rad Laboratories). Each lane was loaded with 20 µg protein. Rat tail collagen type 1 (A1048301, Gibco™) served as positive control.

Histology

As the liver perfusion experiments at high flows may ultimately induce manipulation artefacts, separate groups of animals were used in parallel, solely for the purpose of tissue harvesting and analysis, in the same experimental settings. Rats (n = 6/group) were weighed, anaesthetised, and sacrificed. Livers were weighed, random samples were fixed in 4% formaldehyde (BDH Prolabo, VWR, Belgium), and subsequently dehydrated in 60% isopropanol, followed by paraffin-embedding.

5 µm sections were stained with haematoxylin-eosin (H-E) and picrosirius red according to standard laboratory protocols. Histologic features of MASLD and MASH were assessed using the NASH Clinical Research Network (NASH CRN) Scoring System [1]. Further, slides of H-E-stained liver samples were digitised with a Zeiss Axioscan (using a 10X/0.45 NA Plan Apo objective (at an image resolution of 0.442 µm /pixel). Steatosis was measured as the area fraction of a tissue region of fixed size (ROI size: 8.96 mm^2) covered by macrovesicular lipid droplets using ImageJ software (Bethesda, MD, USA). In brief, RGB images were first converted to 8-bit grayscale after which a manual, fixed intensity threshold was applied to only retain the lipid fraction. A subsequent binary watershed procedure was used to separate touching droplets, and a selective analysis of particles with a size in between 50 and 800 $µm^2$ and circularity above 0.5 was performed to obtain a mask of the lipid fraction. The relative area of the ROI covered by the resulting mask (in %) was used as measure for steatosis.

CD34 immunohistochemistry

Liver tissue sections were treated with heat mediated tris-EDTA buffer solution for antigen retrieval. The sections were incubated with rabbit recombinant monoclonal anti-CD34 antibody (1:2000, ab81289, Abcam, San Francisco, USA) in TBS-Tx overnight. After the sections were washed, the biotinylated goat anti-rabbit secondary antibody (1:200, PK-4001 Vectastain ABC-HRP kit, Labconsult, Belgium) in TBS-Tx was applied for 30 min. Subsequently, incubation with HRP-labelled avidin was performed for 60 min. Slides were stained with 3-amino-9-ethylcarbazole (AEC) substrate followed by haematoxylin staining.

Histological scoring of CD34 positive vessels

Analysis of CD34 staining was performed by a manually semi-quantitative approach counting all CD34-positive stains (size ≥ 3 µm) per image (arrows Fig. 6A & 7A) (portal tracts and central veins excluded) acquired with a 10X/0.25 NA Plan Apo objective (0.442 µm/pixel). Liver tissue section slides were blinded and per slide pictures were taken from 5 random fields, imaging 6 livers for each group. All images were acquired with Universal Grab 6.1 software using an Olympus BX43 microscope. The blindly acquired images of CD34 stained sections were scored using ImageJ software (Bethesda, MD, USA).

In vivo haemodynamics and blood pressures

Under general anaesthesia (ketamine-xylazine), a tracheal tube (PE 240 ID 1.67 mm OD 2.42 mm Intramedic Clay Adams brand non-radiopaque polyethylene tubing) was inserted by tracheostomy. The blood flow in the carotid artery, portal vein and caudal caval vein was measured using a 1 PR or 1.5 PR Doppler flow probe and a TS420 flowmeter (Transonic Systems, Inc., Ithaca, NY, USA). After blood flow assessment, a 24G catheter was inserted into the carotid artery. The abdomen was opened by a median incision. The portal vein was exposed and cannulated with a 24G catheter. The caudal cava vein was cannulated with a 22G catheter, which was advanced with the tip into the retrohepatic part of the caudal cava vein. The different catheters were connected to a Gould Statham pressure transducer (model P23 ID, Hato Rey, Puerto Rico). Haemodynamic and blood pressure variables were recorded with in-house monitoring equipment (Powerlab 8/30 and LabChart 7, AD Instruments, Oxford, UK). Carotid artery pressure (mean arterial blood pressure (MABP)), pulse rate (PR), portal venous pressure (PVP) and caudal cava vein pressure (CCVP) were measured.

In situ ex vivo liver perfusion

To investigate the IHVR in the same animals, the transhepatic pressure gradient (THPG) was assessed immediately after the *in vivo* measurements, directly by *in situ ex vivo* liver perfusion experiments (n = 6-8/group), as described previously [4]. Briefly, after the *in vivo* assessments, heparin (1,400 U/kg) was injected intravenously in the caudal cava vein. The portal vein was cannulated with a 14 G catheter, the thoracic cavity was opened and the suprahepatic cava vein was cannulated through the right atrium with a 16 G catheter. Next, the liver was perfused in a single-pass way by oxygenated Krebs-Ringer solution (37°C) and the catheters were connected to the previously mentioned pressure and flow monitoring equipment. After a stabilisation period of 20 min at a constant flow of 10 mL/min, the flow was gradually increased by 5 mL/min every 5 min, from 10 to 50 mL/min. In all experiments, the portal vein (inflow) pressure and the suprahepatic cava vein (outflow) pressure (which was kept at a constant level of -1 mmHg) were measured continuously and the THPG was calculated by subtracting the outflow from the inflow pressure.

Dose-response experiments

A syringe pump with vasoactive substance was connected to the *ex vivo in situ* perfusion set-up. The pump was started after a stabilisation period of 20 min at a constant perfusion flow of 30 mL/min. The dose was increased by 0.5 log every 5 min and the THPG was continuously measured while increasing the dose. The effect of PPAR agonists on hepatic vascular reactivity was tested with following vasoactive compounds: 10^{-12} to 3×10^{-9} mol/L endothelin-1 (ET-1), 10^{-6} to 3×10^{-4} mol/L methoxamine (Mx, alpha-1-adrenoceptor agonist), and 10^{-6}

to 3 × 10^{-4} mol/L acetylcholine (ACh, endothelial-dependent vasodilator) after preconstriction with 3 × 10^{-5} mol/L Mx for the MCDD model and 1.5 × 10^{-4} mol/L for the HFHFD model. Results are expressed in relative changes (*i.e.*, the increase or decrease in THPG compared to the baseline value before the compound was added).

Vascular corrosion casting
To explore the architecture of the hepatic vasculature, rats (n = 3/group) from the early MASLD model were sacrificed and vascular corrosion casts were prepared as previously described [3, 7]. The casts were examined systematically by scanning electron microscopy (Jeol JSM-IT100, Jeol, Tokyo, Japan). Images were made at x100 and x300 magnification and at 20.0 kV, and were subsequently described visually.

Drugs and solutions
Animals were anesthetized with a combination of ketamine (KETALAR®, 100 mg/kg body weight, Pfizer, Berlin, Germany) and xylazine (Rompun® 2%, 10 mg/kg body weight, Bayer, Leverkusen, Germany) intraperitoneally.
The isotonic Krebs–Ringer solution had the following composition: KCl 4.75 mM, MgSO4·7H2O 1.2 mM, KH2PO4 1.2 mM, CaCl2·2H2O 2.5 mM, CaEDTA 0.03 mM, NaCl 118.5 mM, NaHCO3 25 mM, glucose 11.1 mM; pH = 7.4.
ACh and ET-1 were purchased from Sigma-Aldrich Chemie Gmbh (Steinheim, Germany). Mx was purchased from Santa Cruz Biotechnology, Inc. (Dallas, Texas). All drugs were first dissolved in aqua destillata before diluting them in the Krebs–Ringer solution to obtain the given concentrations. All solutions were freshly prepared on the day the experiments were performed.
Placebo (1% methylcellulose + 0.05% poloxamer) and PPAR agonists (solubilized with 1% methylcellulose + 0.05% poloxamer) for oral treatment were provided by Inventiva Pharma (Daix, France).

Statistical analyses
Clinical approaches:
Continuous variables were described using median (interquartile range, IQR) and compared using the Wilcoxon-Mann-Whitney test for two groups comparisons, or the Kruskall-Wallis test followed by Dunn test for more than two groups comparisons. The 95% two-sided confidence interval was calculated when appropriate. Categorical variables were described as percentages of each category. The 95% two-sided confidence interval was calculated when appropriate using the exact (Clopper-Pearson) method. Categorical variables were compared between two groups using the Chi² test or the Fisher's exact test, and between more than two groups using the Cochran–Armitage asymptotic test for trend followed by Chi² or Fisher tests. Threshold for Fib-4 classes were based on EASL recommendations for noninvasive tests for the evaluation of liver disease severity and prognosis [8]. Treatment effect on continuous variables was evaluated using a Mixed Model for Repeated Measures with the variable change from baseline as endpoint and the treatment group and variable baseline value as fixed effect. The link between two continuous variables was assessed using the Spearman correlation. Missing data were not replaced. No multiplicity adjustments were defined as all endpoints are to be considered exploratory. The confidence intervals have not been adjusted for multiple comparisons and should not be used to infer definitive treatment effects. SAS® software version 9.2 was used.

Preclinical approaches:
Variables were presented as mean ± standard error of the mean (SEM) or median (interquartile range (IQR)) when appropriate and compared with two-way ANOVA (with the diet as the first factor (between), the treatment used as the second factor (within)) for normally distributed data followed by Tukey test and Kruskall-Wallis test for skewed data followed Dunn test, using Prism 9 (Version 9.2.0; Graphpad, California, USA). The THPG data were analysed

using a generalised estimating equation model followed by least significant difference *post hoc* testing when appropriate, using SPPS v29.0 (IBM, Armonk, NY, USA).
A p value < 0.05 was considered to represent a statistical difference.

Supplementary figures

Fig. S1. CD34 histology and quantification

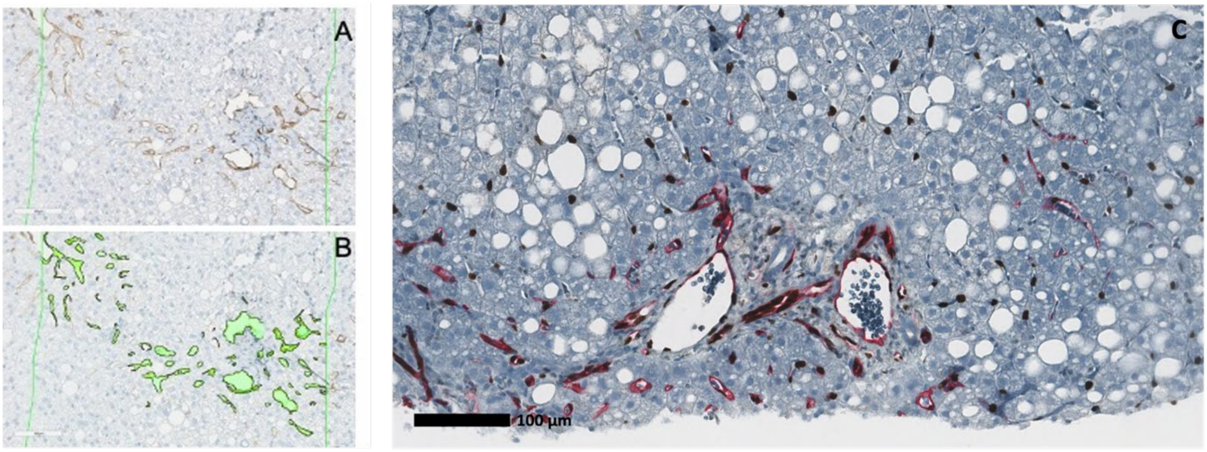

(A) CD34 immunostaining showing vessels delineating in brown; **(B)** segmentation of CD34 immunostaining using the microvessel algorithm (vessels are highlighted in green); **(C)** CD34 (pink) cytoplasmic staining co-localises with ERG nuclear staining (brown) in endothelial cells (representative of n=29).

Fig. S2A. Experimental design of an early MASLD rat model in a preventive treatment set-up.
Male Wistar Han rats of 8 weeks old were either fed a chow diet (CD) or a methionine-choline-deficient diet (MCDD) for 4 weeks and simultaneously treated with either placebo, fenofibrate (30 mg/kg), GW501516 (10 mg/kg), rosiglitazone (5 mg/kg) or lanifibranor (100 mg/kg) daily QD via oral gavage. After 4 weeks of diet and treatment, the following parameters were assessed: in vivo haemodynamics and blood pressures followed by ex vivo intrahepatic vascular resistance (IHVR) measurement in the same animals, dose-response experiments with endothelin-1 (ET-1), methoxamine (Mx) and acetylcholine (ACh), and histology.

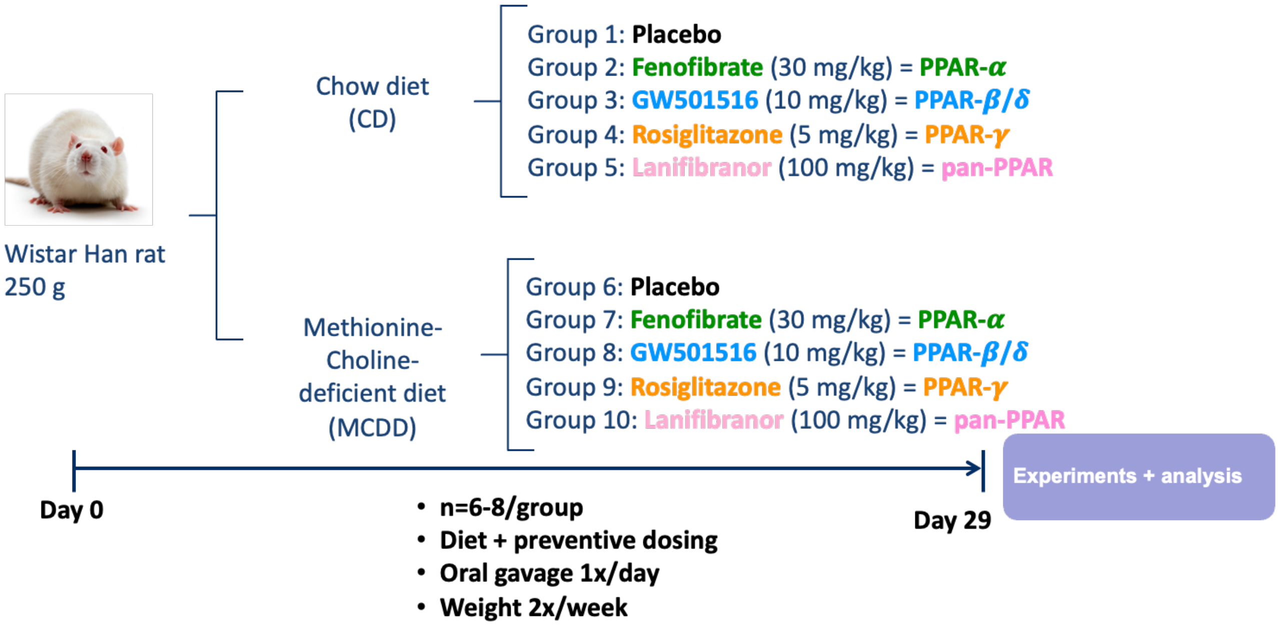

Fig. S2B. Experimental design of a rat model of MASH in a preventive treatment set-up.
8 weeks old male Zucker fatty rats fed a high-fat high-fructose diet (HFHFD) and 8 weeks old male Zucker lean rats fed a chow diet (CD) were concomitantly treated with either placebo or lanifibranor (100 mg/kg) daily QD via oral gavage during the whole period of 8 weeks of diet. After 8 weeks of diet and treatment, the following parameters were assessed: in vivo haemodynamics and blood pressures followed by ex vivo intrahepatic vascular resistance (IHVR) measurement in the same animals, dose-response experiments with methoxamine (Mx) and acetylcholine (ACh), and histology.

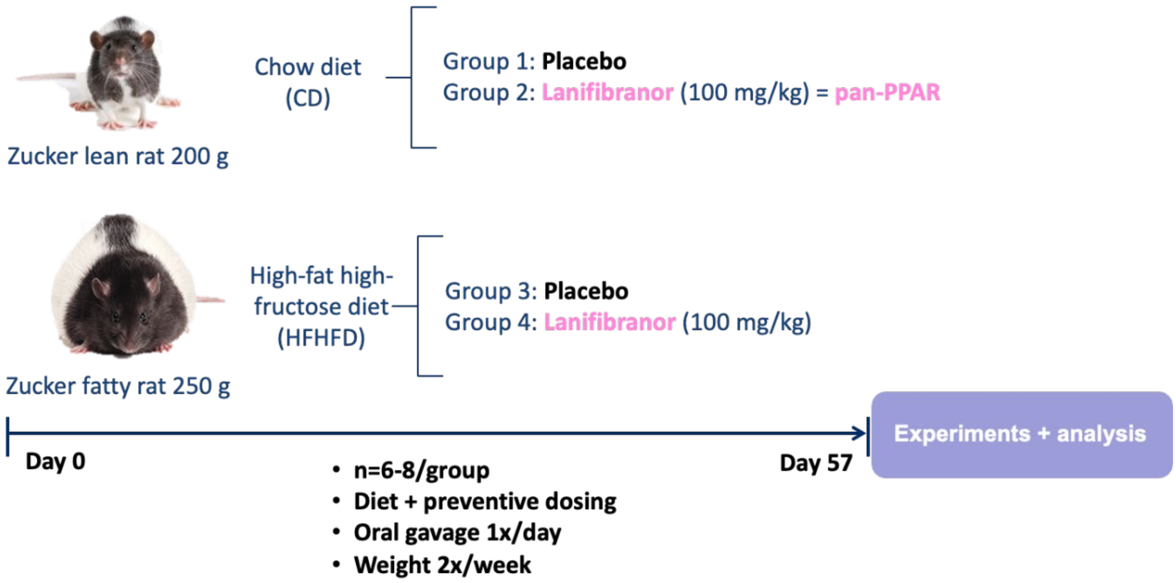

Fig. S3. Flow chart. Patients' disposition and definition of the analysed population

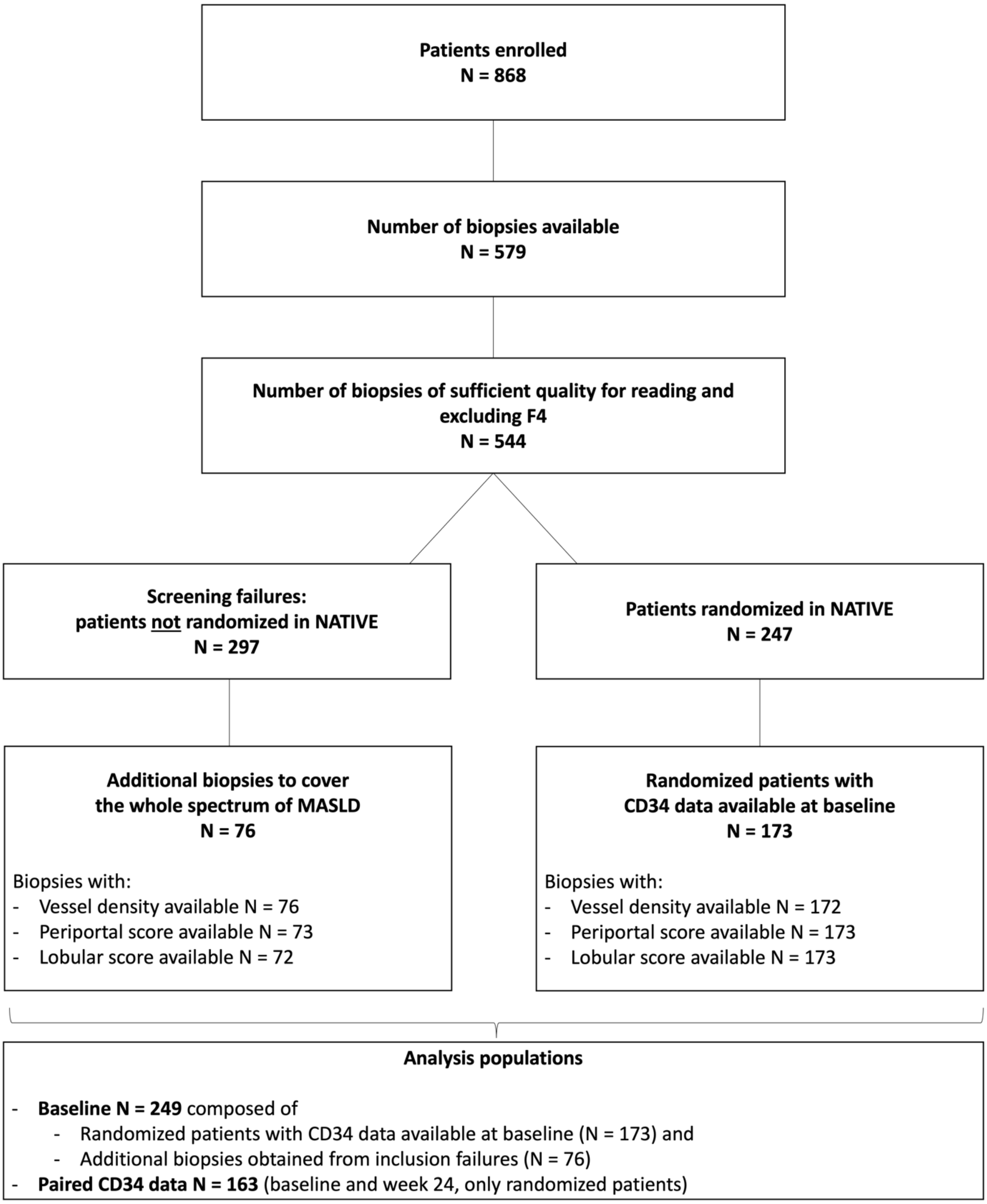

Fig. S4. Association between density of CD34 positive vessels and periportal and lobular scores.

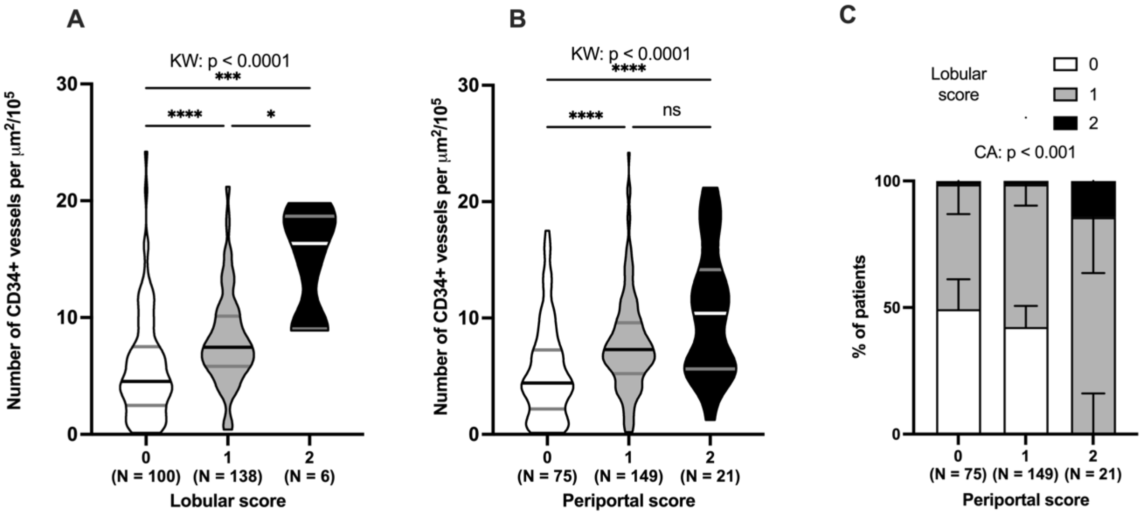

Density of CD34 positive vessels, periportal score and lobular score were available in 248, 246 and 245 patients, respectively, as detailed in Fig S3. Correlation between density of CD34 positive vessels and lobular score **(A)**, between density of CD34 positive vessels and periportal score **(B)** and between lobular and periportal scores **(C)** at baseline.

KW. Kruskal-Wallis; CA. Cochran-Armitage. When appropriate after Kruskal-Wallis, post-hoc Dunn's tests were performed between all columns with *: p<0.05; **: p<0.01; ***: p<0.001; ****: p<0.0001. For violin plots, the bars represent the median ± IQR, otherwise bars represent 95% confidence intervals.

Fig. S5. Relationship between CD34 staining level and localisation and histological features in MASLD

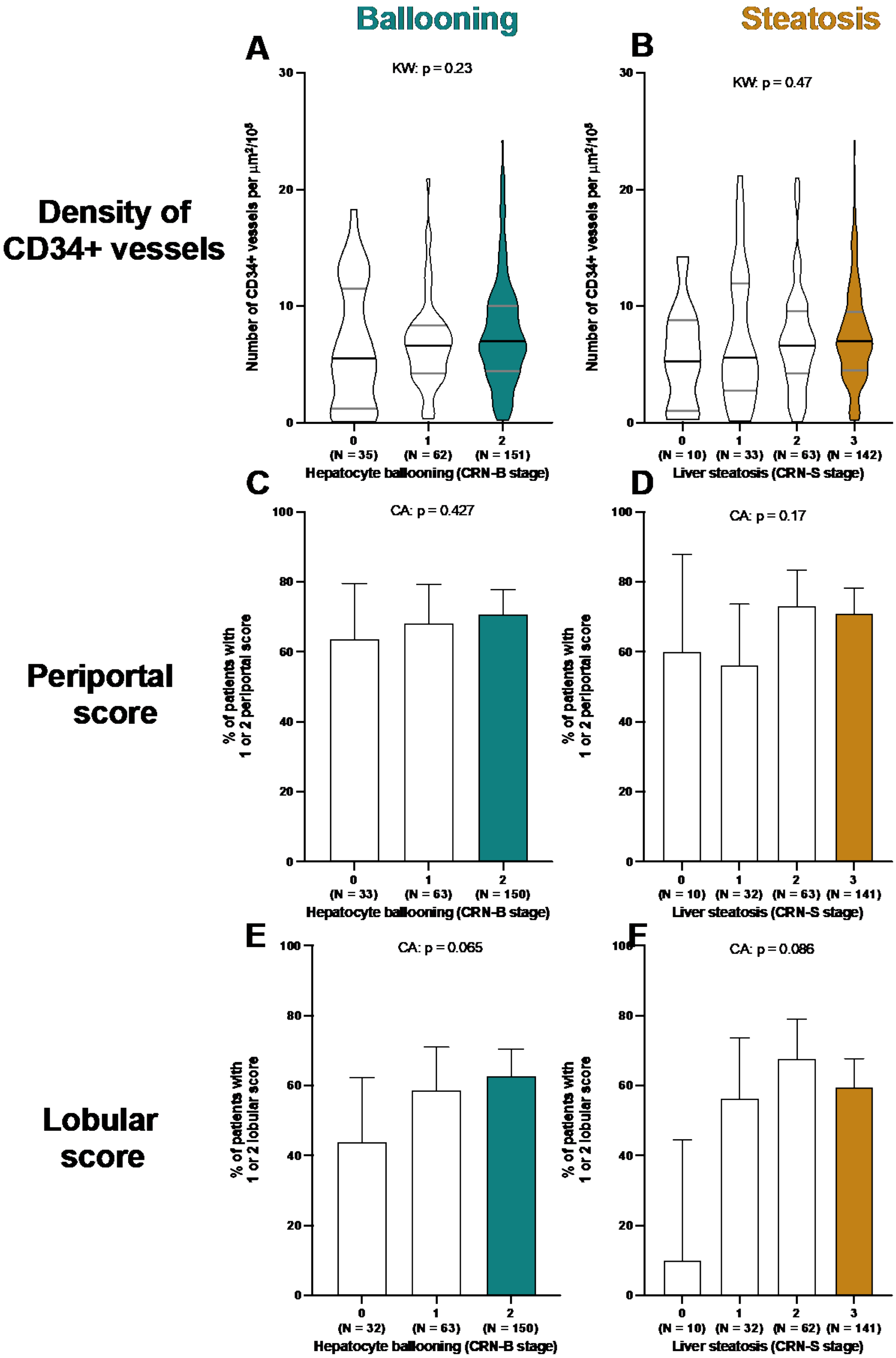

Density of CD34 positive vessels, periportal score and lobular score were available in 248, 246 and 245 patients, respectively, as detailed in Fig. S3. Baseline density of CD34 positive vessels is displayed according to ballooning (CRN-B grade) **(A)** and according to steatosis (CRN-S grade) **(B)**. Percentage of patients with periportal score for CD34 staining of 1 or 2 is displayed according to ballooning (CRN-B grade) **(C)** and according to steatosis (CRN-S grade) **(D)**. Percentage of patients with lobular score for CD34 staining of 1 or 2 is displayed according to ballooning (CRN-B grade) **(E)** and according to steatosis (CRN-S grade) **(F)**.
CRN, Clinical Research Network; KW, Kruskal-Wallis; CA, Cochran-Armitage. For violin plots, the bars represent the median ± IQR, otherwise bars represent 95% confidence intervals.

Fig. S6. Liver over total body weight ratio in MCDD model.

Male Wistar Han rats of 8 weeks old were either fed a chow diet (CD) or a methionine-choline-deficient diet (MCDD) for 4 weeks and simultaneously treated with either placebo, fenofibrate (30 mg/kg), GW501516 (10 mg/kg), rosiglitazone (5 mg/kg) or lanifibranor (100 mg/kg) daily QD via oral gavage. After 4 weeks of diet and treatment in the MCDD-fed groups the liver/total body weight ratio was significantly increased compared to their healthy counterparts, except for fenofibrate, because the liver weight of CD rats treated with fenofibrate is increased (a well-known PPAR-α effect). This further implies that the weight increase in the MCDD group treated with fenofibrate is mainly a compound effect rather than steatosis. n = 5-8/group per experiment. Pooled data from all experiments (n = 37-60/group) were analysed using Kruskal-Wallis followed by Dunn test and presented as median (IQR) with ns (not significant), **: p<0.01; ***: p<0.001; ****: p<0.0001.

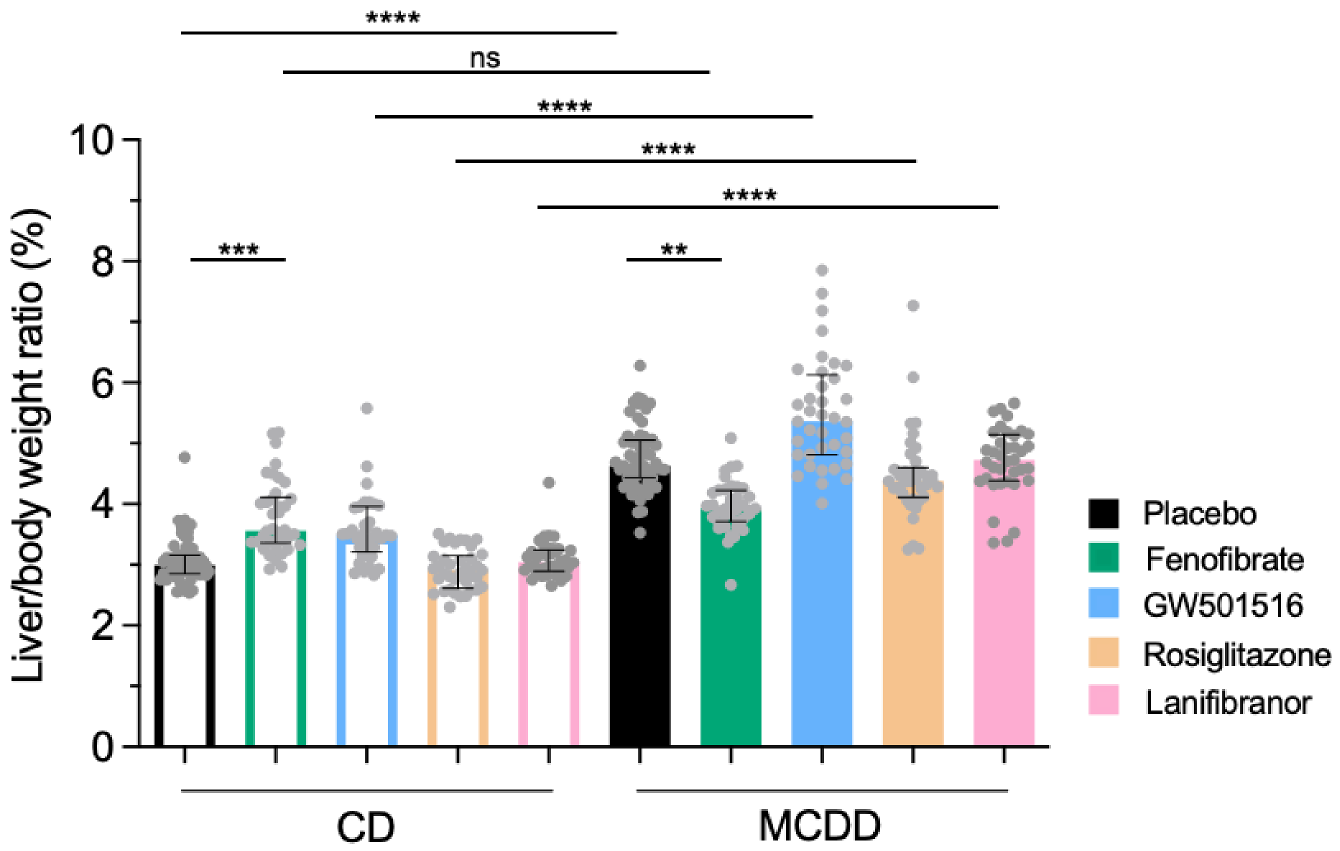

Fig. S7. Histological assessment of fibrosis in histological liver sections of early MASLD.

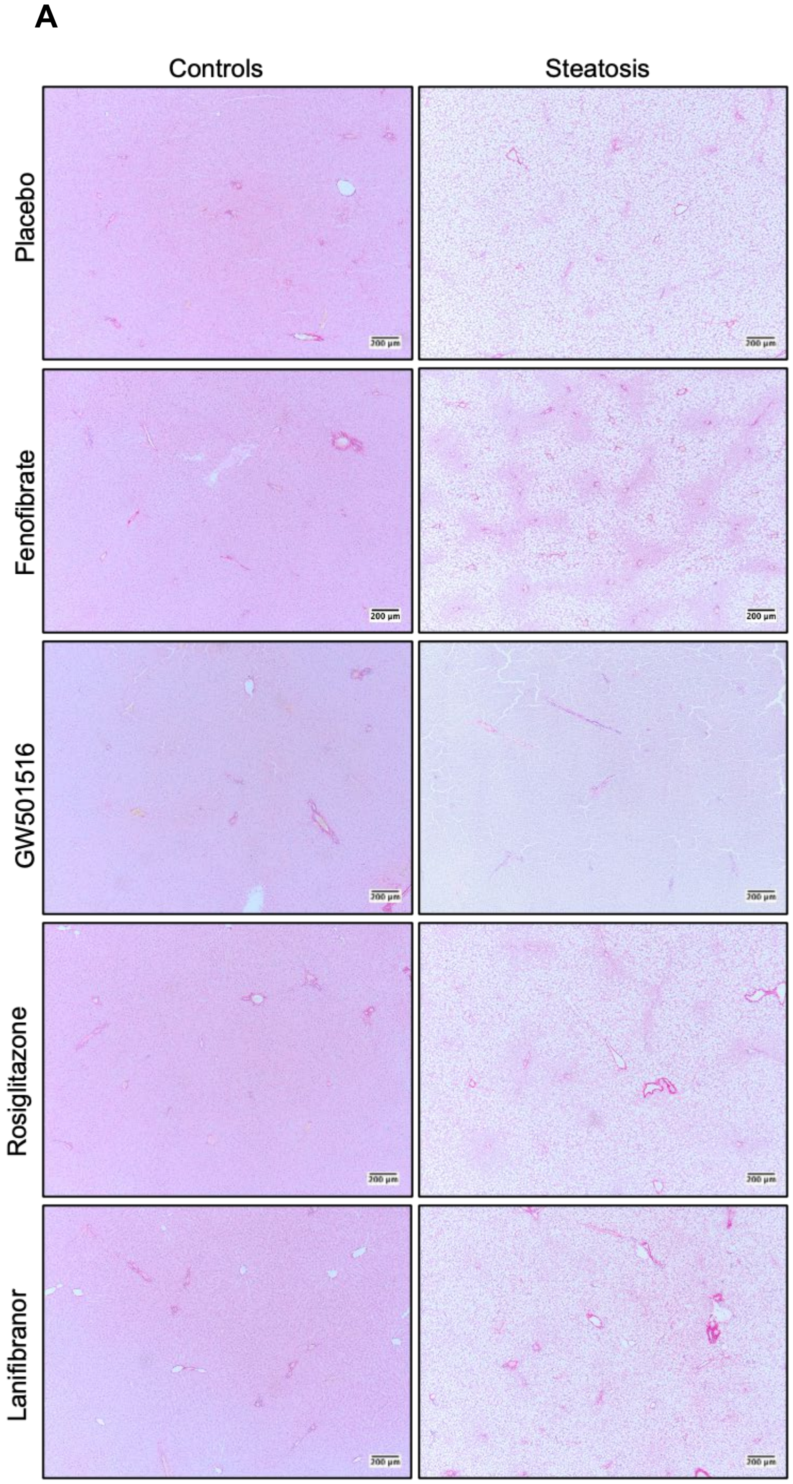

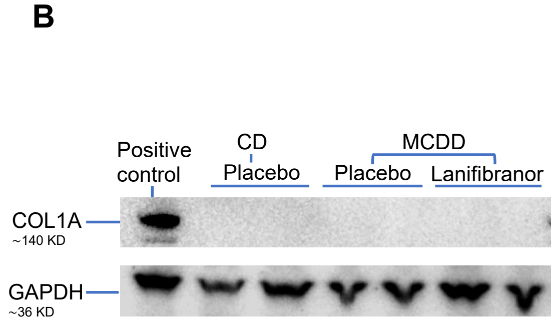

A) Sirius red staining, original magnification 4x (Olympus BX43, microscope lens 4X/0.1 NA Plan Apo; 1.099 μm/pixel). 8 weeks old male Wistar Han rats either fed a chow diet (CD) or a methionine-choline-deficient diet (MCDD) for 4 weeks were preventively treated with either placebo, fenofibrate (30 mg/kg), GW501516 (10 mg/kg), rosiglitazone (5 mg/kg) or lanifibranor (100 mg/kg) daily, QD via oral gavage. There were no signs of reactive fibrosis in liver tissues from all the groups.

B) Western blot analysis of collagen type 1 (COL1A), and GAPDH as loading control, in liver tissues of rats fed a chow diet (n = 2), or MCDD with placebo (n = 2) or lanifibranor (n = 2). Rat tail collagen was used as a positive control and displayed a strong signal for collagen, while in the experimental groups a clear signal for collagen was absent confirming the histological findings.

Fig. S8. Assessment of steatosis in early MASLD.

A) Images of haematoxylin and eosin (H-E) stained liver tissue sections (Olympus BX43, microscope lens 10X/0.45 NA Plan Apo; resolution 1 pixel = 0.442 μm). **B)** Steatosis quantification defined as macrovesicular fat droplets fraction per area (%). Male Wistar Han rats (n = 6/group) were either fed a chow diet (CD) or a methionine-choline-deficient diet (MCDD) for 4 weeks and preventively treated with either placebo, fenofibrate (30 mg/kg), GW501516 (10 mg/kg), rosiglitazone (5 mg/kg) daily QD via oral gavage. Data were analysed using two-way ANOVA followed by post hoc Tukey and presented as mean ± standard error of the mean with ns: not significant; **: p<0.01.

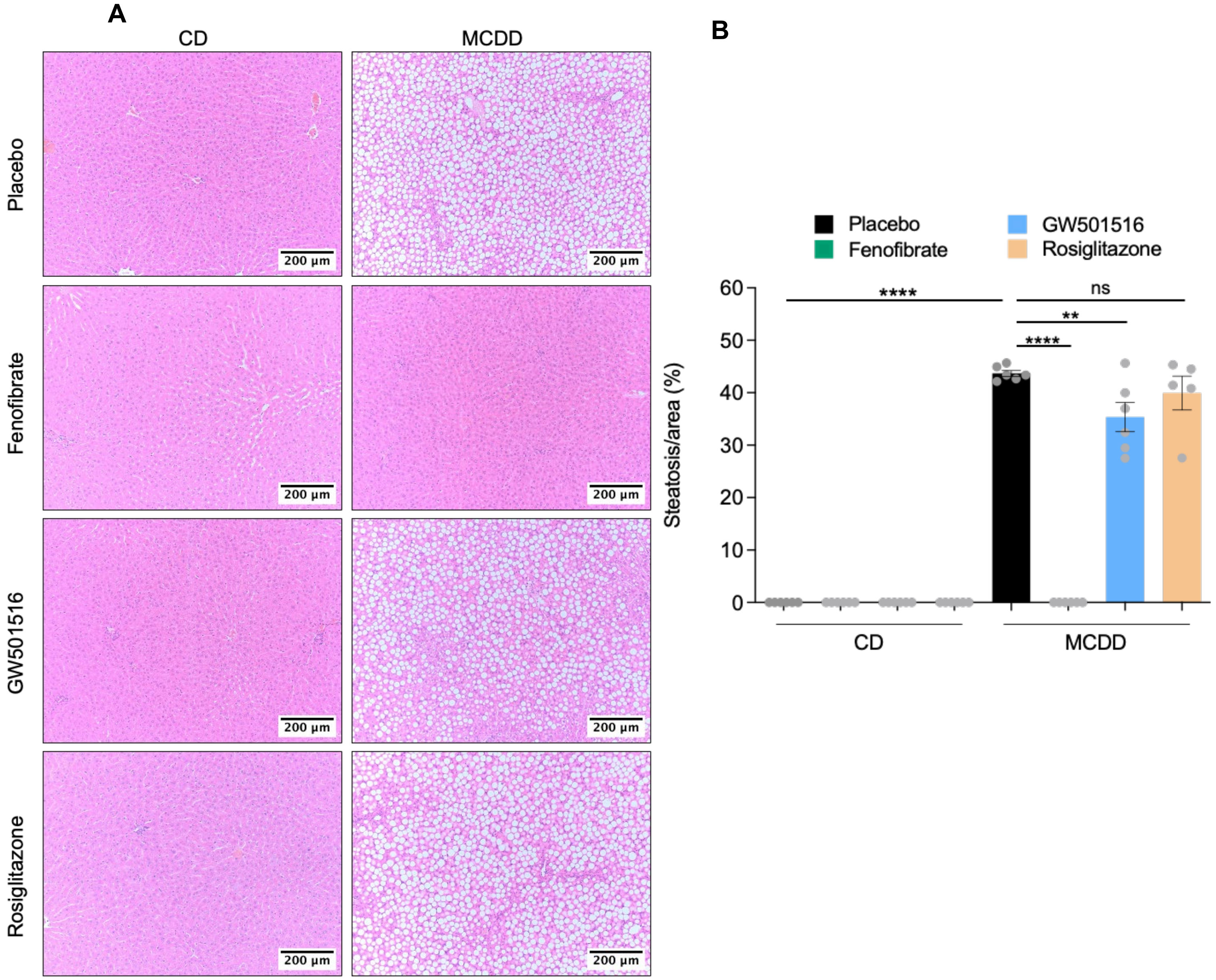

Fig. S9. Histological assessment of liver sections of rats with MASH

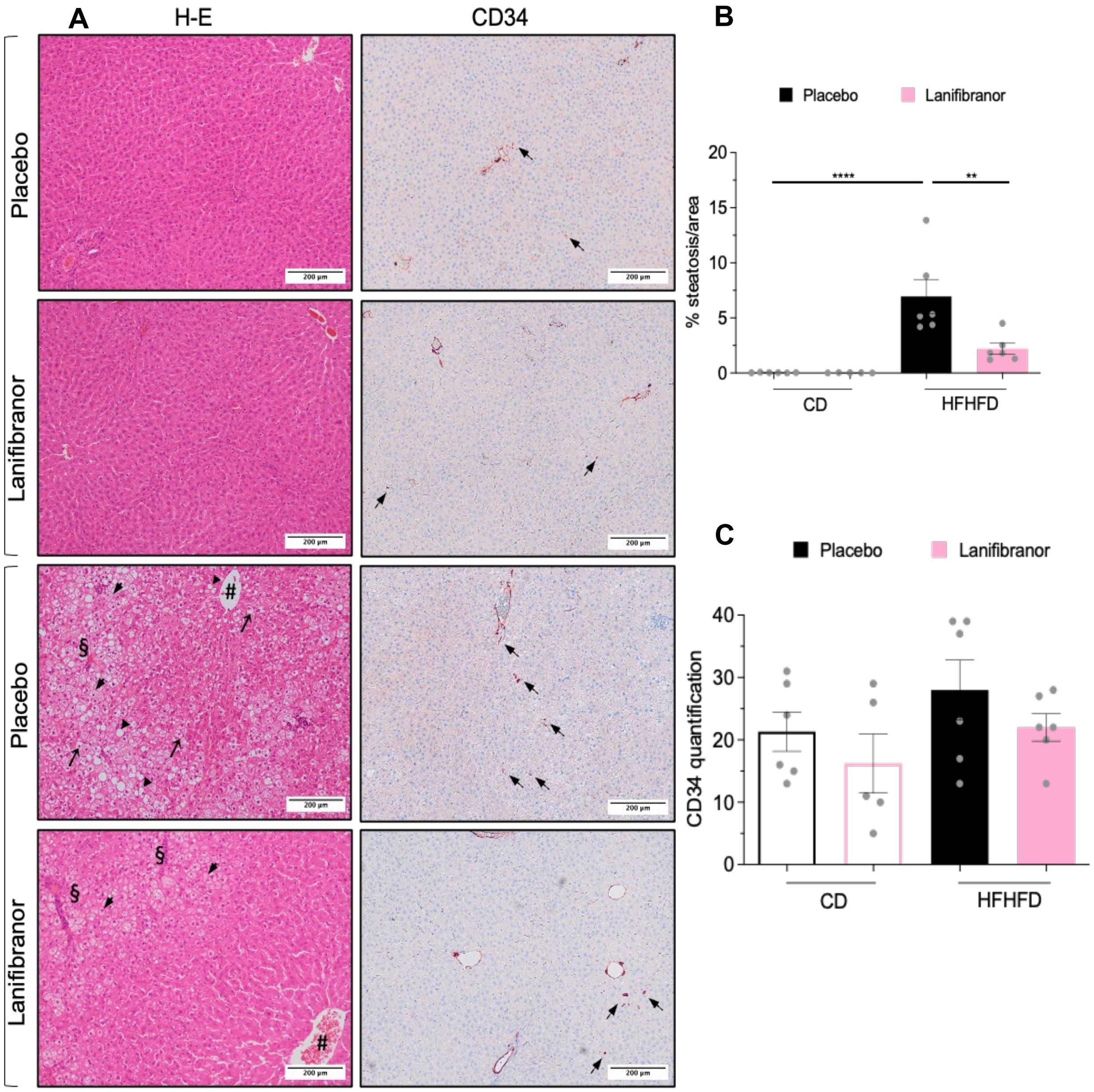

8 weeks old male Zucker fatty rats (n = 6/group) fed a high-fat high-fructose diet (HFHFD) and 8 weeks old male Zucker lean rats (n = 5-6/group) fed a chow diet (CD) were concomitantly treated with either placebo or lanifibranor (100 mg/kg) daily QD via oral gavage during the complete period of 8 weeks of diet. (Olympus BX43, microscope lens 10X/0.45 NA Plan Apo; resolution 1 pixel = 0.442 μm). **A)** Haematoxylin and eosin (H-E), and CD34 stained histological liver sections. In H-E images periportal area is marked with "§". Centrilobular area is marked with "#". Short arrows point microvesicular steatosis, arrow heads show macrovesicular steatosis and thin arrows show ballooning. In CD34 images arrows point CD34 positive spots. **B)** Steatosis quantification defined as fraction of macrovesicular fat droplets per area (%). **C)** Blinded CD34 semi-quantification. Data is presented as mean ± SEM. Two-way ANOVA was performed followed by post hoc Tukey.

Fig. S10. Assessment of fibrosis on Sirius red stained livers of Zucker rats.

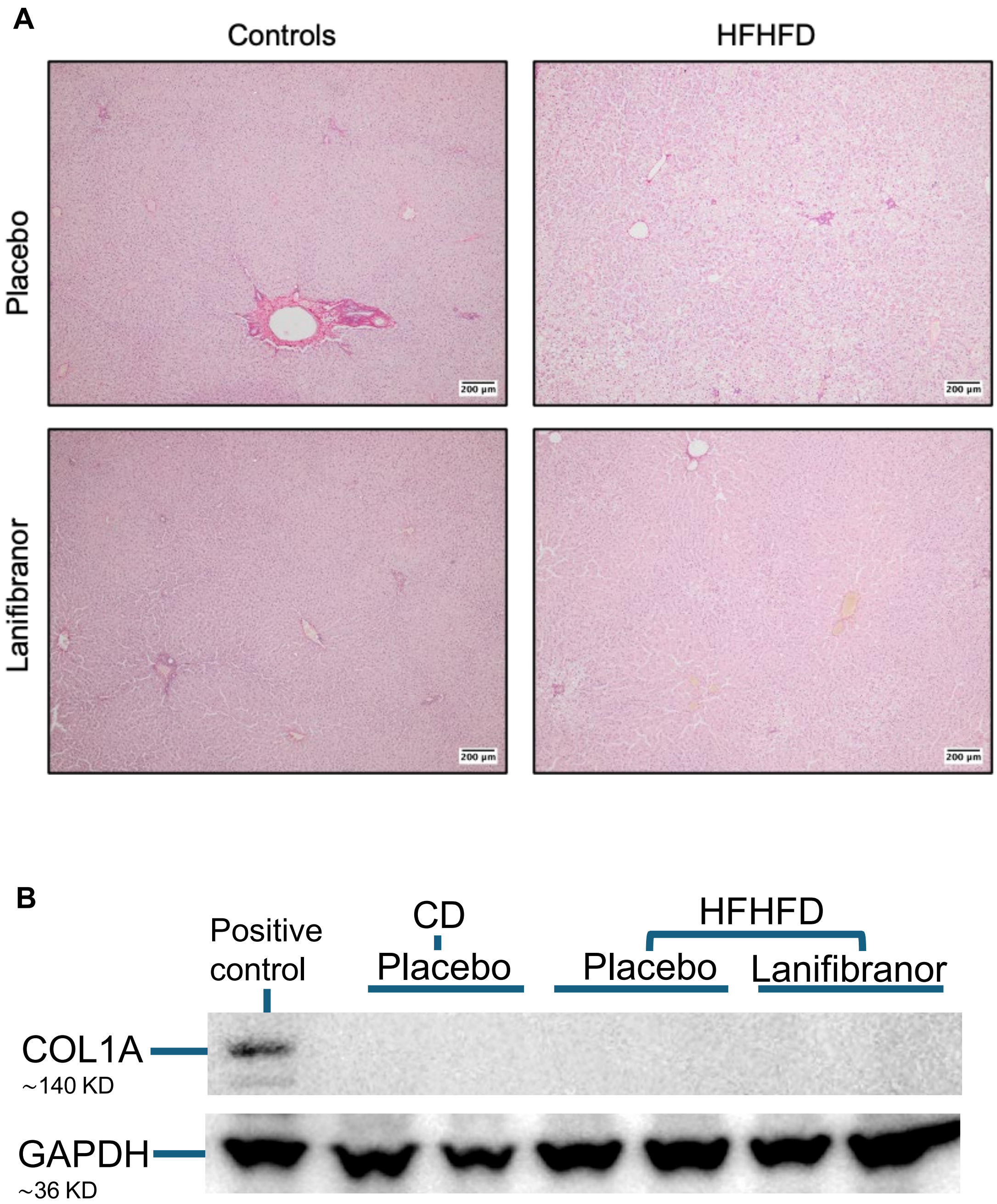

A) 8 weeks old male Zucker fatty rats (n = 6/group) fed a high-fat high-fructose diet (HFHFD) and male Zucker lean rats (n = 6/group) fed a chow diet (CD) were concomitantly treated with either placebo or lanifibranor (100 mg/kg) daily QD via oral gavage during the complete period of 8 weeks of diet. Control livers did not show any abnormalities. In placebo treated HFHFD-fed Zucker rats there was no sign of fibrosis. Original magnification 4x (Olympus BX43, microscope lens 4X/0.1 NA Plan Apo; 1.099 µm/pixel). **B)** Western blot analysis of collagen type 1 (COL1A), and GAPDH as loading control, in liver tissues of rats male Zucker lean rats fed a chow diet (CD) (n = 2), in placebo-treated HFHFD-fed (MASH) rats (n = 2) and lanifibranor-treated HFHFD-fed (MASH) rats (n = 2). Rat tail collagen was used as a positive control and displayed a strong signal for collagen, while in the experimental groups there was no signal for collagen. This is in line with the histological findings.

Fig. S11. Body weight, liver weight and spleen weight evolution in a MASH model following 8 weeks of chow diet or high-fat high-fructose diet.

A) Weight gain, **B)** Liver weight, **C)** Liver over total body weight ratio (%), **D)** Spleen weight. 8 weeks old male Zucker fatty rats fed a high-fat high-fructose diet (HFHFD) and 8 weeks old male Zucker lean rats fed a chow diet (CD) were preventively treated with either placebo or lanifibranor (100 mg/kg) daily QD via oral gavage during the complete period of 8 weeks of diet. n = 5-8/group per experiment. Pooled data (n = 28-30/group) were analysed using two-way ANOVA (for A & D) followed by post hoc Tukey and presented as mean ± standard error of the mean or Kruskal-Wallis (B & C) followed by Dunn test and presented as median (IQR). ns: not significant; *: p<0.05; **: p<0.01; ****: p<0.0001. Spleen weight was only determined in one experiment, therefore, no pooled data available.

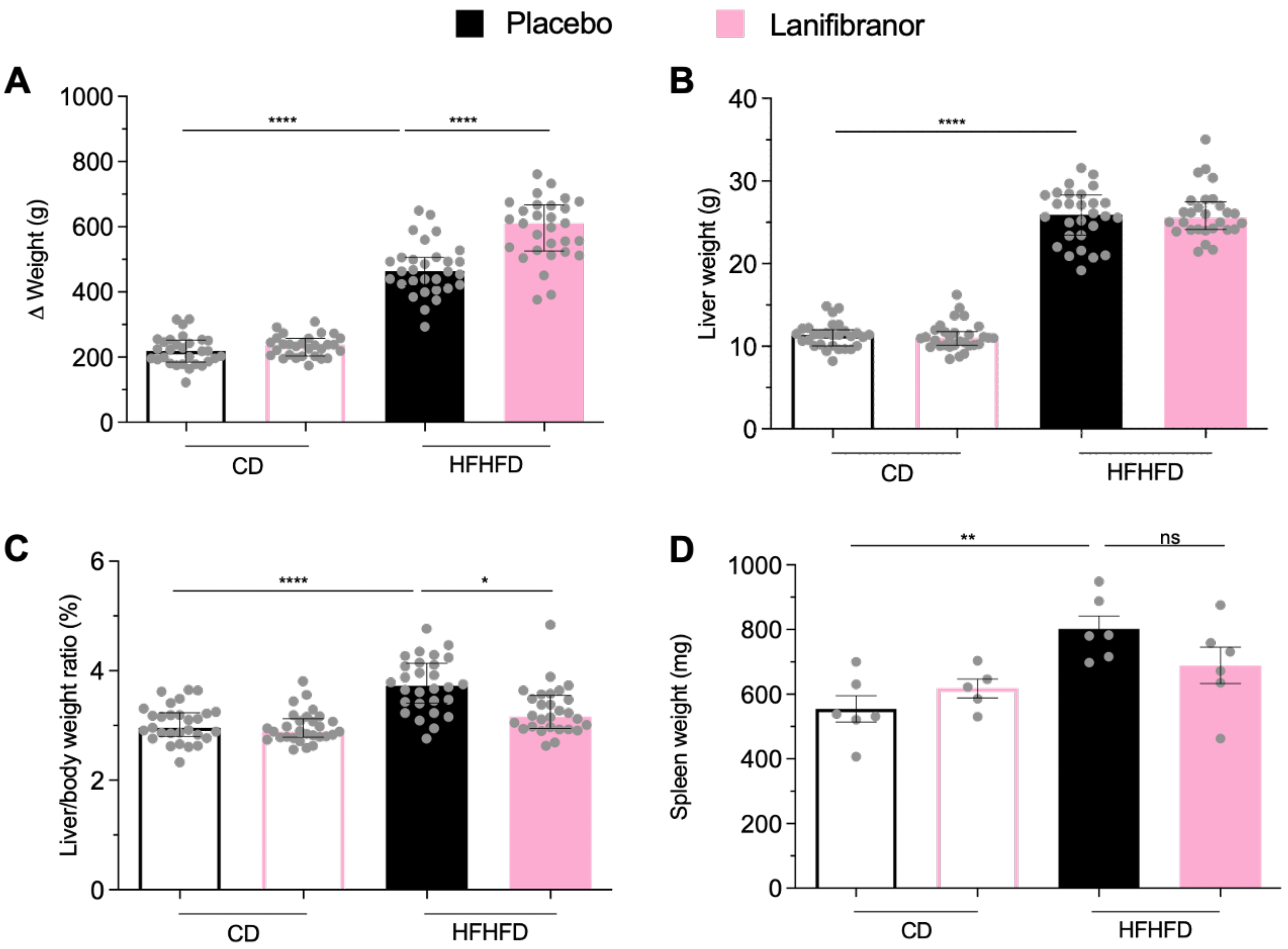

Fig. S12. Immunohistology of liver sections.

A) Images of CD34 stained liver tissue sections (Olympus BX43, microscope lens 10X/0.45 NA Plan Apo; resolution 1 pixel = 0.442 μm). **B)** Blinded CD34 semi-quantification. 8 weeks old male Wistar Han rats fed chow- (CD) or methionine - choline - deficient diet (MCDD) for 4 weeks were preventively treated with either placebo, fenofibrate (30 mg/kg), GW501516 (10 mg/kg), rosiglitazone (5 mg/kg) or lanifibranor (100 mg/kg). Data presented as mean ± SEM (n = 5-6/group). Two-way ANOVA was performed followed by post hoc Tukey with ns: not significant; **: p<0.01; ***: p<0.001; ****: p<0.0001. For clarity only the comparisons with MCDD-placebo are shown. Arrows point CD34 positive staining.

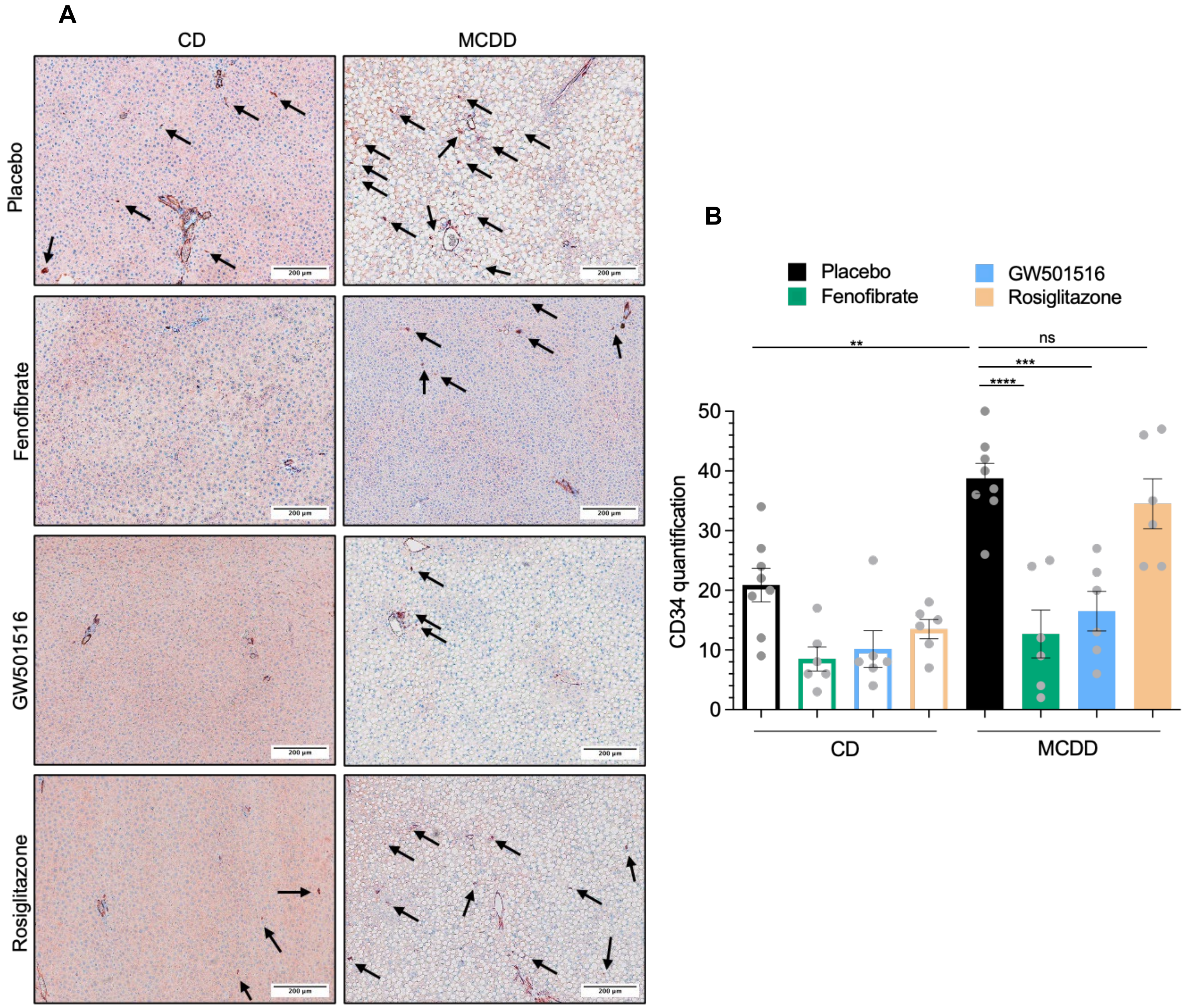

Fig. S13. In vivo portal venous pressure in early MASLD.

Male Wistar Han rats of 8 weeks old were either fed a chow diet (CD) or a methionine-choline-deficient diet (MCDD) for 4 weeks and simultaneously treated with either placebo, fenofibrate (30 mg/kg), GW501516 (10 mg/kg), rosiglitazone (5 mg/kg) or lanifibranor (100 mg/kg) daily QD via oral gavage. n = 7-8/ group per experiment. Pooled data (n = 24-60/group) were analysed using Kruskal-Wallis followed by Dunn test and presented as median (IQR) with ns: not significant; *: p<0.05; ****: p<0.0001. For clarity only the comparisons with MCDD-placebo are shown. PVP, portal venous pressure.

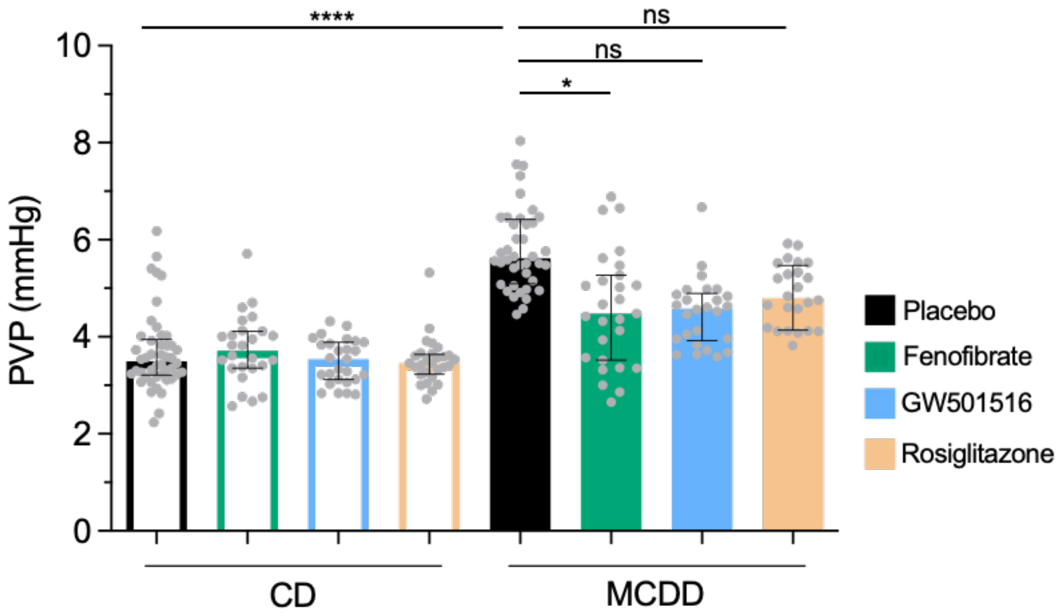

Fig. S14. *In vivo* arterial pressures and pulse rates in early MASLD.
Male Wistar Han rats of 8 weeks old were either fed a chow diet (CD) or a methionine-choline-deficient diet (MCDD) for 4 weeks and simultaneously treated with either placebo, fenofibrate (30 mg/kg), GW501516 (10 mg/kg), rosiglitazone (5 mg/kg) or lanifibranor (100 mg/kg) daily QD via oral gavage. n = 6-8/group per experiment. Pooled data (n = 25-43/group) were analysed using Kruskal-Wallis followed by Dunn test and presented as median (IQR) with **: p<0.01; ***: p<0.001; ****: p<0.0001. For clarity only the relevant comparisons with MCDD-placebo are shown. PVP, portal venous pressure; MABP, mean arterial blood pressure; SBP, systolic blood pressure; DBP, diastolic blood pressure; BPM, beats per minute.

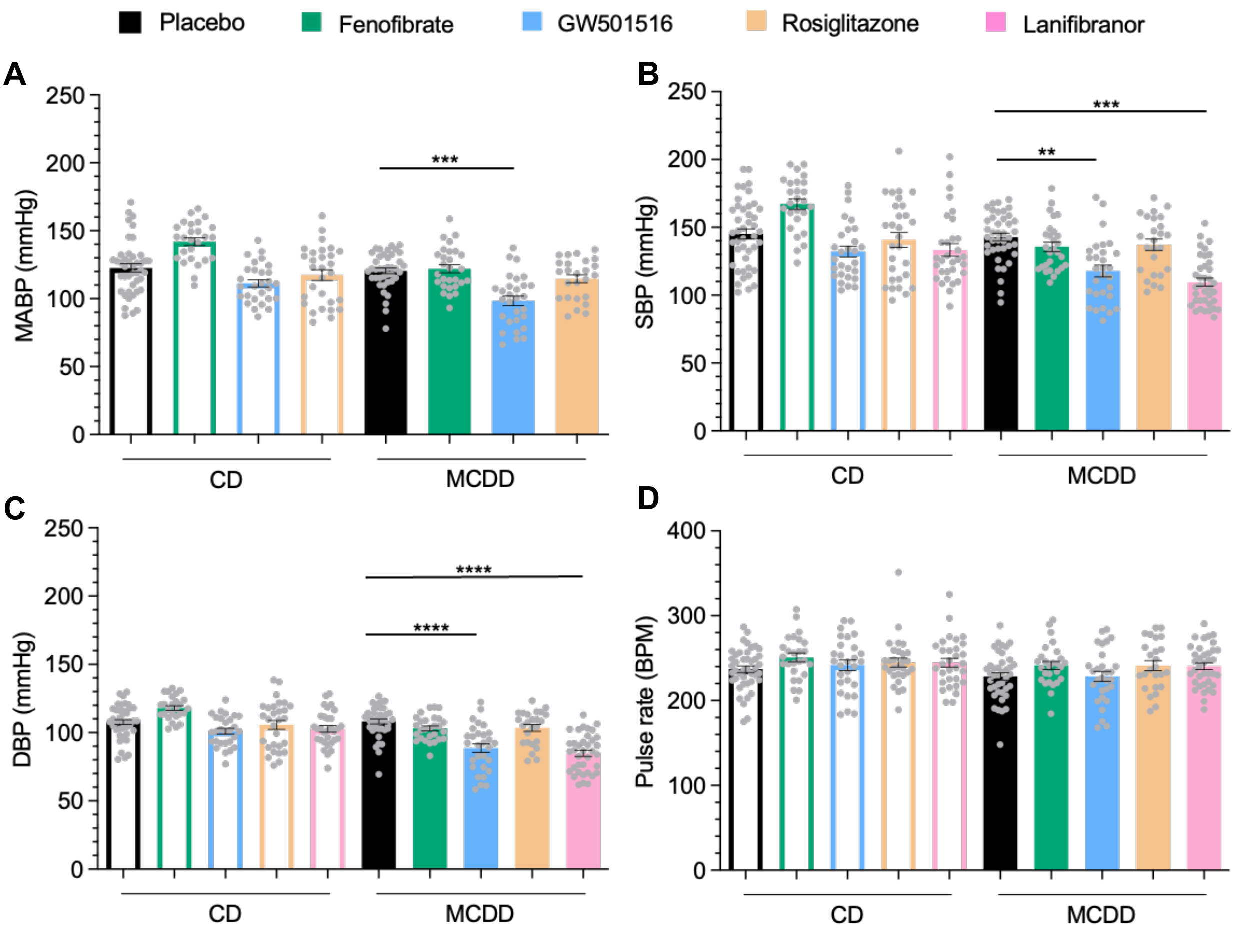

Fig. S15. *In vivo* arterial pressures and pulse rates in Zucker rats.
8 weeks old male Zucker fatty rats fed a high-fat high-fructose (HFHFD) diet and 8 weeks old male Zucker lean rats fed a chow diet (CD) were preventively treated with either placebo or lanifibranor (100 mg/kg) daily QD via oral gavage during the complete period of 8 weeks of diet. n = 6-8/group per experiment. Pooled data (n = 16-19/group) were analysed using two-way ANOVA followed by post hoc Tukey and presented as mean ± standard error of the mean. ****: p<0.0001. For clarity only the relevant comparisons with MCDD-placebo are shown. PVP, portal venous pressure; MABP, mean arterial blood pressure; SBP, systolic blood pressure; DBP, diastolic blood pressure; BPM, beats per minute.

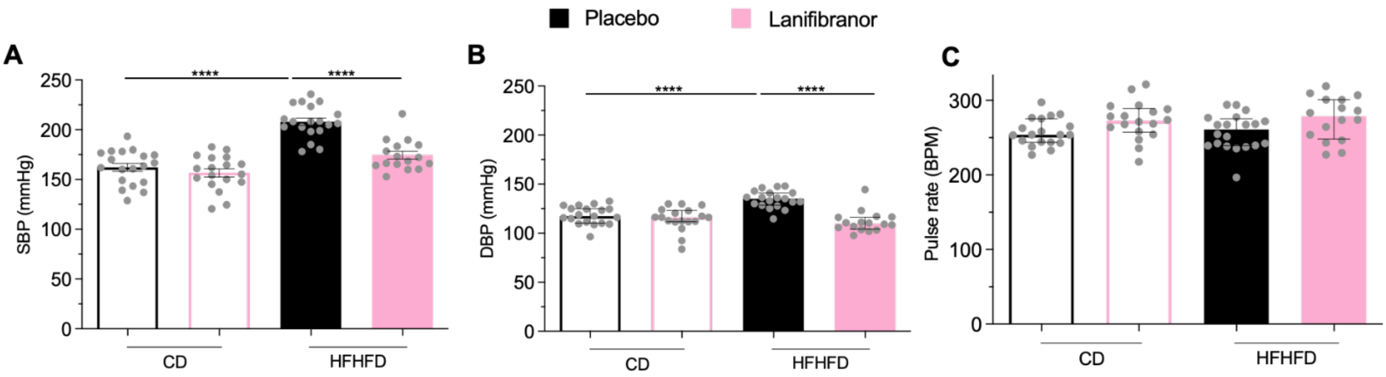

Fig. S16. ***In situ ex vivo*** **liver perfusion: transhepatic pressure gradient for assessment of intrahepatic vascular resistance in early MASLD.**
Male Wistar Han rats of 8 weeks old were either fed a chow diet (CD) or a methionine-choline-deficient diet (MCDD) for 4 weeks and simultaneously treated with either **A)** placebo, **B)** fenofibrate (30 mg/kg), **C)** GW501516 (10 mg/kg) or **D)** rosiglitazone (5 mg/kg) daily QD via oral gavage during the complete 4 weeks of diet. n = 7-8/group. Data presented as mean ± SEM. The THPG data were analysed using a generalised estimating equation model followed by least significant difference post hoc testing with *: p<0.05; ***: p<0.001; ****: p<0.0001. Statistics on graph shown for most important comparisons only (MCDD + treatment vs. MCDD + placebo). THPG, transhepatic pressure gradient; min, minute.

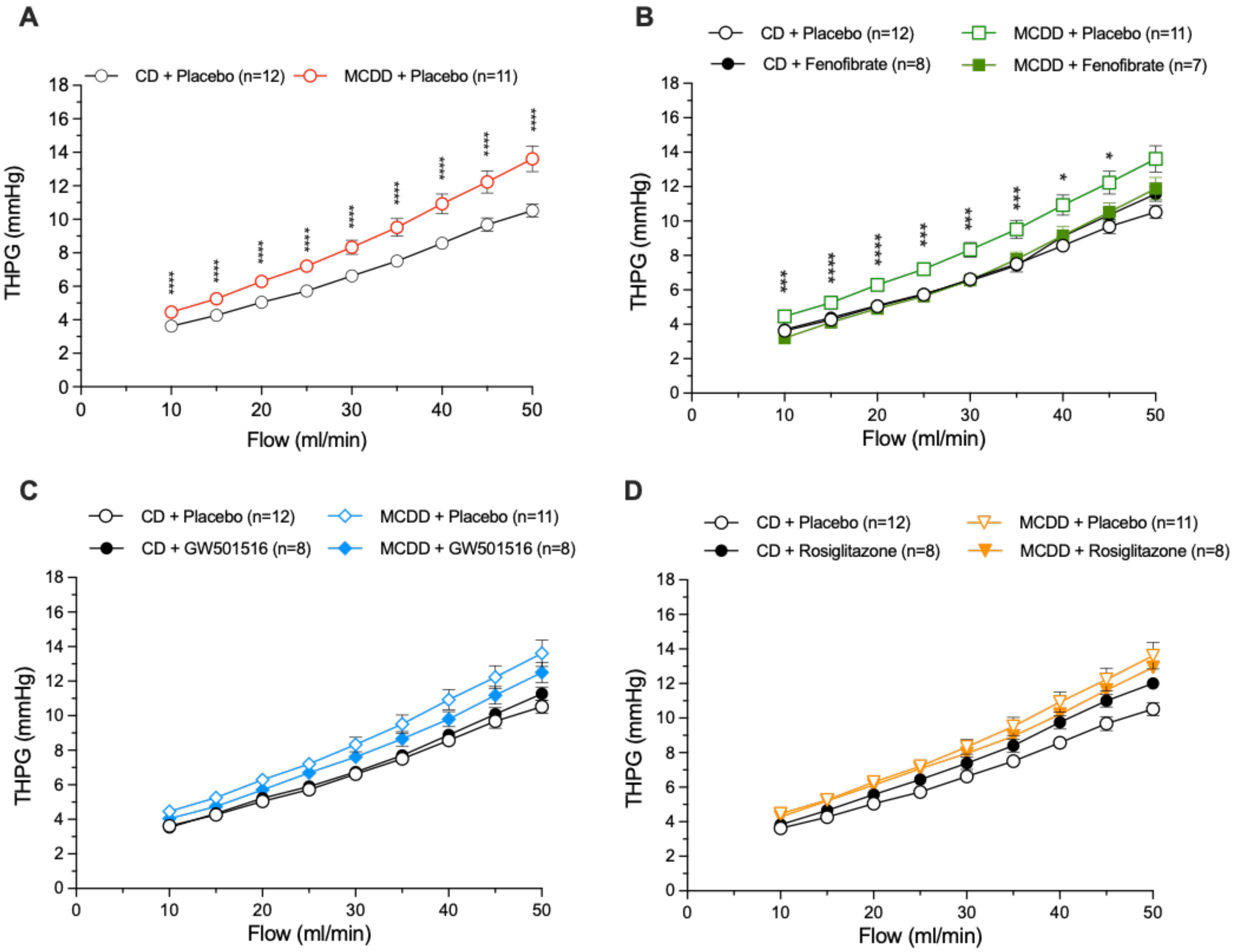

Fig. S17. *In situ ex vivo* liver perfusion: relative change of transhepatic pressure gradient after dose-response measurement with endothelin-1.

Male Wistar Han rats of 8 weeks old were either fed a chow diet (CD) or a methionine-choline-deficient diet (MCDD) for 4 weeks and simultaneously treated with either **A)** placebo, **B)** fenofibrate (30 mg/kg), **C)** GW501516 (10 mg/kg) or **D)** rosiglitazone (5 mg/kg) daily, QD via oral gavage. n = 7-8/group). Data presented as mean ± SEM. The THPG data were analysed using a generalised estimating equation model followed by least significant difference post hoc testing. Statistics on graph show with * for MCDD + treatment vs MCDD + placebo and with ° for CD + treatment vs. CD + placebo. *: p<0.05; **: p<0.01; ***: p<0.001; ****: p<0.0001. °: p<0.05; °°: p<0.01; °°°°: p<0.0001. THPG, transhepatic pressure gradient; ET-1, endothelin-1; (Log M), logarithmic concentration in mol/Liter.

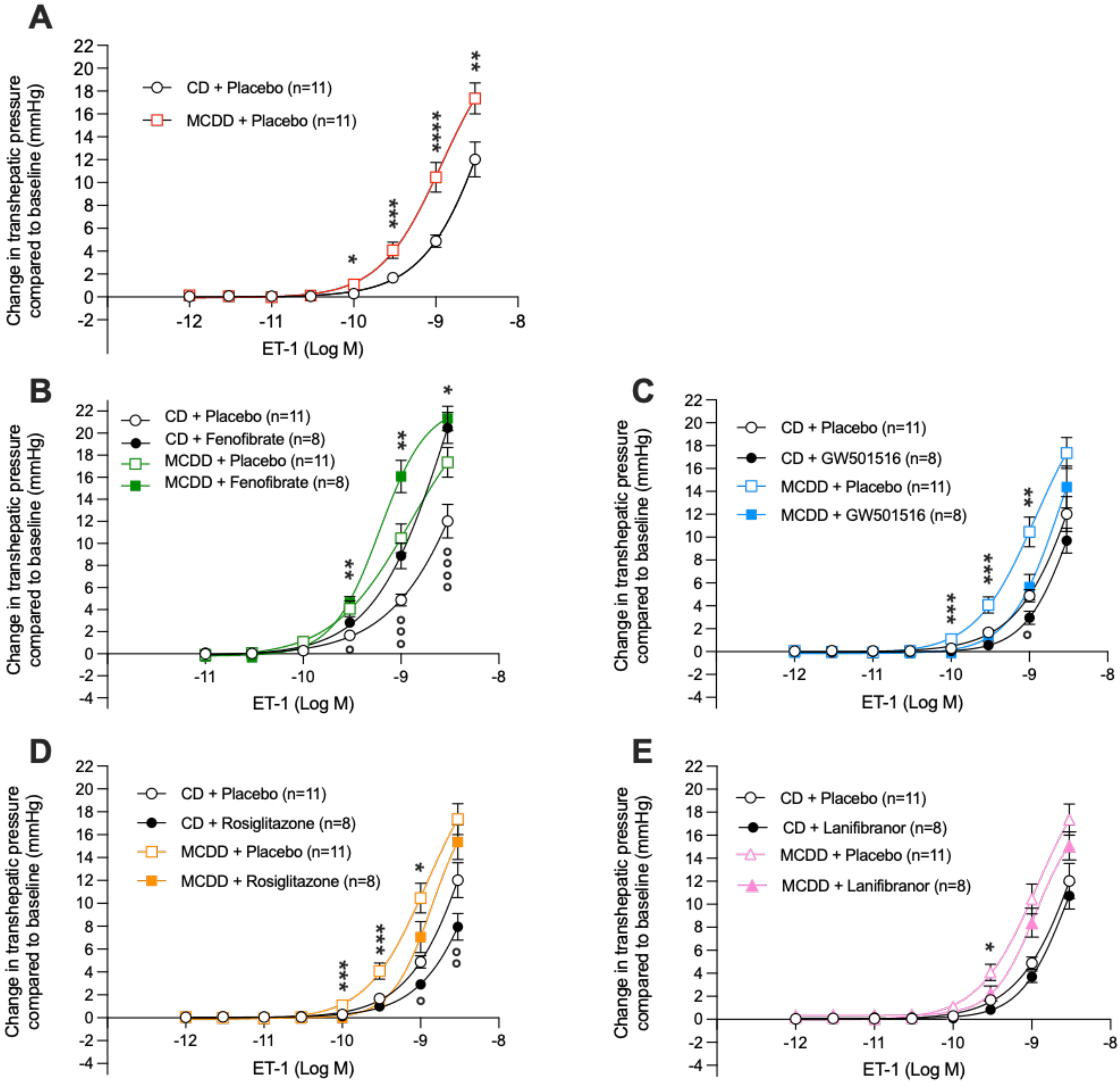

Fig. S18. *In situ ex vivo* liver perfusion in early MASLD: relative change of transhepatic pressure gradient after dose-response measurement with methoxamine.

Male Wistar Han rats of 8 weeks old were either fed a chow diet (CD) or a methionine-choline-deficient diet (MCDD) for 4 weeks and were simultaneously treated with either **A)** placebo, **B)** fenofibrate (30 mg/kg), **C)** GW501516 (10 mg/kg) or **D)** rosiglitazone (5 mg/kg) daily QD via oral gavage. n = 7-11/group). Data is presented as mean ± SEM. The THPG data were analysed using a generalised estimating equation model followed by least significant difference post hoc testing with *: p<0.05; **: p<0.01; ***: p<0.001; ****: p<0.0001. Statistics on graph shown for most important comparisons only (MCDD + treatment vs. MCDD + placebo). THPG, transhepatic pressure gradient; Mx, methoxamine; (Log M), logarithmic concentration in mol/Liter.

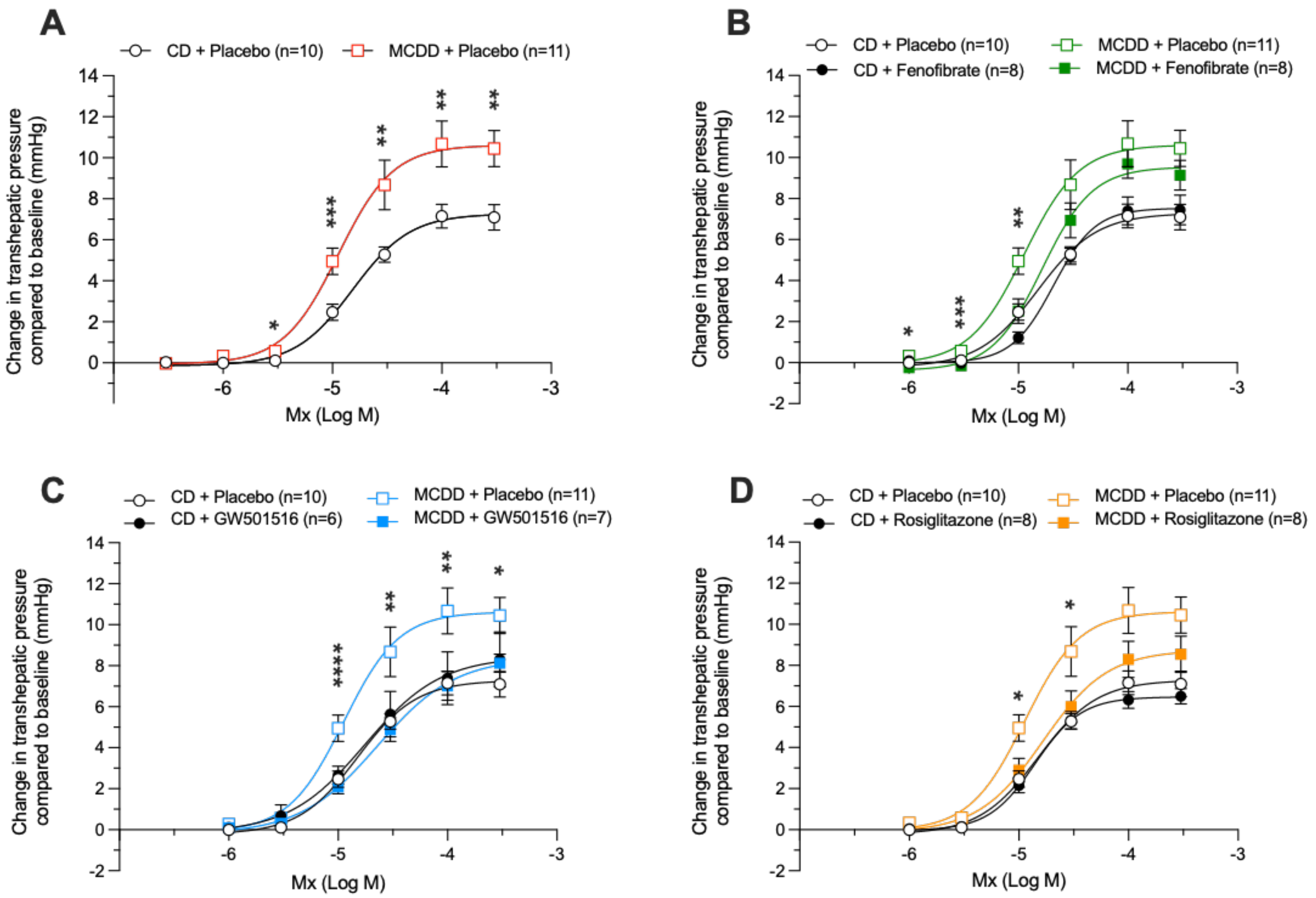

Fig. S19. *In situ ex vivo* liver perfusion in early MASLD: % vascular relaxation with increasing doses of acetylcholine, after methoxamine preconstriction.
Male Wistar Han rats of 8 weeks old were either fed a chow diet (CD) or a methionine-choline-deficient diet (MCDD) for 4 weeks and were simultaneously treated with either **A)** placebo, **B)** fenofibrate (30 mg/kg), **C)** GW501516 (10 mg/kg) or **D)** rosiglitazone (5 mg/kg) daily QD via oral gavage. n = 7-13/group). Data presented as mean ± SEM. The vascular relaxation data were analysed using a generalised estimating equation model followed by least significant difference post hoc testing with *: $p<0.05$; **: $p<0.01$; ****: $p<0.0001$. Statistics on graph shown for most important comparisons only (MCDD + treatment vs MCDD + placebo). ACh, acetylcholine; (Log M), logarithmic concentration in mol/Liter.

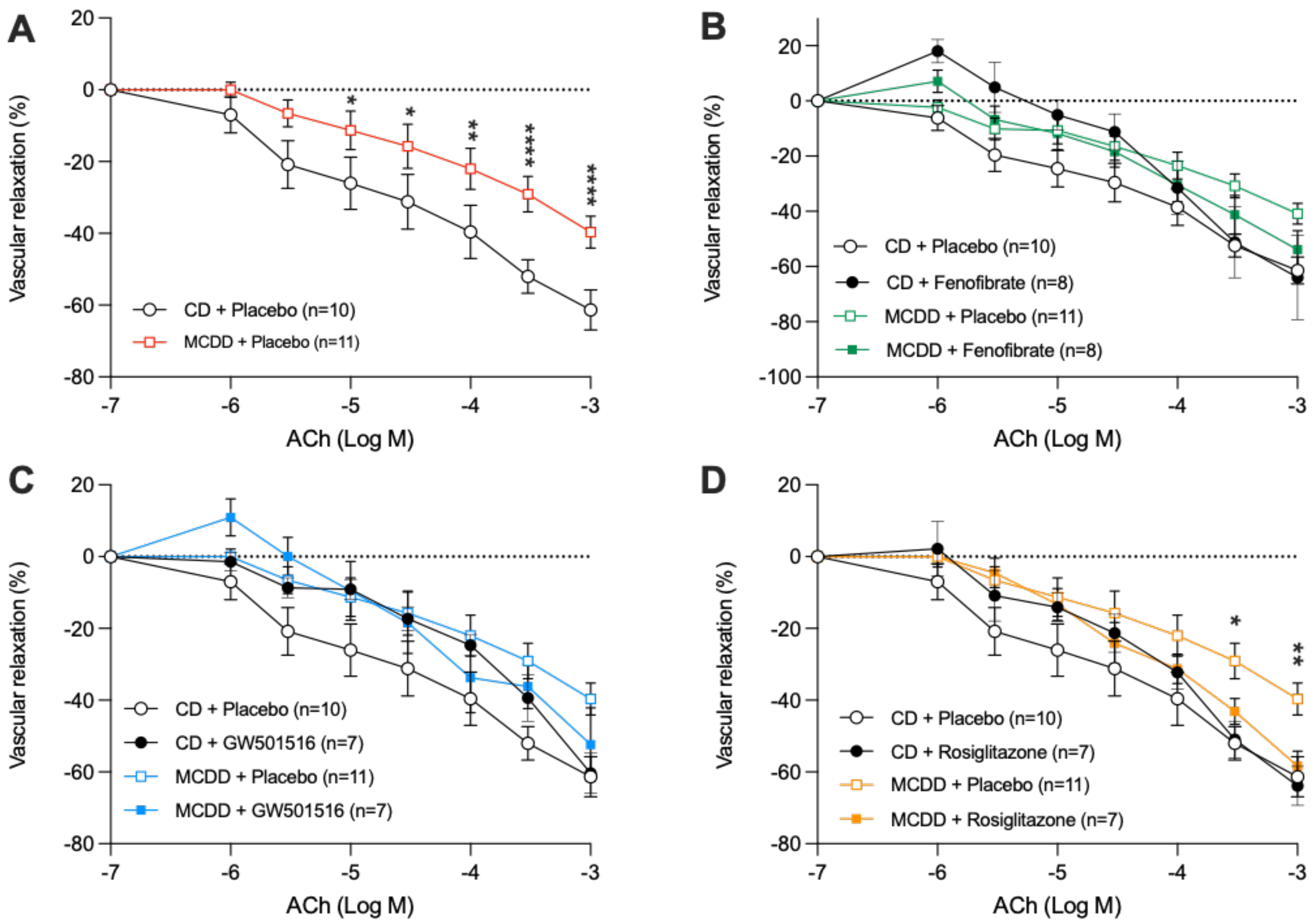

Fig. S20. Qualitative assessment of hepatic vascular corrosion casts acquired by scanning electron microscopy.

A) CD + placebo, **B)** MCDD + placebo, **C)** MCDD + fenofibrate, **D)** MCDD + GW501516**, E)** MCDD + rosiglitazone, **F)** MCDD + lanifibranor)**.** 8 weeks old male Wistar Han rats either fed a chow diet (CD) or a methionine-choline-deficient diet (MCDD) for 4 weeks were preventively treated with either placebo, fenofibrate (30 mg/kg), GW501516 (10 mg/kg), rosiglitazone (5 mg/kg) or lanifibranor (100 mg/kg) daily, QD via oral gavage. n = 3/group. Magnification 300x. Voltage: 20.0 kV.

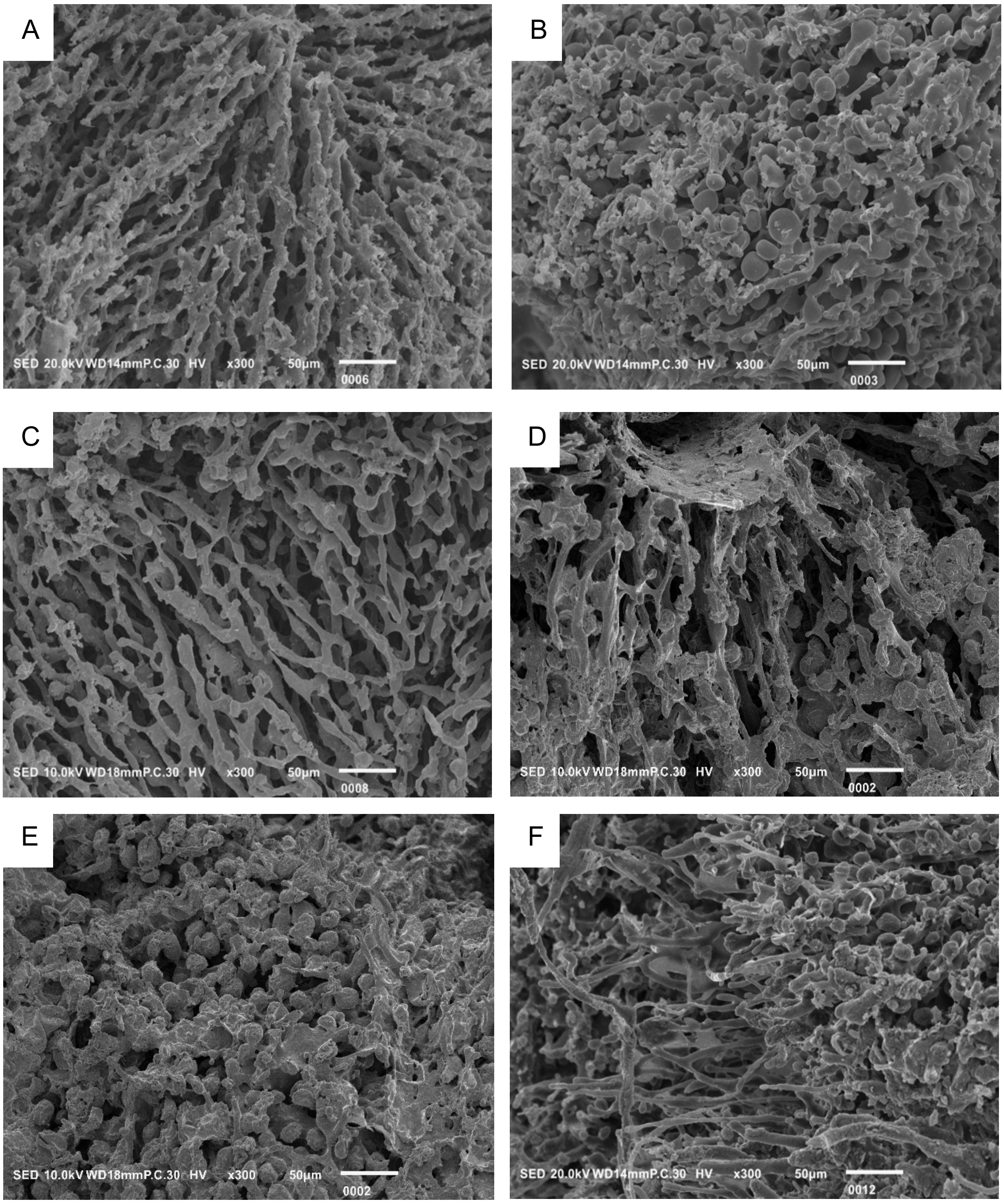

Supplementary tables

Table S1. List of Independent Ethics Committee / Institutional Review Board

Country	CEC or LEC for Site	Ethical Committees (or IRB)	Chair Person
Australia	CEC - all Sites	Monash Health Human Research EthicsCommittee (EC00382): Research Support ServicesMonash Health, Level 2, Block 1, Monash Medical Centre 246 Clayton Road, Clayton, Victoria, 3168. Email: research@monashhealth.org	Deborah DELL (Manager)
Australia	Site 1201	Research Support ServicesMonash Health, Level 2, Block 1, Monash Medical Centre 246 Clayton Road, Clayton, Victoria, 3168. Email: michael.kios@monashhealth.org	Deborah DELL (Manager)
Australia	Site 1202	NALHN Research Governance OfficerResearch Secretariat Northern Adelaide Local Health NetworkLyell McEwin Hospital Clinical Trials Unit, Level 2 Haydown Road Elizabeth Vale SA 5112 Email: healthnalhnrgo@sa.gov.au	Lorraine CICHON
Australia	Site 1203	Research Services Research GovernanceOfficer Executive Administration Lower GroundFloor Dr. James Mayne Building Royal Brisbane and Women's HospitalCampus Herston Qld 4029 Generic Email: RBWH-RGO@health.qld.gov.au www.health.qld.gov.au/metronorth	Janani Balakrishnan MCOM
Australia	Site 1204	Research Governance Officer Southern Adelaide Local Health Network Flinders Medical Centre, Bedford Park SA5042 (WARD 6C Room 6A219) Email: karen.saxty@sa.gov.au http://www.sahealth.sa.gov.au/SALHNresearch	Karen SAXTY BHlthSc (Hons)
Australia	Site 1205	Research Governance OfficerLevel 2, Education Building Fiona Stanley Hospital 14 Barry Marsh ParadeMurdoch, WA 6150 Email: SMHS.RGO@health.wa.gov.au	Cathy GARTNER
Austria	CEC - Site 1001 (no patient screened in Site 1002)	Ethikkommission der MedizinischenUniversität Wien, Borschkegasse 8b/E06,1090 Wien, Österreich Tel: +43(0)1 404 00-21470; Fax: +43(0)1404 00-16900 Email: ethik-kom@meduniwien.ac.at http://ethikkommission meduniwien.ac.at/	Jürgen ZEZULA
Belgium	CEC - Site 0001	Comité voor Medische Ethiek UZA, Wilrijkstraat 10, B-2650 Edegem Tel +32 3 821 30 00 Email: EthischComite@uza.bewww.uza.be	Peter MICHIELSEN
Belgium	LEC - Site 0002	Comité d'Ethique hospitalo-facultaire Promenade de l'Alma 51 bte B1.43.031200 Bruxelles Email: commission.ethique-saintluc@uclouvain.be Tel: 02/ 764 55 14 https://www.saintluc.be/recherche/comite-ethique-accueil.php	Jean-Marie MALOTEAUX
Belgium	LEC - Site 0003	Comité d'éthique Hospitalo-facultaireErasme-ULB Route de Lennik, 8081070 Bruxelles Tel: +32 (0) 2 555 37 07; Fax: +32 (0) 2 555 46 20 Email: Comite.ethique@erasme.ulb.ac.behttp://www.hopitalerasme.be/ethique	Prof. J.-M. BOEYNAEMs
Belgium	LEC - Site 0004	Commissie voor Medische ethiekUniversitair Ziekenhuis Gent, C. Heymanslaan 10,9000 Gent Tel: +32 9 332 22 66 Email: ethisch.comite@uzgent.be https://www.uzgent.be/nl/home/Paginas/home.aspx	Prof. Dr. D.MATTHYS
Belgium	LEC - Site 0005	Comité Medische Ethiek Ziekenhuis Oost-Limburg Secretariaat Comité Medische EthiekSchiepse Bos 6 3600 GENK Tel: 089 32 15 09; Fax: 089 32 79 00 (clear mention CME) Email: ec.submission@zol.be https://www.zol.be/comite-medische-ethiek	Patrick NOYENS
Bulgaria	CEC	Ethics Committee for Clinical Trials, 8, Damyan Gruev str., 1303 Sofia, Bulgaria	Stefan DIMITROV, MD
Bulgaria	LEC - Site 1501	Local Ethics Committee at UMHAT "Sv. Ivan Rilski" EAD, 15 Akad. I. Geshov Blvd.,1431 Sofia, Bulgaria, Tel: +35929523554	Assoc. Prof. ZHELEV
Bulgaria	LEC - Site 1502	Local Ethics Committee at UMHAT "Tsaritsa Yoanna - ISUL" EAD, 8, ByaloMore Str., 1527 Sofia, Bulgaria, Tel: +35929432170	Prof. Boryana DELIISKA

Bulgaria	LEC - Site 1503	Local Ethics Committee at "Acibadem CityClinic MHAT Tokuda"EAD, 51B NikolaVaptzarov Blvd, 1407 Sofia, Bulgaria,<br>Tel.: +35924034000	Rozalina BALABANSKA, MD
Bulgaria	LEC - Site 1504	Local Ethics Committee at UMHAT "SvetaAnna" - Sofia AD, 1, Dimitar Mollov Str., 1750 Sofia, Bulgaria	Nikolay DOBREV, MD
Bulgaria	LEC - Site 1505	Local Ethics Committee at Military Medical Academy - MHAT - Sofia, 3 Georgi Sofiyski Str., 1606 Sofia, Bulgaria	Col. Prof. Ventsislav MUTAFCHIISKI
Bulgaria	LEC - Site 1506	Local Ethics Committee at DCC "AlexanDr.ovska" EOOD, 1 Georgi Sofyiskistr., 1431 Sofia, Bulgaria,<br>Tel: +35929230815	Nataliya TEMELKOVA, MD
Bulgaria	LEC - Site 1507	Acibadem City Clinic University Hospital EOOD, UIC 202139132, with address of business 127 Okolovrasten pat Str., Sofia andcorresponding address of the Second clinical<br>base: 66A Tsarigradsko shosse Blvd., 1784 Sofia, Bulgaria.	Dr Victoria Sasheva Dimitrova Biliana GENOVA*
Canada	Site 1301 and 1306	McGill University Health Centre (MUHC)REB<br>5100, boul. de Maisonneuve Ouest, 5th floorMontréal, Québec, H4A 3T2 Tel: 514-934-1934, ext-71461<br>Email: renaud.boulanger@muhc.mcgill.ca	PAGE Sonya;<br>MANIATIS Thomas;<br>LEBOUCH É Bertrand;<br>BOULANGER Renaud;<br>CANTINI Franca
Canada	Site 1302	Conjoint Health Research Ethics board(CHREB) Research Services Office<br>2500 University Dr.ive NW, Calgary, AB,T2N1N4 Tel: (403) 220-7990; Fax: (403) 289-0693<br>Email: chreb@ucalgary.ca	STACEY A. Page, PhD
Canada	Site 1303	RESEARCH ETHICS OFFICE<br>HEALTH RESEARCH ETHICS BOARD<br>308 Campus Tower, 8625 – 112 St Edmonton, Alberta, Canada T6G IK8 Tel: 780.492.0459; Fax: 780.492.9429<br>Email: reoffice@ualberta.cawww.reo.ualberta.ca	Dr. Shane KIMBER<br>Dr. Glen PEARSON<br>Dr. Don MORRIS H<br>Dr. Robert RENNIE<br>Dr. Anthony JOYCE
Canada	Site 1304	Western University<br>Health Science Research Ethics Board(HSREB)<br>Room 5150 Support Services Building, 1393Western Road<br>London, Ontario, Canada, N6G I G9<br>Tel: 519-661-2161<br>Email: ethics@uwo.ca	GILBERT, Joseph MD (Jones, Philip MD Matsui, Doreen MD)
Canada	Sites 1305 and 1307	Advarra Institutional Review Board (IRB)–Advancing Better Research,<br>Stacey Neshevich phone 905-841-2257 Email: stacey.neshevich@advarra.com 372 Hollandview Trail, Suite 300, Aurora,ON L4G 0A5 Canada	Sara HARNISH, J.D., ExecutiveChair
Czech Republic	Site 0901 Urbanek	Etické komise<br>Fakultní nemocnice v MotoleV úvalu<br>84 150 06 Praha 5 - MotolCzech Republic<br>Tel.: +420 224 431 195; Fax: +420 224 431 196<br>Email: etickakomise@fnmotol.cz	MUDr. Vratislav ŠMELHAUS
Czech Republic	Site 0902 Sperl	Ethics Committee of IKEM and TNThomayer Hospital<br>Vídeňská 800<br>140 59 Prague 4 - KrčCzech Republic<br>Tel: 26108 3481<br>Email: alena.hruba@ftn.cz	Prof. MUDr. Vladimír STANĚK, CSc.
Czech Republic	Site 0903 Hejda	Research Site s.r.o.<br>Slovanská 27<br>326 00 Plzeň<br>Czech Republic<br>Tel - fax: +420 377 320 027<br>Email: eticka.komise@researchSite.cz	MUDr. Luboš JANŮ Ph.D.
France	CEC - all Sites (No LEC) [Sites 0201 to 0217]	Comité de Protection des Personnes Ouest IIIBat. Vie La Santé – 1er étage porte 101 CHU de Poitiers - 2 rue de la milétrie86021 Poitiers Cedex<br>Tel: 05.49.45.21.57; Fax: 05 .49.46.12.62<br>Email: cpp-ouest3@chu-poitiers.fr	Prof. Denis FRASCA

Germany	CEC - 0801<br>Schattenberg	Ethik-Kommission Landesärztekammer Rheinland-PfalzDeutschhausplatz 3 - 55116 Mainz Postfach 29 26 - 55019 Mainz<br>Tel: 06131 28822-62; Fax: 06131 28822-66<br>Email: wagner@laek-rlp.de<br>Web: http://www.laek-rlp.de/ausschuesse-kommissionen/ethikkommission/	Univ.-Prof. Dr. med. Dipl. Ing.<br>Stephan LETZEL
Germany	LEC - 0802<br>Geier	Ethics Committee of the University ofWürzburg<br>Institute for Pharmacology and ToxicologyVersbacher Str. 9<br>97078 Würzburg<br>Tel: 0931 31 48315; Fax: 0931 31 87520<br>Email: ethikkommission@uni-wuerzburg.de	Prof. Dr. med.<br>R.JAHNS
Germany	LEC - 0803<br>Merle	Ethikkommission HeidelbergAlte Glockengießerei 11/1 69115 Heidelberg Tel: +49 6221 56264-72; Fax: +49 6221 56264-80<br>Email: Marion.Teichmann@med.uni-heidelberg.de<br>http://www.medizinische-fakultaet-hd.uni-heidelberg.de/ethikkommission	Dr. med. Dr. hc Thomas STROWITZKI
Germany	LEC - 0805<br>Trautwein	Ethik-Kommission an der Medizinischen Fakultät der Rheinisch-Westfälischen Technischen Hochschule Aachen (RWTHAachen).<br>Universitätsklinikum AachenPauwelsstraße 30<br>52074 Aachen.<br>Tel: +49 241 80-89963; Fax: +49 241 80-82012<br>Email: ekaachen@ukaachen.de	Günther<br>SCHMALZING
Germany	LEC 0807-<br>Boettler	ETHIK-KOMMISSION<br>Albert-Ludwigs-Universität Freiburg Engelberger Straße 21, 79106 Freiburg Tel: (+49) 0761 270-72500;<br>Fax: (+49) 0761 270-72630<br>Email: ekfr.amg@uniklinik-freiburg.de<br>http://www.ethik-kommission.uniklinik-freiburg.de	Prof. Dr. Rudolf<br>KORINTHENBERG
Germany	LEC - 0808<br>Heinzow	Ethics Commission of the MedicalAssociation Westphalia-Lippe and the Westphalian Wilhelms University ofMünster<br>Gartenstrasse 210 - 214D-48147 Münster Tel: +49 (0) 2 51/929 – 2460;<br>Fax: +49 (0) 2 51/929 - 2478<br>Email: ethikkommission @ aekwl.de	Univ.-Prof. Dr. med. Wolfgang E. BERDEL
Italy	CEC - 0101<br>Bugianesi	Comitato Etico Interaziendale A.O.U. Cittàdella Salute e della Scienza di Torino/ A.O.Ordine Mauriziano/ASL Città di Torino Corso Bramante 88/90<br>10126 - Torino<br>Tel: 011.633.6820; Fax: 011.633.4171<br>Email: comitatoetico@cittadellasalute.to.it	Dr. Marcello<br>MADDALENA
Italy	LEC - 0102<br>Svegliati	Comitato Etico Regionale delle Marche Azienda Ospedaliero-Universitaria OspedaliRiuniti di Ancona<br>Via Conca n. 71<br>60126 Torrette di Ancona<br>Tel: +39 071 596 3667<br>Email: comitato.etico@ospedaliriuniti.marche.it	Prof. Paolo PELAIA
Italy	LEC - 0103<br>Miele	Comitato Etico<br>Fondazione Policlinico Universitario A.Gemelli<br>Università Cattolica del Sacro CuoreLargo Agostino Gemelli 8 00168 Roma<br>Fondazione Policlinico UniversitarioAgostino Gemelli IRCCS<br>Università Cattolica del Sacro Cuore Largo Agostino Gemelli 8, 00168 Roma Tel: +39 06 30155556; Fax: +39 06 30155345<br>Email: comitato.etico@policlinicogemelli.it	Prof. Gigliola SICA
Italy	LEC - 0105<br>Craxi	Comitato Etico Palermo 1Via del Vespro 129 90127 Palermo Tel: 0916555210; Fax: 0916553747<br>Email: bioetica@policlinico.pa.it	Prof. Salvatore LEONE
Italy	LEC - 0107<br>Lampertico	Comitato Etico Milano Area B Ospedale Maggiore Policlinico,Via F. Sforza n. 28 20122 Milano<br>Tel 02-55032982; Fax 02-55036618<br>Email federica.massacesi@policlinico.mi.it	Gaetana MUSERRA
Italy	LEC - 0108<br>Mangia	Comitato etico<br>Fondazione Casa Sollievo della SofferenzaIRCCS<br>Opera di San Pio da Pietrelcina 71013 San Giovanni Rotondo FG Tel 0882 410831; Fax 0882410813<br>Email comitatoetico@operapaDr.epio.it	Luigi RENNA
Mauritius	1401	Name: Ethics committee<br>AdDr.ess: Bacha Building, 2nd floor, PortLouis Tel: +230 52553636, +230 57525264<br>Fax: not available<br>email: kdhurmah@govmu.org	Dr. Satyabhoosun DOMAH
	CEC and LEC - 0701	Komisja Bioetyczna Uniwersytetu Medycznego w Białym Stoku, Kilińskiego 1,15-	

Poland	/Flisiak (National Coordinator)/ - Site did not screen patients	089 Białystok<br>Tel: +48 85 748 54 07<br>Fax: +48 85 748 55 08<br>Email: prorektorkl@umb.edu.pl	Prof. Dr. hab. Otylia Kowal-BIELECKA
Poland	LEC - 0702 /Tomasiewicz/	Komisja Bioetyczna przy Uniwersytecie Medycznym w Lublinie, Al. Racławickie 1,20-059 Lublin;<br>Tel.: +48 81 448 52 13;<br>Fax: +48 81 448 52 11; Email:komisja.bioetyczna@umlub.pl	Dr. hab. n. med. Marcin OLAJOSSY
Poland	LEC - 0703 /Piekarska/	Komisja Bioetyki ds. Badań na ludziach przyUniwersytecie Medycznym, Pl. Hallera 1B, 90-647 Łódź;<br>Tel: +48 42 272 52 43, +48 42 272 52 44,<br>+48 785 911 596;<br>Email: bioetyka@umed.lodz.pl	Prof. Dr. hab. Józef Dr. ZEWOSKI
Poland	LEC - 0704 /Napora/	Dolnośląska Izba Lekarska we Wrocławiu Komisja Bioetyczna, Kazimierza Wielkiego45, 50- 077 Wrocław;<br>Tel. +48 71 798 80 74, +48 607 552 143;<br>Email: kb@dilnet.wroc.pl	Dr. n. med. Włodzimierz BEDNORZ
Poland	LEC - 0705 /Hartleb/	Komisja Bioetyczna Śląskiego UniwersytetuMedycznego w Katowicach, Poniatowskiego15, 40- 055 Katowice;<br>Tel. +48 32 208 36 42, +48 32 208 35 46;<br>Fax: +48 32 208 36 94;<br>Email: kombioet@sum.edu.pl	Prof. Dr. hab. n. med. Bogusław OKOPIE Ń
Slovenia	Site 1701	Republic of Slovenia National MedicalEthics Committee, Ministry of Health, Štefanova ulica 5, SI-1000 Ljubljana;<br>Tel: +386 01 478 69 13; Fax: +386 01 478 60 58;<br>Email: kme.mz@gov.si	Dr. Božidar VOLJC
Slovenia	Site 1702	Republic of Slovenia National Medical Ethics Committee, Ministry of Health, Štefanova ulica 5, SI-1000 Ljubljana; Tel:<br>+386 01 478 69 13; Fax: +386 01 478 60 58;<br>Email: kme.mz@gov.si	Dr. Božidar VOLJC
Spain	CEC - Sites 0501; 0502; 0503; 0504; 0505	CEIm Provincial de Sevilla<br>Avda. Dr. FeDr.iani, 3 – Unidad de Investigación 2ª planta Sevilla 41009<br>SevillaEspaña Tel. 600 162 458; Fax. 955 00 80 15<br>Email: administracion.eecc.hvm.sspa@juntadeandalucia.es	Dr. Víctor Sánchez MARGALET
Switzerland	CEC Site 0401	Kantonale Ethikkommission für<br>dieForschung Murtenstrasse 31<br>3010 Bern<br>Tel: +41 31 633 70 70; Fax: +41 31 633 70 71<br>Email: info.kek.kapa@gef.be.chwww.be.ch/kek	Dr. med. Christian SEILER
Switzerland	Site 0402	Commission Cantonale d'éthique de larecherche Genève<br>(CCER) Rue ADr.ien-Lachenal 8<br>1207 Genève<br>Tel: +41 22 5465101; Email: ccer@etat.ge.ch	Prof. Bernard HIRSCHEL
Switzerland	Site 0403	Comitato etico cantonale Ticinoc/o Ufficio di<br>sanità Via Orico 5<br>6501 Bellinzona<br>Tel: +41 91 814 30 57; Email: dss-ce@ti.ch	Giovan Maria ZANINI
UK	REC - Sites 0301; 0302; 0303	North East - Newcastle & North Tyneside 1Research Ethics<br>Committee NHSBT Newcastle Blood Donor CentreHolland Drive<br>Newcastle upon TyneNE2<br>4NQ Tel: 0207 104 8089<br>Email: newcastlenorthtyneside1.rec@hra nhs.uk	Mr Paddy STEVENSO N
USA	LEC - Site 1607	DUHS Institutional Review Board2424 Erwin<br>Road Durham NC 919-668-5111	John FALLETTA
USA	LEC - Site 1603	IRB for Health Sciences Research University of Virginia PO box<br>800483Charlottesville VA 434-924-9634	MEDARD H.T. Ng
USA	LEC - Site 1612	UCSD Institutional Review Boards9452 Medical Center<br>Drive La Jolla CA 858-246-4777	Kip KANTELO
USA	LEC - Site 1604	Advarra IRB<br>6940 Columbia Gateway Drive #110 Columbia<br>MD 410-884-2900	Tony DAVIS
USA	CEC - Sites 1609, 1616,1611, 1613, 1602, 1605, 1614, 1615, 1601, 1606	WIRB Copernicus IRB<br>5000 Centregreen Way STE 200Cary<br>NC 888-303-2224	Donald A. DEIESO

*The activity of the Ethics committee for medical and scientific research established at Acibadem City Clinic University hospital EOOD terminated on 15 October 2018; NA: Not applicable; UK: United Kingdom; USA: United States of America.
Abbreviation: CEC, Central Ethics Committee; LEC, Local Ethics Committee

Table S2. Selection of the additional 76 patients not randomised in NATIVE, but with available liver tissue remaining from the screening period,
Among the NATIVE screening failure patients with either: i) severe fibrosis and severe activity, ii) mild fibrosis and mild activity, iii) severe fibrosis and mild activity,or iv) mild fibrosis and severe activity (black, n=207), 76 patients were chosen (red) to obtain a representative sample of mild, moderate and severe steatosis, MASH activity, and fibrosis.

Activity	Fibrosis	Steatosis								Total available	Total selected
		S0		S1		S2		S3			
A0	F0	16	**8**	10	**7**	2	**2**	2	**2**	30	**19**
A1	F0	2		10		12		9		33	**0**
A2	F0	0		7	**1**	11	**2**	9	**2**	27	**5**
A2	F1	0		8	**1**	21	**4**	29	**5**	58	**10**
A3	F0	0		0		0		0		0	**0**
A3	F1	0		0		1	**1**	4	**4**	5	**5**
A0	F2	0		0		0		0		0	**0**
A1	F2	1	**1**	6	**6**	7	**7**	0		14	**14**
A0	F3	1	**1**	1	**1**	0		0		2	**2**
A1	F3	0		2	**2**	1	**1**	0		3	**3**
A3	F2	0		3		2		12	**5**	17	**5**
A3	F3	0		2		3		4	**4**	9	**4**
A4	F2	0		0		0		5	**5**	5	**5**
A4	F3	0		0		2	**2**	2	**2**	4	**4**
**TOTAL**										207	**76**

Table S3. Clinical features at baseline.

Parameter	Screening failures N=76	Randomized patients			
		All N=173	Placebo N=56	Lanifibranor 800mg N=57	Lanifibranor 1200mg N=60
**Demographic and clinical characteristics**					
Age – yrs	54 (45; 62)	56 (49; 62)	54 (46; 62)	58 (50; 63)	56 (49; 62)
Female sex – no. (%)	37 (49%)	100 (58%)	29 (52%)	34 (60%)	37 (62%)
Weight – kg	87 (79; 99)	92 (81; 107)	93 (82; 107)	94 (81; 106)	89 (79; 108)
Body-mass index – kg/m²	31.1 (28.1; 35.3)	32.9 (29.4; 36.3)	33.3 (29.4; 36.6)	32.2 (29.4; 35.9)	33.3 (29.6; 37.5)
Body-mass index > 25 (%)	68 (89%)	164 (95%)	52 (93%)	55 (96%)	57 (95%)
Body-mass index > 30 (%)	41 (54%)	120 (69%)	39 (70%)	37 (65%)	44 (73%)
Arterial hypertension – %	34 (45%)	106 (61%)	31 (55%)	39 (68%)	36 (60%)
Type 2 diabetes mellitus – no. (%)	28 (37%)	72 (42%)	22 (39%)	25 (44%)	25 (42%)
Time between screening biopsy and randomization - months	NA	2.3 (1.3; 3.5)	2.2 (1.3; 3.4)	2.0 (1.2; 3.1)	2.4 (1.5; 3.6)
**Histological parameters**					
Steatosis grade*	2 (1; 3)	3 (2; 3)	3 (2; 3)	3 (2; 3)	3 (2; 3)
S0 – no. (%)	10 (13%)	0 (0%)	0 (0%)	0 (0%)	0 (0%)
S1 – no. (%)	18 (24%)	15 (9%)	4 (7%)	5 (9%)	6 (10%)
S2 – no. (%)	19 (25%)	45 (26%)	17 (30%)	11 (19%)	17 (28%)
S3 – no. (%)	29 (38%)	113 (65%)	35 (63%)	41 (72%)	37 (62%)
Lobular inflammation grade**	1 (0; 1)	1 (1; 2)	2 (1; 2)	2 (1; 2)	1 (1; 2)
I0 – no. (%)	25 (33%)	0 (0%)	0 (0%)	0 (0%)	0 (0%)
I1 – no. (%)	35 (46%)	88 (51%)	27 (48%)	28 (49%)	33 (55%)
I2 – no. (%)	14 (18%)	76 (44%)	27 (48%)	26 (46%)	23 (38%)
I3 – no. (%)	2 (2%)	9 (5%)	2 (4%)	3 (5%)	4 (7%)
Ballooning grade***	1 (0; 1)	2 (2; 2)	2 (2; 2)	2 (1; 2)	2 (2; 2)
B0 – no. (%)	35 (46%)	0 (0%)	0 (0%)	0 (0%)	0 (0%)
B1 – no. (%)	23 (30%)	40 (23%)	12 (21%)	17 (30%)	11 (18%)
B2 – no. (%)	18 (24%)	133 (77%)	44 (79%)	40 (70%)	49 (82%)
Fibrosis stage****	1 (0; 2)	2 (2; 3)	2 (1; 3)	2 (2; 3)	2 (2; 3)
F0 – no. (%)	24 (32%)	4 (2%)	3 (5%)	0 (0%)	1 (1%)
F1 – no. (%)	15 (20%)	36 (21%)	13 (23%)	10 (18%)	13 (22%)
F2 – no. (%)	23 (30%)	85 (49%)	26 (46%)	32 (56%)	27 (45%)
F3 – no. (%)	14 (18%)	48 (28%)	14 (25%)	15 (26%)	19 (32%)
Fibrosis stage F2 or F3 – no. (%)	37 (49%)	133 (77%)	40 (71%)	47 (82%)	46 (77%)
SAF-A score	2 (0; 3)	3 (3; 4)	3 (3; 4)	3 (3; 3)	3 (3; 4)
NAS	3 (2; 6)	6 (5; 6)	6 (5; 6)	6 (6; 6)	6 (5; 6)

**NAS≥6 no. (%)**	22 (29%)	123 (71%)	39 (70%)	43 (75%)	41 (68%)
**Plasma biochemistry parameters**					
**Serum alanine aminotransferase – IU/L**	41 (27; 63)	50 (35; 76)	43 (32; 76)	50 (38; 75)	52 (35; 79)
**Serum aspartate aminotransferase – IU/L**	28 (23; 40)	37 (29; 54)	35 (26; 51)	37 (29; 56)	41 (29; 55)
**Serum γ-Glutamyltransferase– IU/L**	46 (31; 76)	44 (30; 69)	44 (29; 71)	48 (31; 79)	39 (31; 59)
**Fasting HDL cholesterol level – mmol/L**	1.08 (0.91; 1.44)	1.17 (0.99; 1.40)	1.14 (0.92; 1.30)	1.18 (1.07; 1.43)	1.16 (0.98; 1.42)
**Fasting triglycerides – mmol/L**	1.81 (1.27; 2.59)	1.76 (1.32; 2.41)	1.93 (1.38; 2.44)	1.70 (1.35; 2.30)	1.63 (1.29; 2.54)
**Fasting glucose level – mmol/L**	5.39 (4.89; 6.29)	5.66 (5.07; 6.58)	5.37 (5.11; 6.43)	6.00 (5.07; 7.16)	5.65 (5.03; 6.36)
**Glycated hemoglobin level – %**	6 (5; 6)	6 (6; 7)	6 (5; 6)	6 (6; 7)	6 (6; 7)
**Fasting insulin level – pmol/L**	NA	190.8 (141.5; 261.3)	180.3 (132.0; 224.3)	196.5 (145.8; 251.2)	197.6 (131.4; 314.3)
**Cytokeratin 18 M65 – pmol/L**	NA	460 (248 ; 817)	336 (248 ; 675)	503 (334 ; 858)	512 (248, 911)
**FIB-4 score**	0.97 (0.74 ; 1.34)	1.20 (0.79 ; 1.97)	1.14 (0.65 ; 1.90)	1.37 (0.94 ; 2.0)	1.09 (0.73 ; 1.77)
**FIB-4 classes**<br>**≤ 1.3**<br>**> 1.3 and ≤ 3.25**<br>**> 3.25**	 55 (72%)<br>18 (24%)<br>3 (4%)	 95 (55%)<br>72 (42%)<br>5 (3%)	 34 (61%)<br>20 (36%)<br>2 (4%)	 27 (47%)<br>28 (49%)<br>2 (4%)	 34 (58%)<br>24 (41%)<br>1 (2%)
**FibroScan® values**					
**LSM - kPa**	6.8 (5.7; 9.7)	8.8 (6.8; 11.6)	8.6 (6.9; 10.8)	9.9 (7.8; 11.7)	8.4 (6.1; 12.0)
**CAP- dB m^{-1}**	322 (279; 361)	328 (300; 359)	325 (301; 376)	330 (295; 354)	327 (297; 357)
**Treatments**					
**At least one antidiabetic drug**	29 (38%)	64 (37%)	21 (38%)	22 (39%)	21 (35%)
**Statin (alone or in combination) – no. (%)**	13 (17%)	32 (18%)	8 (14%)	11 (19%)	13 (22%)

If not stated otherwise, data is presented as median (interquartile range).
*Steatosis was assessed as the percentage of hepatocytes containing large and medium-sized intracytoplasmic lipid droplets and graded as 0 (<5%), 1 (5 to 33%), 2 (34 to 66%), or 3 (≥67%), according to the Nonalcoholic Steatohepatitis Clinical Research Network (NASH CRN) grading system.
**Lobular inflammation was classified as grade 0 (no foci), grade 1 (<2 foci per 200x field) or grade 2 (2-4 foci per 200x field), according to the NASH CRN scoring system.
***Ballooning was classified as grade 0 (no balloon hepatocyte) grade 1 (few but definite ballooned hepatocytes) or grade 2 (prominent ballooning), according to the NASH CRN grading system.

****Fibrosis was classified as stage F0 (no fibrosis), stage F1 (mild fibrosis), stage F2 (significant fibrosis), stage F3 (advanced fibrosis), or stage F4 (cirrhosis), according to the SAF–NASH CRN staging system.

Abbreviations: SAF-A: The SAF-Activity (SAF-A) score ranges from 0 to 4; with higher scores indicating more-severe disease activity; NAS: The Nonalcoholic Fatty Liver Disease Activity Score (NAS) ranges from 0 to 8. A score of 2 or less indicates “not NASH”; a score of 3 or 4, “borderline NASH” and a score of 5 to 8, “definite NASH”. LSM: Liver Stiffness Measure assessed by FibroScan®; CAP: Controlled Attenuation Parameter assessed by FibroScan®.

Table S4: Relationship between clinical parameters and density of CD34 positive vessels, periportal score and lobular score at baseline

Parameter	Density of CD34 positive vessels		Periportal score		Lobular score	
	Test	p-value	Test	p-value	Test	p-value
**Demographic and clinical characteristics**						
**Age**	Sp: r: 0.064	0.312	W	0.459	W	0.268
**Sex**	W	0.806	Chi²	0.698	Chi²	0.137
**Weight**	Sp: r: -0.062	0.333	W	0.484	W	0.312
**Body Mass Index**	Sp: r: -0.087	0.172	W	0.995	W	0.239
**Body Mass Index ≥25**	W	0.89	F	0.581	Chi²	0.805
**Body Mass Index ≥30**	W	0.646	Chi²	0.406	Chi²	0.495
**All classes Body Mass Index**	KW	0.218	CA	0.766	CA	0.183
**Arterial hypertension**	W	0.183	Chi²	0.704	Chi²	0.615
**Type 2 Diabetes Mellitus**	W	0.968	Chi²	0.214	Chi²	0.875
**Plasma biochemistry parameters**						
**Serum AST**	Sp: r:0.161	**0.011**	W	0.668	W	**0.006**
**Serum ALT**	Sp: r: 0.119	0.062	W	0.609	W	**0.013**
**Serum γ-Glutamyltransferase**	Sp: r: 0.131	**0.039**	W	0.978	W	0.147
**Fasting HDL cholesterol**	Sp: r: -0.003	0.961	W	0.757	W	0.164
**Fasting triglycerides**	Sp: r: 0.073	0.256	W	0.901	W	0.532
**Fasting glucose**	Sp: r: 0.037	0.563	W	0.951	W	0.672
**Glycated hemoglobin**	Sp: r: 0.049	0.443	W	0.72	W	0.471
**Fasting insulin level***	Sp: r: -0.011	0.888	W	0.259	W	0.663
**CK18 M65***	Sp: r: 0.125	0.105	W	0.663	W	**0.046**
**FIB-4**	Sp: r: 0.164	**0.01**	W	0.835	W	0.136
**FIB-4 classes**	W	**0.004**	CA	0.792	CA	0.064
**FibroScan® values**						
**CAP**	Sp: r: -0.028	0.704	W	0.085	W	0.427
**LSM**	Sp: r: 0.212	**0.002**	W	0.439	W	0.094
**Treatments**						
**Anti-diabetic drugs**	W	0.816	Chi²	0.192	Chi²	0.511
**Statins**	W	0.811	Chi²	0.747	Chi²	0.646

Density of CD34 positive vessels, of periportal score and of lobular score were available in 248, 246 and 245 patients, respectively, as detailed in Fig S3. P-values are displayed for the following tests: CA: Cochran-Armitage test for trend (ordered vs. 2 modalities categorial parameters); Chi^2: categorial parameters (all expected values > 5); F: Fisher test (categorial parameters, at least one expected value ≤ 5); Sp: Spearman correlation test (continuous), r: Spearman correlation coefficient; W: Wilcoxon (continuous non-normally distributed vs. 2

modalities categorial parameters) (non-N distribution; KW); * Data available only for the 173 randomized patients.

AST, Aspartate aminotransferase; ALT, Alanine Aminotransferase; HDL, high density lipoprotein; CK18, cytokeratin 18; CAP, controlled attenuation parameter assessed by Fibroscan®; LSM, liver stiffness measurement assessed by FibroScan®. Bold indicates p < 0.05. Colors represent the p-value, with red (0) to green (1).

Table S5. Baseline characteristics and haemodynamics of rats fed a chow – or methionine-choline-deficient diet.

**Chow diet-fed rats**	Groups (n/n/n/n/n)	Placebo	Fenofibrate (30mg/kg)	GW501516 (10 mg/kg)	Rosiglitazone (5 mg/kg)	Lanifibranor (100 mg/kg)
Age (weeks)	58/38/38/38/38	8	8	8	8	8
Body weight (g): baseline	50/24/38/38/38	251.0 (242.8 – 263.3)	257.0 (248.5 – 267.0)	250.5 (247.0 – 258.0)	253.0 (244.8 – 257.3)	249.5 (243.0 – 257.0)
Body weight (g): after 4 weeks	58/38/37/37/38	349.0 (328.3 – 360.3)*	340.0 (319.8 – 354.0)#	330.0 (316.5 – 340.5)#	331.0 (321.0 – 350.0)#	338.5 (326.3 – 373.0)#
∆ Weight (g)	50/24/37/37/38	92.5 (72.0 – 104.5)*	79.0 (69.0 – 85.8)#	78.0 (67.0 – 86.0)#	79.0 (69.5 – 93.5)#	87.0 (69.3 – 103.3)#
Liver weight (g)	54/38/37/38/38	10.5 (9.3 – 11.3)	12.6 (11.3 – 14.5)°	11.5 (10.5 – 12.9)	9.9 (8.5 – 11.2)	10.4 (9.6 – 11.1)
% liver/total body weight	56/38/37/37/38	3.0 (2.9 – 3.2)*	4.6 (4.4 – 5.1)°	3.5 (3.2 – 4.0)#	2.9 (2.6 – 3.2)#	3.0 (2.9 – 3.2)#
MABP (mmHg)	43/26/28/29/31	123.7 (106.3 – 130.8)	144.9 (129.8 – 153.2)°	110.1 (100.0 – 121.5)	122.6 (96.9 – 134.3)	110.7 (101.1 – 122.1)#
In vivo PVP (mmHg)	46/26/27/28/31	3.5 (3.2 – 3.9)*	3.7 (3.4 – 4.1)	3.5 (3.1 – 3.9)#	3.5 (3.2 – 3.6)#	3.3 (3.1 – 3.7)
Portal blood flow (mL/min)	38/25/26/26/28	12.5 (11.0 – 15.1)*	13.5 (11.3 – 14.8)	12.7 (11.4 – 14.3)	12.6 (10.9 – 15.8)	13.8 (12.1 – 15.1)#
THPG (mmHg) at 10 mL/min	8/8/8/8/8	3.7 ± 0.1	3.7 ± 0.2	3.5 ± 0.1	3.8 ± 0.2	3.5 ± 0.1
THPG (mmHg) at 30 mL/min	8/8/8/8/8	6.6 ± 0.2	7.0 ± 0.4	6.5 ± 0.3	6.7 ± 0.6	7.4 ± 0.4

**MCDD-fed rats**	Groups (n/n/n/n/n)	Placebo	Fenofibrate (30 mg/kg)	GW501516 (10 mg/kg)	Rosiglitazone (5 mg/kg)	Lanifibranor (100 mg/kg)
Age (weeks)	64/38/38/38/38	8	8	8	8	8
Body weight (g): baseline	50/24/38/38/38	251.0 (242.8 – 263.3)	260.0 (228.3 – 274.8)	250.0 (247.8 – 258.0)	251.5 (240.8 – 259.8)	250.0 (243.5 – 258.0)
Body weight (g): after 4 weeks	62/38/37/38/38	210.0 (202.0 – 219.3)	205.0 (198.0 – 211.8)	211.0 (202.0 – 218.0)	213.0 (208.0 – 220.3)	211.0 (203.8 – 215.0)
∆ Weight (g)	50/24/38/38/38	-43.5 (-50.0 – -38.75)	-50.5 (-60.0 – -37.5)	-42.0 (-44.5 – -36.8)	-36.0 (-44.0 – -29.0)	-43.5 (-46.0 – -35.0)
Liver weight (g)	60/38/37/38/37	10.5 (9.3 – 11.3)	8.1 (7.6 – 8.7)*	11.4 (9.7 – 13.4)	9.5 (8.8 – 9.9)	9.7 (9.2 – 11.1)
% liver/total body weight	60/38/37/38/37	4.6 (4.4 – 5.1)	3.9 (3.7 – 4.2)*	5.3 (4.8 – 6.1)	4.4 (4.1 – 4.6)	4.7 (4.4 – 5.1)
MABP (mmHg)	40/28/29/25/37	121.9 (115.3 – 132.3)	120.0 (110.0 – 134.2)	100.5 (83.7 – 109.7)*	118.1 (100.5 – 127.1)	92.7 (78.1 – 104.1)*
In vivo PVP (mmHg)	40/27/26/24/37	5.6 (5.1 – 6.4)	4.5 (3.5 – 5.3)*	4.6 (3.9 – 4.9)	4.8 (4.1 – 5.5)	3.7 (3.2 – 4.0)*
Portal blood flow (mL/min)	34/27/25/23/34	10.1 (9.2 – 11.3)	10.8 (9.5 – 12.6)	11.7 (10.4 – 13.4)	11.1 (9.8 – 14.0)	10.8 (9.2 – 12.7)
THPG (mmHg) at 10 mL/min	7/8/8/8/8	4.8 ± 0.2	3.2 ± 0.2*	4.0 ± 0.2	4.3 ± 0.3	3.5 ± 0.1*
THPG (mmHg) at 30 mL/min	7/8/8/8/8	8.3 ± 0.4	6.6 ± 0.2*	6.7 ± 0.3	8.0 ± 1.0	6.3 ± 0.3*

Male Wistar Han rats of 8 weeks old (n = 6-8/group) were either fed a chow diet (CD) or a methionine-choline-deficient diet (MCDD) for 4 weeks and simultaneously treated with either placebo, fenofibrate (30 mg/kg), GW501516 (10 mg/kg), rosiglitazone (5 mg/kg) or lanifibranor (100 mg/kg) daily QD via oral gavage. Pooled data were analysed using Kruskal-Wallis followed by Dunn test and presented as median (IQR). The THPG data were analysed using a generalised estimating equation model followed by least significant difference post hoc testing when appropriate. * for comparison with MCDD + placebo; ° CD + treatment vs. CD + placebo; # CD + treatment vs. MCDD + treatment counterparts. */°/#: $p<0.05$. MABP, mean arterial blood pressure; PVP, portal venous pressure; THPG, transhepatic pressure gradient.

Table S6. Blinded semi-quantification in early MASLD of CD34 liver sinusoidal endothelial marker immunostained sections.

8 weeks old rats fed chow- (CD) or methionine - choline - deficient diet (MCDD) for 4 weeks were preventively treated with placebo, fenofibrate (30 mg/kg), GW501516 (10 mg/kg), rosiglitazone (5 mg/kg) or lanifibranor (100 mg/kg). n = 5-6/group. Data presented as mean ± SEM. Two-way ANOVA was performed followed by post hoc Tukey, with * p<0.05; **: p<0.01; ***: p<0.001. p* towards placebo-treated MCDD.

CD34 quantification	Placebo	Lanifibranor	Fenofibrate	GW501516	Rosiglitazone
CD	20.9 ± 2.8**	14.3 ± 2.5	8.5 ± 2.0	10.2 ± 3.1	13.5 ± 1.6
MCDD	38.8 ± 2.5	21.4 ± 5.7*	12.7 ± 4.0****	16.5 ± 3.3***	34.5 ± 4.2

Table S7. *In situ ex vivo* liver perfusion in early MASLD: relative change of transhepatic pressure gradient at a dose of 3 × 10^{-9} mol/L ET-1.

Male Wistar Han rats of 8 weeks old were either fed a chow diet (CD) or a methionine-choline-deficient diet (MCDD) for 4 weeks and simultaneously treated with either placebo, fenofibrate (30 mg/kg), GW501516 (10 mg/kg), rosiglitazone (5 mg/kg) or lanifibranor (100 mg/kg) daily, QD via oral gavage. n = 7-8/group). The THPG data were analysed using a generalised estimating equation model followed by least significant difference post hoc testing. Data presented as mean ± SEM.* for comparison with MCDD + placebo; ° CD + treatment vs. CD + placebo. */°: $p<0.05$. M, molar. ET-1, endothelin-1.

ET-1 dose-response	Group (n)	ΔTHPG at 3 x 10^{-9} M ± SEM (mmHg)
CD	Placebo (11)	12.0 ± 1.5*
	Fenofibrate (8)	20.5 ± 1.4°
	GW501516 (7)	9.7 ± 1.1
	Rosiglitazone (8)	7.9 ± 1.2°
	Lanifibranor (8)	10.7 ± 1.1
MCDD	Placebo (11)	17.4 ± 1.4
	Fenofibrate (8)	21.3 ± 1.1*
	GW501516 (8)	14.4 ± 1.8
	Rosiglitazone (8)	15.4 ± 1.5
	Lanifibranor (8)	15.1 ± 1.2

Table S8. *In situ ex vivo* liver perfusion in early MASLD: EC50 and Emax from dose-response curves with methoxamine.

Male Wistar Han rats of 8 weeks old were either fed a chow diet (CD) or a methionine-choline-deficient diet (MCDD) for 4 weeks and simultaneously treated with either placebo, fenofibrate (30 mg/kg), GW501516 (10 mg/kg), rosiglitazone (5 mg/kg) or lanifibranor (100 mg/kg) daily QD via oral gavage. n = 7-11/group). Nonlinear regression (curve fit) was performed to calculate EC50 and Emax. Data were analysed using two-way ANOVA followed by post hoc Tukey and presented as mean ± SEM. * for comparison with MCDD + placebo. *: p<0.05. M, molar.

Methoxamine dose-response	Group (n)	EC50 ± SEM (M)	Emax ± SEM (mmHg)
CD	Placebo (8)	1.8e-005 ± 2.1e-006	7.8 ± 0.6*
	Fenofibrate (8)	2.2e-005 ± 1.8e-006	7.6 ± 0.7
	GW501516 (6)	2.0e-005 ± 3.1e-006	8.5 ± 1.3
	Rosiglitazone (8)	1.5e-005 ± 1.9e-006	6.5 ± 0.4
	Lanifibranor (8)	2.8.e-005 ± 6.3e-006	7.7 ± 1.0
MCDD	Placebo (12)	1.2e-005 ± 8.0e-007	10.8 ± 0.9
	Fenofibrate (8)	1.9e-005 ± 2.6e-006	9.7 ± 0.7
	GW501516 (8)	2.2e-005 ± 2.5e-006	9.3 ± 1.2
	Rosiglitazone (8)	1.8e-005 ± 2.1e-006	8.8 ± 1.0
	Lanifibranor (8)	1.7e-005 ± 2.7e-006	8.7 ± 1.3

Table S9. *In situ ex vivo* liver perfusion in MASH: EC50 and Emax from dose-response curves with methoxamine.

8 weeks old male Zucker fatty rats fed a high-fat high-fructose (HFHFD) diet and 8 weeks old male Zucker lean rats fed a chow diet (CD) were preventively treated with either placebo or lanifibranor (100 mg/kg) daily QD via oral gavage during the complete period of 8 weeks of diet. Data is presented as mean ± SEM. Nonlinear regression (curve fit) was performed to calculate EC50 and Emax, followed by two-way ANOVA and post hoc Tukey with *: p<0.05. * for comparison with MCDD + placebo.

Methoxamine dose-response	Group (n)	EC50 ± SEM (M)	Emax ± SEM (mmHg)
CD	Placebo (8)	6.4e-005 ± 1.4e-005	8.2 ± 0.5
	Lanifibranor (8)	6.5e-005 ± 1.5e-005	8.7 ± 0.9
HFHFD	Placebo (8)	7.7e-005 ± 2.1e-005	7.8 ± 0.7
	Lanifibranor (7)	6.3e-005 ± 9.3e-006	5.8 ± 0.9*

Table S10. ***In situ ex vivo*** **liver perfusion in early MASLD: % vascular relaxation with acetylcholine at i.e. 10^{-5} mol/L and 10^{-3} mol/L.**
Male Wistar Han rats of 8 weeks old were either fed a chow diet (CD) or a methionine-choline-deficient diet (MCDD) for 4 weeks and were simultaneously treated with either placebo, fenofibrate (30 mg/kg), GW501516 (10 mg/kg), rosiglitazone (5 mg/kg) or lanifibranor (100 mg/kg) daily, QD via oral gavage. n = 7-13/group). Data is presented as mean ± SEM. The THPG data were analysed using a generalised estimating equation model followed by least significant difference post hoc testing with *: p<0.05. M, molar. * for comparison with MCDD + placebo.

Acetylchlonine dose-response	Group (n)	Vascular relaxtion (%) at 10^{-5} M ± SEM	Vascular relaxtion (%) at 10^{-3} M ± SEM
CD	Placebo (10)	-26.0 ± 7.3	-61.4 ± 5.6*
	Fenofibrate (8)	-5.1 ± 5.4	-64.0 ± 15.3
	GW501516 (7)	-17.3 ± 3.4	-60.3 ± 5.6
	Rosiglitazone (7)	-21.3 ± 5.3	-63.9 ± 5.6
	Lanifibranor (6)	-15.8 ± 8.5	-72.9 ± 4.3
MCDD	Placebo (11)	-11.3 ± 5.4	-39.7 ± 4.5
	Fenofibrate (8)	-11.2 ± 5.9	-53.9 ± 6.9
	GW501516 (7)	-18.4 ± 8.6	-53.4 ± 10.2
	Rosiglitazone (7)	-15.7 ± 6.2	-58.3 ± 4.2*
	Lanifibranor (10)	-29.0 ± 6.7	-60.5 ± 6.1*

Table S11. *In situ ex vivo* liver perfusion in MASH: vascular relaxation with acetylcholine at doses of i.e. 10^{-5} mol/L and 10^{-3} mol/L.

8 weeks old male Zucker fatty rats fed a high-fat high-fructose (HFHFD) diet and 8 weeks old male Zucker lean rats fed a chow diet (CD) were preventively treated with either placebo or lanifibranor (100 mg/kg) daily QD via oral gavage during the complete period of 8 weeks of diet. n = 7-8/group. Data is presented as mean ± SEM. The THPG data were analysed using a generalised estimating equation model followed by least significant difference post hoc testing with *: $p<0.05$. M, molar. * for comparison with MCDD + placebo.

Acetylchlonine dose-response	Group (n)	Vascular relaxtion (%) at 10^{-5} mol/L	Vascular relaxtion (%) at 10^{-3} mol/L
CD	Placebo (8)	-14.6 ± 5.5*	-42.3 ± 6.5
	Lanifibranor (8)	-13.6 ± 3.1	-45.1 ± 5.0
HFHFD	Placebo (8)	-2.2 ± 2.5	-41.5 ± 4.3
	Lanifibranor (8)	-14.7 ± 1.3*	-40.7 ± 4.7

Supplementary references

[1] Kleiner DE, Brunt EM, Van Natta M, et al. Design and validation of a histological scoring system for nonalcoholic fatty liver disease. Hepatology 2005;41:1313-1321.
[2] **Haber MA, Iranmahboob A**, Thomas C, et al. ERG is a novel and reliable marker for endothelial cells in central nervous system tumors. Clin Neuropathol 2015;34:117-127.
[3] **Francque S, Laleman W**, Verbeke L, et al. Increased intrahepatic resistance in severe steatosis: endothelial dysfunction, vasoconstrictor overproduction and altered microvascular architecture. Lab Invest 2012;92:1428-1439.
[4] **Van der Graaff D, Kwanten WJ,** Couturier FJ, et al. Severe steatosis induces portal hypertension by systemic arterial hyporeactivity and hepatic vasoconstrictor hyperreactivity in rats. Lab Invest 2018;98:1263-1275.
[5] Boubia B, Poupardin O, Barth M, et al. Design, Synthesis, and Evaluation of a Novel Series of Indole Sulfonamide Peroxisome Proliferator Activated Receptor (PPAR) α/γ/δ Triple Activators: Discovery of Lanifibranor, a New Antifibrotic Clinical Candidate. Journal of Medicinal Chemistry 2018;61:2246-2265.
[6] Wettstein G, Luccarini JM, Poekes L, et al. The new-generation pan-peroxisome proliferator-activated receptor agonist IVA337 protects the liver from metabolic disorders and fibrosis. Hepatol Commun 2017;1:524-537.
[7] van der Graaff D, Chotkoe S, De Winter B, et al. Vasoconstrictor antagonism improves functional and structural vascular alterations and liver damage in rats with early NAFLD. JHEP Rep 2022;4:100412.
[8] European Association for Study of L, Asociacion Latinoamericana para el Estudio del H. EASL-ALEH Clinical Practice Guidelines: Non-invasive tests for evaluation of liver disease severity and prognosis. J Hepatol 2015;63:237-264.

Bold indicates co-first authors
