# Supplementary material for: Altered liver sinusoidal endothelial cells in MASLD and their evolution following lanifibranor treatment
Source: JHEP Rep. 2025 Feb 22;7(6):101366. doi: 10.1016/j.jhepr.2025.101366 (PMC12142333; doi:10.1016/j.jhepr.2025.101366)
Supplement: Multimedia component 2 [file mmc2.docx]

**JHEP Reports**

**CTAT methods**

Tables for a “Complete, Transparent, Accurate and Timely account” (CTAT) are now mandatory for all revised submissions. The aim is to enhance the reproducibility of methods.

- Only include the parts relevant to your study
- Refer to the CTAT in the main text as ‘Supplementary CTAT Table’
- Do not add subheadings
- Add as many rows as needed to include all information
- Only include one item per row

**If the CTAT form is not relevant to your study, please outline the reasons why:**

|  |
| --- |

- 1. **Antibodies**

| **Name** | **Citation** | **Supplier** | **Cat no.** | **Clone no.** |
| --- | --- | --- | --- | --- |
| Anti-CD34 |  | Abcam | ab8536 | QBEnd-10 |
| Anti-CD34 |  | Abcam | ab81289 | EP373Y |
| Anti-ERG |  | Abcam | ab92513 | EPR3864 |

- 1. **Cell lines**

| **Name** | **Citation** | **Supplier** | **Cat no.** | **Passage no.** | **Authentication test method** |
| --- | --- | --- | --- | --- | --- |
| **N/A** |  |  |  |  |  |

- 1. **Organisms**

| **Name** | **Citation** | **Supplier** | **Strain** | **Sex** | **Age** | **Overall n number** |
| --- | --- | --- | --- | --- | --- | --- |
| Rat |  | Charles River | Wistar Han | Male | 8 weeks old | 426 |
| Rat |  | Charles River | Zucker fatty rat | Male | 8 weeks old | 60 |
| Rat |  | Charles River | Zucker lean rat | Male | 8 weeks old | 60 |

- 1. **Sequence based reagents**

| **Name** | **Sequence** | **Supplier** |
| --- | --- | --- |
| **N/A** |  |  |

- 1. **Biological samples**

| **Description** | **Source** | **Identifier** |
| --- | --- | --- |
| **N/A** |  |  |

- 1. **Deposited data**

| **Name of repository** | **Identifier** | **Link** |
| --- | --- | --- |
| **N/A** |  |  |

- 1. **Software**

| **Software name** | **Manufacturer** | **Version** |
| --- | --- | --- |
| ImageJ | Bathesda | 2.14.0 |
| LabChart | AD Instruments | 7 |
| SPSS | IBM, SPSS Inc. | 29.0 |
| Prism | GraphPad | 9.2.0 |

- 1. **Other (*e.g*. drugs, proteins, vectors etc.)**

| **Name** | **Supplier** | **Cat no.** |
| --- | --- | --- |
| Placebo | Inventiva Pharma |  |
| Fenofibrate | Inventiva Pharma |  |
| GW501515 | Inventiva Pharma |  |
| Rosiglitazone | Inventiva Pharma |  |
| Lanifibranor | Inventiva Pharma |  |
| Ketalar | Pfizer |  |
| Rompun | Bayer |  |
| ET-1 | Sigma | 05-23-3800 |
| Acetylcholine | Sigma | A6625 |
| Methoxamine | Santa Cruz | sc-263468 |
| MCDD | Envigo RMS B.V. |  |
| HFHFD | Research Diets | D16042610 |
| Chow diet | ICN Biomedicals SA |  |

- 1. **Please provide the details of the corresponding methods author for the manuscript:**

| Preclinical part:  Shivani Chotkoe  Laboratory of Experimental Medicine and Paediatrics  University of Antwerp  e-mail: [shivani.chotkoe@uantwerpen.be](mailto:shivani.chotkoe@uantwerpen.be) |
| --- |

**2.0 Please confirm for randomised controlled trials all versions of the clinical protocol are included in the submission. These will be published online as supplementary information.**

| **N/A** |
| --- |
