# Supplementary material for: Altered liver sinusoidal endothelial cells in MASLD and their evolution following lanifibranor treatment
Source: JHEP Rep. 2025 Feb 22;7(6):101366. doi: 10.1016/j.jhepr.2025.101366 (PMC12142333; doi:10.1016/j.jhepr.2025.101366)
Supplement: Multimedia component 4 [file mmc4.pdf]

Altered liver sinusoidal endothelial cells in MASLD and their evolution following lanifibranor treatment

Pierre-Emmanuel Rautou[1,2,*,†], **Shivani Chotkoe**[3,4,†], Louise Biquard[1], Guillaume Wettstein[5], Denise van der Graaff[3,4], Yao Liu[6], Joris De Man[4], Christophe Casteleyn[7,8], Sofie Thys[9,10], Winnok H. De Vos[9,10,11], Pierre Bedossa[12], Michael P. Cooreman[5], Martine Baudin[5], Jean-Louis Abitbol[5], Philippe Huot-Marchand[5], Lucile Dzen[5], Miguel Albuquerque[12], Pierre Broqua[5], Jean-Louis Junien[5], Luisa Vonghia[3,4], Manal F. Abdelmalek[13], Wilhelmus J. Kwanten[3,4,‡], Valérie Paradis[1,14,‡], Sven M. Francque[3,4,*,‡]

JHEP Reports **2025**. vol. 7 | 1–15

Check for updates

Background & Aims: Data on changes in liver sinusoidal endothelial cells (LSECs) in patients with metabolic dysfunction-associated steatotic liver disease (MASLD) and their response to treatment are limited. This study aimed at determining (i) features associated with LSEC capillarisation in patients with MASLD; (ii) whether LSEC changes can regress with the pan-peroxisome proliferator-activated receptor (PPAR) agonist lanifibranor; (iii) the role of the different PPAR isotypes on LSEC changes in MASLD.

Methods: We analysed CD34 expression, a marker of LSEC capillarisation, on liver biopsies from patients considered for inclusion in the NATIVE trial at baseline (n = 249), and after 24 weeks of placebo or lanifibranor (n = 173). Two rat models of MASLD were used to investigate the effect of lanifibranor or of mono-PPAR agonists on LSECs.

Results: Lobular CD34 staining was more intense in patients with isolated steatosis than in those with no MASLD (52% *vs.* 10%; $p = 0.03$). In the overall cohort, this staining was more intense in patients with metabolic dysfunction-associated steatohepatitis (MASH) than in those without (63% *vs.* 41%; $p = 0.01$) and strongly correlated with liver fibrosis and to a lesser extent with liver inflammation. Lanifibranor treatment was associated with more common improvement in CD34 periportal staining ($p = 0.025$), and less frequent worsening of lobular staining ($p = 0.028$). Compared with healthy rats, rats with MASLD had higher CD34 staining, portal venous pressure, intrahepatic vascular resistance, and impaired liver endothelial function. Lanifibranor normalised or strongly improved these abnormalities, whereas mono-PPAR agonists caused partial improvements.

Conclusions: In patients, LSEC capillarisation was increased at the earliest stages of MASLD and was associated with liver fibrosis and inflammation. In both patients and rats with MASLD, lanifibranor treatment was associated with improvement in liver endothelial phenotype.

© 2025 The Authors. Published by Elsevier B.V. on behalf of European Association for the Study of the Liver (EASL). This is an open access article under the CC BY license (http://creativecommons.org/licenses/by/4.0/).

Introduction

Metabolic dysfunction-associated steatotic liver disease (MASLD), formerly known as non-alcoholic fatty liver disease (NAFLD), is defined as the presence of steatosis (*i.e.* abnormal hepatic triglyceride accumulation) in >5% of hepatocytes according to histological analysis, in the presence of cardiometabolic risk factors.[1] MASLD encompasses a spectrum of different conditions, including isolated steatosis (metabolic dysfunction-associated steatotic liver [MASL]) and metabolic dysfunction-associated steatohepatitis (MASH).[2,3] Contrary to MASL, MASH can progress to cirrhosis and hepatocellular carcinoma. It is estimated that 25% of the adult population worldwide has MASLD, increasing concomitantly with the global obesity epidemic.[4]

The current view of MASH pathogenesis focuses on hepatocytic alterations with metabolic and lipotoxic stresses leading to cell death and the onset of liver inflammation.[5–8] However, an increased intrahepatic vascular resistance (IHVR) has repetitively been documented, both in humans[9–12] and in preclinical models,[13–19] as an early event in MASLD, significantly contributing to the progression of the disease through impaired intrahepatic blood flow and subsequent hepatic hypoxia.[20–22] Liver sinusoidal endothelial cells (LSECs) play a central role in the vascular aspects of liver diseases, including in MASH. In chronic liver diseases, capillarisation, that is loss of fenestrae and development of a basal membrane, is an important feature because it is associated with the loss of the antifibrotic and the anti-inflammatory properties of LSECs.[13,23]

* Corresponding authors. Addresses: Service d'Hépatologie, Hôpital Beaujon, 100 Boulevard du General Leclerc, 92100 Clichy, France. Tel.: +331 40 87 52 83; fax +331 40 87 44 35 (P-E. Rautou); Department of Gastroenterology and Hepatology, University Hospital Antwerp, Drie Eikenstraat 655, 2650 Edegem, Belgium. Tel.: +32 3 821 44 75 (S. Francque).
E-mail addresses: pierre-emmanuel.rautou@inserm.fr (P.-E. Rautou), sven.francque@uza.be (S.M. Francque).
† Shared first authors.
‡ Shared senior authors.
https://doi.org/10.1016/j.jhepr.2025.101366

In MASLD, animal data suggested that LSECs undergo capillarisation already at the stage of MASL, before MASH onset.[24] A small human study did not confirm this finding in patients.[25] This study included only 29 patients and did not consider the potential spatial distribution of capillarisation (which might coincide with the perisinusoidal fibrosis, typically found in the lobular area in MASH).[25–27]

Despite its prevalence and potential severity, therapeutic options for MASH are currently limited. A thyroid hormone receptor beta-selective agonist has recently been approved,[28] and several other candidate molecules are being investigated in phase II and III trials.[29] Among those, peroxisome proliferator-activated receptor (PPAR) agonists have sparked major interest as therapeutic agents for MASH, given the pleiotropic roles of the three PPAR isotypes (α, β/δ, γ) in the regulation of energy metabolism, fibrosis, and inflammation, but also their role in endothelial cells and their impact on IHVR in models of portal hypertension.[30–32] However, the effect of PPAR agonists on the hepatic vasculature in early MASLD and MASH has not been investigated.

In the present study we analysed liver biopsies from 249 patients with a suspicion of MASH screened for inclusion in the NATIVE clinical trial,[31] aiming at identifying clinical and histological features associated with LSECs capillarisation and its spatial distribution in MASLD, and at assessing whether LSEC changes in patients can regress following treatment with the pan-PPAR agonist lanifibranor. We further investigated the role of the different PPAR isotypes (and their therapeutic potential) on the structural and functional aspects of the altered hepatic vascular biology, including LSEC changes, in two animal models of early MASLD, with the aim to understand the role of these vascular changes in disease progression. We also examined the effects of the combined pan-PPAR approach, and its potential benefit compared to mono-PPAR agonists.

Materials and methods

Clinical approaches

Clinical study design and patients

The clinical section of the present work is an ancillary study of the NATIVE study (funded by Inventiva Pharma; NATIVE ClinicalTrials.gov number, NCT03008070), a phase IIb, double-blind, randomised, placebo-controlled trial evaluating the efficacy and safety of lanifibranor in patients with biopsy-proven non-cirrhotic MASH with severe disease activity.[33,34] NATIVE was approved by independent ethical committees and appropriate authorities in all 16 countries where at least one patient underwent randomisation and complies with the declaration of Helsinki (Table S1).[33] All patients gave written informed consent.

Between February 2018 and July 2019, a total of 868 adult patients presenting with a suspicion of MASH were screened. They underwent a liver biopsy if none was obtained in the preceding 6 months. These biopsies will be designated hereafter as obtained at baseline. Classification of the liver lesions on histology (no MASL, MASL, MASH) was performed according to the Steatosis-Activity-Fibrosis (SAF) scoring system and algorithm described by Bedossa *et al.*[26] Patients meeting the study eligibility criteria for whom the liver biopsy confirmed the presence of MASH without cirrhosis, with a SAF activity score ≥3 and SAF steatosis score ≥1, were included in the NATIVE trial (n = 247) and randomised in a 1:1:1 ratio to receive placebo, 800 mg, or 1,200 mg lanifibranor orally once daily for 24 weeks.[31,33] At the end of the treatment period, patients underwent another liver biopsy. These patients are hereafter referred to as ‘randomised patients’. The 621 patients who did not meet these criteria are referred to as ‘screening failures’, including 297 patients with a liver biopsy of sufficient quality but with non-inclusion criteria; 207 of these 297 patients had available unstained liver slides. Among them, we selected 76 patients to obtain a balanced distribution of mild, moderate, and severe steatosis, MASH activity and fibrosis, as detailed in Table S2.

Histological scoring of density of CD34 positive vessels

Analysis of CD34 staining was performed by two approaches: (i) a semiquantitative approach based on a three-tier grading system according to the extent of the sinusoidal CD34 positivity, performed by an expert pathologist (VP) unaware of the patients groups, separating periportal and lobular zones, with lobular score of 1 indicating CD34 positivity restricted to the centrolobular area and lobular score of 2 extending to mediolobular and with periportal score of 1 indicating CD34 positivity restricted to the periportal area and periportal score of 2 extending to mediolobular; and (ii) an automatically quantitative approach, defined as the number of vessels per unit of area (μm^2) (Fig. S1). Co-staining with erythroblast transformation-specific related gene (ERG)[35] confirmed endothelial localisation of the CD34 staining (Fig. S1).[35] More details are provided in the Supplementary **Materials and methods.**

Preclinical approaches

Animal models

We used two rat models to represent two stages of the disease. Early MASLD was modelled by feeding male Wistar Han rats with a methionine-choline-deficient diet (MCDD) for 4 weeks, to induce severe steatosis in the absence of MASH.[16,17] To study the intrahepatic effects of the different PPAR isotypes, rats (n = 6–8/group) underwent gavage once a day (QD) with either placebo (1% methylcellulose + 0.05% poloxamer), fenofibrate (PPAR-α agonist, 30 mg/kg), GW501516 (PPAR-β/δ agonist, 10 mg/kg), rosiglitazone (PPAR-γ agonist, 5 mg/kg), or lanifibranor[36] (balanced pan-PPAR agonist, not directly affecting other pathways than PPARs; 100 mg/kg), during the entire 4 weeks of diet as a preventive treatment (Fig. S2A). The doses of the mono-PPAR agonists were chosen because their potency and efficacy for their respective nuclear receptors were similar to that of lanifibranor for the same respective nuclear receptor.

To exclude model specificity and to examine the effects at the stage of steatohepatitis rather than isolated steatosis, the most important results of lanifibranor were tested in a second model considered a more clinically relevant representation, that is male Zucker fatty rats (ZFR, fa/fa) were fed a high-fat high-fructose diet (HFHFD) (Fig. S2B), and compared with Zucker

lean rats (ZLR) fed a chow diet (CD). More details are provided in the Supplementary **Materials and methods.**

Histology, CD34 immunohistochemistry, in vivo *haemodynamics,* in situ ex vivo *liver perfusion, and vascular corrosion casting*

Details are provided in the Supplementary **Materials and methods**.

Statistical analyses

Details are provided in the Supplementary **Materials and methods**.

Results

Patients

Between February 2018 and July 2019, 868 patients with a suspicion of MASH were screened and 247 patients were randomised in the NATIVE study. Among the latter, 173 patients had baseline liver tissue available for CD34 staining, including 163 with liver tissue also available at week 24. These 173 patients were selected for the present study. Among the 297 patients not randomised in NATIVE study, but with available liver tissue remaining from the screening period, 76 were selected for the present study (Fig. S3). These 173 and 76 patients will be designated hereafter as the whole baseline cohort (N = 249). The main characteristics of all patients at baseline are summarised in Table S3.

LSEC CD34 staining according to the presence of MASL or MASH

Immunohistological staining for CD34 was performed as illustrated in Fig. 1A and B. We observed that the manual semi-quantitative assessments of CD34 staining performed by the expert pathologist were strongly correlated with the automatic quantification of the density of CD34-positive vessels, attesting the consistency of these evaluations (Fig. S4).

Of the 249 patients from the whole baseline cohort, 209 had MASH (40 had no MASH and were obviously recruited out of the NATIVE screening failures). When comparing liver CD34

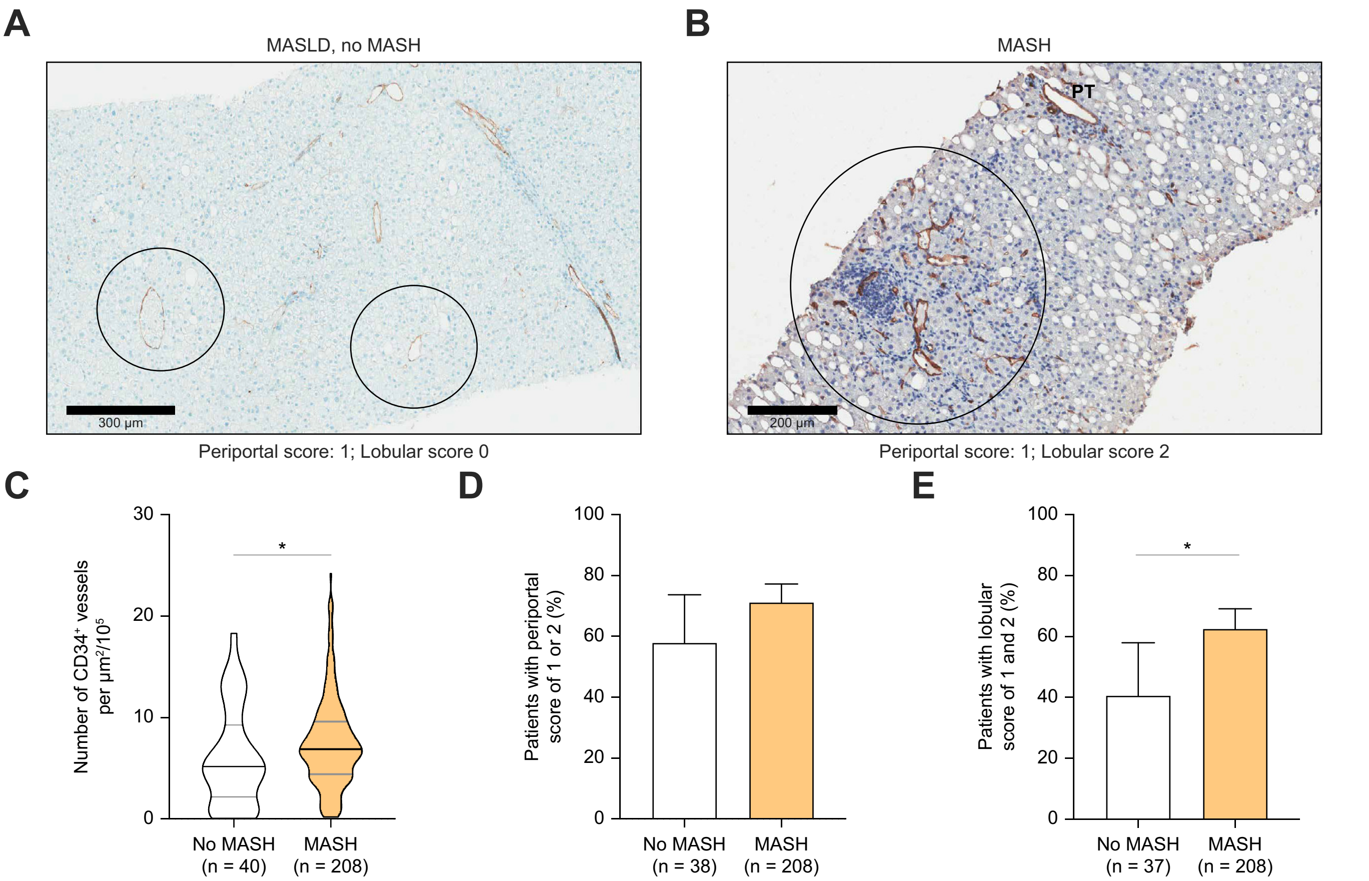

Fig. 1. CD34 staining is more pronounced in patients with MASH than in patients without MASH (classified according to the SAF algorithm[26]), particularly in the lobular area. Density of CD34-positive vessels, periportal score, and lobular score were available in 248, 246 and 245 patients, respectively, as detailed in Fig. S3. (A,B) Representative images of CD34 staining with corresponding periportal and lobular score of patients with no MASH and MASH, respectively. (C) Density of CD34-positive vessels is displayed for patients without MASH and with MASH. Percentage of patients with, respectively, periportal (D) and lobular (E) score of 1 or 2 is displayed. Lobular areas are circled. For violin plots, the bars represent the median ± IQR, otherwise bars represent 95% CIs. *p <0.05. The Wilcoxon-Mann-Whitney U test, the X^2 test, or Fisher test was used when appropriate. Patients' numbers vary between graphs because vessel density, periportal score, and lobular score were unavailable for technical and staining quality reasons for, respectively, one, three, and four patients out of 249. MASH, metabolic dysfunction-associated steatohepatitis; PT, portal tracts; SAF, Steatosis-Activity-Fibrosis score.

staining in patients without MASH and with MASH, we observed that patients with MASH had a higher density of CD34 positive vessels (Fig. 1C, p <0.05) and a higher rate of CD34 staining in the lobular area (Fig. 1E, p <0.05), but not in the periportal area (Fig. 1D). Interestingly, when focusing on the no-MASH group (n = 40), we observed that lobular CD34 staining was more common in patients with MASL than in those with normal histology (Fig. 2A–E). There was no association in patients without MASH between CD34 staining and liver inflammation (Fig. 2F–H).

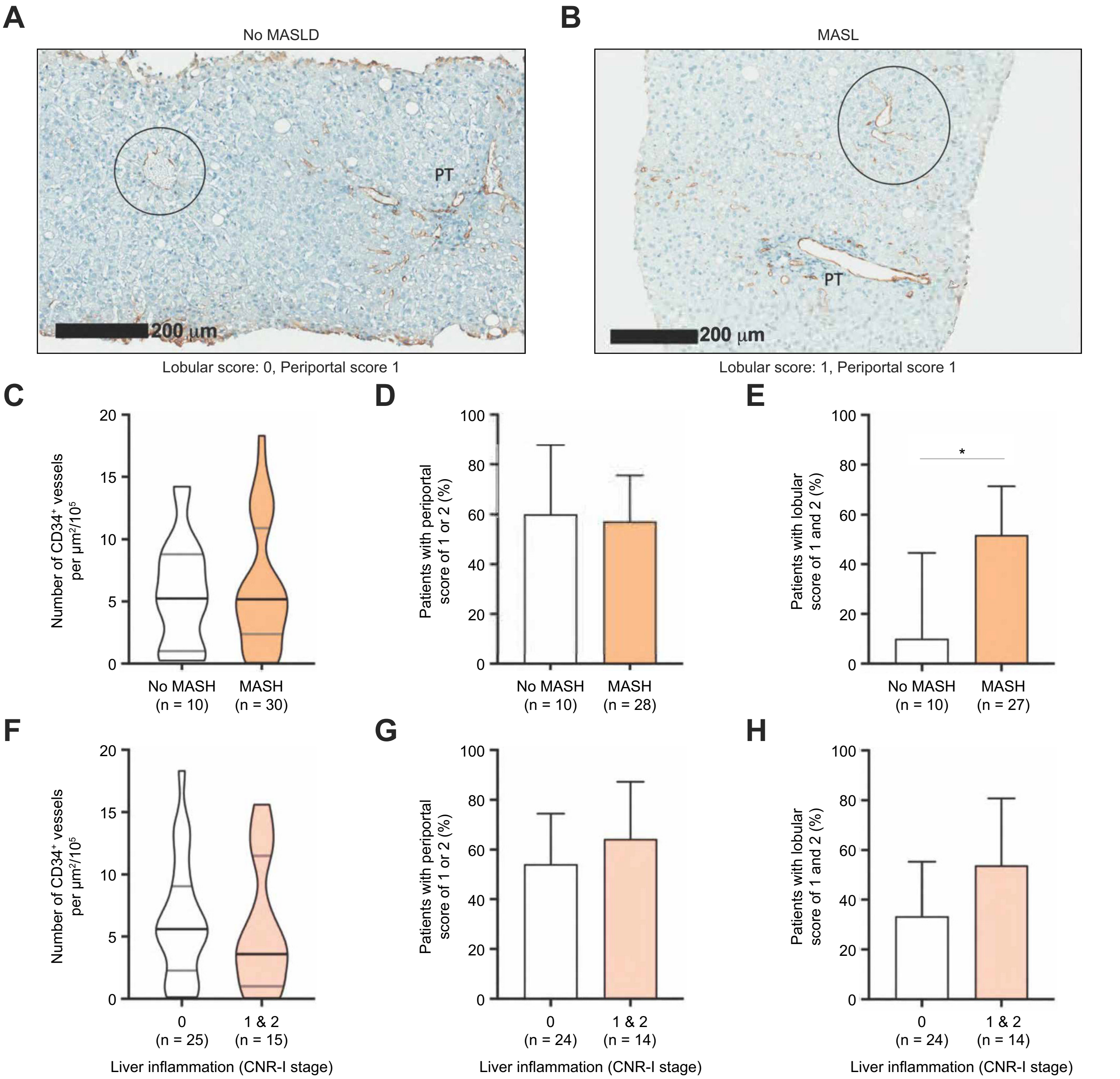

Fig. 2. Comparison of CD34 staining between patients with isolated steatosis (MASL, but no MASH) and without MASLD (according to the SAF algorithm[26]). Density of CD34-positive vessels, periportal score, and lobular score were available in 40, 38, and 37 patients without MASH, respectively, as detailed in Fig. S3. (A,B) Representative images of CD34 staining with corresponding periportal and lobular score of patients with no MASLD and MASL, respectively. (C) Density of CD34 positive vessels is displayed for patients without MASLD and with MASL. Percentage of patients with, respectively, periportal (D) and lobular (E) score of 1 or 2 is displayed. Density of CD34-positive vessels (F), periportal score (G), and lobular score (H) is displayed according to the level of liver inflammation (CRN-I stages). The Wilcoxon-Mann-Whitney U test was used. Lobular areas are circled. For violin plots, the bars represent the median ± IQR, otherwise bars represent 95% CIs. Patients' numbers vary between graphs because vessel density, periportal score, and lobular score were unavailable for technical and staining quality reasons for, respectively, one, three, and four patients out of 249. *p <0.05. CRN, Clinical Research Network; MASL, metabolic dysfunction-associated steatotic liver; MASLD, metabolic dysfunction-associated steatotic liver disease; MASH, metabolic dysfunction-associated steatohepatitis; PT, portal tracts; SAF, Steatosis-Activity-Fibrosis score.

LSEC CD34 expression, localisation, and liver histology in MASLD

In the whole baseline cohort, the density of CD34-positive vessels was strongly linked with liver fibrosis (*p* <0.001) (Fig. 3A) and liver inflammation (*p* = 0.027) using the NASH Clinical Research Network (CRN) scoring system[37] instead of the SAF-score,[26] because of a more granular score for inflammation ranging from 0 to 3 (Fig. 3B). Periportal staining and lobular staining were also associated with liver fibrosis and liver inflammation, although the association was more pronounced for lobular staining (Fig. 3C–F). No association was found between CD34 staining and steatosis or ballooning (Fig. S5).

LSEC CD34 expression and clinical and laboratory features

We then aimed at identifying what clinical and laboratory features are linked to LSEC CD34 staining. We found statistically significant positive correlations between the density of CD34-positive vessels and serum aspartate aminotransferase (AST), serum γ-glutamyltransferase, Fibrosis-4 (FIB-4) score[38] and liver stiffness measurement using vibration-controlled transient elastography (FibroScan®, Echosens, Paris, France), but with Spearman correlation coefficients <0.212, indicating only weak correlations (Table S4). There was no association between periportal CD34 staining and clinical or laboratory features (Table S4), whereas patients with lobular CD34 staining had higher serum AST (Fig. 4A), serum alanine aminotransferase (ALT) (Fig. 4B), cytokeratin 18 M65 (a marker of cell death) (Fig. 4C) and a trend towards higher FIB-4 (Fig. 4D) than patients without lobular CD34 staining.

Effect of lanifibranor on LSEC capillarisation

In patients with MASH randomised into the NATIVE trial, 24-week treatment with lanifibranor at both dosages had no effect on the density of CD34-positive vessels (Fig. 5A). Improvement in periportal score was more common in patients treated with lanifibranor than in those treated with placebo, with a dose–response effect (placebo: 7.5%. lanifibranor 800 mg: 18.5%. lanifibranor 1,200 mg: 23.2%. *p* = 0.025, Fig. 5C). Patients with lanifibranor treatment had less worsening of lobular score (placebo: 39.6%, lanifibranor 800 mg: 18.5%, lanifibranor 1,200 mg: 23.2%; *p* = 0.028, Fig. 5F).

Animal models

Early MASLD

After 4 weeks of MCDD, animals developed marked hepatomegaly (liver/total body weight [TBW] ratio): MCDD 4.6 (4.4–5.0) *vs*. CD 3.0 (2.9–3.2), *p* <0.001; Table 1; Fig. S6, Table S5). At histology, placebo-treated rats fed an MCDD had severe grade 3 steatosis with no microscopic indications of liver inflammation or ballooning (Fig. 6A), nor fibrosis (Fig. S7A). Likewise, rats fed an MCDD had no liver collagen-1 protein expression (Fig. S7B). Fenofibrate induced full inhibition of steatosis in MCDD rats (Fig. S8) despite a significant increase in liver volume (Fig. S6). GW501516 reduced steatosis area by 8.3% (absolute reduction compared with 43.7% in MCDD with placebo), *p* <0.01. Rosiglitazone resulted in a slight reduction of 3.7% (Fig. S8). Lanifibranor improved steatosis in MCDD rats compared to the placebo group, mainly in the centrilobular zones, with a reduction in steatosis area of 15.4%, *p* <0.0001 (Fig. 6B).

MASH

After 8 weeks of diet and treatment (Fig. S2B), in placebo groups, both TBW and liver weight were significantly increased in HFHFD-fed ZFRs compared with lean controls (Table 2). Histology demonstrated that ZLRs on a CD had normal liver histology without any steatosis, ballooning, inflammation, or fibrosis (Figs S9 and S10), whereas in ZFRs borderline MASH was present (NAFLD Activity Score [NAS] = 0.0 (0.0–0.25) in ZRLs *vs.* 3.0 (3.0–4.3) in ZFRs, *p* <0.001). Out of all animals, one had definite MASH (NAS score = 5), whereas five had borderline MASH (NAS = 3–4). The animals with MASH had mild steatosis (7.0 ± 1.5% *vs.* 0.04 ± 0.0% in ZLRs, *p* <0.001, Fig. S9B), ballooning (Fig. S9A) and inflammation as well, without the development of fibrosis (Fig. S10A). Likewise, rats with MASH had no liver collagen-1 protein expression (Fig. S10B). In HFHFD-fed ZFRs, lanifibranor even further increased TBW and decreased liver/TBW ratio. However, there was no difference in liver weight compared with placebo-treated HFHFD-fed ZFRs (Table 2; Fig. S11). At histology, lanifibranor tended to decrease the NAS score (from 3.0 [3.0–4.3] to 2.0 [1.8 – 3.3]), however not significantly. Steatosis decreased from 7.0 ± 1.5% to 2.3 ± 0.5% (Fig. S9B, *p* <0.004), mainly in the lobular area, while in the periportal area microvesicular steatosis was still present together with macrovesicular steatosis and a tendency of little to no ballooning (from 1.0 [1.0–1.5] to 0.0 [0.0–1.0]) (Fig. S9A). HFHFD-fed ZFRs treated with placebo had a significantly increased spleen weight compared with ZLRs. Treatment with lanifibranor caused minimal insignificant decrease of the spleen weight in ZFRs (Table 2; Fig. S11D).

LSEC CD34 staining

Capillarisation in early MASLD

We then assessed liver CD34 staining. In placebo-treated groups, MCDD-fed rats presented twice more CD34 staining than control rats (Fig. 6A and C). In MCDD-fed rats, fenofibrate, GW501516, and lanifibranor almost normalised CD34 staining, whereas treatment with rosiglitazone had minimal effect (Fig. S12). Of note, treatment with all mono-agonists and lanifibranor showed a trend towards decreased CD34 staining in CD-fed rats, however, with no statistical significance (Fig. 6; Fig. S12, Table S6).

Capillarisation in MASH

In placebo-treated animals, there was a non-significant trend towards increased CD34 staining in MASH livers compared to control livers. Lanifibranor, also insignificantly, tended to lower

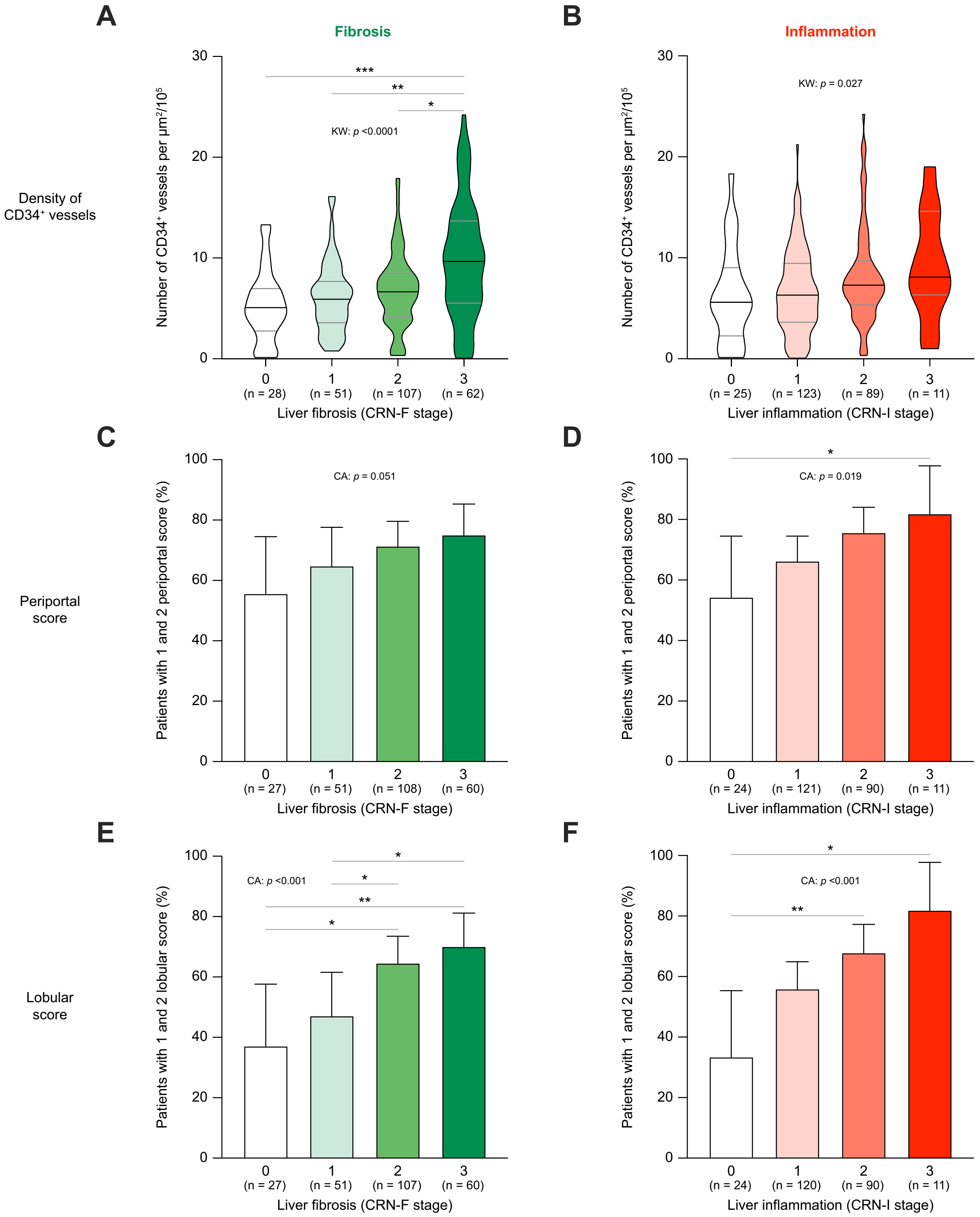

Fig. 3. Relationship between CD34 staining level and localisation and histological features of MASLD in 249 patients with a suspicion of MASH. Density of CD34-positive vessels, periportal score, and lobular score were available in 248, 246, and 245 patients, respectively, as detailed in Fig. S3. Baseline density of CD34-positive vessels is displayed according to fibrosis (CRN-F grade) (A) and according to inflammation (CRN-I grade) (B). Percentage of patients with periportal score for CD34 staining of 1 or 2 is displayed according to fibrosis (CRN-F grade) (C) and according to inflammation (CRN-I grade) (D). Percentage of patients with lobular score for CD34 staining of 1 or 2 is displayed according to fibrosis (CRN-F grade) (E) and according to inflammation (CRN-I grade) (F). When appropriate, Kruskal–Wallis, and *post hoc* Dunn's tests were performed between all columns, with *p <0.05; **p <0.01; ***p <0.001. For violin plots, the bars represent the median ± IQR, otherwise bars

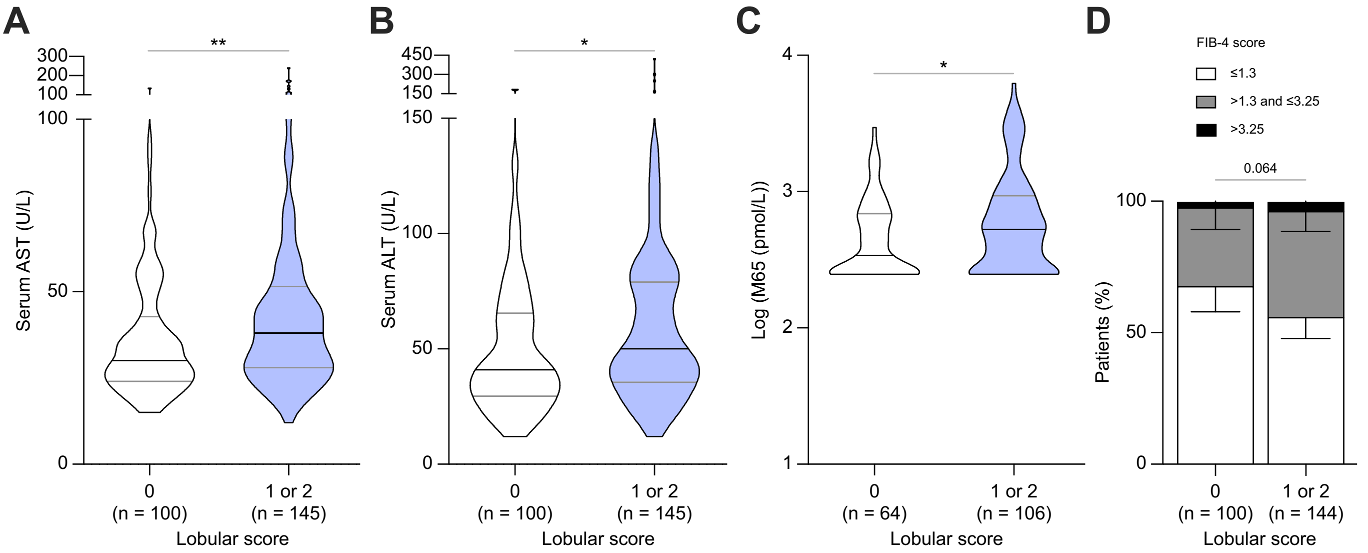

Fig. 4. Association of CD34 staining in the lobular area with clinical features. CD34 lobular score was available 245 patients, as detailed in Fig. S3. On the analysis population (screening failure and randomised patients), serum AST (A), serum ALT (B), cytokeratin 18 M65 fragments (C) and FIB-4 score (D) were significantly increased in patients with CD34 lobular staining score of 1 or 2. The Wilcoxon-Mann-Whitney *U* test, the X^2 test, or Fisher test was used when appropriate. For violin plots, the bars represent the median ± IQR, otherwise bars represent 95% CIs. **p* <0.05; ***p* <0.01. AST, aspartate aminotransferase; ALT, alanine aminotransferase; FIB-4, Fibrosis-4.

the CD34 staining in control livers as well as in MASH livers (Table 2; Fig. S9A and B).

In vivo haemodynamics and pressures

Early MASLD

In placebo-treated animals, MCDD-fed rats had a significantly higher portal venous pressure (measured as described in the Supplementary **Materials and methods)** compared with CD rats. The values were 5.6 (5.1–6.4) and 3.5 (3.2–3.9) mmHg, respectively, *p* <0.0001 (Fig. 7A; Table 1). In CD rats, none of the drugs induced a change in portal vein pressure (PVP) measurements. In MCDD-fed rats, all mono-PPAR agonists tended to decrease PVP, however, only fenofibrate caused a significant decrease (Fig. S13). Lanifibranor had a more pronounced effect (*p* <0.0001) compared with the mono-agonists and completely normalised the PVP in MCDD rats. Besides the impact on PVP, lanifibranor and also GW501516 to a lesser extent, decreased the mean arterial blood pressure in MCDD rats (Fig. 7B and S14A), while fenofibrate and rosiglitazone did not (Table 1; Fig. S14A, Table S5). There was no difference in pulse rate (Fig. S14D) nor in caudal cava vein pressure (CCVP) between the groups (data not shown).

MASH

In line with our findings in the MCDD model of steatosis, in ZFR after 8 weeks of HFHFD, the PVP was significantly increased compared to controls (MASH 7.1 ± 0.2 mmHg *vs.* controls 4.9 ± 0.2 mmHg, *p* <0.0001, Fig. 7C, Table 2). Lanifibranor caused a significant decrease of PVP in ZFR from 7.1 ± 0.2 mmHg to 5.2 ± 0.2 mmHg, *p* < 0.001. Besides the impact on PVP, the mean arterial pressure, that was elevated as well, decreased with lanifibranor treatment (Fig. 7D). The systolic and diastolic pressures were both decreased by lanifibranor (Fig. S15A and B). No differences in pulse rate (Fig. S15D) and CCVP were observed between the groups (data not shown).

In situ ex vivo liver perfusion: assessment of the intrahepatic vascular resistance

Early MASLD

In line with the *in vivo* data, the transhepatic pressure gradient (THPG) in MCDD-fed rats was significantly elevated at every perfusion flow velocity compared with CD-fed rats in placebo-treated groups (Fig. 7E; Fig. S16A). Fenofibrate significantly improved the THPG to normal values in MCDD rats at low perfusion flows, but this effect decreased at higher flows (Fig. S16B). Both GW501516 and rosiglitazone did not significantly improve the THPG in MCDD rats (Fig. S16C and D). Treatment with lanifibranor induced no changes in the THPG measurements in control rats, but it normalised the values at all flow rates in MCDD rats (Fig. 7E). Data points at 10 and 30 ml/min can be found in Table 1 and Table S5.

MASH

The THPG in placebo-treated ZFR was significantly increased at all flows compared with controls, and ZFR treated with lanifibranor during 8 weeks of HFHFD demonstrated

represent 95% CIs. Patients' numbers vary between graphs because vessel density, periportal score, and lobular score were unavailable for technical and staining quality reasons for, respectively, one, three, and four patients out of 249. CA, Cochran–Armitage; CRN, Clinical Research Network; KW, Kruskal–Wallis; MASL, metabolic dysfunction-associated steatotic liver; MASLD, metabolic dysfunction-associated steatotic liver disease; MASH, metabolic dysfunction-associated steatohepatitis; PT, portal tracts; SAF, Steatosis-Activity-Fibrosis score.

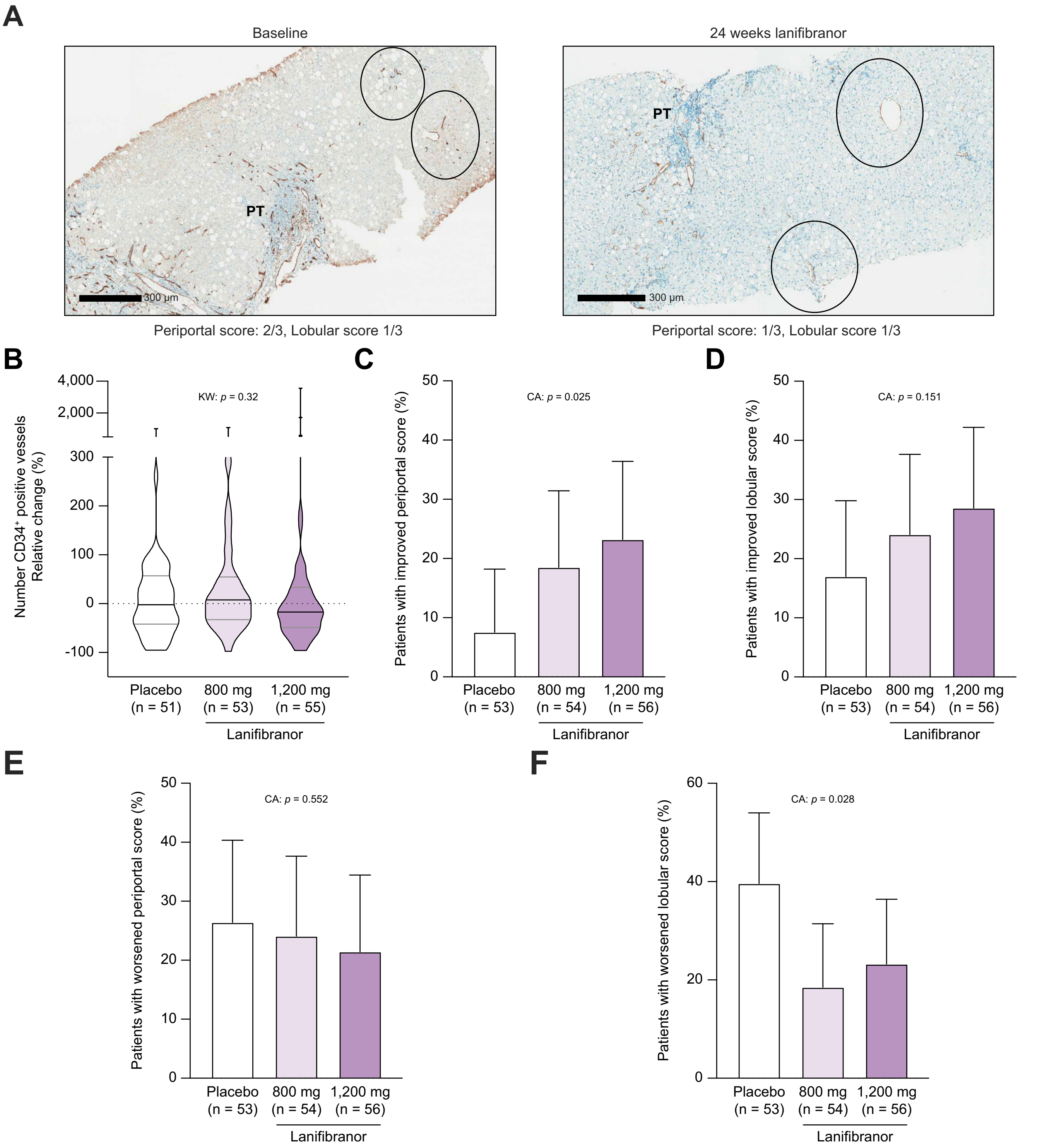

Fig. 5. Effect of lanifibranor treatment on CD34 staining. (A) Representative images of CD34 staining of a patient treated with 1,200 mg lanifibranor at baseline (left) and after 24 weeks of treatment (right). (B) Relative change of density of CD34 positive vessels according to treatment group in randomised patients. (C,D) Percentage of patients with improved (decreased by minimum 1 stage) periportal score (C) and lobular score (D) at week 24. (E,F) Percentage of patients with worsened (increased by minimum 1 stage) periportal score (E) and lobular score (F) at week 24. For violin plots, the bars represent the median ± IQR, otherwise bars represent 95% CIs. CA, Cochran–Armitage; KW, Kruskal–Wallis.

Table 1. Baseline characteristics, haemodynamics and pressures in an early MASLD rat model.

		Chow diet		MCDD	
**Parameter**	**Groups (n)**	**Placebo**	**Lanifibranor**	**Placebo**	**Lanifibranor**
**Demographic characteristics**					
Age (weeks)	58/38/64/38	8	8	8	8
Body weight (g): baseline	50/38/50/38	251.0 (242.8; 263.3)	249.5 (243.0; 257.0)	251.0 (242.8; 263.3)	250.0 (243.5; 258.0)
Body weight (g): after 4 weeks	58/38/62/38	349.0 (328.3; 360.3)*	338.5 (326.3; 373.0)‡	210.0 (202.0; 219.3)	211.0 (203.8; 215.0)
Δ Weight (g)	50/38/50/38	92.5 (72.0; 104.5)*	87.0 (69.3; 103.3)‡	-43.5 (-50.0; -38.75)	-43.5 (-46.0; -35.0)
Liver weight (g)	54/38/60/37	10.5 (9.3; 11.3)	10.4 (9.6; 11.1)	10.5 (9.3; 11.3)	9.7 (9.2; 11.1)
% liver/total body weight	56/38/60/37	3.0 (2.9; 3.2)*	3.0 (2.9; 3.2)‡	4.6 (4.4; 5.1)	4.7 (4.4; 5.1)
**Haemodynamics and pressures**					
MABP (mmHg)	43/31/40/37	123.7 (106.3; 130.8)	110.7 (101.1; 122.1)‡	121.9 (115.3; 132.3)	92.7 (78.1; 104.1)*
In vivo PVP (mmHg)	46/31/40/37	3.5 (3.2; 3.9)*	3.3 (3.1; 3.7)	5.6 (5.1; 6.4)	3.7 (3.2; 4.0)*
Portal blood flow (ml/min)	38/28/34/34	12.5 (11.0; 15.1)*	13.8 (12.1; 15.1)‡	10.1 (9.2; 11.3)	10.8 (9.2; 12.7)
THPG (mmHg) at 10 ml/min	8/8/7/8	3.7 ± 0.1*	3.5 ± 0.1	4.8 ± 0.2	3.5 ± 0.1*
THPG (mmHg) at 30 ml/min	8/8/7/8	6.6 ± 0.2*	7.4 ± 0.4	8.3 ± 0.4	6.3 ± 0.3*
**Histological parameters; H-E, PSR**					
Steatosis grade§	6/6/6/6	0 (0; 0)*	0 (0; 0)‡	3 (3; 3)	3 (3; 3)
Lobular inflammation grade¶		0 (0; 0)	0 (0; 0)	0 (0; 0)	0 (0; 0)
Ballooning grade**		0 (0; 0)	0 (0; 0)	0 (0; 0)	0 (0; 0)
Fibrosis stage††		0 (0; 0)	0 (0; 0)	0 (0; 0)	0 (0; 0)
NAS		0 (0; 0)*	0 (0; 0)‡	3 (3; 3)	3 (3; 3)
**Histological parameters; CD34 staining**					
CD34 quantification	6/6/6/6	20.9 ± 2.8*	14.3 ± 2.5	38.8 ± 2.5	21.4 ± 5.7*

Male Wistar Han rats of 8 weeks old (n = 6–8/group) were either fed a chow diet (CD) or a methionine-choline-deficient diet (MCDD) for 4 weeks and simultaneously treated with either placebo or lanifibranor (100 mg/kg) daily QD via oral gavage. Pooled data were analysed using the Kruskal–Wallis test followed by the Dunn test and presented as median (IQR). The THPG data were analysed using a generalised estimating equation model followed by least significant difference *post hoc* testing when appropriate. *For comparison with MCDD + placebo; †CD + lanifibranor *vs.* CD + placebo; ‡CD + lanifibranor *vs.* MCDD + lanifibranor; *,†,‡p <0.05. §Steatosis was assessed as the percentage of hepatocytes containing large and medium-sized intracytoplasmic lipid droplets and graded as 0 (<5%), 1 (5–33%), 2 (34–66%), or 3 (≥67%), according to the non-alcoholic steatohepatitis Clinical Research Network (NASH CRN) grading system. ¶Lobular inflammation was classified as grade 0 (no foci), grade 1 (<2 foci per 200 × field) or grade 2 (2–4 foci per 200 × field), according to the NASH CRN scoring system. **Ballooning was classified as grade 0 (no balloon hepatocyte) grade 1 (few but definite ballooned hepatocytes) or grade 2 (prominent ballooning), according to the NASH CRN grading system. ††Fibrosis was classified as stage F0 (no fibrosis), stage F1 (mild fibrosis), stage F2 (significant fibrosis), stage F3 (advanced fibrosis), or stage F4 (cirrhosis), according to the SAF–NASH CRN staging system. MABP, mean arterial blood pressure; NAS, NAFLD Activity Score; PVP, portal venous pressure; SAF, Steatosis-Activity-Fibrosis score; THPG, transhepatic pressure gradient.

normalisation of the increased THPG (Fig. 7F). Data points at 20 and 30 ml/min can be found in Table 2.

Dose–response experiments; hepatic vascular reactivity

Endothelin pathway

Endothelin-1 (ET-1) showed a dose-dependent increase of the THPG both in placebo-treated CD-fed animals and MCDD-fed animals, with a significantly increased responsiveness to ET-1 in MCDD compared with CD animals (Fig. S17, Table S7). Although fenofibrate increased ET-1 responsiveness, GW501516 and rosiglitazone considerably decreased ET-1 response, but this effect diminishes at the higher dose (Fig. S17B–D). The hyperreactivity to ET-1 was barely decreased with lanifibranor treatment (Fig. S17E). As lanifibranor did not relevantly alter ET-1 reactivity, experiments were not validated in HFHFD-fed ZFR rats.

Alpha-1 adrenergic pathway (methoxamine)

Early MASLD. In line with previous data,[17] MCDD-fed animals showed a significantly increased vascular reactivity (Emax) to methoxamine compared to CD, but with no difference in sensitivity (EC_{50}) to the drug (Fig. 7G; Table S8). All three mono-agonists (Fig. S18B–D) as well as lanifibranor decreased methoxamine hyperreactivity in MCDD animals close to control values (Fig. 7G).
MASH. In contrast to our findings in the MCDD model of steatosis, the HFHFD-fed ZFR were not hyperresponsive to methoxamine. However, lanifibranor significantly decreased Mx responsiveness below control values in HFHFD-fed ZFR without affecting the responses in CD rats (Fig. 7H; Table S9).

Muscarinergic pathway (acetylcholine)

Early MASLD. After pre-constriction with 3×10^{-5} mol/L methoxamine, the vasodilatory response to acetylcholine (ACh) was overall blunted in a dose-dependent manner in MCDD rats compared with control rats in placebo-treated groups (Fig. 7I; Table S10). Both fenofibrate and GW501516 did not improve impaired responsiveness to acetylcholine, while rosiglitazone improved acetylcholine hyporeactivity only at the highest doses (Fig. S19B–D). Lanifibranor improved the reactivity of ACh to control values in MCDD rats (Fig. 7I).
MASH. In HFHFD-fed ZFR, after pre-constriction with 1.5×10^{-4} mol/L methoxamine, the vasodilatory response to ACh was blunted at lower doses. Treatment with lanifibranor normalised acetylcholine hyporeactivity in HFHFD-fed ZFR (Fig. 7J; Table S11).

Vascular corrosion casting; 3D structure of liver sinusoids

In the placebo-treated CD group (Fig. S20A), examination of liver vascular corrosion casts using scanning electron microscopy unveiled a regular arrangement of sinusoids within lobules, characterised by small sinusoids with even diameters. In contrast, the MCDD-fed group exhibited a disruption of this regular sinusoidal pattern, resulting in a disarrayed network of vessels. Within this irregular arrangement, the sinusoids

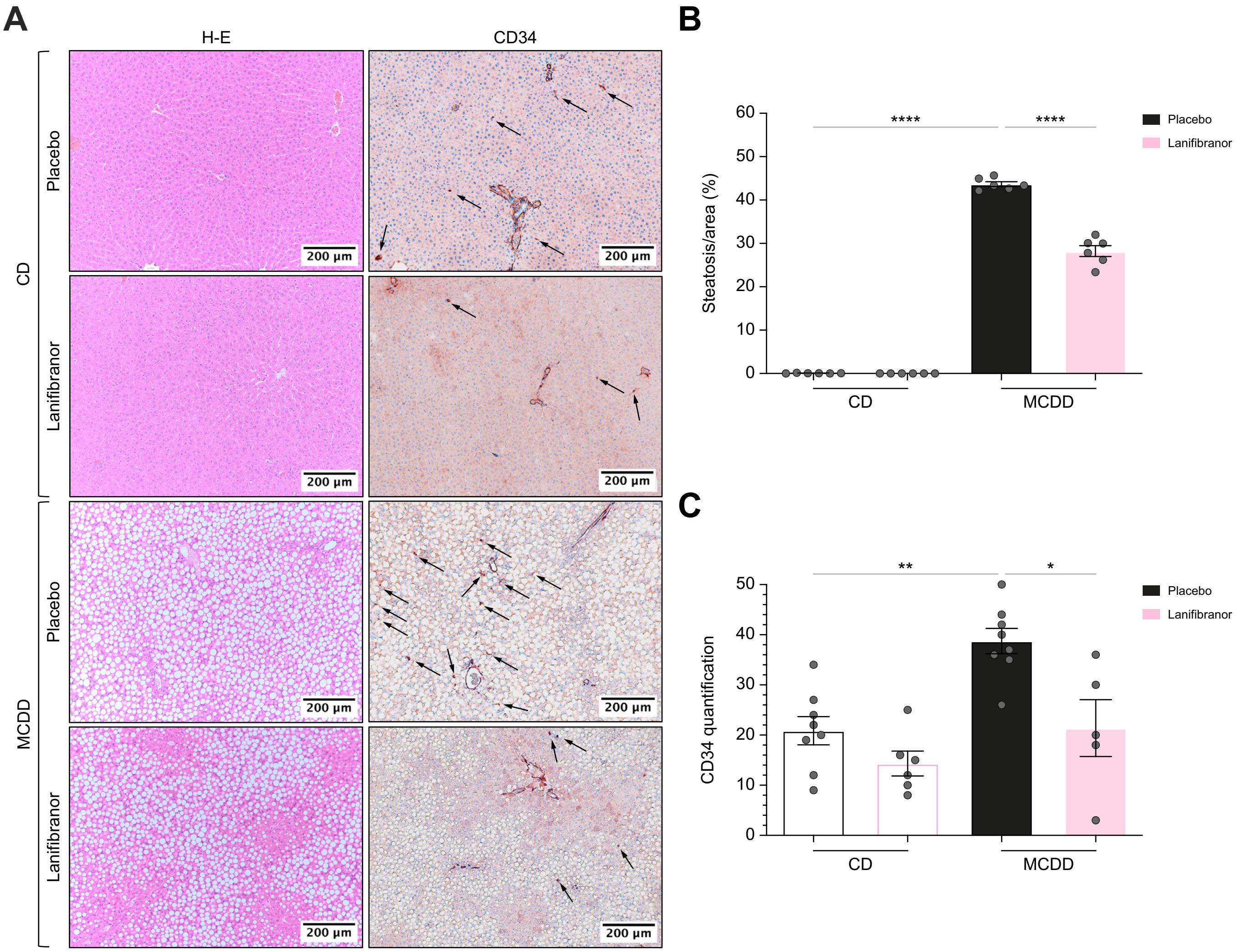

Fig. 6. Assessment of steatosis and LSEC capillarisation in early MASLD. Eight-week-old male Wistar Han rats (n = 6–8/group) were either fed a chow diet (CD) or a methionine-choline-deficient diet (MCDD) for 4 weeks and preventively treated with either placebo or lanifibranor (100 mg/kg) daily QD via oral gavage. (A) Images of H&E-stained and CD34-stained liver tissue sections (Olympus BX43, microscope lens 10 × /0.45 NA Plan Apo; resolution 1 pixel = 0.442 μm). (B) Steatosis quantification defined as fraction of macrovesicular fat droplets per area (%). (C) Blinded CD34 semiquantification. Data were analysed using two-way ANOVA followed by the *post hoc* Tukey test and presented as mean ± standard error of the mean with $^{*}p < 0.05$; $^{**}p < 0.01$; $^{****}p < 0.0001$. Arrows indicate CD34-positive staining. LSEC, liver sinusoidal endothelial cell; MASLD, metabolic dysfunction-associated steatotic liver disease.

demonstrated uneven and enlarged diameters. Furthermore, a notable observation was the presence of numerous vessels that branched into *cul-de-sac*-like dilated vessel stumps, commonly referred to as blebs (Fig. S20B). Fenofibrate treatment substantially improved the sinusoidal organisation in MCDD-fed rats, with a more regular arrangement of the sinusoids comparable to control animals, and a considerable decrease of the number of blebs (Fig. S20C). GW501516 (Fig. S20D) induced minor improvements, whereas rosiglitazone had no noticeable effect, yielding images comparable to placebo-treated MCDD rats (Fig. S20E). Lanifibranor induced (heterogeneously distributed) improvements of the sinusoidal organisation compared with placebo-treated steatotic rat livers, with more regular and untangled patterns, and more small sinusoids with diameters resembling those of controls (Fig. S20F).

Discussion

In this study, we demonstrated in a large cohort of patients with MASLD that CD34 staining, representing LSEC capillarisation, appears already at the stage of MASL – hence before MASH onset – and increases with the severity of MASLD, being strongly linked to liver fibrosis, and to a lesser extent to liver inflammation, and that it regresses following treatment with the pan-PPAR agonist lanifibranor.[33] Then, using two animal models of MASLD, we extended those results and showed that the beneficial effect of lanifibranor on LSEC capillarisation was

Table 2. Baseline characteristics, haemodynamics, and pressures in a rat model of MASH.

		Chow diet		HFHFD	
**Parameter**	**Groups (n)**	**Placebo**	**Lanifibranor**	**Placebo**	**Lanifibranor**
**Demographic characteristics**					
Age (weeks)	30/29/30/30	8	8	8	8
Body weight (g): baseline	30/29/30/30	146.0 ± 5.2*	137.0 ± 4.5‡	219.0 ± 8.02	208.0 ± 6.0
Body weight (g): after 8 weeks	30/29/30/29	372.0 ± 5.2*	370.2 ± 5.6‡	691.9 ± 10.36	803.0 ± 14.9*
Δ Body Weight(g)	30/28/30/29	220.0 ± 8.2*	234.0 ± 6.0‡	469.6 ± 14.76	592.0 ± 17.6*
Liver weight (g)	29/29/28/28	11.4 (10.1; 12.0)*,†	11.0 (10.1; 11.8)†,‡	25.4 (23.4; 28.4)†	25.5 (24.1; 27.5)†
% liver/total body weight	29/29/28/28	3.0 (2.8; 3.2)*,†	2.8 (2.8; 3.1)†,‡	3.7 (3.4; 4.1)†	3.1 (3.0; 3.6)*,†
Spleen weight (mg)	6/5/6/6	555.0 ± 41.1*	618.0 ± 29.1	802.0 ± 40.0	689.0 ± 56.4
% spleen/total body weight	6/5/6/6	0.15 ± 0.01*	0.16 ± 0.03‡	0.12 ± 0.00	0.09 ± 0.01*
**Haemodynamics and pressures**					
MABP (mmHg)	19/18/19/16	133.0 ± 2.7*	129.0 ± 3.2	159.0 ± 2.5	133.0 ± 3.0*
In vivo portal pressure (mmHg)	19/18/19/16	4.9 ± 0.3*	4.9 ± 0.4	7.1 ± 0.2	5.2 ± 0.2*
Portal blood flow (ml/min)	19/20/19/14	19.4 ± 0.8	19.6 ± 0.7‡	21.5 ± 0.8	26.2 ± 1.5*
THPG (mmHg) at 20 ml/min	8/8/8/7	5.0 ± 0.2*	4.9 ± 0.1	6.3 ± 0.2	4.9 ± 0.2*
THPG (mmHg) at 30 ml/min	8/8/8/7	6.3 ± 0.3*	6.2 ± 0.1	7.7 ± 0.2	6.4 ± 0.1*
**Histological parameters; H-E, PSR**					
Steatosis grade§	6/5/6/6	0 (0; 0)*,†	0 (0; 0)†,‡	1 (1; 2)†	1 (1; 1.3)†
S0 – no.		6	5	0	0
S1 – no.		0	0	4	5
S2 – no.		0	0	2	1
S3 – no.		0	0	0	0
Lobular inflammation grade¶		0 (0; 0.25)*,†	0 (0; 1)†	1 (1; 1.3)†	1 (0; 1)†
I0 – no.		5	3	0	2
I1 – no.		1	2	5	4
I2 – no.		0	0	1	0
I3 – no.		0	0	0	0
Ballooning grade**		0 (0; 0)*,†	0 (0; 0)†	1 (1; 1.3)†	0 (0; 1)†
B0 – no.		6	5	0	4
B1 – no.		0	0	5	2
B2 – no.		0	0	1	0
Fibrosis stage††		0 (0; 0)†	0 (0; 0)†	0 (0; 0)†	0 (0; 0)†
NAS		0 (0; 0.3)*,†	0 (0; 1)†	3 (3; 4.3)†	2 (1.8; 3)†
**Histological parameters; CD34 staining**					
CD34 quantification	6/5/6/6	21.3 ± 3.1	16.2 ± 4.7	28.0 ± 4.8	22.0 ± 2.2

Eight-week-old male Zucker fatty rats fed a high-fat high-fructose diet (HFHFD) and 8-week-old male Zucker lean rats fed a chow diet (CD) were concomitantly treated with either placebo or lanifibranor (100 mg/kg) daily QD via oral gavage during the whole period of 8 weeks of diet. Data were analysed using two-way ANOVA followed by the *post hoc* Tukey test and presented as mean ± standard error of the mean or the Kruskal–Wallis test followed by the Dunn test and presented as median (IQR). The THPG data were analysed using a generalised estimating equation model followed by least significant difference *post hoc* testing when appropriate. *For comparison with HFHFD + placebo; †CD + lanifibranor *vs.* CD + placebo; ‡CD + lanifibranor *vs.* HFHFD + lanifibranor; $^{*,\dagger,\ddagger}p$ <0.05. §Steatosis was assessed as the percentage of hepatocytes containing large and medium-sized intracytoplasmic lipid droplets and graded as 0 (<5%), 1 (5–33%), 2 (34–66%), or 3 (≥67%), according to the non-alcoholic steatohepatitis Clinical Research Network (NASH CRN) grading system. ¶Lobular inflammation was classified as grade 0 (no foci), grade 1 (<2 foci per 200 × field) or grade 2 (2–4 foci per 200 × field), according to the NASH CRN scoring system. **Ballooning was classified as grade 0 (no balloon hepatocyte) grade 1 (few but definite ballooned hepatocytes) or grade 2 (prominent ballooning), according to the NASH CRN grading system. ††Fibrosis was classified as stage F0 (no fibrosis), stage F1 (mild fibrosis), stage F2 (significant fibrosis), stage F3 (advanced fibrosis), or stage F4 (cirrhosis), according to the SAF–NASH CRN staging system. MABP, mean arterial blood pressure; NAS, NAFLD Activity Score; PVP, portal venous pressure; SAF, Steatosis-Activity-Fibrosis score; THPG, transhepatic pressure gradient.

accompanied by a functional improvement attested by a normalisation of portal pressure and of intrahepatic vascular resistance. The effect of lanifibranor was more pronounced than that of single PPAR agonists.

A first major observation in the present study is that CD34 LSEC expression is higher in the lobular area in patients with MASL than in those with no MASLD. Such an early change in LSEC phenotype is also observed here in rats fed a MCDD and had been previously reported in mouse models,[24] but had not been observed in patients to date. A previous study including 39 patients covering the MASLD spectrum did not reveal any difference in global CD34 staining between patients with MASL and those without MASLD, which is in line with our observation when only global CD34 staining was compared.[25] The increase in lobular CD34 expression between both groups appeared in our study when we refined the quantification by considering the zonation of CD34 staining. It is well known that the LSEC phenotype differs between periportal and lobular areas in the healthy liver.[39,40] We can thus speculate that lobular LSECs are particularly sensitive to stimuli derived from the portal vein in a context of metabolic syndrome, like excessive dietary macronutrients or gut microbiota-derived products, leading to early capillarisation in that area.[13] This localisation of LSEC capillarisation, confined at the early stages of MASLD to the lobular area, is reminiscent of the zone 3 perisinusoidal fibrosis typical for MASLD.[26,27] Although normal LSECs are known to maintain hepatic stellate cell quiescence, capillarised LSECs lose this ability, thus allowing fibrosis deposition.[13] Our observations thus reinforce the hypothesis that LSEC capillarisation occurs in MASLD before the development of liver fibrosis and

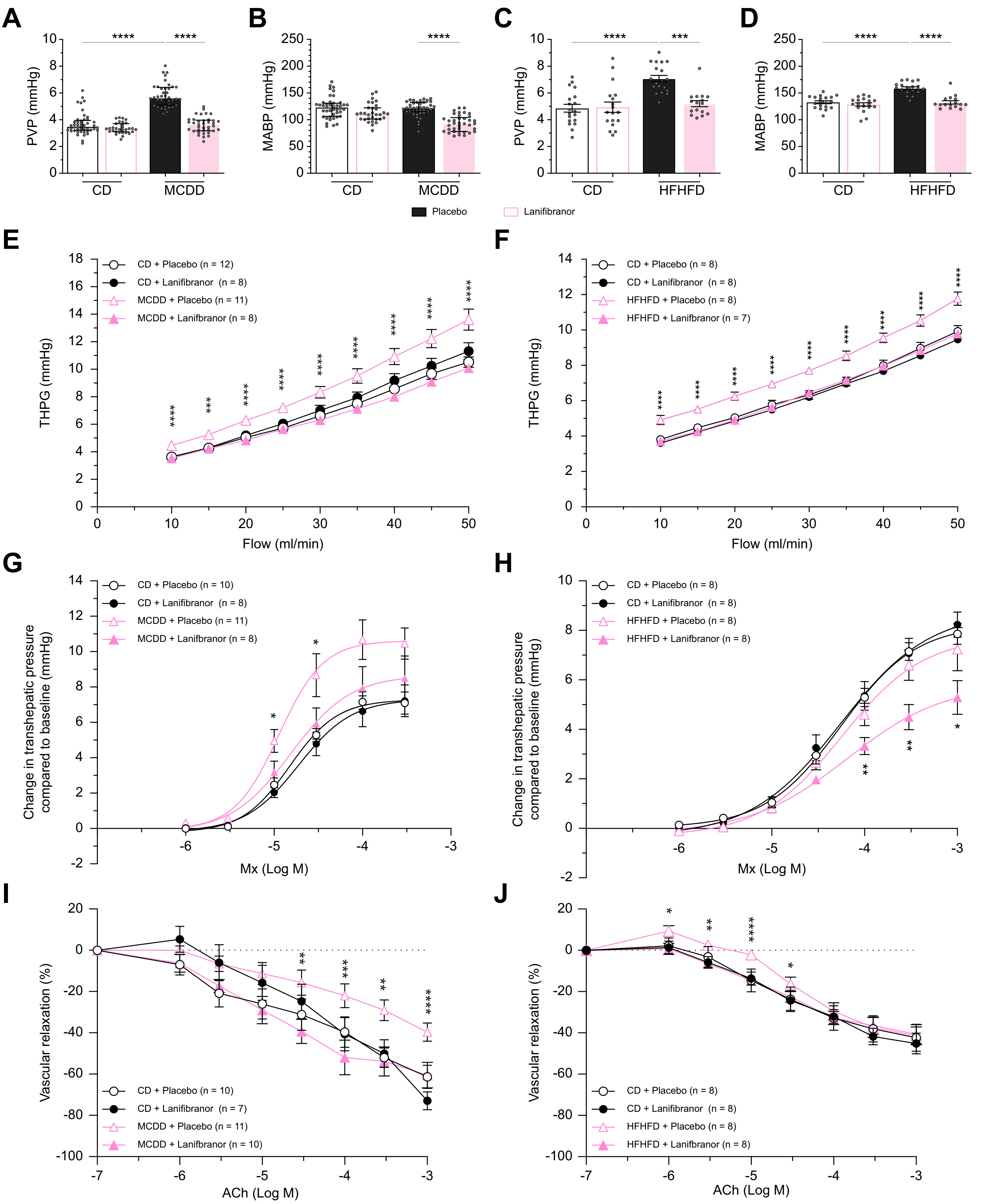

Fig. 7. ***In vivo*** **haemodynamics and pressures assessment, and** ***in situ ex vivo*** **liver perfusions in two preclinical models of MASLD.** Eight-week-old male Wistar Han rats (n = 7–12/group per experiment) were either fed a chow diet (CD) or a methionine-choline-deficient diet (MCDD) for 4 weeks and preventively treated with either placebo or lanifibranor (100 mg/kg) daily QD via oral gavage. *In vivo* parameters: (A) PVP, (B) MABP. *Ex vivo* parameters: (E) THPG, (G) dose–response Mx, (I) dose–response ACh. Model 2: 8-week-old male Zucker fatty rats (n = 7–8/group per experiment) fed a high-fat high-fructose diet (HFHFD) and 8-week-old male Zucker

contributes to its development[13] and are also in line with the observations of endothelial dysfunction early in the disease course.[16,41]

A second major finding of this study is a strong link between liver CD34 staining and liver fibrosis – and to a lesser extent liver inflammation – observed in 249 patients covering the whole MASLD spectrum (except for MASH cirrhosis), consistent with the associations between liver CD34 staining and FIB-4 (a marker of liver fibrosis), serum AST, ALT, and cytokeratin 18 M65 (a marker of cell death) concentrations observed in the same patients. In cohorts of 37 and 39 patients, higher CD34 staining has been described in patients with MASH as compared with MASL,[25,42] further increasing with the stage of liver fibrosis.[42] The present study not only firmly establishes those associations, but also identifies that they are much more pronounced for lobular than for periportal CD34 staining. Importantly, our large patient population allows us to confidently rule out a link between CD34 staining and hepatocyte ballooning or steatosis, suggesting that drivers for LSEC capillarisation might not be derived from hepatocytes but possibly rather from circulating cells or mediators present in the portal blood in the context of metabolic syndrome. Dedicated studies would be needed to investigate this hypothesis.

A third major finding is that CD34 staining can regress with MASH treatment – in this case lanifibranor – after only 24 weeks in patients, which is in line with our preclinical observations. The rapid regression of CD34 staining observed in the present study contrasts with data obtained in patients with hepatitis C virus-related cirrhosis where CD34 staining remained unchanged 5 years after sustained virological response.[43] A first explanation for these differences could be that the patients included in the present study were at a less advanced stage of their liver disease, as none had cirrhosis. Indeed, capillarisation might regress more quickly at earlier stages of the liver disease. Another explanation could be that the observed effect is related to the drug itself, as PPARs regulate endothelial function and phenotype,[44–46] besides glucose and fatty acid metabolism.[5] Lanifibranor (IVA337) is a pan-PPAR agonist that has a moderate and well-balanced activity on the three PPAR isoforms, thereby addressing the different components of MASH.[36,47] Investigations carried out in the present study, using two rat models of MASLD, support that view of an effect of lanifibranor on endothelial function and phenotype and extend the previous demonstration of a beneficial effect of lanifibranor in animal models of cirrhosis.[32] Indeed, we observed that only lanifibranor was able to normalise the IHVR, as attested by normalisation of PVP and of the *ex vivo* measured THPG, whereas PPARs mono-agonists showed only partial improvements. This effect of lanifibranor was accounted for by a restoration of liver endothelial function rather than to an effect on liver steatosis that was only partially improved. This vascular effect of lanifibranor might explain the significant results of this drug on both MASH resolution and fibrosis regression after only 24 months of treatment, compared with the absence of efficacy in terms of fibrosis regression by the PPAR-γ agonist pioglitazone or the glucagon-like peptide 1 receptor agonist semaglutide after 1.5 years of treatment.[48]

A final interesting finding is that lanifibranor decreased the mean arterial blood pressure (MABP) in MASLD animals. This appeared to be mainly a PPAR-β/δ agonistic effect. Interestingly, the MABP lowering effect by lanifibranor was also observed in patients during the NATIVE 2b trial.[31] Patients with MASH usually have cardiometabolic alterations including hypertension, and treatments that improve their cardiometabolic health can be beneficial beyond a pure liver-centred benefit.[34] This needs, however, further confirmation in the ongoing phase III NATiV3 trial (NCT04849728).

In conclusion, this study showed that LSEC capillarisation occurred in patients already at the stage of simple steatosis, just as in animal models. In patients, LSEC capillarisation further increased with MASH, and was strongly associated with liver fibrosis and to a lesser extent inflammation, but regressed following treatment with the pan-PPAR agonist lanifibranor. Lanifibranor also normalised PVP and IHVR in rats with early MASLD as well as in those with MASH, mainly by improving functional alterations, but also structural vascular alterations. The effect of lanifibranor was more pronounced than that of mono-PPAR agonists.

Affiliations

[1]*Université Paris-Cité, Inserm, Centre de recherche sur l'inflammation, Paris, France;* [2]*AP-HP, Hôpital Beaujon, Service d'Hépatologie, DMU DIGEST, Centre de Référence des Maladies Vasculaires du Foie, FILFOIE, ERN RARE-LIVER, Clichy, France;* [3]*Department of Gastroenterology and Hepatology, Antwerp University Hospital, Antwerp, Belgium;* [4]*Laboratory of Experimental Medicine and Paediatrics, University of Antwerp, Antwerp, Belgium;* [5]*INVENTIVA, Daix, France and New York, NY, USA;* [6]*Department of Hepatology, Beijing Hospital of Traditional Chinese Medicine, Capital Medical University, Beijing, China;* [7]*Department of Morphology, Imaging, Orthopedics, Rehabilitation and Nutrition, Faculty of Veterinary Medicine, Ghent University, Merelbeke, Belgium;* [8]*Comparative Perinatal Development, Department of Veterinary Sciences, Faculty of Pharmaceutical, Biomedical and Veterinary Sciences, University of Antwerp, Antwerp, Belgium;* [9]*Laboratory of Cell Biology and Histology, University of Antwerp, Antwerp, Belgium;* [10]*Antwerp Centre for Advanced Microscopy (ACAM), University of Antwerp, Antwerp, Belgium;* [11]*μNEURO, Centre of Excellence, University of Antwerp, Antwerp, Belgium;* [12]*Liverpat, Paris, France;* [13]*Division of Gastroenterology and Hepatology, Mayo Clinic, Rochester, MN, USA;* [14]*AP-HP, Hôpital Beaujon, Department of Pathology, FHU MOSAIC, Clichy, France*

lean rats (n = 8/group per experiment) fed a chow diet (CD) were concomitantly treated with either placebo or lanifibranor (100 mg/kg) daily QD via oral gavage during the complete period of 8 weeks of diet. *In vivo* parameters: (C) PVP, (D) MABP. *Ex vivo* parameters: (F) THPG, (H) dose–response Mx, (J) dose–response ACh. Pooled *in vivo* data (n = 16–43/group) were analysed using two-way ANOVA (C,D) followed by the *post hoc* Tukey test and presented as mean ± standard error of the mean or Kruskal–Wallis test (A,B) followed by the Dunn test and presented as median (IQR). The THPG and vascular relaxation data were analysed using a generalised estimating equation model followed by least significant difference *post hoc* testing. Data are presented as mean ± SEM. **p* <0.05; ***p* <0.01; ****p* <0.001; *****p* <0.0001. For clarity only the comparisons with MCDD-placebo are shown in perfusion graphs. ACh, acetylcholine; Log M, logarithmic concentration in mol/L; MABP, mean arterial blood pressure; MASLD, metabolic dysfunction-associated steatotic liver disease; Mx, methoxamine; PVP, portal venous pressure; QD, once per day; THPG, transhepatic pressure gradient.

Abbreviations

ACh, acetylcholine; AST, aspartate aminotransferase; ALT, alanine aminotransferase; CCVP, caudal cava venous pressure; CD, chow diet; CRN, Clinical Research Network; EC_{50}, half maximal effective concentration; Emax, maximum effect; EGR, erythroblast transformation-specific related gene; ET-1, endothelin-1; FIB-4, Fibrosis-4; HFHFD, high-fat high-fructose diet; IHVR, intrahepatic vascular resistance; LSECs, liver sinusoidal endothelial cells; MABP, mean arterial blood pressure; MASL, metabolic dysfunction-associated steatotic liver; MASLD, metabolic dysfunction-associated steatotic liver disease; MASH, metabolic dysfunction-associated steatohepatitis; MCDD, methionine-choline-deficient diet; Mx, methoxamine; NAFLD, non-alcoholic fatty liver disease; NAS, NAFLD Activity Score; PPARs, peroxisome proliferator-activated receptors; PVP, portal venous pressure; QD, once a day; SAF, Steatosis-Activity-Fibrosis score; TBW, total body weight; THPG, transhepatic pressure gradient; ZFR, Zucker fatty rat; ZLR, Zucker lean rat.

Financial support

This study was funded by Inventiva Pharma. P-ER's research laboratory is supported by the Fondation pour la Recherche Médicale (FRM EQU202303016287), 'Institut National de la Santé et de la Recherche Médicale' (ATIP AVENIR), by the 'Agence Nationale pour la Recherche' (ANR-18-CE14-0006-01, RHU QUID-NASH, ANR-18-IDEX-0001, ANR-22-CE14-0002), by 'Émergence, Ville de Paris', by Fondation ARC (R23087HH), by the European Union's Horizon 2020 research and innovation programme under grant agreement No 847949, and by France 2030 RHU LIVER-TRACK (ANR-23-RHUS-0014). SMF holds a senior clinical investigator fellowship from the Research Foundation Flanders (FWO) (1802154N). WDV holds investigator fellowships from the FWO (I000123N, I003420N).

Conflicts of interest

P-ER has received research funding from Terrafirma and acted as consultant for Mursla, Genfit, Boehringer Ingelheim, Cook, Jazz, and Abbelight, and received speaker fees from AbbVie. SMF has been lecturer for AbbVie, Allergan, Bayer, Eisai, Genfit, Gilead Sciences, Janssens Cilag, Intercept, Inventiva, Merck Sharp & Dome, Novo Nordisk, Promethera, Siemens. He has acted as consultant for AbbVie, Actelion, Aelin Therapeutics, AgomAb, Aligos Therapeutics, Allergan, Astellas, Astra Zeneca, Bayer, Boehringer Ingelheim, Bristoll-Meyers Squibb, CSL Behring, Coherus, Echosens, Eisai, Enyo, Galapagos, Galmed, Genetech, Genfit, Genflow Bio, Gilead Sciences, Intercept, Inventiva, Janssens Pharmaceutica, Julius Clinical, Madrigal, Medimmune, Merck Sharp & Dome, NGM Bio, Novartis, Novo Nordisk, PRO.MED.CS, Promethera, Roche. His institution has received grants from Astellas, Falk Pharma, Genfit, Gilead Sciences, GlympsBio, Janssens Pharmaceutica, Inventiva, Merck Sharp & Dome, Pfizer, Roche. WJK received lecturer fees for the PanNASH initiative and received travel grants from Ipsen and Norgine. He is a co-inventor of a patent on the use of lipopigment imaging for disease (filed by MGH/MIT: US 20190307390). MFA has acted as an advisor for 89Bio, Boehringer Ingelheim, Hanmi, Intercept, Inventiva, Madrigal, and Novo Nordisk. She has received grants (paid to her institution) from 89Bio, Akero, Hamni, Inventiva, Madrigal and Novo Nordisk. She has served as a speaker for MedScape, Chronic Liver Disease Foundation, Clinical Care Options, and Fishawack, Inc.

Please refer to the accompanying ICMJE disclosure forms for further details.

Authors' contributions

Conceptualisation (lead): P-ER, VP, SMF. Conceptualisation (equal): SC, J-LJ, WJK. Visualization (lead): SC. Investigation (lead): SC. Investigation (equal): YL, CC, ST, PB, PH-M, LD, MFA. Methodology (equal): P-ER, SC, LB, DVdG, YL, JDM, CC, ST, WHDV, PB, PH-M, LD, MA, LV, WJK, VP, SF. Data curation (lead): P-ER, SC. Data curation (equal): WJK. Software (equal): WHDV. Formal analysis (lead): P-ER, SC, LB. Supervision (lead): P-ER, WJK, VP, SF. Supervision (equal): DVdG, JDM, LV. Writing – original draft (lead): P-ER, SC, LB. Writing – review and editing (lead): WJK, VP, SF. Writing – review and editing (equal): GW, DVdG, YL, JDM, CC, WHDV, PB, MPC, MB, J-LA, PH-M, LD, PB, J-LJ, LV, MFA. Funding acquisition (lead): P-ER, GW, PB, VP, SF.

Data availability statement

The data that support the findings of this study are available from the corresponding author, upon reasonable request.

Acknowledgements

The authors thank Mandy Vermont, Lieve Vitz, Annelies De Bondt, Marleen Verhoye, Nicky Cortenberghe, Petra Aerts, and Amber Verhaegen for their technical support.

Supplementary data

Supplementary data to this article can be found online at https://doi.org/10.1016/j.jhepr.2025.101366.

References

Author names in bold designate shared co-first authorship

[1] Rinella ME, Lazarus JV, Ratziu V, et al. A multisociety Delphi consensus statement on new fatty liver disease nomenclature. J Hepatol 2023;79:1542–1556.
[2] EASL–EASD–EASO Clinical Practice Guidelines for the management of non-alcoholic fatty liver disease. J Hepatol 2016;64:1388–1402.
[3] Rinella ME, Neuschwander-Tetri BA, Siddiqui MS, et al. AASLD Practice Guidance on the clinical assessment and management of nonalcoholic fatty liver disease. Hepatology 2023;77:1797–1835.
[4] **Qi X**, **Li J**, Caussy C, et al. Epidemiology, screening, and co-management of type 2 diabetes mellitus and metabolic dysfunction-associated steatotic liver disease. Hepatology 2024. https://doi.org/10.1097/HEP.0000000000000913.
[5] **Francque S**, **Szabo G**, Abdelmalek MF, et al. Nonalcoholic steatohepatitis: the role of peroxisome proliferator-activated receptors. Nat Rev Gastroenterol Hepatol 2021;18:24–39.
[6] Parthasarathy G, Revelo X, Malhi H. Pathogenesis of nonalcoholic steatohepatitis: an overview. Hepatol Commun 2020;4:478–492.
[7] **Huby T**, **Gautier EL**. Immune cell-mediated features of non-alcoholic steatohepatitis. Nat Rev Immunol 2022;22:429–443.
[8] **Sharma S**, **Le Guillou D**, Chen JY. Cellular stress in the pathogenesis of nonalcoholic steatohepatitis and liver fibrosis. Nat Rev Gastroenterol Hepatol 2023;20:662–678.
[9] Baffy G. Origins of portal hypertension in nonalcoholic fatty liver disease. Dig Dis Sci 2018;63:563–576.
[10] Francque S, Verrijken A, Mertens I, et al. Visceral adiposity and insulin resistance are independent predictors of the presence of non-cirrhotic NAFLD-related portal hypertension. Int J Obes (Lond) 2011;35:270–278.
[11] Francque S, Verrijken A, Mertens I, et al. Noncirrhotic human nonalcoholic fatty liver disease induces portal hypertension in relation to the histological degree of steatosis. Eur J Gastroenterol Hepatol 2010;22:1449–1457.
[12] **Moga L**, **Laroyenne A**, Larrue H, et al. Patients with NAFLD do not have severe portal hypertension in the absence of cirrhosis. J Hepatol 2021;74:1269–1270.
[13] Hammoutene A, Rautou P-E. Role of liver sinusoidal endothelial cells in nonalcoholic fatty liver disease. J Hepatol 2019;70:1278–1291.
[14] van der Graaff D, Kwanten WJ, Francque SM. The potential role of vascular alterations and subsequent impaired liver blood flow and hepatic hypoxia in the pathophysiology of non-alcoholic steatohepatitis. Med Hypotheses 2019;122:188–197.
[15] Francque S, Wamutu S, Chatterjee S, et al. Non-alcoholic steatohepatitis induces non-fibrosis-related portal hypertension associated with splanchnic vasodilation and signs of a hyperdynamic circulation in vitro and in vivo in a rat model. Liver Int 2010;30:365–375.
[16] **Francque S**, **Laleman W**, Verbeke L, et al. Increased intrahepatic resistance in severe steatosis: endothelial dysfunction, vasoconstrictor overproduction and altered microvascular architecture. Lab Invest 2012;92:1428–1439.
[17] **Van der Graaff D**, **Kwanten WJ**, Couturier FJ, et al. Severe steatosis induces portal hypertension by systemic arterial hyporeactivity and hepatic vasoconstrictor hyperreactivity in rats. Lab Invest 2018;98:1263–1275.
[18] Pasarín M, La Mura V, Gracia-Sancho J, et al. Sinusoidal endothelial dysfunction precedes inflammation and fibrosis in a model of NAFLD. PLoS One 2012;7:e32785.
[19] Pasarín M, Abraldes JG, Liguori E, et al. Intrahepatic vascular changes in non-alcoholic fatty liver disease: potential role of insulin-resistance and endothelial dysfunction. World J Gastroenterol 2017;23:6777–6787.

[20] Van Eyck A, Kwanten WJ, Peleman C, et al. The role of adipose tissue and subsequent liver tissue hypoxia in obesity and early stage metabolic dysfunction associated steatotic liver disease. Int J Obes (Lond) 2024;48:512–522.
[21] Peleman C, De Vos WH, Pintelon I, et al. Zonated quantification of immunohistochemistry in normal and steatotic livers. Virchows Arch 2023;482:1035–1045.
[22] **Gao J**, **Lan T**, **Kostallari E**, et al. Angiocrine signaling in sinusoidal homeostasis and liver diseases. J Hepatol 2024;81:543–561.
[23] McConnell MJ, Kostallari E, Ibrahim SH, et al. The evolving role of liver sinusoidal endothelial cells in liver health and disease. Hepatology 2023;78:649–669.
[24] Miyao M, Kotani H, Ishida T, et al. Pivotal role of liver sinusoidal endothelial cells in NAFLD/NASH progression. Lab Invest 2015;95:1130–1144.
[25] Lefere S, Van de Velde F, Hoorens A, et al. Angiopoietin-2 promotes pathological angiogenesis and is a therapeutic target in murine nonalcoholic fatty liver disease. Hepatology 2019;69:1087–1104.
[26] Bedossa P, Poitou C, Veyrie N, et al. Histopathological algorithm and scoring system for evaluation of liver lesions in morbidly obese patients. Hepatology 2012;56:1751–1759.
[27] Kleiner DE, Makhlouf HR. Histology of nonalcoholic fatty liver disease and nonalcoholic steatohepatitis in adults and children. Clin Liver Dis 2016;20:293–312.
[28] Petta S, Targher G, Romeo S, et al. The first MASH drug therapy on the horizon: current perspectives of resmetirom. Liver Int 2024;44:1526–1536.
[29] Allen AM, Younossi ZM, Diehl AM, et al. Envisioning how to advance the MASH field. Nat Rev Gastroenterol Hepatol 2024;21:726–738.
[30] Albhaisi SAM, Sanyal AJ. New drugs for NASH. Liver Int 2021;41:112–118.
[31] Francque SM, Bedossa P, Abdelmalek MF, et al. A randomised, double-blind, placebo-controlled, multi-centre, dose-range, proof-of-concept, 24-week treatment study of lanifibranor in adult subjects with non-alcoholic steatohepatitis: design of the NATIVE study. Contemp Clin Trials 2020;98:106170.
[32] **Boyer-Diaz Z**, **Aristu-Zabalza P**, Andrés-Rozas M, et al. Pan-PPAR agonist lanifibranor improves portal hypertension and hepatic fibrosis in experimental advanced chronic liver disease. J Hepatol 2021;74:1188–1199.
[33] Francque SM, Bedossa P, Ratziu V, et al. A randomized, controlled trial of the pan-PPAR agonist lanifibranor in NASH. N Engl J Med 2021;385:1547–1558.
[34] Cooreman MP, Butler J, Giugliano RP, et al. The pan-PPAR agonist lanifibranor improves cardiometabolic health in patients with metabolic dysfunction-associated steatohepatitis. Nat Commun 2024;15:3962.
[35] **Haber MA**, **Iranmahboob A**, Thomas C, et al. ERG is a novel and reliable marker for endothelial cells in central nervous system tumors. Clin Neuropathol 2015;34:117–127.
[36] Boubia B, Poupardin O, Barth M, et al. Design, synthesis, and evaluation of a novel series of indole sulfonamide peroxisome proliferator activated receptor (PPAR) $\alpha/\gamma/\delta$ triple activators: discovery of lanifibranor, a new antifibrotic clinical candidate. J Med Chem 2018;61:2246–2265.
[37] Kleiner DE, Brunt EM, Van Natta M, et al. Design and validation of a histological scoring system for nonalcoholic fatty liver disease. Hepatology 2005;41:1313–1321.
[38] Sterling RK, Lissen E, Clumeck N, et al. Development of a simple noninvasive index to predict significant fibrosis in patients with HIV/HCV coinfection. Hepatology 2006;43:1317–1325.
[39] Strauss O, Phillips A, Ruggiero K, et al. Immunofluorescence identifies distinct subsets of endothelial cells in the human liver. Sci Rep 2017;7:44356.
[40] MacParland SA, Liu JC, Ma X-Z, et al. Single cell RNA sequencing of human liver reveals distinct intrahepatic macrophage populations. Nat Commun 2018;9:4383.
[41] van der Graaff D, Chotkoe S, De Winter B, et al. Vasoconstrictor antagonism improves functional and structural vascular alterations and liver damage in rats with early NAFLD. JHEP Rep 2022;4:100412.
[42] Kitade M, Yoshiji H, Kojima H, et al. Neovascularization and oxidative stress in the progression of non-alcoholic steatohepatitis. Mol Med Rep 2008;1:543–548.
[43] D'Ambrosio R, Aghemo A, Rumi MG, et al. A morphometric and immunohistochemical study to assess the benefit of a sustained virological response in hepatitis C virus patients with cirrhosis. Hepatology 2012;56:532–543.
[44] Han L, Shen W-J, Bittner S, et al. PPARs: regulators of metabolism and as therapeutic targets in cardiovascular disease. Part II: PPAR-β/δ and PPAR-γ. Future Cardiol 2017;13:279–296.
[45] Han L, Shen W-J, Bittner S, et al. PPARs: regulators of metabolism and as therapeutic targets in cardiovascular disease. Part I: PPAR-α. Future Cardiol 2017;13:259–278.
[46] **Guixe-Muntet S**, **Biquard L**, Szabo G, et al. Review article: vascular effects of PPARs in the context of NASH. Aliment Pharmacol Ther 2022;56:209–223.
[47] Wettstein G, Luccarini JM, Poekes L, et al. The new-generation pan-peroxisome proliferator-activated receptor agonist IVA337 protects the liver from metabolic disorders and fibrosis. Hepatol Commun 2017;1:524–537.
[48] Newsome PN, Buchholtz K, Cusi K, et al. A placebo-controlled trial of subcutaneous semaglutide in nonalcoholic steatohepatitis. New Engl J Med 2021;384:1113–1124.

Keywords: Intrahepatic vascular resistance; Vascular biology; LSECs; CD34; MASH; Liver fibrosis; Liver inflammation; PPAR; lanifibranor.

Received 27 September 2024; received in revised form 11 February 2025; accepted 17 February 2025; Available online 22 February 2025

Journal of Hepatology, Volume *7*

Supplemental information

Altered liver sinusoidal endothelial cells in MASLD and their evolution following lanifibranor treatment

Pierre-Emmanuel Rautou, Shivani Chotkoe, Louise Biquard, Guillaume Wettstein, Denise van der Graaff, Yao Liu, Joris De Man, Christophe Casteleyn, Sofie Thys, Winnok H. De Vos, Pierre Bedossa, Michael P. Cooreman, Martine Baudin, Jean-Louis Abitbol, Philippe Huot-Marchand, Lucile Dzen, Miguel Albuquerque, Pierre Broqua, Jean-Louis Junien, Luisa Vonghia, Manal F. Abdelmalek, Wilhelmus J. Kwanten, Valérie Paradis, and Sven M. Francque

Altered liver sinusoidal endothelial cells in MASLD and their evolution following lanifibranor treatment

Pierre-Emmanuel Rautou, Shivani Chotkoe, Louise Biquard, Guillaume Wettstein, Denise Van der Graaff, Yao Liu, Joris De Man, Christophe Casteleyn, Sofie Thys, Winnok H. De Vos, Pierre Bedossa, Michael P. Cooreman, Martine Baudin, Jean-Louis Abitbol, Philippe Huot-Marchand, Lucile Dzen, Miguel Albuquerque, Pierre Broqua, Jean-Louis Junien, Luisa Vonghia, Manal F. Abdelmalek, Wilhelmus J. Kwanten, Valérie Paradis, Sven M. Francque

Table of contents

Supplementary materials and methods…………………………………………….....2

Supplementary figures……………………………………………………………..….7

Supplementary tables………………………………………………………….….28

Supplementary references……………………………………………………..….46

Supplementary materials and methods

Clinical approaches

Liver biopsy and immunostaining

Liver biopsy slides were stained (Hematoxylin & Eosin, Picrosirius red staining, Perls staining) in a central histology laboratory. Histologic features of MASLD and MASH were assessed using the NASH Clinical Research Network (NASH CRN) Scoring System [1].

Formalin-fixed paraffin-embedded liver biopsies were subjected to CD34 immunostaining, a marker of LSEC capillarisation. Briefly, tissue sections (3 µm) underwent dewaxing, antigen retrieval, and then incubation with antibody against CD34 (monoclonal mouse anti-human CD34, ab8536, clone QBEnd-10, batch number GR49632-27, Abcam, UK, dilution 1:500) using an automated immunohistochemical stainer according to the manufacturer's guidelines (streptavidin-peroxidase protocol, BenchMark, Ventana). Negative controls were systematically included by incubating with PBS instead of primary antibody. To confirm CD34 colocalisation with endothelial cells, double immunostaining with antibody against Erythroblast transformation-specific related gene (ERG, a nuclear marker of LSECs [2]) (ABCAM, ab92513, dilution 1:50) was performed using Ultraview universal alkaline phosphatase red detection and Ultraview DAB kits (Ventana, Benchmark, USA) in selected cases (n=29).

Histological scoring of density of CD34 positive vessels

Quantitative analysis was assessed on digitised slides using Scanscope AT Turbo (LEICA©, Wetzlar, Germany). Slide scanning was performed at x20 magnification (microscope lens 20X/0.75 NA Plan Apo; resolution 1 pixel=0.5 µm) using the manufacturer's software (Imagescope, Leica). Digital images were analysed with a dedicated microvessel algorithm (HALO, Indica Labs, Albuquerque, US) that quantifies positive pixels (brown stained pixel defined by RGB composite) surrounding white areas **(Fig. S1)**. All images were annotated to exclude large vessels and portal tracts.

Preclinical approaches

Animal models

Early MASLD: Male Wistar Han rats (Charles River, Germany; 200-250 g) were fed a chow diet (CD, ICN Biomedicals SA, Asse, Belgium) or a methionine-choline-deficient diet (MCDD, Envigo RMS B.V., Indianapolis, Indiana USA) for 4 weeks, which is known to induce severe steatosis in the absence of MASH in this rat strain [3, 4]. This model hence recapitulates the liver MASLD phenotype and is suited to study the intrahepatic features of the disease, although it does not recapitulate the classical metabolic syndrome phenotype in which the disease usually develops. To study the intrahepatic effects of the different PPAR isotypes, rats (n = 6-8/group) underwent gavage once a day (QD) with either placebo (1% methylcellulose + 0.05% poloxamer), fenofibrate (PPAR-α agonist, 30 mg/kg), GW501516 (PPAR-β/δ agonist, 10 mg/kg), rosiglitazone (PPAR-γ agonist, 5 mg/kg) or lanifibranor [5] (100 mg/kg), during the entire 4 weeks of diet as a preventive treatment **(Fig. S2A)**. Lanifibranor (IVA337) is a pan-PPAR agonist with a unique molecular design that has a moderate and well-balanced activity on the three PPAR isoforms, thereby addressing the different components of MASH [5, 6]. To evaluate the specificity of lanifibranor for modulating PPAR isoforms, comprehensive off-target profiling for more than 100 targets was previously conducted using CEREP's (Eurofins) advanced screening platform. These tests confirmed that lanifibranor did not exhibit off-target modulation, demonstrating its specificity for PPAR pathways without affecting other molecular targets.

MASH: To exclude model specificity and to examine the effects at the stage of steatohepatitis rather than isolated steatosis, the most important results of lanifibranor were tested in a second model considered a more clinically relevant representation of MASLD: male Zucker fatty rats (ZFR, fa/fa) (Charles River, USA; 8 weeks old, n = 6-8/group) were fed a high-fat high-fructose diet (HFHFD, D16042610, Research Diets, New Brunswick, NJ, USA) and compared to lean control rats (ZLR, fa/+) (Charles River, USA; 8 weeks old, n = 6-8/group) fed a chow diet for 8 weeks. Additionally, ZFR were treated orally with lanifibranor (100 mg/kg) or placebo preventively during the complete 8 weeks of HFHFD **(Fig. S2B).** No treatment-related mortality was observed. All animals were kept in a 12 h:12 h light/dark cycle with controlled temperature, humidity, unlimited access to their food and water in enriched cages of up to two animals. The animals were treated according to the ARRIVE guidelines. The protocol was approved by the Antwerp University Ethical Committee on Animal Experiments (ECD 2021-24). With respect to the ethical guidelines, sample size was calculated with G*Power software considering effect size and variability based on prior data from our own research group and from the literature (specified in ECD 2021-24).

Western blots

Rat liver tissue was homogenised with a Precellys 24 homogeniser (Bertin technologies) in 1x RIPA lysis buffer (ab156034) containing a protease inhibitor cocktail (cOmplete Mine; Roche Diagnostics, Mannheim, Germany) and a phosphatase inhibitor cocktail (PhosSTOP; Roche). Protein concentrations were determined with a bicinchonicic acid assay (BCA). Protein samples of the liver were run on an SDS-polyacrylamide gel (Bolt™ Bis-Tris 4-12%; Life Technologies, Carlsbad (CA), USA) and transferred to a PVDF membrane. Membranes were subsequently blocked in Tris-buffered saline containing 0.1% Tween and 5% non-fat dry milk. Membranes were probed overnight at 4°C with primary antibody solution with the following antibodies: anti-COL1A (sc-59772, Santa Cruz Biotechnology, Germany) and anti-GAPDH (39-8600, Thermo Scientific). After incubation with appropriate horseradish peroxidase secondary antibodies, chemiluminescent signals were obtained with Pierce™ ECL Plus Western Blotting Substrate (32132; Thermo Scientific) using a ChemiDoc™ MP imaging system (Bio-Rad Laboratories). Each lane was loaded with 20 µg protein. Rat tail collagen type 1 (A1048301, Gibco™) served as positive control.

Histology

As the liver perfusion experiments at high flows may ultimately induce manipulation artefacts, separate groups of animals were used in parallel, solely for the purpose of tissue harvesting and analysis, in the same experimental settings. Rats (n = 6/group) were weighed, anaesthetised, and sacrificed. Livers were weighed, random samples were fixed in 4% formaldehyde (BDH Prolabo, VWR, Belgium), and subsequently dehydrated in 60% isopropanol, followed by paraffin-embedding.

5 µm sections were stained with haematoxylin-eosin (H-E) and picrosirius red according to standard laboratory protocols. Histologic features of MASLD and MASH were assessed using the NASH Clinical Research Network (NASH CRN) Scoring System [1]. Further, slides of H-E-stained liver samples were digitised with a Zeiss Axioscan (using a 10X/0.45 NA Plan Apo objective (at an image resolution of 0.442 µm /pixel). Steatosis was measured as the area fraction of a tissue region of fixed size (ROI size: 8.96 mm^2) covered by macrovesicular lipid droplets using ImageJ software (Bethesda, MD, USA). In brief, RGB images were first converted to 8-bit grayscale after which a manual, fixed intensity threshold was applied to only retain the lipid fraction. A subsequent binary watershed procedure was used to separate touching droplets, and a selective analysis of particles with a size in between 50 and 800 μm^2 and circularity above 0.5 was performed to obtain a mask of the lipid fraction. The relative area of the ROI covered by the resulting mask (in %) was used as measure for steatosis.

CD34 immunohistochemistry

Liver tissue sections were treated with heat mediated tris-EDTA buffer solution for antigen retrieval. The sections were incubated with rabbit recombinant monoclonal anti-CD34 antibody (1:2000, ab81289, Abcam, San Francisco, USA) in TBS-Tx overnight. After the sections were washed, the biotinylated goat anti-rabbit secondary antibody (1:200, PK-4001 Vectastain ABC-HRP kit, Labconsult, Belgium) in TBS-Tx was applied for 30 min. Subsequently, incubation with HRP-labelled avidin was performed for 60 min. Slides were stained with 3-amino-9-ethylcarbazole (AEC) substrate followed by haematoxylin staining.

Histological scoring of CD34 positive vessels

Analysis of CD34 staining was performed by a manually semi-quantitative approach counting all CD34-positive stains (size ≥ 3 µm) per image (arrows Fig. 6A & 7A) (portal tracts and central veins excluded) acquired with a 10X/0.25 NA Plan Apo objective (0.442 µm/pixel). Liver tissue section slides were blinded and per slide pictures were taken from 5 random fields, imaging 6 livers for each group. All images were acquired with Universal Grab 6.1 software using an Olympus BX43 microscope. The blindly acquired images of CD34 stained sections were scored using ImageJ software (Bethesda, MD, USA).

In vivo haemodynamics and blood pressures

Under general anaesthesia (ketamine-xylazine), a tracheal tube (PE 240 ID 1.67 mm OD 2.42 mm Intramedic Clay Adams brand non-radiopaque polyethylene tubing) was inserted by tracheostomy. The blood flow in the carotid artery, portal vein and caudal caval vein was measured using a 1 PR or 1.5 PR Doppler flow probe and a TS420 flowmeter (Transonic Systems, Inc., Ithaca, NY, USA). After blood flow assessment, a 24G catheter was inserted into the carotid artery. The abdomen was opened by a median incision. The portal vein was exposed and cannulated with a 24G catheter. The caudal cava vein was cannulated with a 22G catheter, which was advanced with the tip into the retrohepatic part of the caudal cava vein. The different catheters were connected to a Gould Statham pressure transducer (model P23 ID, Hato Rey, Puerto Rico). Haemodynamic and blood pressure variables were recorded with in-house monitoring equipment (Powerlab 8/30 and LabChart 7, AD Instruments, Oxford, UK). Carotid artery pressure (mean arterial blood pressure (MABP)), pulse rate (PR), portal venous pressure (PVP) and caudal cava vein pressure (CCVP) were measured.

In situ ex vivo liver perfusion

To investigate the IHVR in the same animals, the transhepatic pressure gradient (THPG) was assessed immediately after the *in vivo* measurements, directly by *in situ ex vivo* liver perfusion experiments (n = 6-8/group), as described previously [4]. Briefly, after the *in vivo* assessments, heparin (1,400 U/kg) was injected intravenously in the caudal cava vein. The portal vein was cannulated with a 14 G catheter, the thoracic cavity was opened and the suprahepatic cava vein was cannulated through the right atrium with a 16 G catheter. Next, the liver was perfused in a single-pass way by oxygenated Krebs-Ringer solution (37°C) and the catheters were connected to the previously mentioned pressure and flow monitoring equipment. After a stabilisation period of 20 min at a constant flow of 10 mL/min, the flow was gradually increased by 5 mL/min every 5 min, from 10 to 50 mL/min. In all experiments, the portal vein (inflow) pressure and the suprahepatic cava vein (outflow) pressure (which was kept at a constant level of -1 mmHg) were measured continuously and the THPG was calculated by subtracting the outflow from the inflow pressure.

Dose-response experiments

A syringe pump with vasoactive substance was connected to the *ex vivo in situ* perfusion set-up. The pump was started after a stabilisation period of 20 min at a constant perfusion flow of 30 mL/min. The dose was increased by 0.5 log every 5 min and the THPG was continuously measured while increasing the dose. The effect of PPAR agonists on hepatic vascular reactivity was tested with following vasoactive compounds: 10^{-12} to 3×10^{-9} mol/L endothelin-1 (ET-1), 10^{-6} to 3×10^{-4} mol/L methoxamine (Mx, alpha-1-adrenoceptor agonist), and 10^{-6}

to 3 × 10^{-4} mol/L acetylcholine (ACh, endothelial-dependent vasodilator) after preconstriction with 3 × 10^{-5} mol/L Mx for the MCDD model and 1.5 × 10^{-4} mol/L for the HFHFD model. Results are expressed in relative changes *(i.e.*, the increase or decrease in THPG compared to the baseline value before the compound was added).

Vascular corrosion casting

To explore the architecture of the hepatic vasculature, rats (n = 3/group) from the early MASLD model were sacrificed and vascular corrosion casts were prepared as previously described [3, 7]. The casts were examined systematically by scanning electron microscopy (Jeol JSM-IT100, Jeol, Tokyo, Japan). Images were made at x100 and x300 magnification and at 20.0 kV, and were subsequently described visually.

Drugs and solutions

Animals were anesthetized with a combination of ketamine (KETALAR®, 100 mg/kg body weight, Pfizer, Berlin, Germany) and xylazine (Rompun® 2%, 10 mg/kg body weight, Bayer, Leverkusen, Germany) intraperitoneally.

The isotonic Krebs–Ringer solution had the following composition: KCl 4.75 mM, MgSO4·7H2O 1.2 mM, KH2PO4 1.2 mM, CaCl2·2H2O 2.5 mM, CaEDTA 0.03 mM, NaCl 118.5 mM, NaHCO3 25 mM, glucose 11.1 mM; pH = 7.4.

ACh and ET-1 were purchased from Sigma-Aldrich Chemie Gmbh (Steinheim, Germany). Mx was purchased from Santa Cruz Biotechnology, Inc. (Dallas, Texas). All drugs were first dissolved in aqua destillata before diluting them in the Krebs–Ringer solution to obtain the given concentrations. All solutions were freshly prepared on the day the experiments were performed.

Placebo (1% methylcellulose + 0.05% poloxamer) and PPAR agonists (solubilized with 1% methylcellulose + 0.05% poloxamer) for oral treatment were provided by Inventiva Pharma (Daix, France).

Statistical analyses

Clinical approaches:

Continuous variables were described using median (interquartile range, IQR) and compared using the Wilcoxon-Mann-Whitney test for two groups comparisons, or the Kruskall-Wallis test followed by Dunn test for more than two groups comparisons. The 95% two-sided confidence interval was calculated when appropriate. Categorical variables were described as percentages of each category. The 95% two-sided confidence interval was calculated when appropriate using the exact (Clopper-Pearson) method. Categorical variables were compared between two groups using the Chi² test or the Fisher's exact test, and between more than two groups using the Cochran–Armitage asymptotic test for trend followed by Chi² or Fisher tests. Threshold for Fib-4 classes were based on EASL recommendations for noninvasive tests for the evaluation of liver disease severity and prognosis [8]. Treatment effect on continuous variables was evaluated using a Mixed Model for Repeated Measures with the variable change from baseline as endpoint and the treatment group and variable baseline value as fixed effect. The link between two continuous variables was assessed using the Spearman correlation. Missing data were not replaced. No multiplicity adjustments were defined as all endpoints are to be considered exploratory. The confidence intervals have not been adjusted for multiple comparisons and should not be used to infer definitive treatment effects. SAS® software version 9.2 was used.

Preclinical approaches:

Variables were presented as mean ± standard error of the mean (SEM) or median (interquartile range (IQR)) when appropriate and compared with two-way ANOVA (with the diet as the first factor (between), the treatment used as the second factor (within)) for normally distributed data followed by Tukey test and Kruskall-Wallis test for skewed data followed Dunn test, using Prism 9 (Version 9.2.0; Graphpad, California, USA). The THPG data were analysed

using a generalised estimating equation model followed by least significant difference *post hoc* testing when appropriate, using SPPS v29.0 (IBM, Armonk, NY, USA).
A p value < 0.05 was considered to represent a statistical difference.

Supplementary figures

Fig. S1. CD34 histology and quantification

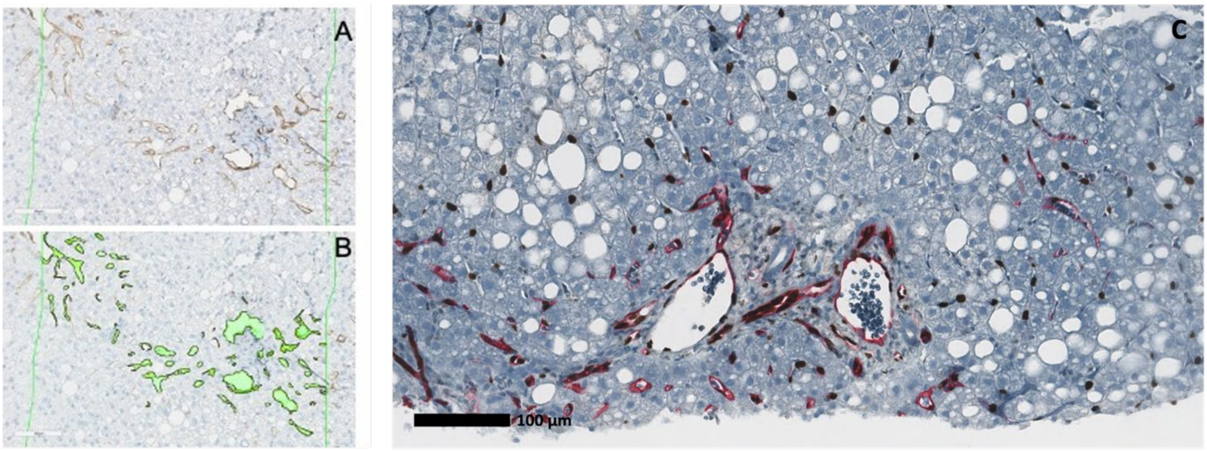

(A) CD34 immunostaining showing vessels delineating in brown; **(B)** segmentation of CD34 immunostaining using the microvessel algorithm (vessels are highlighted in green); **(C)** CD34 (pink) cytoplasmic staining co-localises with ERG nuclear staining (brown) in endothelial cells (representative of n=29).

Fig. S2A. Experimental design of an early MASLD rat model in a preventive treatment set-up.
Male Wistar Han rats of 8 weeks old were either fed a chow diet (CD) or a methionine-choline-deficient diet (MCDD) for 4 weeks and simultaneously treated with either placebo, fenofibrate (30 mg/kg), GW501516 (10 mg/kg), rosiglitazone (5 mg/kg) or lanifibranor (100 mg/kg) daily QD via oral gavage. After 4 weeks of diet and treatment, the following parameters were assessed: in vivo haemodynamics and blood pressures followed by ex vivo intrahepatic vascular resistance (IHVR) measurement in the same animals, dose-response experiments with endothelin-1 (ET-1), methoxamine (Mx) and acetylcholine (ACh), and histology.

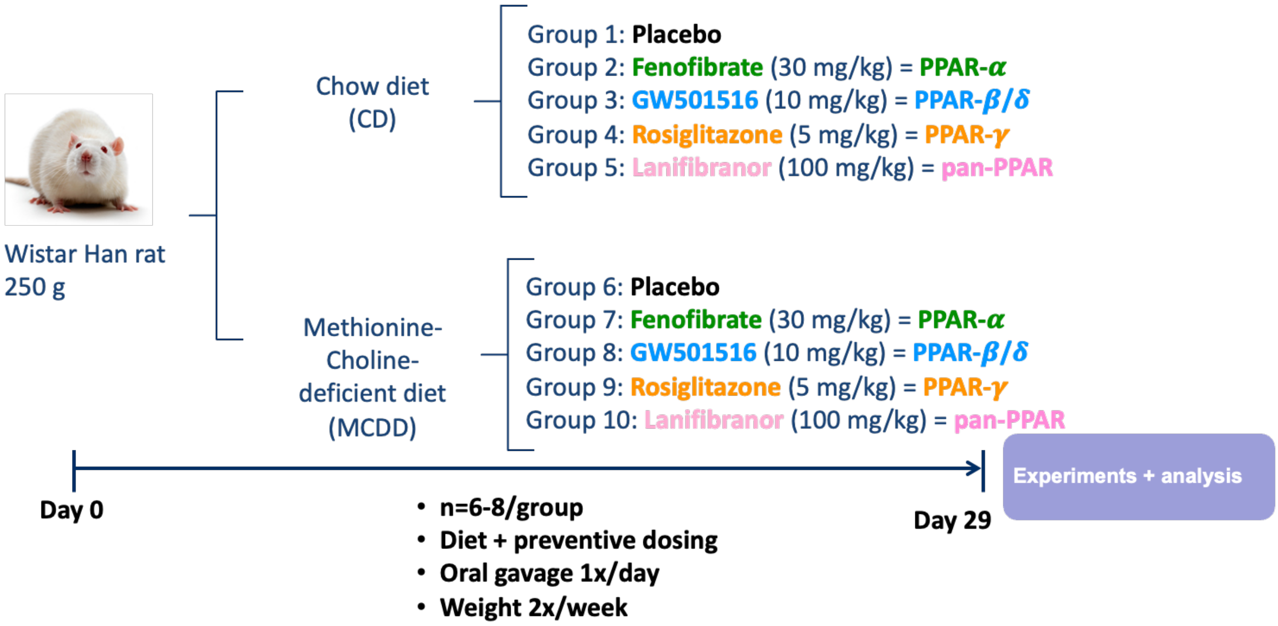

Fig. S2B. Experimental design of a rat model of MASH in a preventive treatment set-up.
8 weeks old male Zucker fatty rats fed a high-fat high-fructose diet (HFHFD) and 8 weeks old male Zucker lean rats fed a chow diet (CD) were concomitantly treated with either placebo or lanifibranor (100 mg/kg) daily QD via oral gavage during the whole period of 8 weeks of diet. After 8 weeks of diet and treatment, the following parameters were assessed: in vivo haemodynamics and blood pressures followed by ex vivo intrahepatic vascular resistance (IHVR) measurement in the same animals, dose-response experiments with methoxamine (Mx) and acetylcholine (ACh), and histology.

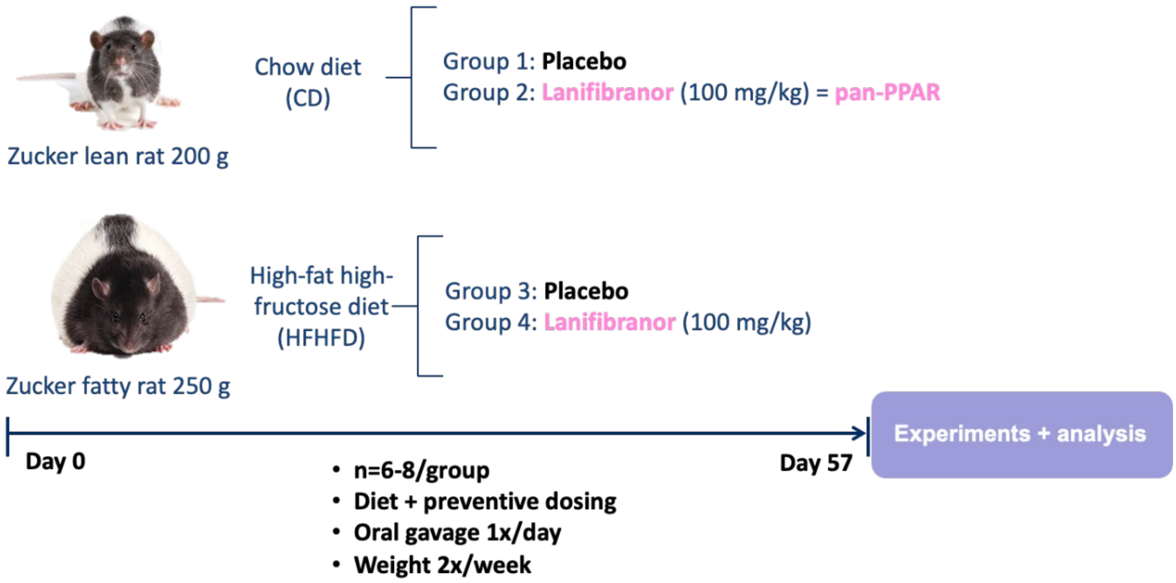

Fig. S3. Flow chart. Patients' disposition and definition of the analysed population

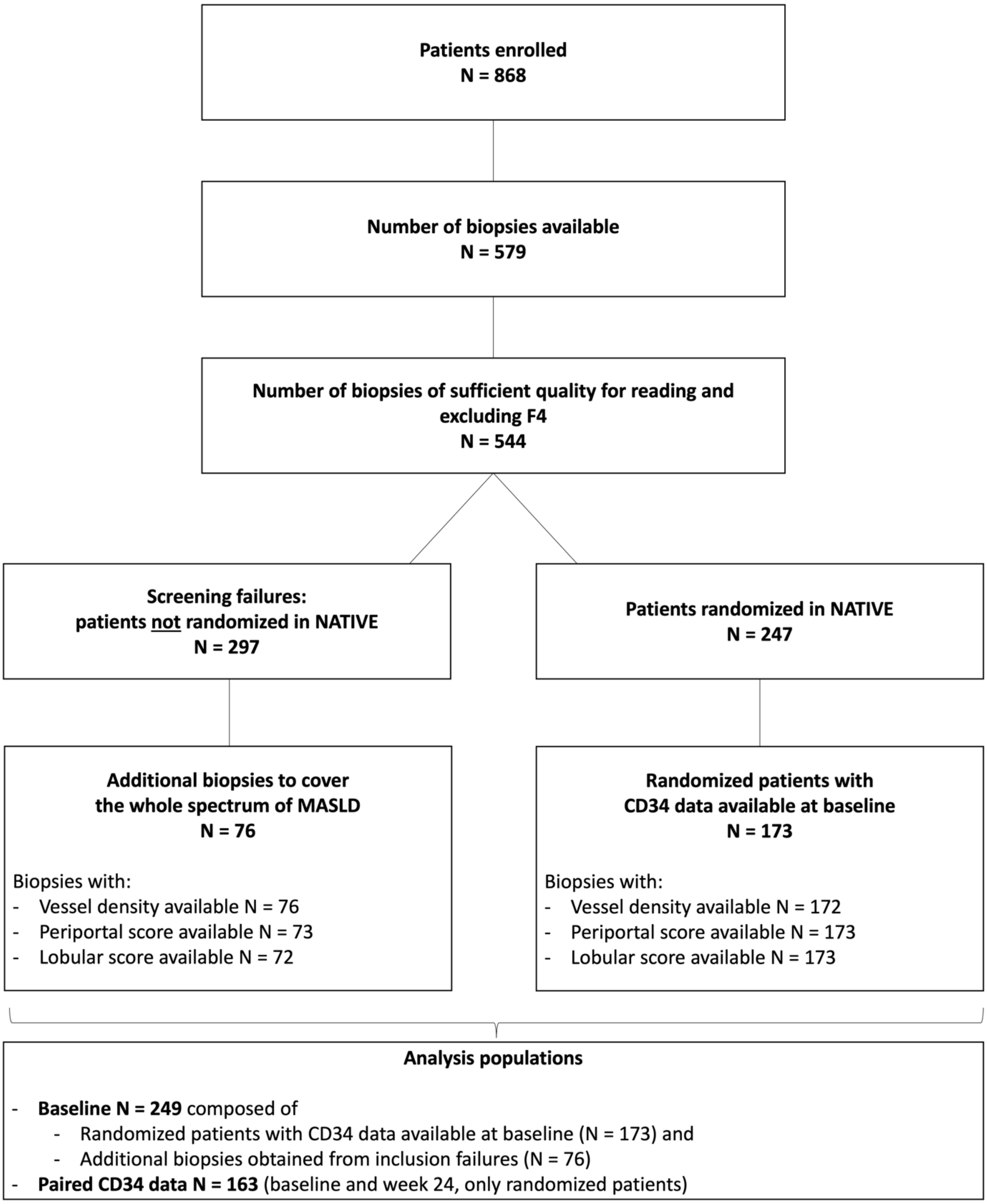

Fig. S4. Association between density of CD34 positive vessels and periportal and lobular scores.

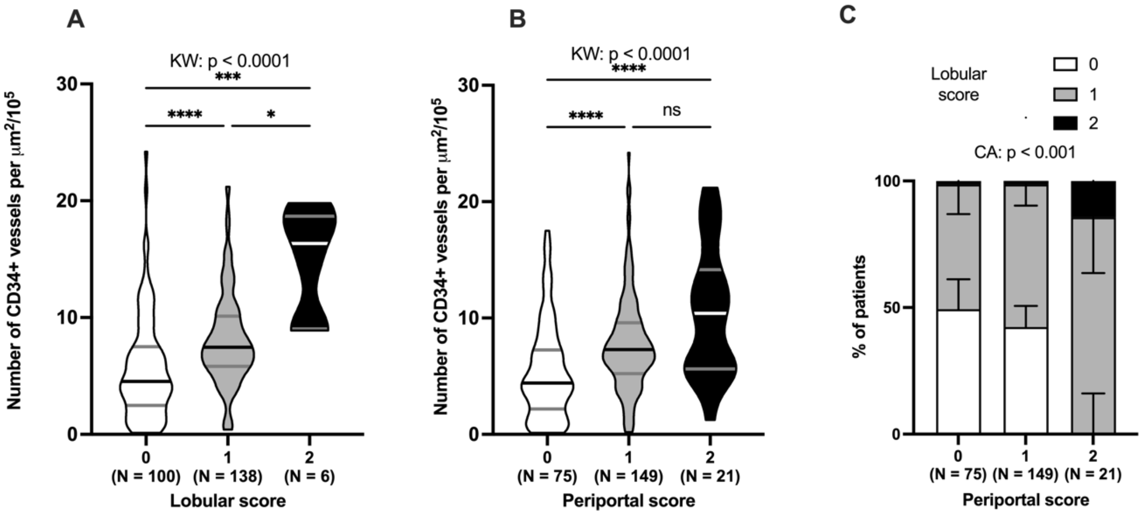

Density of CD34 positive vessels, periportal score and lobular score were available in 248, 246 and 245 patients, respectively, as detailed in Fig S3. Correlation between density of CD34 positive vessels and lobular score **(A)**, between density of CD34 positive vessels and periportal score **(B)** and between lobular and periportal scores **(C)** at baseline.

KW. Kruskal-Wallis; CA. Cochran-Armitage. When appropriate after Kruskal-Wallis, post-hoc Dunn's tests were performed between all columns with *: p<0.05; **: p<0.01; ***: p<0.001; ****: p<0.0001. For violin plots, the bars represent the median ± IQR, otherwise bars represent 95% confidence intervals.

Fig. S5. Relationship between CD34 staining level and localisation and histological features in MASLD

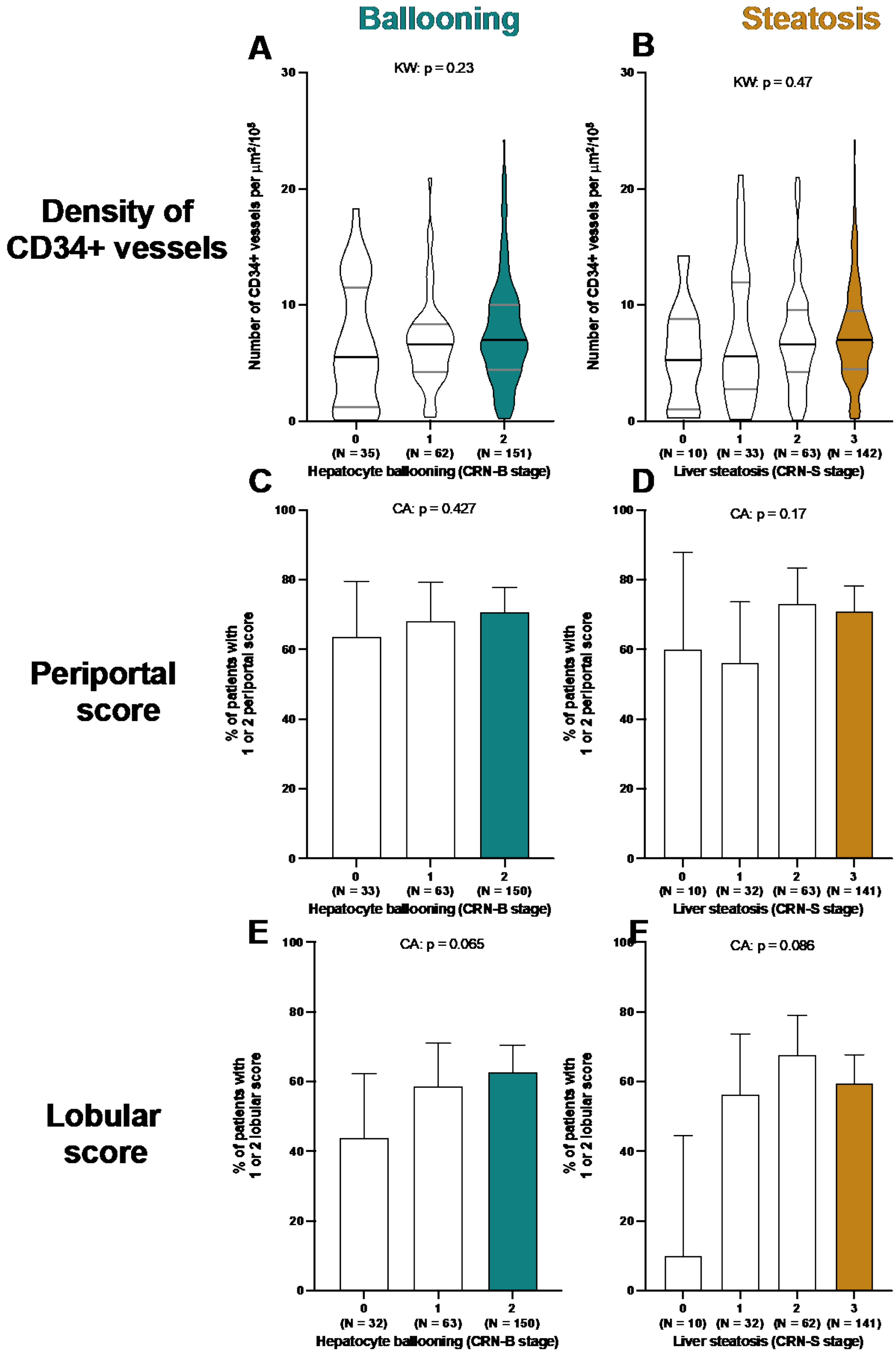

Density of CD34 positive vessels, periportal score and lobular score were available in 248, 246 and 245 patients, respectively, as detailed in Fig. S3. Baseline density of CD34 positive vessels is displayed according to ballooning (CRN-B grade) **(A)** and according to steatosis (CRN-S grade) **(B)**. Percentage of patients with periportal score for CD34 staining of 1 or 2 is displayed according to ballooning (CRN-B grade) **(C)** and according to steatosis (CRN-S grade) **(D)**. Percentage of patients with lobular score for CD34 staining of 1 or 2 is displayed according to ballooning (CRN-B grade) **(E)** and according to steatosis (CRN-S grade) **(F)**.
CRN, Clinical Research Network; KW, Kruskal-Wallis; CA, Cochran-Armitage. For violin plots, the bars represent the median ± IQR, otherwise bars represent 95% confidence intervals.

Fig. S6. Liver over total body weight ratio in MCDD model.

Male Wistar Han rats of 8 weeks old were either fed a chow diet (CD) or a methionine-choline-deficient diet (MCDD) for 4 weeks and simultaneously treated with either placebo, fenofibrate (30 mg/kg), GW501516 (10 mg/kg), rosiglitazone (5 mg/kg) or lanifibranor (100 mg/kg) daily QD via oral gavage. After 4 weeks of diet and treatment in the MCDD-fed groups the liver/total body weight ratio was significantly increased compared to their healthy counterparts, except for fenofibrate, because the liver weight of CD rats treated with fenofibrate is increased (a well-known PPAR-α effect). This further implies that the weight increase in the MCDD group treated with fenofibrate is mainly a compound effect rather than steatosis. n = 5-8/group per experiment. Pooled data from all experiments (n = 37-60/group) were analysed using Kruskal-Wallis followed by Dunn test and presented as median (IQR) with ns (not significant), **: p<0.01; ***: p<0.001; ****: p<0.0001.

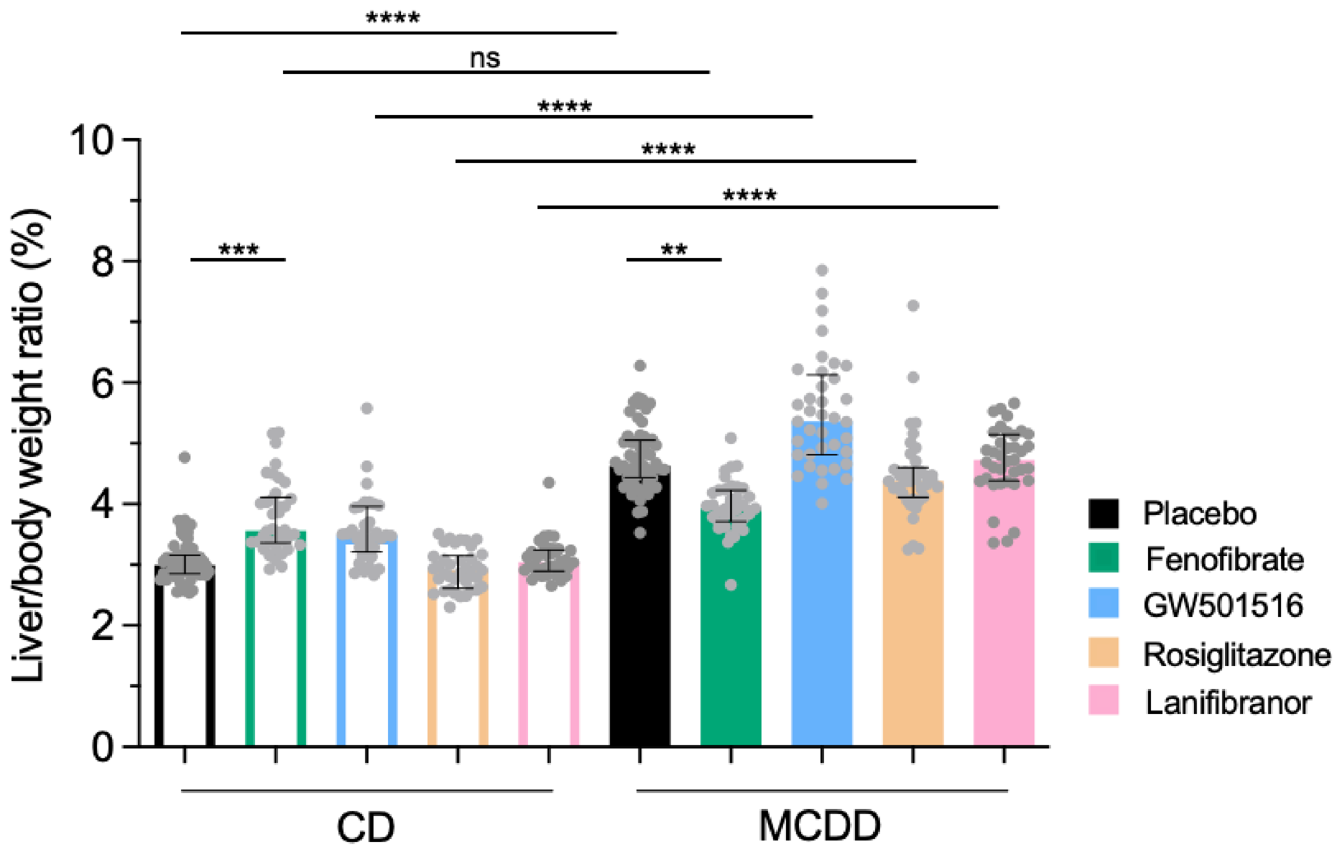

Fig. S7. Histological assessment of fibrosis in histological liver sections of early MASLD.

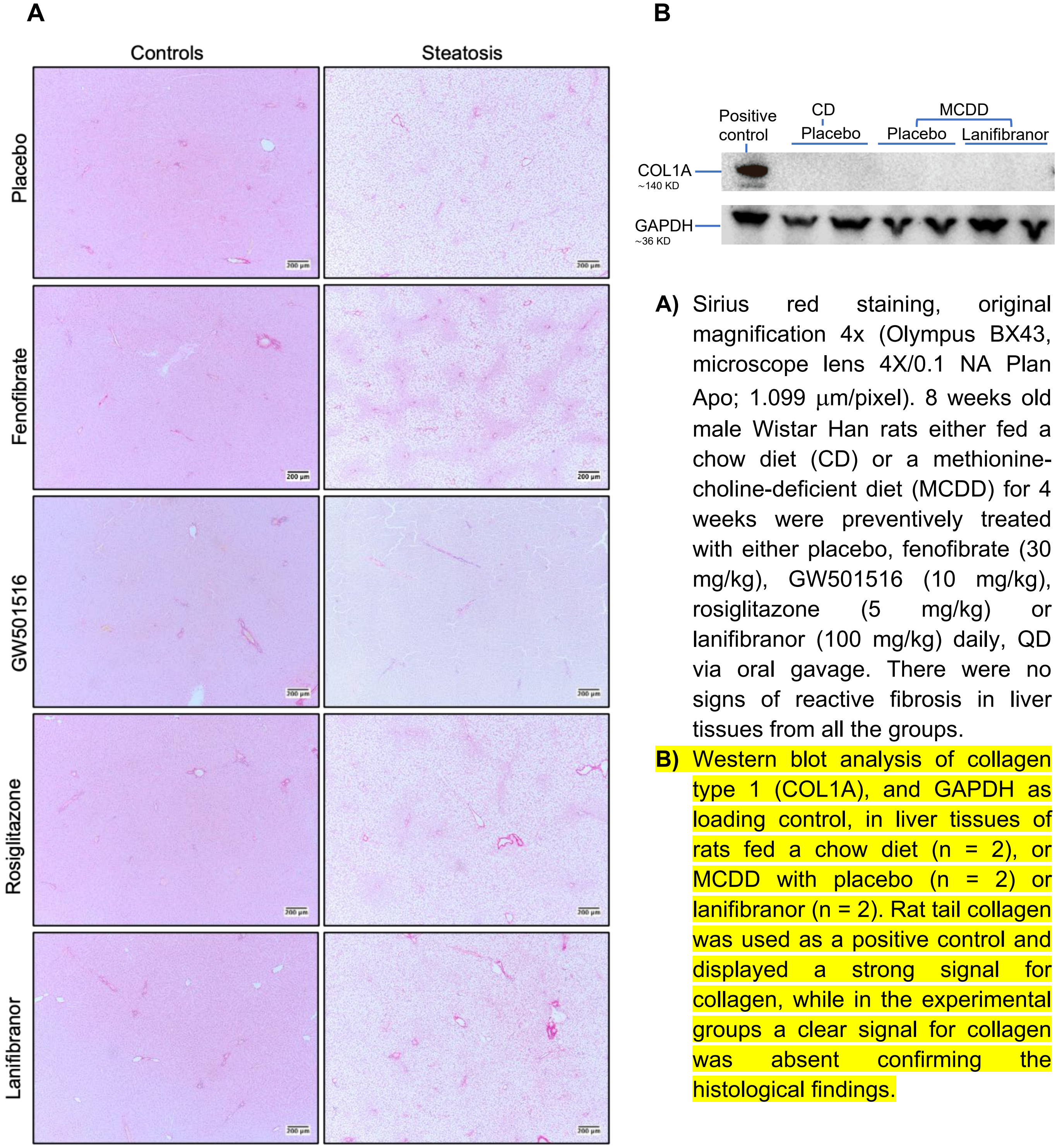

A) Sirius red staining, original magnification 4x (Olympus BX43, microscope lens 4X/0.1 NA Plan Apo; 1.099 µm/pixel). 8 weeks old male Wistar Han rats either fed a chow diet (CD) or a methionine-choline-deficient diet (MCDD) for 4 weeks were preventively treated with either placebo, fenofibrate (30 mg/kg), GW501516 (10 mg/kg), rosiglitazone (5 mg/kg) or lanifibranor (100 mg/kg) daily, QD via oral gavage. There were no signs of reactive fibrosis in liver tissues from all the groups.

B) Western blot analysis of collagen type 1 (COL1A), and GAPDH as loading control, in liver tissues of rats fed a chow diet (n = 2), or MCDD with placebo (n = 2) or lanifibranor (n = 2). Rat tail collagen was used as a positive control and displayed a strong signal for collagen, while in the experimental groups a clear signal for collagen was absent confirming the histological findings.

Fig. S8. Assessment of steatosis in early MASLD.

A) Images of haematoxylin and eosin (H-E) stained liver tissue sections (Olympus BX43, microscope lens 10X/0.45 NA Plan Apo; resolution 1 pixel = 0.442 μm). **B)** Steatosis quantification defined as macrovesicular fat droplets fraction per area (%). Male Wistar Han rats (n = 6/group) were either fed a chow diet (CD) or a methionine-choline-deficient diet (MCDD) for 4 weeks and preventively treated with either placebo, fenofibrate (30 mg/kg), GW501516 (10 mg/kg), rosiglitazone (5 mg/kg) daily QD via oral gavage. Data were analysed using two-way ANOVA followed by post hoc Tukey and presented as mean ± standard error of the mean with ns: not significant; **: p<0.01.

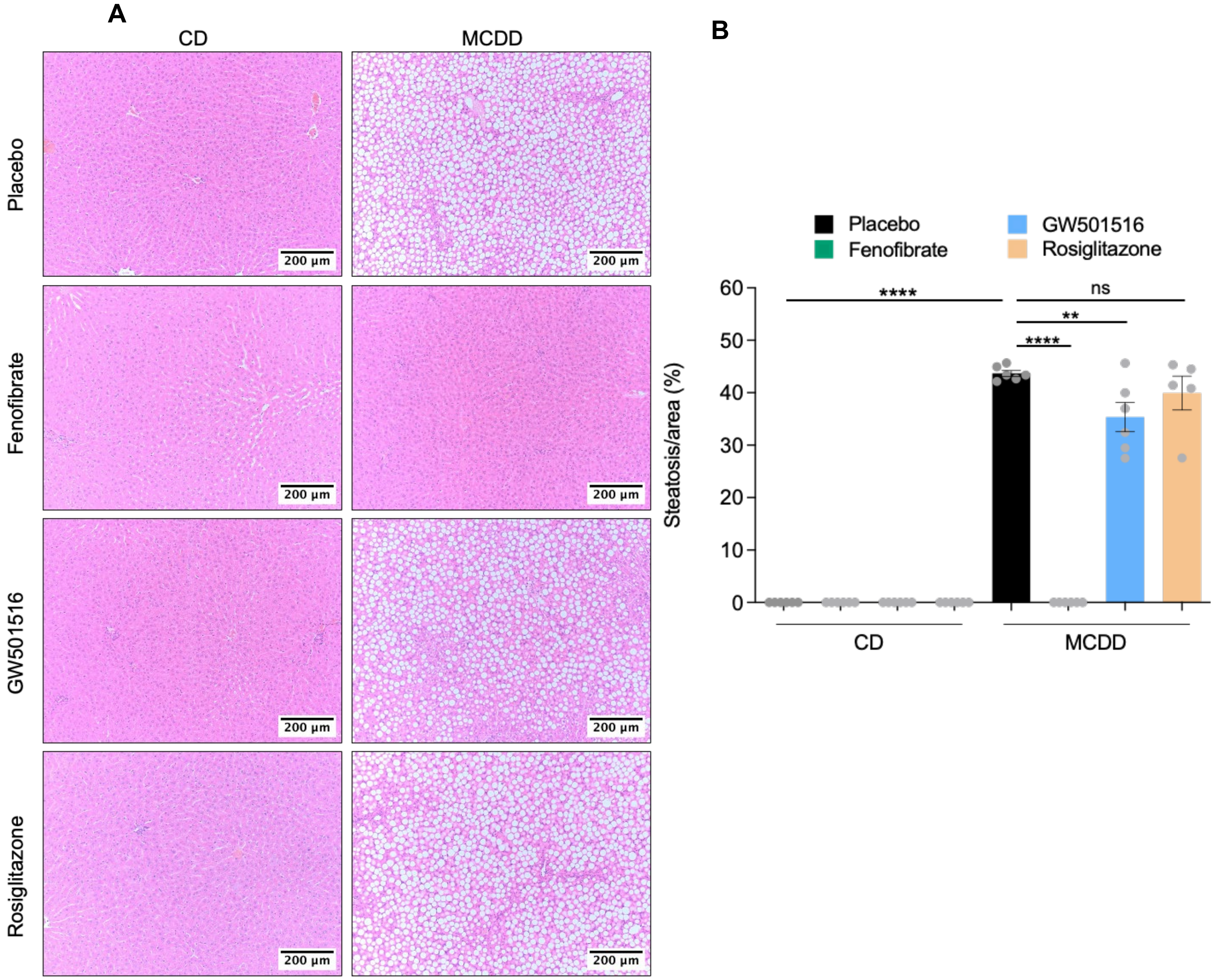

Fig. S9. Histological assessment of liver sections of rats with MASH

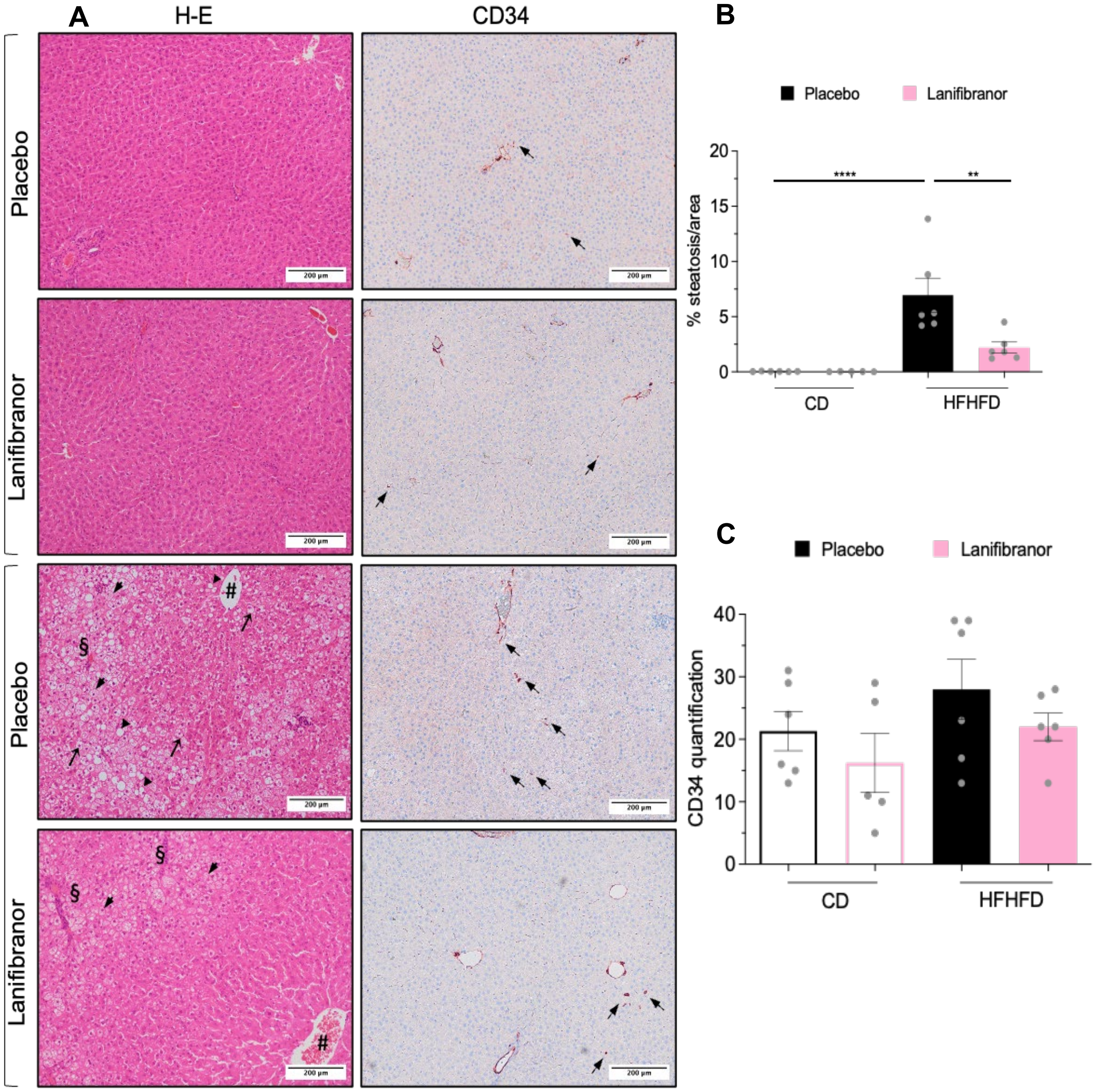

8 weeks old male Zucker fatty rats (n = 6/group) fed a high-fat high-fructose diet (HFHFD) and 8 weeks old male Zucker lean rats (n = 5-6/group) fed a chow diet (CD) were concomitantly treated with either placebo or lanifibranor (100 mg/kg) daily QD via oral gavage during the complete period of 8 weeks of diet. (Olympus BX43, microscope lens 10X/0.45 NA Plan Apo; resolution 1 pixel = 0.442 µm). **A)** Haematoxylin and eosin (H-E), and CD34 stained histological liver sections. In H-E images periportal area is marked with "§". Centrilobular area is marked with "#". Short arrows point microvesicular steatosis, arrow heads show macrovesicular steatosis and thin arrows show ballooning. In CD34 images arrows point CD34 positive spots. **B)** Steatosis quantification defined as fraction of macrovesicular fat droplets per area (%). **C)** Blinded CD34 semi-quantification. Data is presented as mean ± SEM. Two-way ANOVA was performed followed by post hoc Tukey.

Fig. S10. Assessment of fibrosis on Sirius red stained livers of Zucker rats.

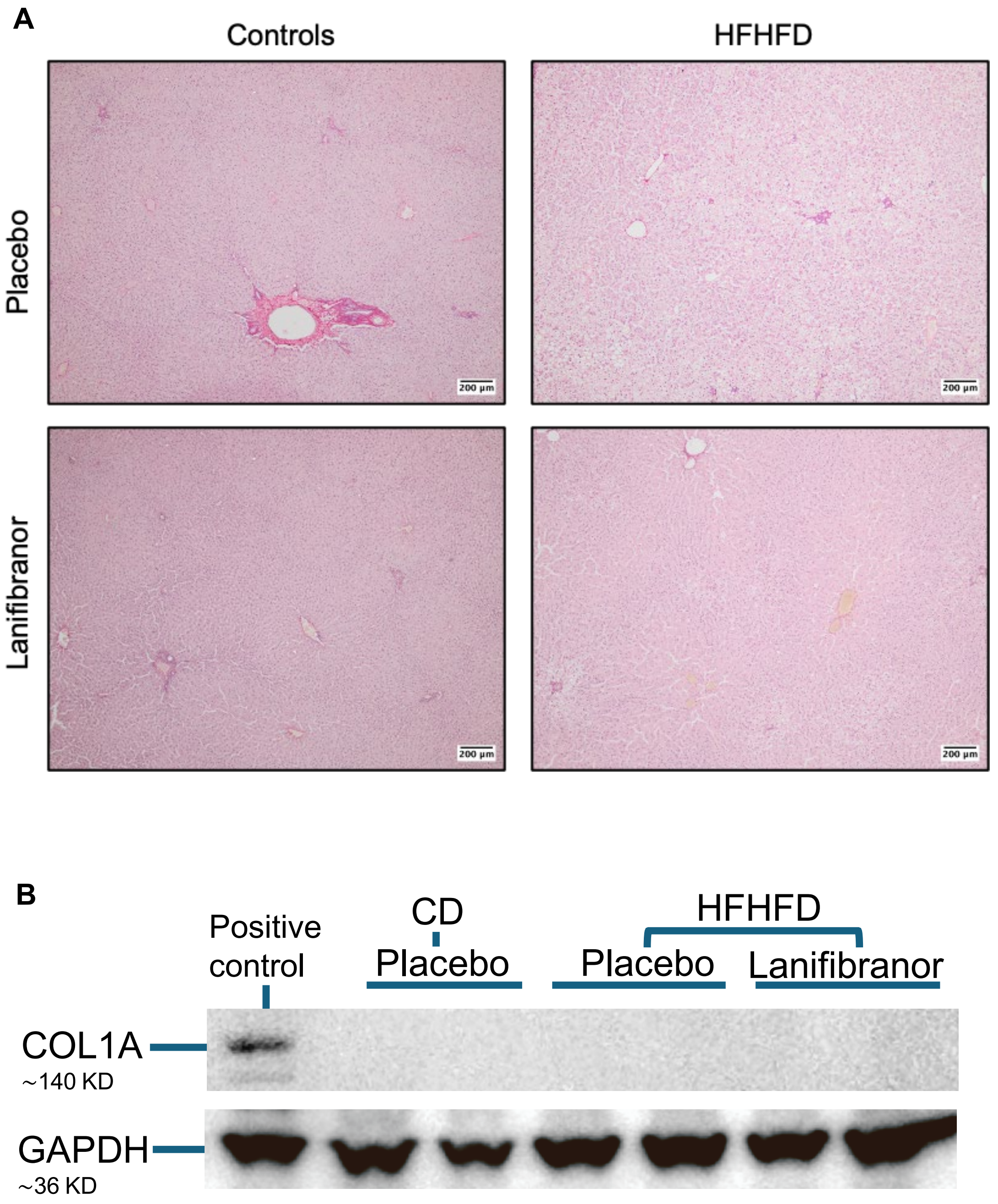

A) 8 weeks old male Zucker fatty rats (n = 6/group) fed a high-fat high-fructose diet (HFHFD) and male Zucker lean rats (n = 6/group) fed a chow diet (CD) were concomitantly treated with either placebo or lanifibranor (100 mg/kg) daily QD via oral gavage during the complete period of 8 weeks of diet. Control livers did not show any abnormalities. In placebo treated HFHFD-fed Zucker rats there was no sign of fibrosis. Original magnification 4x (Olympus BX43, microscope lens 4X/0.1 NA Plan Apo; 1.099 μm/pixel). **B)** Western blot analysis of collagen type 1 (COL1A), and GAPDH as loading control, in liver tissues of rats male Zucker lean rats fed a chow diet (CD) (n = 2), in placebo-treated HFHFD-fed (MASH) rats (n = 2) and lanifibranor-treated HFHFD-fed (MASH) rats (n = 2). Rat tail collagen was used as a positive control and displayed a strong signal for collagen, while in the experimental groups there was no signal for collagen. This is in line with the histological findings.

Fig. S11. Body weight, liver weight and spleen weight evolution in a MASH model following 8 weeks of chow diet or high-fat high-fructose diet.

A) Weight gain, **B)** Liver weight, **C)** Liver over total body weight ratio (%), **D)** Spleen weight. 8 weeks old male Zucker fatty rats fed a high-fat high-fructose diet (HFHFD) and 8 weeks old male Zucker lean rats fed a chow diet (CD) were preventively treated with either placebo or lanifibranor (100 mg/kg) daily QD via oral gavage during the complete period of 8 weeks of diet. n = 5-8/group per experiment. Pooled data (n = 28-30/group) were analysed using two-way ANOVA (for A & D) followed by post hoc Tukey and presented as mean ± standard error of the mean or Kruskal-Wallis (B & C) followed by Dunn test and presented as median (IQR). ns: not significant; *: p<0.05; **: p<0.01; ****: p<0.0001. Spleen weight was only determined in one experiment, therefore, no pooled data available.

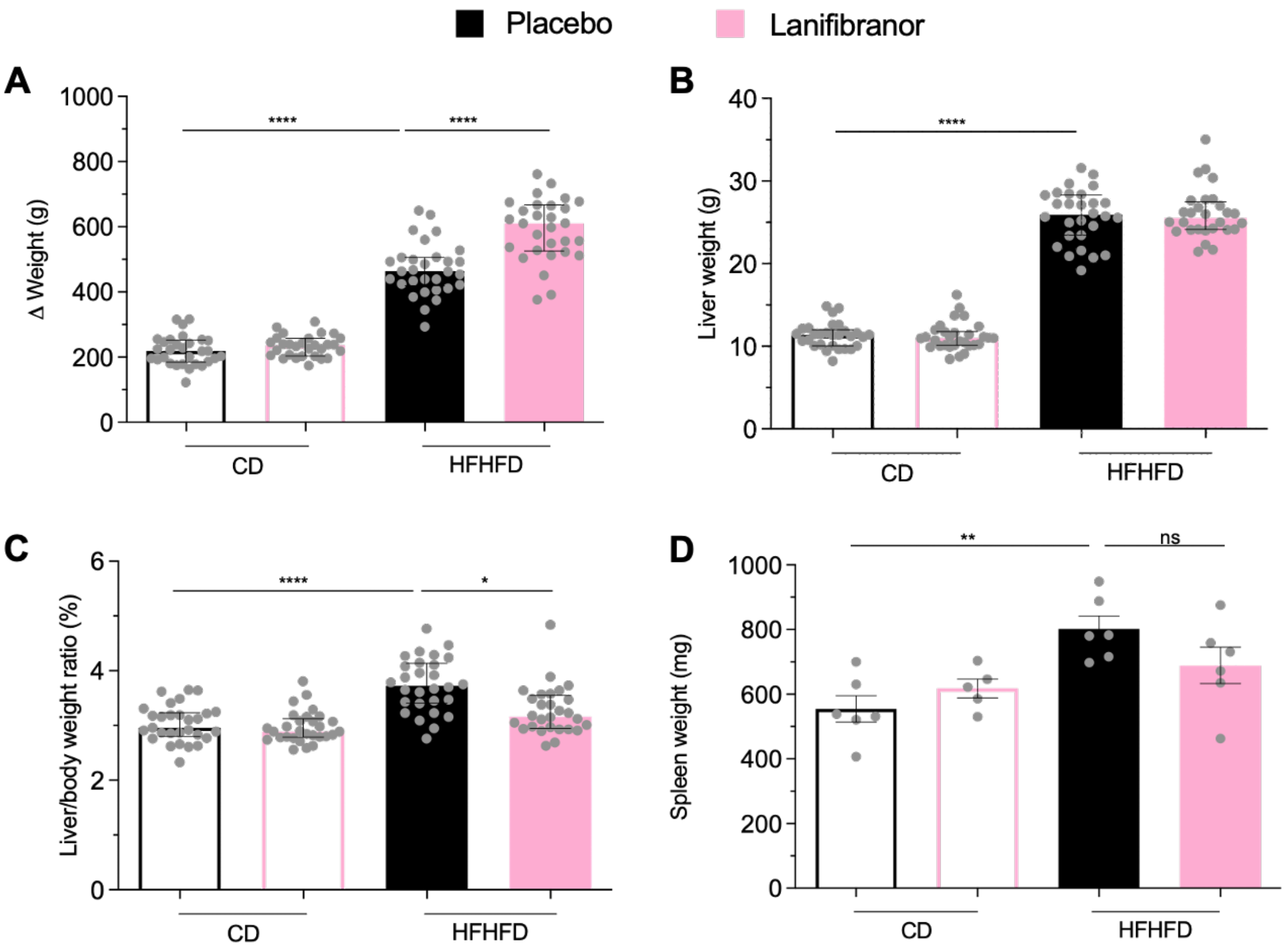

Fig. S12. Immunohistology of liver sections.

A) Images of CD34 stained liver tissue sections (Olympus BX43, microscope lens 10X/0.45 NA Plan Apo; resolution 1 pixel = 0.442 μm). **B)** Blinded CD34 semi-quantification. 8 weeks old male Wistar Han rats fed chow- (CD) or methionine - choline - deficient diet (MCDD) for 4 weeks were preventively treated with either placebo, fenofibrate (30 mg/kg), GW501516 (10 mg/kg), rosiglitazone (5 mg/kg) or lanifibranor (100 mg/kg). Data presented as mean ± SEM (n = 5-6/group). Two-way ANOVA was performed followed by post hoc Tukey with ns: not significant; **: p<0.01; ***: p<0.001; ****: p<0.0001. For clarity only the comparisons with MCDD-placebo are shown. Arrows point CD34 positive staining.

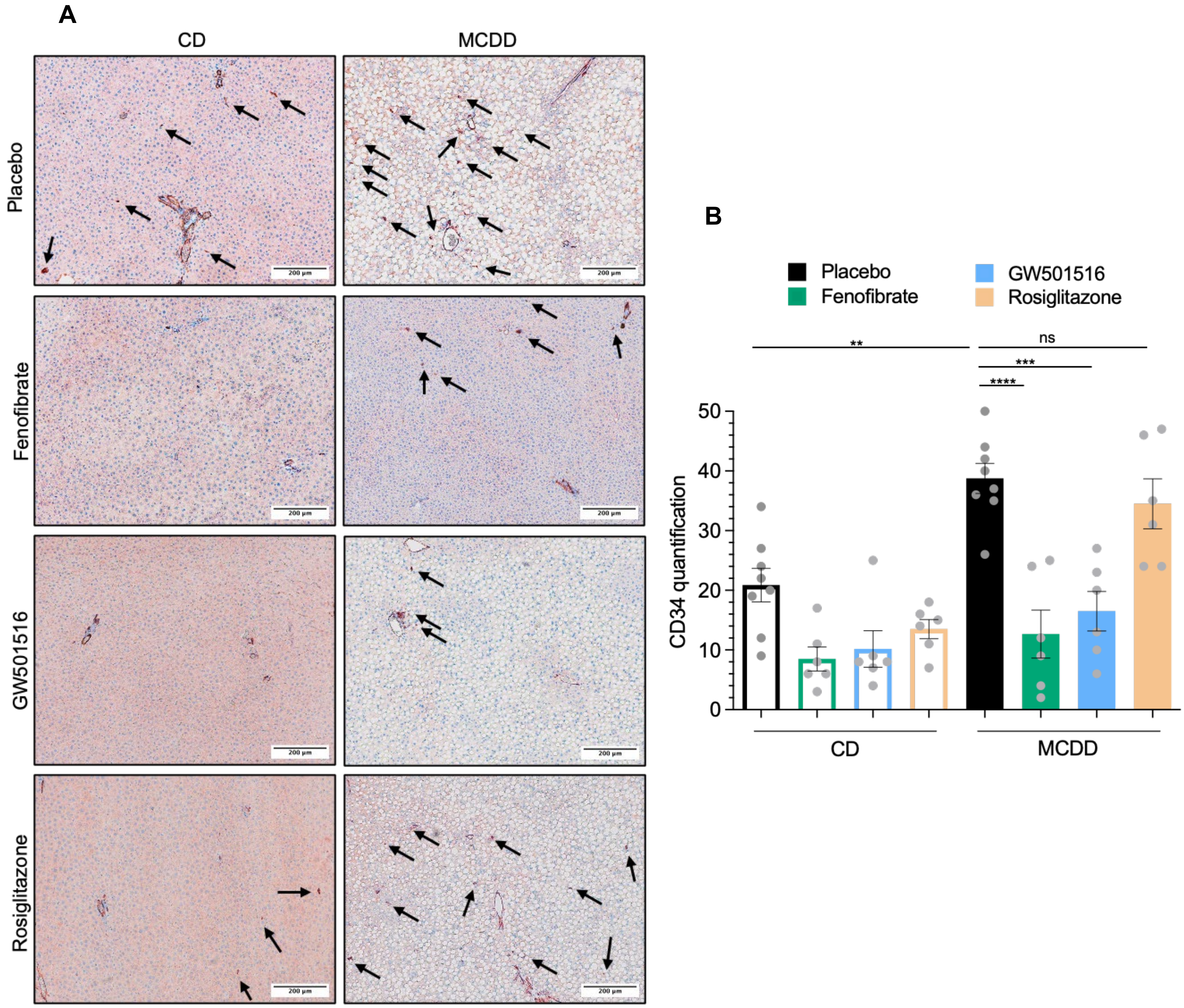

Fig. S13. In vivo portal venous pressure in early MASLD.
Male Wistar Han rats of 8 weeks old were either fed a chow diet (CD) or a methionine-choline-deficient diet (MCDD) for 4 weeks and simultaneously treated with either placebo, fenofibrate (30 mg/kg), GW501516 (10 mg/kg), rosiglitazone (5 mg/kg) or lanifibranor (100 mg/kg) daily QD via oral gavage. n = 7-8/ group per experiment. Pooled data (n = 24-60/group) were analysed using Kruskal-Wallis followed by Dunn test and presented as median (IQR) with ns: not significant; *: p<0.05; ****: p<0.0001. For clarity only the comparisons with MCDD-placebo are shown. PVP, portal venous pressure.

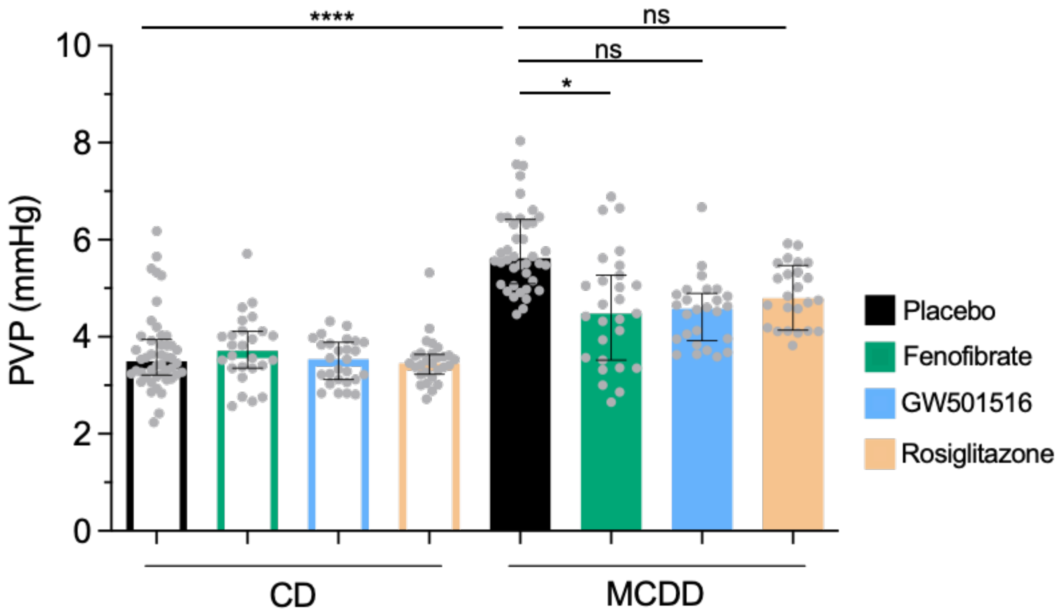

Fig. S14. *In vivo* arterial pressures and pulse rates in early MASLD.
Male Wistar Han rats of 8 weeks old were either fed a chow diet (CD) or a methionine-choline-deficient diet (MCDD) for 4 weeks and simultaneously treated with either placebo, fenofibrate (30 mg/kg), GW501516 (10 mg/kg), rosiglitazone (5 mg/kg) or lanifibranor (100 mg/kg) daily QD via oral gavage. n = 6-8/group per experiment. Pooled data (n = 25-43/group) were analysed using Kruskal-Wallis followed by Dunn test and presented as median (IQR) with **: $p<0.01$; ***: $p<0.001$; ****: $p<0.0001$. For clarity only the relevant comparisons with MCDD-placebo are shown. PVP, portal venous pressure; MABP, mean arterial blood pressure; SBP, systolic blood pressure; DBP, diastolic blood pressure; BPM, beats per minute.

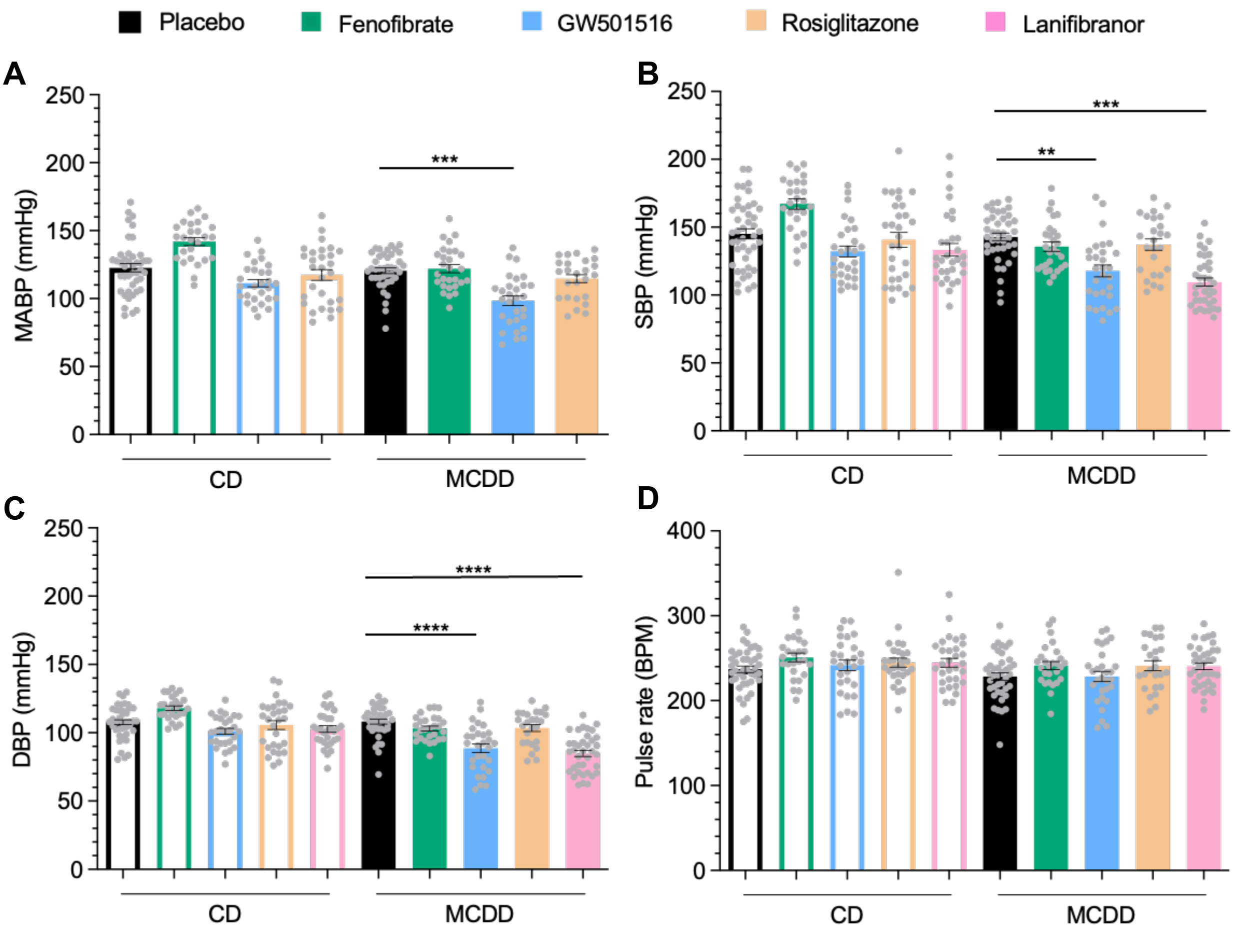

Fig. S15. *In vivo* arterial pressures and pulse rates in Zucker rats.
8 weeks old male Zucker fatty rats fed a high-fat high-fructose (HFHFD) diet and 8 weeks old male Zucker lean rats fed a chow diet (CD) were preventively treated with either placebo or lanifibranor (100 mg/kg) daily QD via oral gavage during the complete period of 8 weeks of diet. n = 6-8/group per experiment. Pooled data (n = 16-19/group) were analysed using two-way ANOVA followed by post hoc Tukey and presented as mean ± standard error of the mean. ****: p<0.0001. For clarity only the relevant comparisons with MCDD-placebo are shown. PVP, portal venous pressure; MABP, mean arterial blood pressure; SBP, systolic blood pressure; DBP, diastolic blood pressure; BPM, beats per minute.

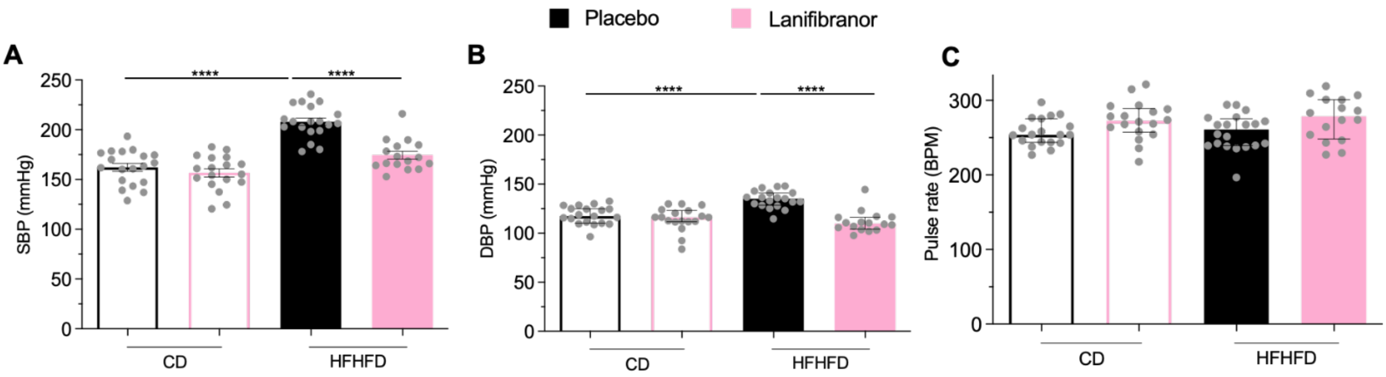

Fig. S16. ***In situ ex vivo*** **liver perfusion: transhepatic pressure gradient for assessment of intrahepatic vascular resistance in early MASLD.**

Male Wistar Han rats of 8 weeks old were either fed a chow diet (CD) or a methionine-choline-deficient diet (MCDD) for 4 weeks and simultaneously treated with either **A)** placebo, **B)** fenofibrate (30 mg/kg), **C)** GW501516 (10 mg/kg) or **D)** rosiglitazone (5 mg/kg) daily QD via oral gavage during the complete 4 weeks of diet. n = 7-8/group. Data presented as mean ± SEM. The THPG data were analysed using a generalised estimating equation model followed by least significant difference post hoc testing with *: p<0.05; ***: p<0.001; ****: p<0.0001. Statistics on graph shown for most important comparisons only (MCDD + treatment vs. MCDD + placebo). THPG, transhepatic pressure gradient; min, minute.

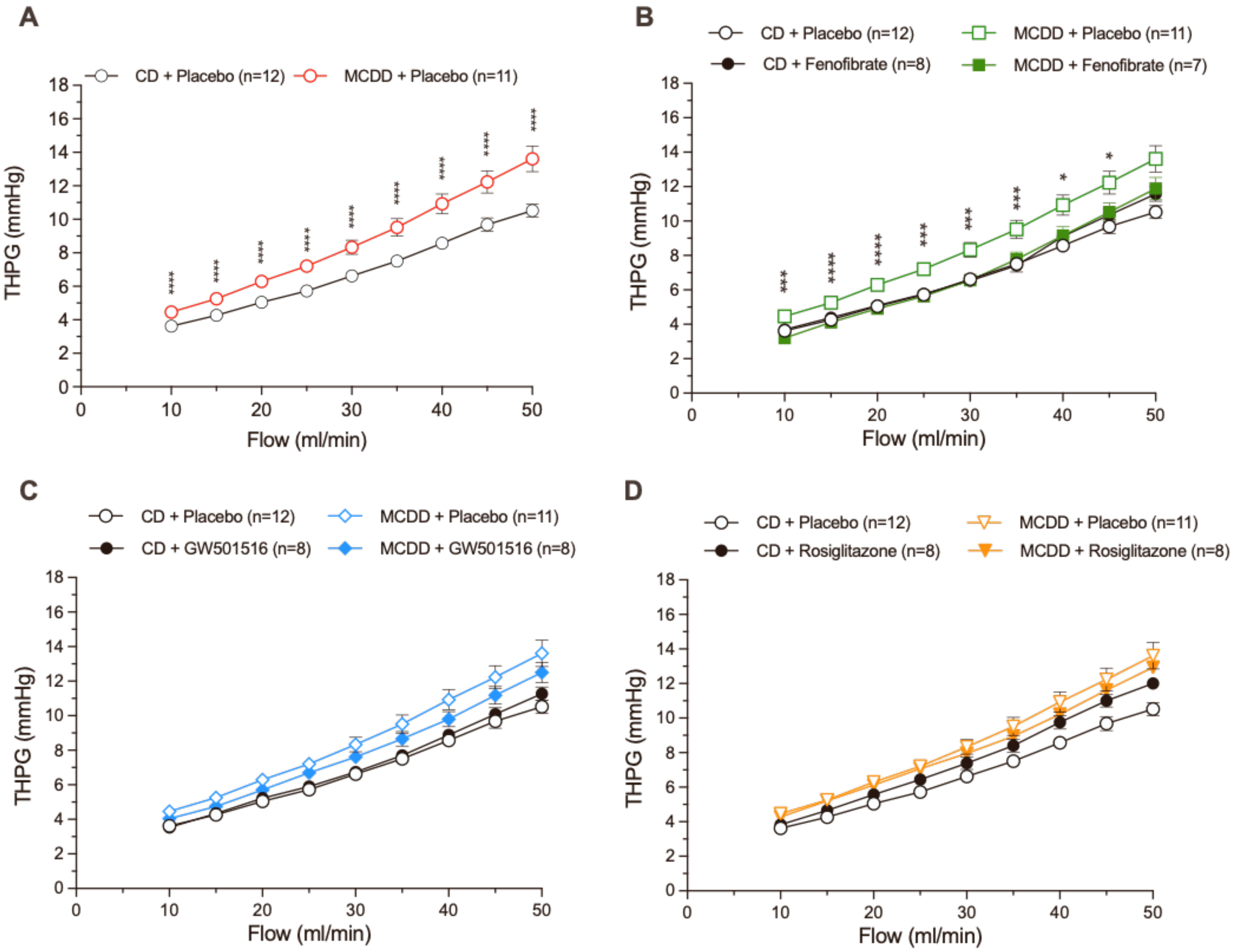

Fig. S17. *In situ ex vivo* liver perfusion: relative change of transhepatic pressure gradient after dose-response measurement with endothelin-1.

Male Wistar Han rats of 8 weeks old were either fed a chow diet (CD) or a methionine-choline-deficient diet (MCDD) for 4 weeks and simultaneously treated with either **A)** placebo, **B)** fenofibrate (30 mg/kg), **C)** GW501516 (10 mg/kg) or **D)** rosiglitazone (5 mg/kg) daily, QD via oral gavage. n = 7-8/group). Data presented as mean ± SEM. The THPG data were analysed using a generalised estimating equation model followed by least significant difference post hoc testing. Statistics on graph show with * for MCDD + treatment vs MCDD + placebo and with ° for CD + treatment vs. CD + placebo. *: $p<0.05$; **: $p<0.01$; ***: $p<0.001$; ****: $p<0.0001$. °: $p<0.05$; °°: $p<0.01$; °°°°: $p<0.0001$. THPG, transhepatic pressure gradient; ET-1, endothelin-1; (Log M), logarithmic concentration in mol/Liter.

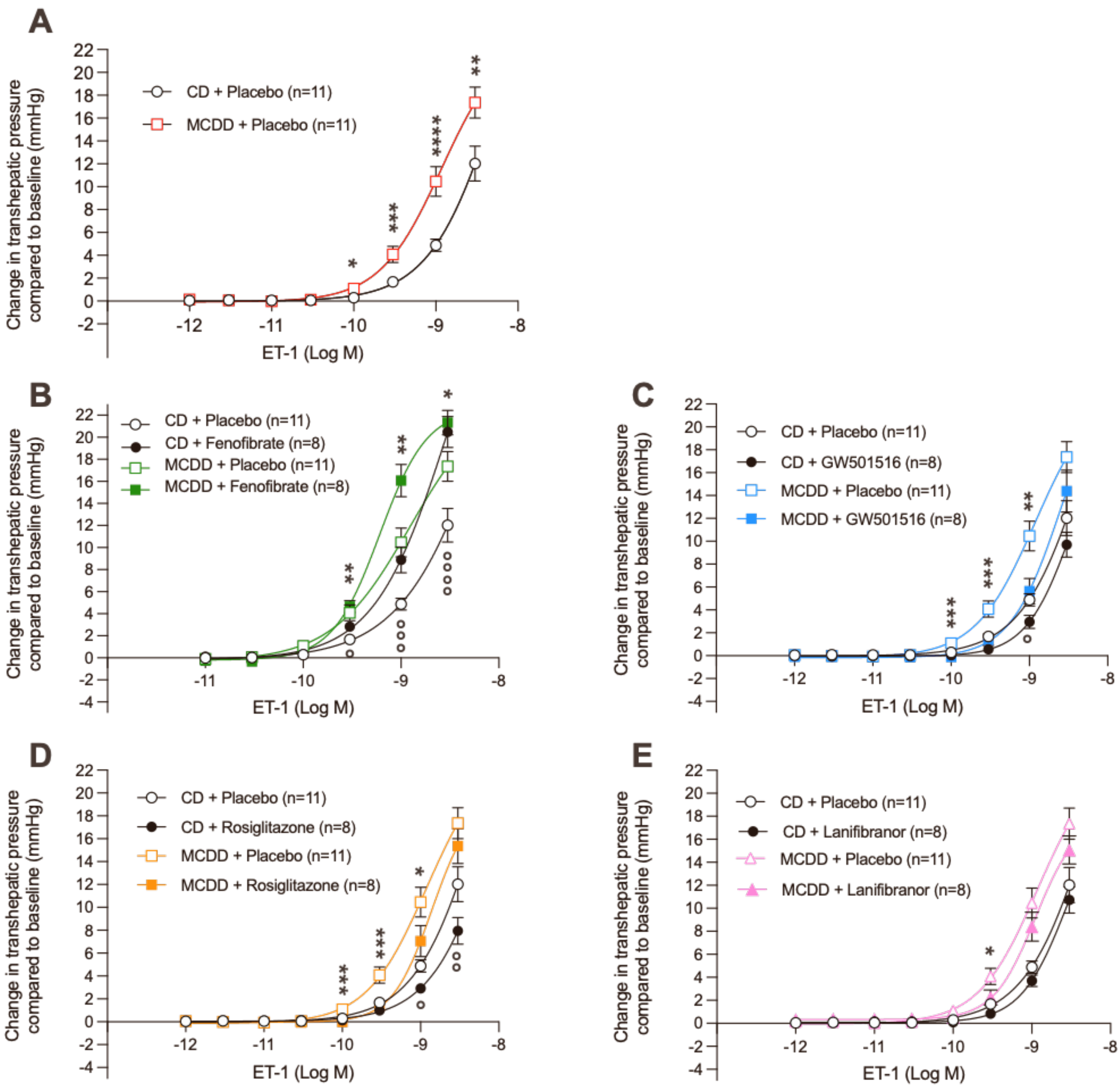

Fig. S18. *In situ ex vivo* liver perfusion in early MASLD: relative change of transhepatic pressure gradient after dose-response measurement with methoxamine.

Male Wistar Han rats of 8 weeks old were either fed a chow diet (CD) or a methionine-choline-deficient diet (MCDD) for 4 weeks and were simultaneously treated with either **A)** placebo, **B)** fenofibrate (30 mg/kg), **C)** GW501516 (10 mg/kg) or **D)** rosiglitazone (5 mg/kg) daily QD via oral gavage. n = 7-11/group). Data is presented as mean ± SEM. The THPG data were analysed using a generalised estimating equation model followed by least significant difference post hoc testing with *: p<0.05; **: p<0.01; ***: p<0.001; ****: p<0.0001. Statistics on graph shown for most important comparisons only (MCDD + treatment vs. MCDD + placebo). THPG, transhepatic pressure gradient; Mx, methoxamine; (Log M), logarithmic concentration in mol/Liter.

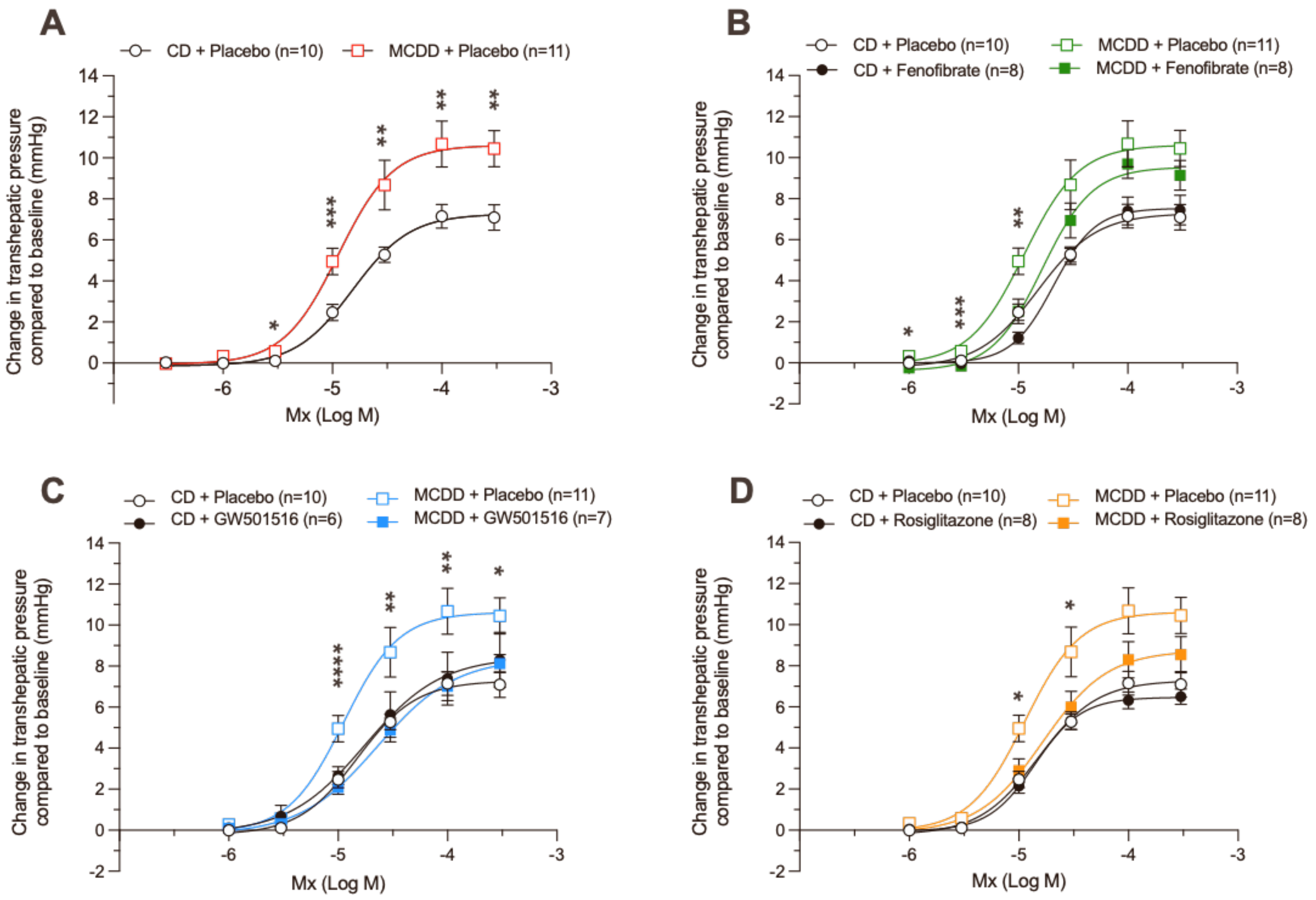

Fig. S19. *In situ ex vivo* liver perfusion in early MASLD: % vascular relaxation with increasing doses of acetylcholine, after methoxamine preconstriction.
Male Wistar Han rats of 8 weeks old were either fed a chow diet (CD) or a methionine-choline-deficient diet (MCDD) for 4 weeks and were simultaneously treated with either **A)** placebo, **B)** fenofibrate (30 mg/kg), **C)** GW501516 (10 mg/kg) or **D)** rosiglitazone (5 mg/kg) daily QD via oral gavage. n = 7-13/group). Data presented as mean ± SEM. The vascular relaxation data were analysed using a generalised estimating equation model followed by least significant difference post hoc testing with *: $p<0.05$; **: $p<0.01$; ****: $p<0.0001$. Statistics on graph shown for most important comparisons only (MCDD + treatment vs MCDD + placebo). ACh, acetylcholine; (Log M), logarithmic concentration in mol/Liter.

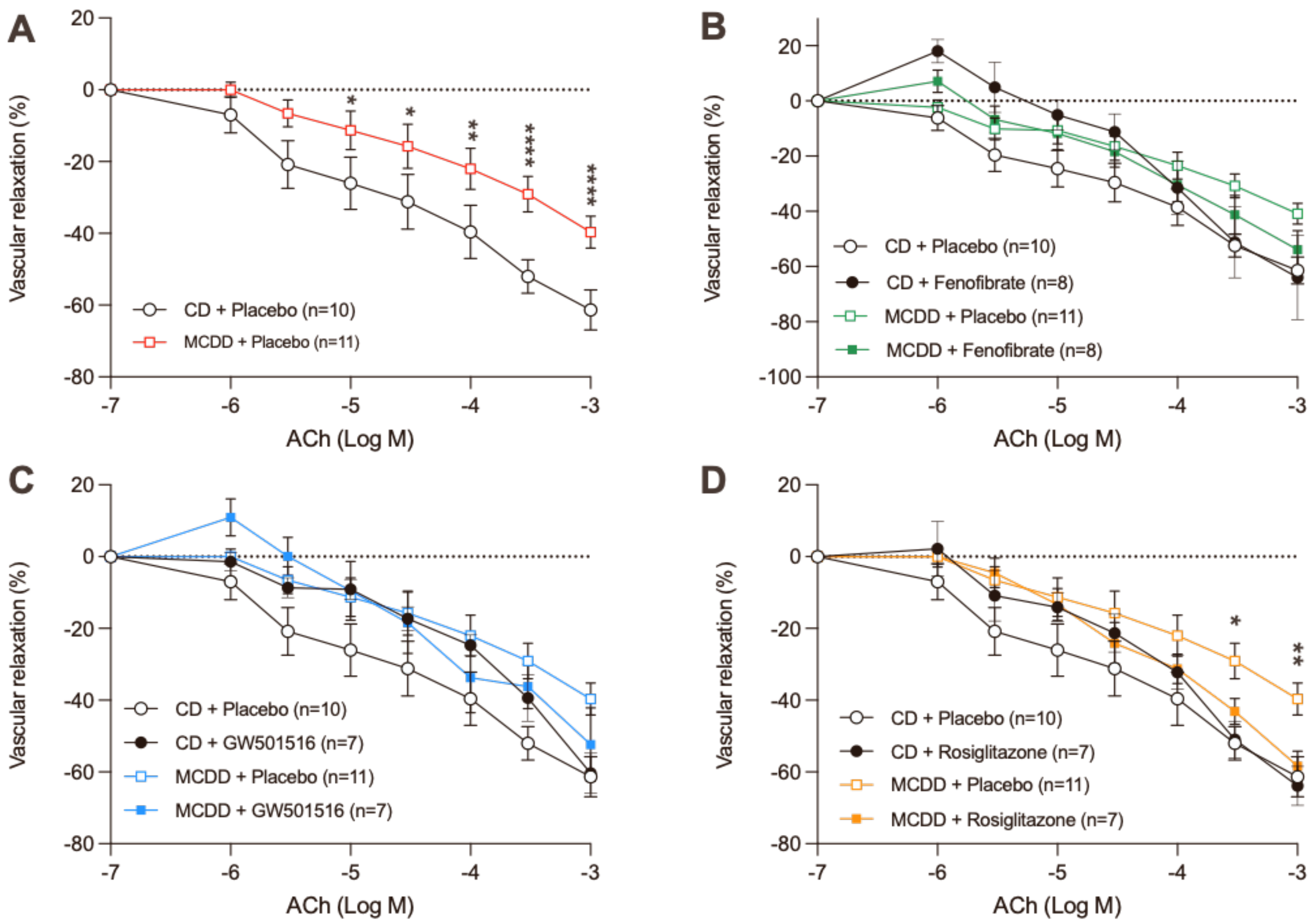

Fig. S20. Qualitative assessment of hepatic vascular corrosion casts acquired by scanning electron microscopy.

A) CD + placebo, **B)** MCDD + placebo, **C)** MCDD + fenofibrate, **D)** MCDD + GW501516**, E)** MCDD + rosiglitazone, **F)** MCDD + lanifibranor)**.** 8 weeks old male Wistar Han rats either fed a chow diet (CD) or a methionine-choline-deficient diet (MCDD) for 4 weeks were preventively treated with either placebo, fenofibrate (30 mg/kg), GW501516 (10 mg/kg), rosiglitazone (5 mg/kg) or lanifibranor (100 mg/kg) daily, QD via oral gavage. n = 3/group. Magnification 300x. Voltage: 20.0 kV.

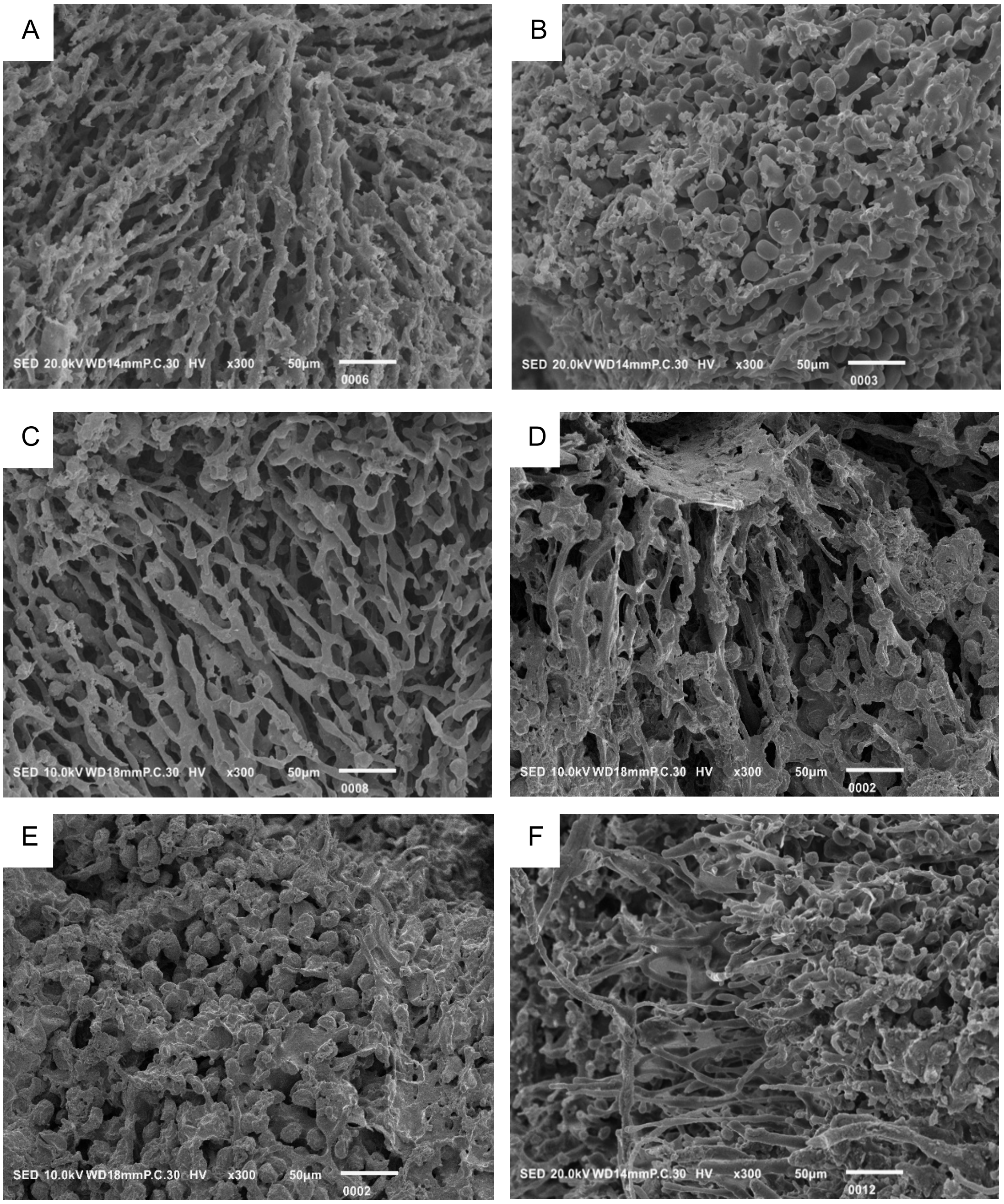

Supplementary tables

Table S1. List of Independent Ethics Committee / Institutional Review Board

Country	CEC or LEC for Site	Ethical Committees (or IRB)	Chair Person
Australia	CEC - all Sites	Monash Health Human Research EthicsCommittee (EC00382): Research Support ServicesMonash Health, Level 2, Block 1, Monash Medical Centre 246 Clayton Road, Clayton, Victoria, 3168. Email: research@monashhealth.org	Deborah DELL (Manager)
Australia	Site 1201	Research Support ServicesMonash Health, Level 2, Block 1, Monash Medical Centre 246 Clayton Road, Clayton, Victoria, 3168. Email: michael.kios@monashhealth.org	Deborah DELL (Manager)
Australia	Site 1202	NALHN Research Governance OfficerResearch Secretariat Northern Adelaide Local Health NetworkLyell McEwin Hospital Clinical Trials Unit, Level 2 Haydown Road Elizabeth Vale SA 5112 Email: healthnalhnrgo@sa.gov.au	Lorraine CICHON
Australia	Site 1203	Research Services Research GovernanceOfficer Executive Administration Lower GroundFloor Dr. James Mayne Building Royal Brisbane and Women's HospitalCampus Herston Qld 4029 Generic Email: RBWH-RGO@health.qld.gov.au www.health.qld.gov.au/metronorth	Janani Balakrishnan MCOM
Australia	Site 1204	Research Governance Officer Southern Adelaide Local Health Network Flinders Medical Centre, Bedford Park SA5042 (WARD 6C Room 6A219) Email: karen.saxty@sa.gov.au http://www.sahealth.sa.gov.au/SALHNresearch	Karen SAXTY BHlthSc (Hons)
Australia	Site 1205	Research Governance OfficerLevel 2, Education Building Fiona Stanley Hospital 14 Barry Marsh ParadeMurdoch, WA 6150 Email: SMHS.RGO@health.wa.gov.au	Cathy GARTNER
Austria	CEC - Site 1001 (no patient screened in Site 1002)	Ethikkommission der MedizinischenUniversität Wien, Borschkegasse 8b/E06,1090 Wien, Österreich Tel: +43(0)1 404 00-21470; Fax: +43(0)1404 00-16900 Email: ethik-kom@meduniwien.ac.at http://ethikkommission meduniwien.ac.at/	Jürgen ZEZULA
Belgium	CEC - Site 0001	Comité voor Medische Ethiek UZA, Wilrijkstraat 10, B-2650 Edegem Tel +32 3 821 30 00 Email: EthischComite@uza.bewww.uza.be	Peter MICHIELSEN
Belgium	LEC - Site 0002	Comité d'Ethique hospitalo-facultaire Promenade de l'Alma 51 bte B1.43.031200 Bruxelles Email: commission.ethique-saintluc@uclouvain.be Tel: 02/ 764 55 14 https://www.saintluc.be/recherche/comite-ethique-accueil.php	Jean-Marie MALOTEAUX
Belgium	LEC - Site 0003	Comité d'éthique Hospitalo-facultaireErasme-ULB Route de Lennik, 8081070 Bruxelles Tel: +32 (0) 2 555 37 07; Fax: +32 (0) 2 555 46 20 Email: Comite.ethique@erasme.ulb.ac.behttp://www.hopitalerasme.be/ethique	Prof. J.-M. BOEYNAEMs
Belgium	LEC - Site 0004	Commissie voor Medische ethiekUniversitair Ziekenhuis Gent, C. Heymanslaan 10,9000 Gent Tel: +32 9 332 22 66 Email: ethisch.comite@uzgent.be https://www.uzgent.be/nl/home/Paginas/home.aspx	Prof. Dr. D.MATTHYS
Belgium	LEC - Site 0005	Comité Medische Ethiek Ziekenhuis Oost-Limburg Secretariaat Comité Medische EthiekSchiepse Bos 6 3600 GENK Tel: 089 32 15 09; Fax: 089 32 79 00 (clear mention CME) Email: ec.submission@zol.be https://www.zol.be/comite-medische-ethiek	Patrick NOYENS
Bulgaria	CEC	Ethics Committee for Clinical Trials, 8, Damyan Gruev str., 1303 Sofia, Bulgaria	Stefan DIMITROV, MD
Bulgaria	LEC - Site 1501	Local Ethics Committee at UMHAT "Sv. Ivan Rilski" EAD, 15 Akad. I. Geshov Blvd.,1431 Sofia, Bulgaria, Tel: +35929523554	Assoc. Prof. ZHELEV
Bulgaria	LEC - Site 1502	Local Ethics Committee at UMHAT "Tsaritsa Yoanna - ISUL" EAD, 8, ByaloMore Str., 1527 Sofia, Bulgaria, Tel: +35929432170	Prof. Boryana DELIISKA

Bulgaria	LEC - Site 1503	Local Ethics Committee at "Acibadem CityClinic MHAT Tokuda"EAD, 51B NikolaVaptzarov Blvd, 1407 Sofia, Bulgaria,<br>Tel.: +35924034000	Rozalina BALABANSKA, MD
Bulgaria	LEC - Site 1504	Local Ethics Committee at UMHAT "SvetaAnna" - Sofia AD, 1, Dimitar Mollov Str., 1750 Sofia, Bulgaria	Nikolay DOBREV, MD
Bulgaria	LEC - Site 1505	Local Ethics Committee at Military Medical Academy - MHAT - Sofia, 3 Georgi Sofiyski Str., 1606 Sofia, Bulgaria	Col. Prof. Ventsislav MUTAFCHIISKI
Bulgaria	LEC - Site 1506	Local Ethics Committee at DCC "AlexanDr.ovska" EOOD, 1 Georgi Sofyiskistr., 1431 Sofia, Bulgaria,<br>Tel: +35929230815	Nataliya TEMELKOVA, MD
Bulgaria	LEC - Site 1507	Acibadem City Clinic University Hospital EOOD, UIC 202139132, with address of business 127 Okolovrasten pat Str., Sofia andcorresponding address of the Second clinical<br>base: 66A Tsarigradsko shosse Blvd., 1784 Sofia, Bulgaria.	Dr Victoria Sasheva Dimitrova Biliana GENOVA*
Canada	Site 1301 and 1306	McGill University Health Centre (MUHC)REB<br>5100, boul. de Maisonneuve Ouest, 5th floorMontréal, Québec, H4A 3T2 Tel: 514-934-1934, ext-71461<br>Email: renaud.boulanger@muhc.mcgill.ca	PAGE Sonya; MANIATIS Thomas; LEBOUCH É Bertrand; BOULANGER Renaud; CANTINI Franca
Canada	Site 1302	Conjoint Health Research Ethics board(CHREB) Research Services Office<br>2500 University Dr.ive NW, Calgary, AB,T2N1N4 Tel: (403) 220-7990; Fax: (403) 289-0693<br>Email: chreb@ucalgary.ca	STACEY A. Page, PhD
Canada	Site 1303	RESEARCH ETHICS OFFICE<br>HEALTH RESEARCH ETHICS BOARD<br>308 Campus Tower, 8625 – 112 St Edmonton, Alberta, Canada T6G IK8 Tel: 780.492.0459; Fax: 780.492.9429<br>Email: reoffice@ualberta.cawww.reo.ualberta.ca	Dr. Shane KIMBER<br>Dr. Glen PEARSON<br>Dr. Don MORRIS H<br>Dr. Robert RENNIE<br>Dr. Anthony JOYCE
Canada	Site 1304	Western University<br>Health Science Research Ethics Board(HSREB)<br>Room 5150 Support Services Building, 1393Western Road<br>London, Ontario, Canada, N6G I G9<br>Tel: 519-661-2161<br>Email: ethics@uwo.ca	GILBERT, Joseph MD (Jones, Philip MD Matsui, Doreen MD)
Canada	Sites 1305 and 1307	Advarra Institutional Review Board (IRB)–Advancing Better Research, Stacey Neshevich phone 905-841-2257 Email: stacey.neshevich@advarra.com 372 Hollandview Trail, Suite 300, Aurora,ON L4G 0A5 Canada	Sara HARNISH, J.D., ExecutiveChair
Czech Republic	Site 0901 Urbanek	Etické komise<br>Fakultní nemocnice v MotoleV úvalu<br>84 150 06 Praha 5 - MotolCzech Republic<br>Tel.: +420 224 431 195; Fax: +420 224 431 196<br>Email: etickakomise@fnmotol.cz	MUDr. Vratislav ŠMELHAUS
Czech Republic	Site 0902 Sperl	Ethics Committee of IKEM and TNThomayer Hospital<br>Vídeňská 800<br>140 59 Prague 4 - KrčCzech Republic<br>Tel: 26108 3481<br>Email: alena.hruba@ftn.cz	Prof. MUDr. Vladimír STANĚK, CSc.
Czech Republic	Site 0903 Hejda	Research Site s.r.o.<br>Slovanská 27<br>326 00 Plzeň<br>Czech Republic<br>Tel - fax: +420 377 320 027<br>Email: eticka.komise@researchSite.cz	MUDr. Luboš JANŮ Ph.D.
France	CEC - all Sites (No LEC) [Sites 0201 to 0217]	Comité de Protection des Personnes Ouest IIIBat. Vie La Santé – 1er étage porte 101 CHU de Poitiers - 2 rue de la milétrie86021 Poitiers Cedex<br>Tel: 05.49.45.21.57; Fax: 05 .49.46.12.62<br>Email: cpp-ouest3@chu-poitiers.fr	Prof. Denis FRASCA

Germany	CEC - 0801<br>Schattenberg	Ethik-Kommission Landesärztekammer Rheinland-PfalzDeutschhausplatz 3 - 55116 Mainz Postfach 29 26 - 55019 Mainz<br>Tel: 06131 28822-62; Fax: 06131 28822-66<br>Email: wagner@laek-rlp.de<br>Web: http://www.laek-rlp.de/ausschuesse-kommissionen/ethikkommission/	Univ.-Prof. Dr. med. Dipl. Ing.<br>Stephan LETZEL
Germany	LEC - 0802<br>Geier	Ethics Committee of the University ofWürzburg<br>Institute for Pharmacology and ToxicologyVersbacher Str. 9<br>97078 Würzburg<br>Tel: 0931 31 48315; Fax: 0931 31 87520<br>Email: ethikkommission@uni-wuerzburg.de	Prof. Dr. med.<br>R.JAHNS
Germany	LEC - 0803<br>Merle	Ethikkommission HeidelbergAlte Glockengießerei 11/1 69115 Heidelberg Tel: +49 6221 56264-72; Fax: +49 6221 56264-80<br>Email: Marion.Teichmann@med.uni-heidelberg.de<br>http://www.medizinische-fakultaet-hd.uni-heidelberg.de/ethikkommission	Dr. med. Dr.<br>hc Thomas<br>STROWITZKI
Germany	LEC - 0805<br>Trautwein	Ethik-Kommission an der Medizinischen Fakultät der Rheinisch-Westfälischen Technischen Hochschule Aachen (RWTHAachen).<br>Universitätsklinikum AachenPauwelsstraße 30<br>52074 Aachen.<br>Tel: +49 241 80-89963; Fax: +49 241 80-82012<br>Email: ekaachen@ukaachen.de	Günther<br>SCHMALZING
Germany	LEC 0807-<br>Boettler	ETHIK-KOMMISSION<br>Albert-Ludwigs-Universität Freiburg Engelberger Straße 21, 79106 Freiburg Tel: (+49) 0761 270-72500;<br>Fax: (+49) 0761 270-72630<br>Email: ekfr.amg@uniklinik-freiburg.de<br>http://www.ethik-kommission.uniklinik-freiburg.de	Prof. Dr. Rudolf<br>KORINTHENBERG
Germany	LEC - 0808<br>Heinzow	Ethics Commission of the MedicalAssociation Westphalia-Lippe and the Westphalian Wilhelms University ofMünster<br>Gartenstrasse 210 - 214D-48147<br>Münster Tel: +49 (0) 2 51/929 –<br>2460;<br>Fax: +49 (0) 2 51/929 - 2478<br>Email: ethikkommission @ aekwl.de	Univ.-Prof. Dr.<br>med. Wolfgang E.<br>BERDEL
Italy	CEC - 0101<br>Bugianesi	Comitato Etico Interaziendale A.O.U. Cittàdella Salute e della Scienza di Torino/ A.O.Ordine Mauriziano/ASL Città di Torino Corso Bramante 88/90<br>10126 - Torino<br>Tel: 011.633.6820; Fax: 011.633.4171<br>Email: comitatoetico@cittadellasalute.to.it	Dr. Marcello<br>MADDALENA
Italy	LEC - 0102<br>Svegliati	Comitato Etico Regionale delle Marche Azienda Ospedaliero-Universitaria OspedaliRiuniti di Ancona<br>Via Conca n. 71<br>60126 Torrette di Ancona<br>Tel: +39 071 596 3667<br>Email: comitato.etico@ospedaliriuniti.marche.it	Prof. Paolo PELAIA
Italy	LEC - 0103<br>Miele	Comitato Etico<br>Fondazione Policlinico Universitario A.Gemelli<br>Università Cattolica del Sacro CuoreLargo Agostino<br>Gemelli 8 00168 Roma<br>Fondazione Policlinico UniversitarioAgostino Gemelli IRCCS<br>Università Cattolica del Sacro Cuore Largo Agostino Gemelli 8, 00168<br>Roma Tel: +39 06 30155556; Fax: +39 06 30155345<br>Email: comitato.etico@policlinicogemelli.it	Prof. Gigliola SICA
Italy	LEC - 0105<br>Craxi	Comitato Etico Palermo 1Via del Vespro 129 90127<br>Palermo Tel: 0916555210; Fax: 0916553747<br>Email: bioetica@policlinico.pa.it	Prof.<br>Salvatore<br>LEONE
Italy	LEC - 0107<br>Lampertico	Comitato Etico Milano Area B Ospedale Maggiore Policlinico,Via F. Sforza n. 28 20122 Milano<br>Tel 02-55032982; Fax 02-55036618<br>Email federica.massacesi@policlinico.mi.it	Gaetana MUSERRA
Italy	LEC - 0108<br>Mangia	Comitato etico<br>Fondazione Casa Sollievo della SofferenzaIRCCS<br>Opera di San Pio da Pietrelcina 71013 San Giovanni<br>Rotondo FG Tel 0882 410831; Fax 0882410813<br>Email comitatoetico@operapaDr.epio.it	Luigi RENNA
Mauritius	1401	Name: Ethics committee<br>AdDr.ess: Bacha Building, 2nd floor,<br>PortLouis Tel: +230 52553636, +230<br>57525264<br>Fax: not available<br>email: kdhurmah@govmu.org	Dr.<br>Satyabhoosun<br>DOMAH
	CEC and LEC<br>- 0701	Komisja Bioetyczna Uniwersytetu Medycznego w Białym Stoku, Kilińskiego 1,15-	

Poland	/Flisiak (National Coordinator)/ - Site did not screen patients	089 Białystok<br>Tel: +48 85 748 54 07<br>Fax: +48 85 748 55 08<br>Email: prorektorkl@umb.edu.pl	Prof. Dr. hab. Otylia Kowal-BIELECKA
Poland	LEC - 0702 /Tomasiewicz/	Komisja Bioetyczna przy Uniwersytecie Medycznym w Lublinie, Al. Racławickie 1,20-059 Lublin;<br>Tel.: +48 81 448 52 13;<br>Fax: +48 81 448 52 11; Email:komisja.bioetyczna@umlub.pl	Dr. hab. n. med. Marcin OLAJOSSY
Poland	LEC - 0703 /Piekarska/	Komisja Bioetyki ds. Badań na ludziach przyUniwersytecie Medycznym, Pl. Hallera 1B, 90-647 Łódź;<br>Tel: +48 42 272 52 43, +48 42 272 52 44,<br>+48 785 911 596;<br>Email: bioetyka@umed.lodz.pl	Prof. Dr. hab. Józef Dr. ZEWOSKI
Poland	LEC - 0704 /Napora/	Dolnośląska Izba Lekarska we Wrocławiu Komisja Bioetyczna, Kazimierza Wielkiego45, 50- 077 Wrocław;<br>Tel. +48 71 798 80 74, +48 607 552 143;<br>Email: kb@dilnet.wroc.pl	Dr. n. med. Włodzimierz BEDNORZ
Poland	LEC - 0705 /Hartleb/	Komisja Bioetyczna Śląskiego UniwersytetuMedycznego w Katowicach, Poniatowskiego15, 40- 055 Katowice;<br>Tel. +48 32 208 36 42, +48 32 208 35 46;<br>Fax: +48 32 208 36 94;<br>Email: kombioet@sum.edu.pl	Prof. Dr. hab. n. med. Bogusław OKOPIE Ń
Slovenia	Site 1701	Republic of Slovenia National MedicalEthics Committee, Ministry of Health, Štefanova ulica 5, SI-1000 Ljubljana;<br>Tel: +386 01 478 69 13; Fax: +386 01 478 60 58;<br>Email: kme.mz@gov.si	Dr. Božidar VOLJC
Slovenia	Site 1702	Republic of Slovenia National Medical Ethics Committee, Ministry of Health, Štefanova ulica 5, SI-1000 Ljubljana; Tel:<br>+386 01 478 69 13; Fax: +386 01 478 60 58;<br>Email: kme.mz@gov.si	Dr. Božidar VOLJC
Spain	CEC - Sites 0501; 0502; 0503; 0504; 0505	CEIm Provincial de Sevilla<br>Avda. Dr. FeDr.iani, 3 – Unidad de Investigación 2ª planta Sevilla 41009<br>SevillaEspaña Tel. 600 162 458; Fax. 955 00 80 15<br>Email: administracion.eecc.hvm.sspa@juntadeandalucia.es	Dr. Víctor Sánchez MARGALET
Switzerland	CEC Site 0401	Kantonale Ethikkommission für<br>dieForschung Murtenstrasse 31<br>3010 Bern<br>Tel: +41 31 633 70 70; Fax: +41 31 633 70 71<br>Email: info.kek.kapa@gef.be.chwww.be.ch/kek	Dr. med. Christian SEILER
Switzerland	Site 0402	Commission Cantonale d'éthique de larecherche Genève<br>(CCER) Rue ADr.ien-Lachenal 8<br>1207 Genève<br>Tel: +41 22 5465101; Email: ccer@etat.ge.ch	Prof. Bernard HIRSCHEL
Switzerland	Site 0403	Comitato etico cantonale Ticinoc/o Ufficio di<br>sanità Via Orico 5<br>6501 Bellinzona<br>Tel: +41 91 814 30 57; Email: dss-ce@ti.ch	Giovan Maria ZANINI
UK	REC - Sites 0301; 0302; 0303	North East - Newcastle & North Tyneside 1Research Ethics<br>Committee NHSBT Newcastle Blood Donor CentreHolland Drive<br>Newcastle upon TyneNE2<br>4NQ Tel: 0207 104 8089<br>Email: newcastlenorthtyneside1.rec@hra nhs.uk	Mr Paddy STEVENSO N
USA	LEC - Site 1607	DUHS Institutional Review Board2424 Erwin<br>Road Durham NC 919-668-5111	John FALLETTA
USA	LEC - Site 1603	IRB for Health Sciences Research University of Virginia PO box<br>800483Charlottesville VA 434-924-9634	MEDARD H.T. Ng
USA	LEC - Site 1612	UCSD Institutional Review Boards9452 Medical Center<br>Drive La Jolla CA 858-246-4777	Kip KANTELO
USA	LEC - Site 1604	Advarra IRB<br>6940 Columbia Gateway Drive #110 Columbia<br>MD 410-884-2900	Tony DAVIS
USA	CEC - Sites 1609, 1616,1611, 1613, 1602, 1605, 1614, 1615, 1601, 1606	WIRB Copernicus IRB<br>5000 Centregreen Way STE 200Cary<br>NC 888-303-2224	Donald A. DEIESO
*The activity of the Ethics committee for medical and scientific research established at Acibadem City Clinic University hospital EOOD terminated on 15 October 2018; NA: Not applicable; UK: United Kingdom; USA: United States of America.<br>Abbreviation: CEC, Central Ethics Committee; LEC, Local Ethics Committee			

Table S2. Selection of the additional 76 patients not randomised in NATIVE, but with available liver tissue remaining from the screening period,

Among the NATIVE screening failure patients with either: i) severe fibrosis and severe activity, ii) mild fibrosis and mild activity, iii) severe fibrosis and mild activity,or iv) mild fibrosis and severe activity (black, n=207), 76 patients were chosen (red) to obtain a representative sample of mild, moderate and severe steatosis, MASH activity, and fibrosis.

Activity	Fibrosis	Steatosis								Total available	Total selected
		S0		S1		S2		S3			
A0	F0	16	**8**	10	**7**	2	**2**	2	**2**	30	**19**
A1	F0	2		10		12		9		33	**0**
A2	F0	0		7	**1**	11	**2**	9	**2**	27	**5**
A2	F1	0		8	**1**	21	**4**	29	**5**	58	**10**
A3	F0	0		0		0		0		0	**0**
A3	F1	0		0		1	**1**	4	**4**	5	**5**
A0	F2	0		0		0		0		0	**0**
A1	F2	1	**1**	6	**6**	7	**7**	0		14	**14**
A0	F3	1	**1**	1	**1**	0		0		2	**2**
A1	F3	0		2	**2**	1	**1**	0		3	**3**
A3	F2	0		3		2		12	**5**	17	**5**
A3	F3	0		2		3		4	**4**	9	**4**
A4	F2	0		0		0		5	**5**	5	**5**
A4	F3	0		0		2	**2**	2	**2**	4	**4**
**TOTAL**										207	**76**

Table S3. Clinical features at baseline.

Parameter	Screening failures N=76	Randomized patients			
		All N=173	Placebo N=56	Lanifibranor 800mg N=57	Lanifibranor 1200mg N=60
**Demographic and clinical characteristics**					
**Age – yrs**	54 (45; 62)	56 (49; 62)	54 (46; 62)	58 (50; 63)	56 (49; 62)
**Female sex – no. (%)**	37 (49%)	100 (58%)	29 (52%)	34 (60%)	37 (62%)
**Weight – kg**	87 (79; 99)	92 (81; 107)	93 (82; 107)	94 (81; 106)	89 (79; 108)
**Body-mass index – kg/m²**	31.1 (28.1; 35.3)	32.9 (29.4; 36.3)	33.3 (29.4; 36.6)	32.2 (29.4; 35.9)	33.3 (29.6; 37.5)
**Body-mass index > 25 (%)**	68 (89%)	164 (95%)	52 (93%)	55 (96%)	57 (95%)
**Body-mass index > 30 (%)**	41 (54%)	120 (69%)	39 (70%)	37 (65%)	44 (73%)
**Arterial hypertension – %**	34 (45%)	106 (61%)	31 (55%)	39 (68%)	36 (60%)
**Type 2 diabetes mellitus – no. (%)**	28 (37%)	72 (42%)	22 (39%)	25 (44%)	25 (42%)
**Time between screening biopsy and randomization - months**	NA	2.3 (1.3; 3.5)	2.2 (1.3; 3.4)	2.0 (1.2; 3.1)	2.4 (1.5; 3.6)
**Histological parameters**					
**Steatosis grade***	2 (1; 3)	3 (2; 3)	3 (2; 3)	3 (2; 3)	3 (2; 3)
**S0 – no. (%)**	10 (13%)	0 (0%)	0 (0%)	0 (0%)	0 (0%)
**S1 – no. (%)**	18 (24%)	15 (9%)	4 (7%)	5 (9%)	6 (10%)
**S2 – no. (%)**	19 (25%)	45 (26%)	17 (30%)	11 (19%)	17 (28%)
**S3 – no. (%)**	29 (38%)	113 (65%)	35 (63%)	41 (72%)	37 (62%)
**Lobular inflammation grade****	1 (0; 1)	1 (1; 2)	2 (1; 2)	2 (1; 2)	1 (1; 2)
**I0 – no. (%)**	25 (33%)	0 (0%)	0 (0%)	0 (0%)	0 (0%)
**I1 – no. (%)**	35 (46%)	88 (51%)	27 (48%)	28 (49%)	33 (55%)
**I2 – no. (%)**	14 (18%)	76 (44%)	27 (48%)	26 (46%)	23 (38%)
**I3 – no. (%)**	2 (2%)	9 (5%)	2 (4%)	3 (5%)	4 (7%)
**Ballooning grade*****	1 (0; 1)	2 (2; 2)	2 (2; 2)	2 (1; 2)	2 (2; 2)
**B0 – no. (%)**	35 (46%)	0 (0%)	0 (0%)	0 (0%)	0 (0%)
**B1 – no. (%)**	23 (30%)	40 (23%)	12 (21%)	17 (30%)	11 (18%)
**B2 – no. (%)**	18 (24%)	133 (77%)	44 (79%)	40 (70%)	49 (82%)
**Fibrosis stage******	1 (0; 2)	2 (2; 3)	2 (1; 3)	2 (2; 3)	2 (2; 3)
**F0 – no. (%)**	24 (32%)	4 (2%)	3 (5%)	0 (0%)	1 (1%)
**F1 – no. (%)**	15 (20%)	36 (21%)	13 (23%)	10 (18%)	13 (22%)
**F2 – no. (%)**	23 (30%)	85 (49%)	26 (46%)	32 (56%)	27 (45%)
**F3 – no. (%)**	14 (18%)	48 (28%)	14 (25%)	15 (26%)	19 (32%)
**Fibrosis stage F2 or F3 – no. (%)**	37 (49%)	133 (77%)	40 (71%)	47 (82%)	46 (77%)
**SAF-A score**	2 (0; 3)	3 (3; 4)	3 (3; 4)	3 (3; 3)	3 (3; 4)
**NAS**	3 (2; 6)	6 (5; 6)	6 (5; 6)	6 (6; 6)	6 (5; 6)

**NAS≥6 no. (%)**	22 (29%)	123 (71%)	39 (70%)	43 (75%)	41 (68%)
**Plasma biochemistry parameters**					
**Serum alanine aminotransferase – IU/L**	41 (27; 63)	50 (35; 76)	43 (32; 76)	50 (38; 75)	52 (35; 79)
**Serum aspartate aminotransferase – IU/L**	28 (23; 40)	37 (29; 54)	35 (26; 51)	37 (29; 56)	41 (29; 55)
**Serum γ-Glutamyltransferase– IU/L**	46 (31; 76)	44 (30; 69)	44 (29; 71)	48 (31; 79)	39 (31; 59)
**Fasting HDL cholesterol level – mmol/L**	1.08 (0.91; 1.44)	1.17 (0.99; 1.40)	1.14 (0.92; 1.30)	1.18 (1.07; 1.43)	1.16 (0.98; 1.42)
**Fasting triglycerides – mmol/L**	1.81 (1.27; 2.59)	1.76 (1.32; 2.41)	1.93 (1.38; 2.44)	1.70 (1.35; 2.30)	1.63 (1.29; 2.54)
**Fasting glucose level – mmol/L**	5.39 (4.89; 6.29)	5.66 (5.07; 6.58)	5.37 (5.11; 6.43)	6.00 (5.07; 7.16)	5.65 (5.03; 6.36)
**Glycated hemoglobin level – %**	6 (5; 6)	6 (6; 7)	6 (5; 6)	6 (6; 7)	6 (6; 7)
**Fasting insulin level – pmol/L**	NA	190.8 (141.5; 261.3)	180.3 (132.0; 224.3)	196.5 (145.8; 251.2)	197.6 (131.4; 314.3)
**Cytokeratin 18 M65 – pmol/L**	NA	460 (248 ; 817)	336 (248 ; 675)	503 (334 ; 858)	512 (248, 911)
**FIB-4 score**	0.97 (0.74 ; 1.34)	1.20 (0.79 ; 1.97)	1.14 (0.65 ; 1.90)	1.37 (0.94 ; 2.0)	1.09 (0.73 ; 1.77)
**FIB-4 classes**<br>**≤ 1.3**<br>**> 1.3 and ≤ 3.25**<br>**> 3.25**	 55 (72%)<br>18 (24%)<br>3 (4%)	 95 (55%)<br>72 (42%)<br>5 (3%)	 34 (61%)<br>20 (36%)<br>2 (4%)	 27 (47%)<br>28 (49%)<br>2 (4%)	 34 (58%)<br>24 (41%)<br>1 (2%)
**FibroScan® values**					
**LSM - kPa**	6.8 (5.7; 9.7)	8.8 (6.8; 11.6)	8.6 (6.9; 10.8)	9.9 (7.8; 11.7)	8.4 (6.1; 12.0)
**CAP- dB m^{-1}**	322 (279; 361)	328 (300; 359)	325 (301; 376)	330 (295; 354)	327 (297; 357)
**Treatments**					
**At least one antidiabetic drug**	29 (38%)	64 (37%)	21 (38%)	22 (39%)	21 (35%)
**Statin (alone or in combination) – no. (%)**	13 (17%)	32 (18%)	8 (14%)	11 (19%)	13 (22%)

If not stated otherwise, data is presented as median (interquartile range).
*Steatosis was assessed as the percentage of hepatocytes containing large and medium-sized intracytoplasmic lipid droplets and graded as 0 (<5%), 1 (5 to 33%), 2 (34 to 66%), or 3 (≥67%), according to the Nonalcoholic Steatohepatitis Clinical Research Network (NASH CRN) grading system.
**Lobular inflammation was classified as grade 0 (no foci), grade 1 (<2 foci per 200x field) or grade 2 (2-4 foci per 200x field), according to the NASH CRN scoring system.
***Ballooning was classified as grade 0 (no balloon hepatocyte) grade 1 (few but definite ballooned hepatocytes) or grade 2 (prominent ballooning), according to the NASH CRN grading system.

****Fibrosis was classified as stage F0 (no fibrosis), stage F1 (mild fibrosis), stage F2 (significant fibrosis), stage F3 (advanced fibrosis), or stage F4 (cirrhosis), according to the SAF–NASH CRN staging system.

Abbreviations: SAF-A: The SAF-Activity (SAF-A) score ranges from 0 to 4; with higher scores indicating more-severe disease activity; NAS: The Nonalcoholic Fatty Liver Disease Activity Score (NAS) ranges from 0 to 8. A score of 2 or less indicates “not NASH”; a score of 3 or 4, “borderline NASH” and a score of 5 to 8, “definite NASH”. LSM: Liver Stiffness Measure assessed by FibroScan®; CAP: Controlled Attenuation Parameter assessed by FibroScan®.

Table S4: Relationship between clinical parameters and density of CD34 positive vessels, periportal score and lobular score at baseline

Parameter	Density of CD34 positive vessels		Periportal score		Lobular score	
	Test	p-value	Test	p-value	Test	p-value
**Demographic and clinical characteristics**						
**Age**	Sp: r: 0.064	0.312	W	0.459	W	0.268
**Sex**	W	0.806	Chi²	0.698	Chi²	0.137
**Weight**	Sp: r: -0.062	0.333	W	0.484	W	0.312
**Body Mass Index**	Sp: r: -0.087	0.172	W	0.995	W	0.239
**Body Mass Index ≥25**	W	0.89	F	0.581	Chi²	0.805
**Body Mass Index ≥30**	W	0.646	Chi²	0.406	Chi²	0.495
**All classes Body Mass Index**	KW	0.218	CA	0.766	CA	0.183
**Arterial hypertension**	W	0.183	Chi²	0.704	Chi²	0.615
**Type 2 Diabetes Mellitus**	W	0.968	Chi²	0.214	Chi²	0.875
**Plasma biochemistry parameters**						
**Serum AST**	Sp: r:0.161	**0.011**	W	0.668	W	**0.006**
**Serum ALT**	Sp: r: 0.119	0.062	W	0.609	W	**0.013**
**Serum γ-Glutamyltransferase**	Sp: r: 0.131	**0.039**	W	0.978	W	0.147
**Fasting HDL cholesterol**	Sp: r: -0.003	0.961	W	0.757	W	0.164
**Fasting triglycerides**	Sp: r: 0.073	0.256	W	0.901	W	0.532
**Fasting glucose**	Sp: r: 0.037	0.563	W	0.951	W	0.672
**Glycated hemoglobin**	Sp: r: 0.049	0.443	W	0.72	W	0.471
**Fasting insulin level***	Sp: r: -0.011	0.888	W	0.259	W	0.663
**CK18 M65***	Sp: r: 0.125	0.105	W	0.663	W	**0.046**
**FIB-4**	Sp: r: 0.164	**0.01**	W	0.835	W	0.136
**FIB-4 classes**	W	**0.004**	CA	0.792	CA	0.064
**FibroScan® values**						
**CAP**	Sp: r: -0.028	0.704	W	0.085	W	0.427
**LSM**	Sp: r: 0.212	**0.002**	W	0.439	W	0.094
**Treatments**						
**Anti-diabetic drugs**	W	0.816	Chi²	0.192	Chi²	0.511
**Statins**	W	0.811	Chi²	0.747	Chi²	0.646

Density of CD34 positive vessels, of periportal score and of lobular score were available in 248, 246 and 245 patients, respectively, as detailed in Fig S3. P-values are displayed for the following tests: CA: Cochran-Armitage test for trend (ordered vs. 2 modalities categorial parameters); Chi^2: categorial parameters (all expected values > 5); F: Fisher test (categorial parameters, at least one expected value ≤ 5); Sp: Spearman correlation test (continuous), r: Spearman correlation coefficient; W: Wilcoxon (continuous non-normally distributed vs. 2

modalities categorial parameters) (non-N distribution; KW); * Data available only for the 173 randomized patients.

AST, Aspartate aminotransferase; ALT, Alanine Aminotransferase; HDL, high density lipoprotein; CK18, cytokeratin 18; CAP, controlled attenuation parameter assessed by Fibroscan®; LSM, liver stiffness measurement assessed by FibroScan®. Bold indicates p < 0.05. Colors represent the p-value, with red (0) to green (1).

Table S5. Baseline characteristics and haemodynamics of rats fed a chow – or methionine-choline-deficient diet.

**Chow diet-fed rats**	Groups (n/n/n/n/n)	Placebo	Fenofibrate (30mg/kg)	GW501516 (10 mg/kg)	Rosiglitazone (5 mg/kg)	Lanifibranor (100 mg/kg)
Age (weeks)	58/38/38/38/38	8	8	8	8	8
Body weight (g): baseline	50/24/38/38/38	251.0 (242.8 – 263.3)	257.0 (248.5 – 267.0)	250.5 (247.0 – 258.0)	253.0 (244.8 – 257.3)	249.5 (243.0 – 257.0)
Body weight (g): after 4 weeks	58/38/37/37/38	349.0 (328.3 – 360.3)*	340.0 (319.8 – 354.0)#	330.0 (316.5 – 340.5)#	331.0 (321.0 – 350.0)#	338.5 (326.3 – 373.0)#
∆ Weight (g)	50/24/37/37/38	92.5 (72.0 – 104.5)*	79.0 (69.0 – 85.8)#	78.0 (67.0 – 86.0)#	79.0 (69.5 – 93.5)#	87.0 (69.3 – 103.3)#
Liver weight (g)	54/38/37/38/38	10.5 (9.3 – 11.3)	12.6 (11.3 – 14.5)°	11.5 (10.5 – 12.9)	9.9 (8.5 – 11.2)	10.4 (9.6 – 11.1)
% liver/total body weight	56/38/37/37/38	3.0 (2.9 – 3.2)*	4.6 (4.4 – 5.1)°	3.5 (3.2 – 4.0)#	2.9 (2.6 – 3.2)#	3.0 (2.9 – 3.2)#
MABP (mmHg)	43/26/28/29/31	123.7 (106.3 – 130.8)	144.9 (129.8 – 153.2)°	110.1 (100.0 – 121.5)	122.6 (96.9 – 134.3)	110.7 (101.1 – 122.1)#
In vivo PVP (mmHg)	46/26/27/28/31	3.5 (3.2 – 3.9)*	3.7 (3.4 – 4.1)	3.5 (3.1 – 3.9)#	3.5 (3.2 – 3.6)#	3.3 (3.1 – 3.7)
Portal blood flow (mL/min)	38/25/26/26/28	12.5 (11.0 – 15.1)*	13.5 (11.3 – 14.8)	12.7 (11.4 – 14.3)	12.6 (10.9 – 15.8)	13.8 (12.1 – 15.1)#
THPG (mmHg) at 10 mL/min	8/8/8/8/8	3.7 ± 0.1	3.7 ± 0.2	3.5 ± 0.1	3.8 ± 0.2	3.5 ± 0.1
THPG (mmHg) at 30 mL/min	8/8/8/8/8	6.6 ± 0.2	7.0 ± 0.4	6.5 ± 0.3	6.7 ± 0.6	7.4 ± 0.4

**MCDD-fed rats**	Groups (n/n/n/n/n)	Placebo	Fenofibrate (30 mg/kg)	GW501516 (10 mg/kg)	Rosiglitazone (5 mg/kg)	Lanifibranor (100 mg/kg)
Age (weeks)	64/38/38/38/38	8	8	8	8	8
Body weight (g): baseline	50/24/38/38/38	251.0 (242.8 – 263.3)	260.0 (228.3 – 274.8)	250.0 (247.8 – 258.0)	251.5 (240.8 – 259.8)	250.0 (243.5 – 258.0)
Body weight (g): after 4 weeks	62/38/37/38/38	210.0 (202.0 – 219.3)	205.0 (198.0 – 211.8)	211.0 (202.0 – 218.0)	213.0 (208.0 – 220.3)	211.0 (203.8 – 215.0)
Δ Weight (g)	50/24/38/38/38	-43.5 (-50.0 – -38.75)	-50.5 (-60.0 – -37.5)	-42.0 (-44.5 – -36.8)	-36.0 (-44.0 – -29.0)	-43.5 (-46.0 – -35.0)
Liver weight (g)	60/38/37/38/37	10.5 (9.3 – 11.3)	8.1 (7.6 – 8.7)*	11.4 (9.7 – 13.4)	9.5 (8.8 – 9.9)	9.7 (9.2 – 11.1)
% liver/total body weight	60/38/37/38/37	4.6 (4.4 – 5.1)	3.9 (3.7 – 4.2)*	5.3 (4.8 – 6.1)	4.4 (4.1 – 4.6)	4.7 (4.4 – 5.1)
MABP (mmHg)	40/28/29/25/37	121.9 (115.3 – 132.3)	120.0 (110.0 – 134.2)	100.5 (83.7 – 109.7)*	118.1 (100.5 – 127.1)	92.7 (78.1 – 104.1)*
In vivo PVP (mmHg)	40/27/26/24/37	5.6 (5.1 – 6.4)	4.5 (3.5 – 5.3)*	4.6 (3.9 – 4.9)	4.8 (4.1 – 5.5)	3.7 (3.2 – 4.0)*
Portal blood flow (mL/min)	34/27/25/23/34	10.1 (9.2 – 11.3)	10.8 (9.5 – 12.6)	11.7 (10.4 – 13.4)	11.1 (9.8 – 14.0)	10.8 (9.2 – 12.7)
THPG (mmHg) at 10 mL/min	7/8/8/8/8	4.8 ± 0.2	3.2 ± 0.2*	4.0 ± 0.2	4.3 ± 0.3	3.5 ± 0.1*
THPG (mmHg) at 30 mL/min	7/8/8/8/8	8.3 ± 0.4	6.6 ± 0.2*	6.7 ± 0.3	8.0 ± 1.0	6.3 ± 0.3*

Male Wistar Han rats of 8 weeks old (n = 6-8/group) were either fed a chow diet (CD) or a methionine-choline-deficient diet (MCDD) for 4 weeks and simultaneously treated with either placebo, fenofibrate (30 mg/kg), GW501516 (10 mg/kg), rosiglitazone (5 mg/kg) or lanifibranor (100 mg/kg) daily QD via oral gavage. Pooled data were analysed using Kruskal-Wallis followed by Dunn test and presented as median (IQR). The THPG data were analysed using a generalised estimating equation model followed by least significant difference post hoc testing when appropriate. * for comparison with MCDD + placebo; ° CD + treatment vs. CD + placebo; # CD + treatment vs. MCDD + treatment counterparts. */°/#: $p<0.05$. MABP, mean arterial blood pressure; PVP, portal venous pressure; THPG, transhepatic pressure gradient.

Table S6. Blinded semi-quantification in early MASLD of CD34 liver sinusoidal endothelial marker immunostained sections.

8 weeks old rats fed chow- (CD) or methionine - choline - deficient diet (MCDD) for 4 weeks were preventively treated with placebo, fenofibrate (30 mg/kg), GW501516 (10 mg/kg), rosiglitazone (5 mg/kg) or lanifibranor (100 mg/kg). n = 5-6/group. Data presented as mean ± SEM. Two-way ANOVA was performed followed by post hoc Tukey, with * p<0.05; **: p<0.01; ***: p<0.001. p* towards placebo-treated MCDD.

CD34 quantification	Placebo	Lanifibranor	Fenofibrate	GW501516	Rosiglitazone
CD	20.9 ± 2.8**	14.3 ± 2.5	8.5 ± 2.0	10.2 ± 3.1	13.5 ± 1.6
MCDD	38.8 ± 2.5	21.4 ± 5.7*	12.7 ± 4.0****	16.5 ± 3.3***	34.5 ± 4.2

Table S7. *In situ ex vivo* liver perfusion in early MASLD: relative change of transhepatic pressure gradient at a dose of 3 × 10^{-9} mol/L ET-1.

Male Wistar Han rats of 8 weeks old were either fed a chow diet (CD) or a methionine-choline-deficient diet (MCDD) for 4 weeks and simultaneously treated with either placebo, fenofibrate (30 mg/kg), GW501516 (10 mg/kg), rosiglitazone (5 mg/kg) or lanifibranor (100 mg/kg) daily, QD via oral gavage. n = 7-8/group). The THPG data were analysed using a generalised estimating equation model followed by least significant difference post hoc testing. Data presented as mean ± SEM.* for comparison with MCDD + placebo; ° CD + treatment vs. CD + placebo. */°: $p<0.05$. M, molar. ET-1, endothelin-1.

ET-1 dose-response	Group (n)	ΔTHPG at 3 x 10^{-9} M ± SEM (mmHg)
CD	Placebo (11)	12.0 ± 1.5*
	Fenofibrate (8)	20.5 ± 1.4°
	GW501516 (7)	9.7 ± 1.1
	Rosiglitazone (8)	7.9 ± 1.2°
	Lanifibranor (8)	10.7 ± 1.1
MCDD	Placebo (11)	17.4 ± 1.4
	Fenofibrate (8)	21.3 ± 1.1*
	GW501516 (8)	14.4 ± 1.8
	Rosiglitazone (8)	15.4 ± 1.5
	Lanifibranor (8)	15.1 ± 1.2

Table S8. *In situ ex vivo* liver perfusion in early MASLD: EC50 and Emax from dose-response curves with methoxamine.

Male Wistar Han rats of 8 weeks old were either fed a chow diet (CD) or a methionine-choline-deficient diet (MCDD) for 4 weeks and simultaneously treated with either placebo, fenofibrate (30 mg/kg), GW501516 (10 mg/kg), rosiglitazone (5 mg/kg) or lanifibranor (100 mg/kg) daily QD via oral gavage. n = 7-11/group). Nonlinear regression (curve fit) was performed to calculate EC50 and Emax. Data were analysed using two-way ANOVA followed by post hoc Tukey and presented as mean ± SEM. * for comparison with MCDD + placebo. *: p<0.05. M, molar.

Methoxamine dose-response	Group (n)	EC50 ± SEM (M)	Emax ± SEM (mmHg)
CD	Placebo (8)	1.8e-005 ± 2.1e-006	7.8 ± 0.6*
	Fenofibrate (8)	2.2e-005 ± 1.8e-006	7.6 ± 0.7
	GW501516 (6)	2.0e-005 ± 3.1e-006	8.5 ± 1.3
	Rosiglitazone (8)	1.5e-005 ± 1.9e-006	6.5 ± 0.4
	Lanifibranor (8)	2.8.e-005 ± 6.3e-006	7.7 ± 1.0
MCDD	Placebo (12)	1.2e-005 ± 8.0e-007	10.8 ± 0.9
	Fenofibrate (8)	1.9e-005 ± 2.6e-006	9.7 ± 0.7
	GW501516 (8)	2.2e-005 ± 2.5e-006	9.3 ± 1.2
	Rosiglitazone (8)	1.8e-005 ± 2.1e-006	8.8 ± 1.0
	Lanifibranor (8)	1.7e-005 ± 2.7e-006	8.7 ± 1.3

Table S9. *In situ ex vivo* liver perfusion in MASH: EC50 and Emax from dose-response curves with methoxamine.

8 weeks old male Zucker fatty rats fed a high-fat high-fructose (HFHFD) diet and 8 weeks old male Zucker lean rats fed a chow diet (CD) were preventively treated with either placebo or lanifibranor (100 mg/kg) daily QD via oral gavage during the complete period of 8 weeks of diet. Data is presented as mean ± SEM. Nonlinear regression (curve fit) was performed to calculate EC50 and Emax, followed by two-way ANOVA and post hoc Tukey with *: p<0.05. * for comparison with MCDD + placebo.

Methoxamine dose-response	Group (n)	EC50 ± SEM (M)	Emax ± SEM (mmHg)
CD	Placebo (8)	6.4e-005 ± 1.4e-005	8.2 ± 0.5
	Lanifibranor (8)	6.5e-005 ± 1.5e-005	8.7 ± 0.9
HFHFD	Placebo (8)	7.7e-005 ± 2.1e-005	7.8 ± 0.7
	Lanifibranor (7)	6.3e-005 ± 9.3e-006	5.8 ± 0.9*

Table S10. ***In situ ex vivo*** **liver perfusion in early MASLD: % vascular relaxation with acetylcholine at i.e. 10^{-5} mol/L and 10^{-3} mol/L.**

Male Wistar Han rats of 8 weeks old were either fed a chow diet (CD) or a methionine-choline-deficient diet (MCDD) for 4 weeks and were simultaneously treated with either placebo, fenofibrate (30 mg/kg), GW501516 (10 mg/kg), rosiglitazone (5 mg/kg) or lanifibranor (100 mg/kg) daily, QD via oral gavage. n = 7-13/group). Data is presented as mean ± SEM. The THPG data were analysed using a generalised estimating equation model followed by least significant difference post hoc testing with *: p<0.05. M, molar. * for comparison with MCDD + placebo.

Acetylchlonine dose-response	Group (n)	Vascular relaxtion (%) at 10^{-5} M ± SEM	Vascular relaxtion (%) at 10^{-3} M ± SEM
CD	Placebo (10)	-26.0 ± 7.3	-61.4 ± 5.6*
	Fenofibrate (8)	-5.1 ± 5.4	-64.0 ± 15.3
	GW501516 (7)	-17.3 ± 3.4	-60.3 ± 5.6
	Rosiglitazone (7)	-21.3 ± 5.3	-63.9 ± 5.6
	Lanifibranor (6)	-15.8 ± 8.5	-72.9 ± 4.3
MCDD	Placebo (11)	-11.3 ± 5.4	-39.7 ± 4.5
	Fenofibrate (8)	-11.2 ± 5.9	-53.9 ± 6.9
	GW501516 (7)	-18.4 ± 8.6	-53.4 ± 10.2
	Rosiglitazone (7)	-15.7 ± 6.2	-58.3 ± 4.2*
	Lanifibranor (10)	-29.0 ± 6.7	-60.5 ± 6.1*

Table S11. *In situ ex vivo* liver perfusion in MASH: vascular relaxation with acetylcholine at doses of i.e. 10^{-5} mol/L and 10^{-3} mol/L.

8 weeks old male Zucker fatty rats fed a high-fat high-fructose (HFHFD) diet and 8 weeks old male Zucker lean rats fed a chow diet (CD) were preventively treated with either placebo or lanifibranor (100 mg/kg) daily QD via oral gavage during the complete period of 8 weeks of diet. n = 7-8/group. Data is presented as mean ± SEM. The THPG data were analysed using a generalised estimating equation model followed by least significant difference post hoc testing with *: p<0.05. M, molar. * for comparison with MCDD + placebo.

Acetylchlonine dose-response	Group (n)	Vascular relaxtion (%) at 10^{-5} mol/L	Vascular relaxtion (%) at 10^{-3} mol/L
CD	Placebo (8)	-14.6 ± 5.5*	-42.3 ± 6.5
	Lanifibranor (8)	-13.6 ± 3.1	-45.1 ± 5.0
HFHFD	Placebo (8)	-2.2 ± 2.5	-41.5 ± 4.3
	Lanifibranor (8)	-14.7 ± 1.3*	-40.7 ± 4.7

Supplementary references

[1] Kleiner DE, Brunt EM, Van Natta M, et al. Design and validation of a histological scoring system for nonalcoholic fatty liver disease. Hepatology 2005;41:1313-1321.
[2] **Haber MA, Iranmahboob A**, Thomas C, et al. ERG is a novel and reliable marker for endothelial cells in central nervous system tumors. Clin Neuropathol 2015;34:117-127.
[3] **Francque S, Laleman W**, Verbeke L, et al. Increased intrahepatic resistance in severe steatosis: endothelial dysfunction, vasoconstrictor overproduction and altered microvascular architecture. Lab Invest 2012;92:1428-1439.
[4] **Van der Graaff D, Kwanten WJ,** Couturier FJ, et al. Severe steatosis induces portal hypertension by systemic arterial hyporeactivity and hepatic vasoconstrictor hyperreactivity in rats. Lab Invest 2018;98:1263-1275.
[5] Boubia B, Poupardin O, Barth M, et al. Design, Synthesis, and Evaluation of a Novel Series of Indole Sulfonamide Peroxisome Proliferator Activated Receptor (PPAR) α/γ/δ Triple Activators: Discovery of Lanifibranor, a New Antifibrotic Clinical Candidate. Journal of Medicinal Chemistry 2018;61:2246-2265.
[6] Wettstein G, Luccarini JM, Poekes L, et al. The new-generation pan-peroxisome proliferator-activated receptor agonist IVA337 protects the liver from metabolic disorders and fibrosis. Hepatol Commun 2017;1:524-537.
[7] van der Graaff D, Chotkoe S, De Winter B, et al. Vasoconstrictor antagonism improves functional and structural vascular alterations and liver damage in rats with early NAFLD. JHEP Rep 2022;4:100412.
[8] European Association for Study of L, Asociacion Latinoamericana para el Estudio del H. EASL-ALEH Clinical Practice Guidelines: Non-invasive tests for evaluation of liver disease severity and prognosis. J Hepatol 2015;63:237-264.

Bold indicates co-first authors
